# Visible-light-responsive indoleazopyrazole photoswitches: dual enhancement of redshift and half-life by ester modification at the *ortho* position

**DOI:** 10.1039/d5sc03275j

**Published:** 2025-07-21

**Authors:** Xuanchi Yu, Chenyu Zhang, Dongfang Dong, Bing Liu, Dali Wang, Tao Li

**Affiliations:** a School of Chemistry and Chemical Engineering, Shanghai Jiao Tong University Shanghai 200240 China litao1983@sjtu.edu.cn energywang@sjtu.edu.cn; b State Key Laboratory of Synergistic Chem-Bio Synthesis, Shanghai Key Laboratory of Electrical Insulation and Thermal Aging, Shanghai Jiao Tong University Shanghai 200240 China; c Department of Oral and Maxillofacial Surgery, The First Affiliated Hospital of Harbin Medical University Harbin 150001 China; d Zhang Jiang Institute for Advanced Study, Shanghai Jiao Tong University Shanghai 200240 China

## Abstract

As a class of universal light-responsive units, most azo compounds require ultraviolet (UV) excitation. Most conventional π → π* redshift strategies, while enabling visible-light excitation, often compromise the thermal stability of the *Z*-isomer. Herein, we designed a series of *ortho*-substituted indoleazopyrazoles that simultaneously achieve visible-light responsiveness and exceptional thermal stability. Notably, ester substitution at the *ortho* position (relative to the azo group) of the indoleazopyrazole exhibits a *λ*_max_ (π → π*) redshift to 383 nm while maintaining a half-life of up to 4.7 days. Following water-soluble modification, the optimized ester substitution derivative 5-photosurfactant (5-PS) demonstrates visible-light-controlled bioactivity, switching between low toxicity (*E*-isomer) and high toxicity (*Z*-isomer) in three human cancer cell lines. Remarkably, the half-maximal inhibitory concentration (IC_50_) of the *E*-isomer is approximately threefold higher than that of the *Z*-rich isomer in HepG2 cells. This strategy achieves the dual enhancement of π → π* redshift and half-life, opening a new avenue for visible-light-controlled targeted anticancer therapy.

## Abstract 2

Through ester modification at the *ortho* position, a dual enhancement of π → π* redshift and extended half-life was achieved. After modification, the compound exhibited potential for visible-light controlled anticancer therapy.

## Graphical abstract

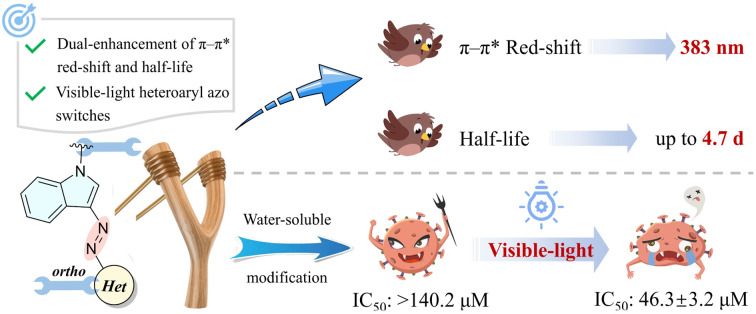

## Introduction

Azo-based photoswitches are widely employed as photocontrol modules in photopharmacology,^1–5^ light-controllable catalysts,^6–8^ smart materials^9–13^ and energy storage systems,^14–20^ owing to their facile synthesis, substantial geometrical configuration changes during isomerization, and remarkable photoswitching properties.^21–23^ However, since the *λ*_max_ (π → π*) absorption of *E*-isomers falls within the UV region, most azo compounds require high-energy photons for isomerization in at least one direction. The limited penetration depth of UV light^24^ and its potential detrimental effects on living organisms^25–27^ have restricted its broader application. Therefore, the development of visible-light-responsive azo systems is of urgent importance.^28–35^

Two molecular modification strategies have been employed to design visible-light photoswitches. (1) Separation of the *E-* and *Z*-isomers' *n* → π* absorption bands in the visible-light region. As an elegant approach to integrating visible-light responsiveness with extended half-life, this strategy has seen limited success, with notable examples including *ortho*-substitutions (–F,^30,36^ –Cl,^37,38^ –Br,^39,40^ –OMe^39,41^), diazocines^29,42,43^ and BF_2_-coordinated azo compounds.^31,44,45^ Due to inherent constraints on modification sites, the structural influence on *n* → π* band separation requires further evaluation. (2) Extension of π-conjugation system to lower the HOMO–LUMO gap of *E*-isomer and redshift the π → π* absorption band. Although this represents a more straightforward design, strategy (2) exhibits inherent drawbacks, as redshifting the *λ*_max_ (π → π*) of the *E*-isomer typically shortens the *Z*-isomer's thermal half-life. For instance, the Fuchter group developed 2-arylazoimidazole with a half-life of 2.95 h, despite the π → π* absorption redshift to 385 nm.^46^ Similarly, the Tamaoki group reported *para*-methoxy-substituted phenylazothiazole with a *λ*_max_ (π → π*) at 385 nm and a *t*_1/2_ of 14.8 min.^34^ Fused-ring incorporation has also been utilized to redshift the absorption maximum, as in the arylazobenzimidazole system, where *λ*_max_ (π → π*) occurs around 380 nm.^47^ However, such modification drastically shortens the *Z*-isomers' thermal half-lives, ranging from minutes to hours. Therefore, it is crucial to design visible-light-responsive azo molecules to address the inverse correlation between the thermal *t*_1/2_ and the redshift of *λ*_max_ (π → π*).

The strategic combination of heteroaryl rings can effectively mitigate this unfavorable effect. From two distinct perspectives, an electron-rich heteroaryl ring contributes to a pronounced redshift of the π → π* transition,^33,34,47–50^ while the presence of heteroatom-involved intramolecular interactions further stabilizes the *Z*-isomer, thereby increasing its thermal half-life.^46,51^ Our interest was to investigate indole-based photoswitches which due to their electron–rich properties extend the π-conjugated system, thereby facilitating a spectral redshift. Meanwhile, indole has been widely investigated as a key structural component in nitrogen-containing heterocyclic drug molecules.^52^ Therefore, indole-based photoswitches hold significant promise for visible-light-responsive photopharmacology studies. Previously, the König group reported that phenylazoindole achieved 61% *E* → *Z* isomerization in acetonitrile (MeCN) and 85% in dimethyl sulfoxide (DMSO) under irradiation at 400 nm.^50^ However, the *Z* → *E* transition relies on thermal relaxation due to the spectral overlap of the tail absorption bands of both isomers. To achieve efficient bidirectional isomerization under visible light while maintaining the half-life, we envisioned the incorporation of an additional heteroaryl moiety. Azopyrazole, known for its long half-life (attributed to potential intramolecular C–H⋯π interaction in the *Z-*isomer) and multiple twisted configurations, has been widely utilized.^20,33,46,51,53^ Additionally, pyrazole is frequently employed as a bioisostere to enhance potency and optimize physicochemical properties, including water solubility.^52,54,55^ Thus, combining pyrazole with azoindole offers the possibility of bidirectional visible-light isomerization, circumvents limitations with *Z* → *E* thermal relaxation, and enhances both the system's hydrophilicity and its potential for photopharmacological applications.

**Scheme 1:**
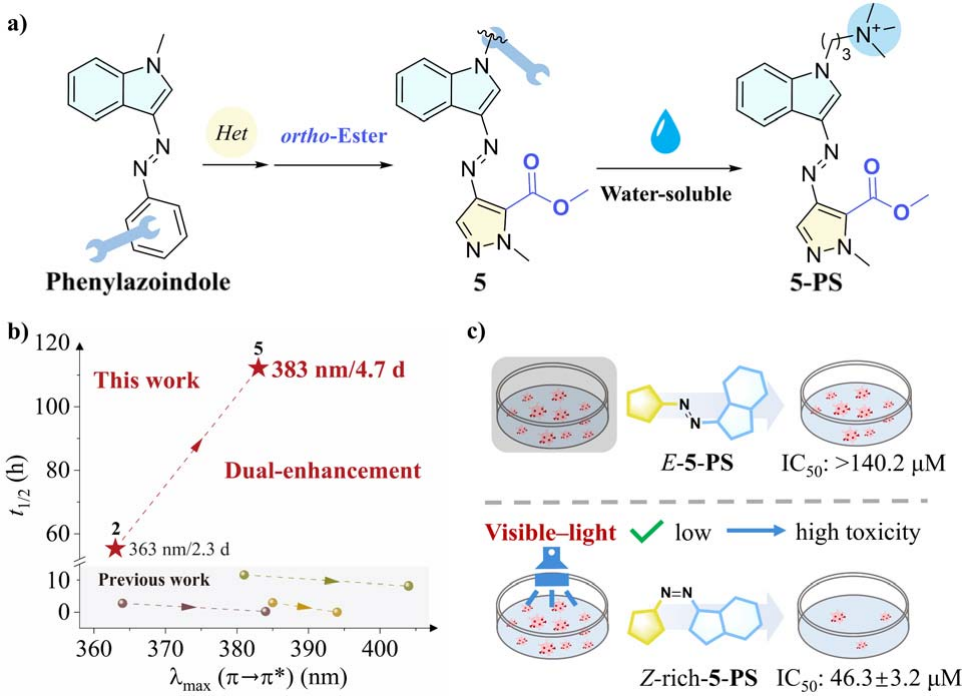
(a) Modification strategies for indoleazopyrazoles. (b) Correlation between *λ*_max_ (π → π*) and half-life (previous work: ref. 34 – purple dots and dotted line; ref. 46 – yellow dots and dotted line; ref. 47 – green dots and dotted line). (c) Photocontrolled cytotoxicity of 5-PS to HepG2 cells (data represent three independent experiments as mean ± SD (*n* = 3)).

Herein, through heteroaryl incorporation and *ortho* modifications, we developed a series of indoleazopyrazoles as visible-light-responsive photoswitches, exhibiting excellent bidirectional isomerization efficiency and extended thermal half-lives of several days, thereby overcoming the deficiency of *Z* → *E* photoisomerization in phenylazoindole systems that rely on thermal recovery. Notably, compound 5 demonstrated excellent photoswitching properties (*λ*_max_ (π → π*): 383 nm; half-life: 4.7 d; bidirectional isomerization ratio: 86.0%/96.2%), successfully achieving a dual enhancement of redshift and half-life (Scheme 1). In addition, given the favourable biological activities of indole and pyrazole rings, the water-soluble derivative 5-PS was evaluated as a potential drug scaffold for photocontrolled anticancer experiments. Interestingly, after visible-light irradiation, the *Z*-rich 5-PS exhibited significant cytotoxicity in the HepG2 cell line (IC_50_ = 46.3 ± 3.2 μM), in contrast to *E*-5-PS, which showed markedly lower activity (IC_50_ > 140.2 μM).

## Results and discussion

### Molecular design and photoswitch properties of indoleazopyrazole/indazoles

**Fig. 1:**
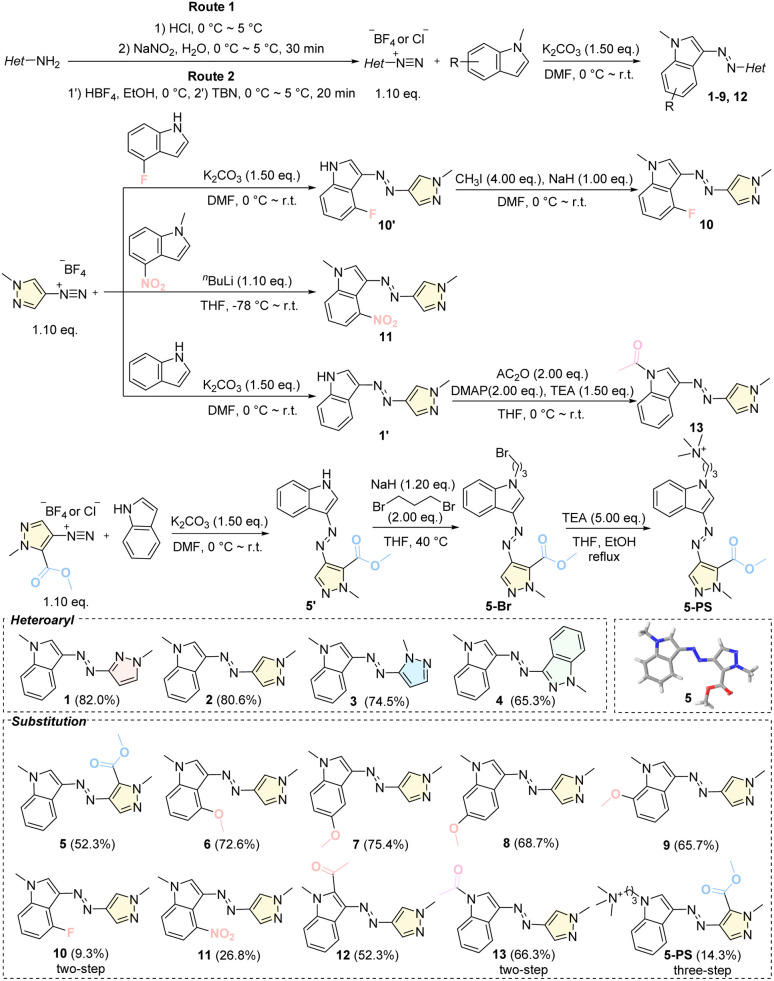
Synthesis and chemical structures of indoleazopyrazole/indazoles 1–13 and X-ray single-crystal structures of 5; see SI for details.

A series of indoleazopyrazole/indazoles (1–13) were synthesized using the classical diazonium coupling reaction (Fig. 1). Initially, various heteroaryl ring units were evaluated (1–4). Following the identification of indoleazo-4-pyrazole as the core structure (2), substituent modification strategies were employed to further modulate the photoswitching properties of compounds 5–13. Most compounds (1–9) were obtained in moderate yields through a one-step reaction starting from preformed diazonium salts. For compounds 10, 13, and 5-PS, precursors containing the N–H group were first synthesized, followed by alkylation or acylation. Finally, the synthesis of indole derivatives with a strong electron-withdrawing group (nitro) required the use of ^*n*^BuLi for effective deprotonation.

**Fig. 2:**
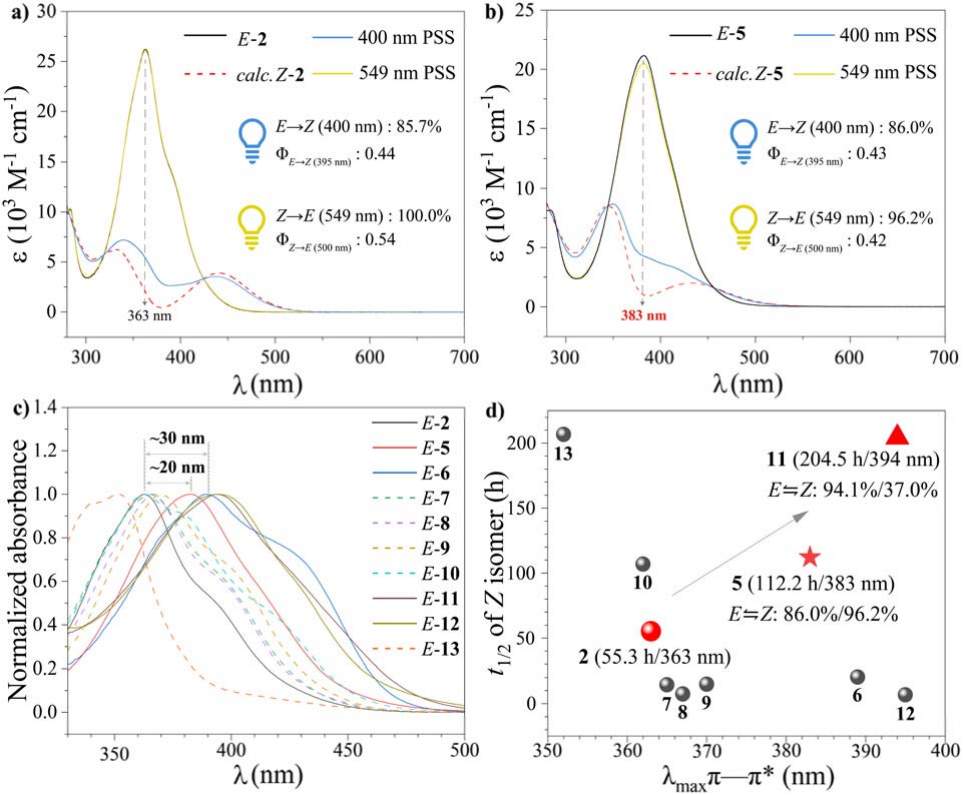
(a) UV-visible absorption spectra of 2 in MeCN. (b) UV-visible absorption spectra of 5 in MeCN. (c) UV-visible absorption spectra (330–500 nm) of *E*-2 and *E*-5–*E*-13 in MeCN. (d) Relationship between the *λ*_max_ (π → π*) and the thermal half-life of the *Z*-isomer for compounds 2, 5–13.

The photoswitching ability of indoleazopyrazole/indazoles was determined by UV-visible absorption spectroscopy in MeCN at 25 °C (Fig. S1–S3). *E*-1 and *E*-2 exhibited a strong π → π* band around 360 nm, while the *n* → π* band was observed around 400 nm as a tail of the π → π* transition. *Z*-1 and *Z*-2 exhibited significantly reduced absorption between 360 nm and 400 nm, enabling *E* → *Z* isomerization of 73.5% and 85.7%, respectively, under 400 nm irradiation (Table 1). Furthermore, both compounds achieved nearly quantitative *Z* → *E* isomerization under 549 nm irradiation. The quantum yield (QY) for *E* → *Z* isomerization (π → π* excitation) of 2 in MeCN was 0.44, substantially higher than that of azobenzene (0.10–0.15) (Fig. 2a). The π → π* absorption bands exhibited a significant redshift (∼20 nm) upon incorporation of more conjugated 5-pyrazole and indazole. However, the *E* → *Z* isomerization ratio under 400-nm irradiation remained largely unchanged, prompting the selection of compound 2 as the core structure for further study. Subsequently, we focused on modifications to the pyrazole ring. Notably, the designed ester substitution at the *ortho* position of the azo group resulted in a substantial redshift (∼20 nm) in *λ*_max_ (π → π*) of *E*-5, resulting in 86.0% *Z*-isomer at 400_PSS_ (Fig. 2b). Despite the significantly reduced *n* → π* transition intensity of *Z*-5 due to symmetry–forbidden transitions, 96.2% *Z* → *E* isomerization was achieved under 549 nm irradiation through weak tail absorption. Furthermore, the QY_*E*→*Z*_ of 5 was 0.43, exceeding that of other fused-ring systems, including azobenzimidazole^47^ (0.22 and 0.17) and arylazoindazole^49^ (0.16 and 0.11).

**Table 1:** The spectroscopic data, *E* ⇄ *Z* photoisomerization and half-life of the indoleazopyrazoles/indazoles

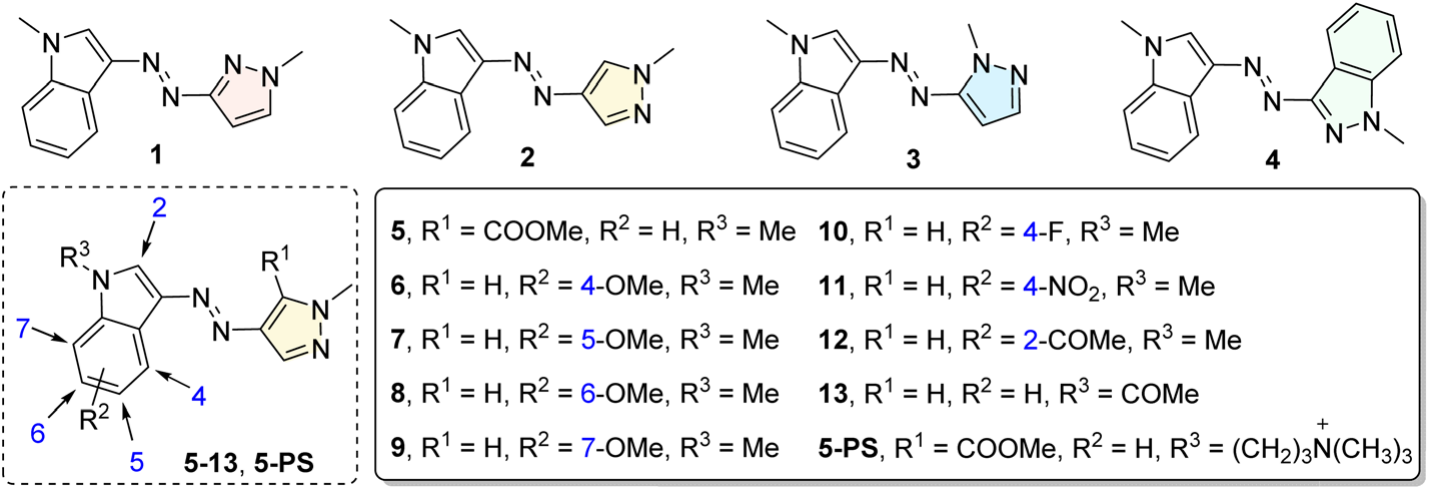					
	*E-*isomer *λ*_max_^a^	*Z-*isomer *λ*_max_^a^	Photoisomerization^b^		*t*_1/2_ (h)^c^
	π → π* (nm)	*n* → π* (nm)	*E* → *Z* (%) 400 nm	*Z* → *E* (%) 549 nm	
1	365	442	73.5	100.0	15.6
2	363	441	85.7	100.0	55.3
3	381	460	81.6	100.0	3.2
4	387	456	>65.9^d^	100.0	0.5
5	**383**	**433**	**86.0**	**96.2**	**112.2**
6	389	447	91.1	98.0	20.5
7	365	440	81.3	100.0	14.5
8	367	443	78.5	96.2	7.4
9	370	444	87.5	99.0	14.9
10	362	427	87.1	96.2	106.9
11	394	429	94.1	37.0^e^	204.5
12	395	448	90.8	79.4	6.8
13	352	426	65.4	88.5^e^	206.7
5-PS^f^	371	440	73.2^g^	100.0^g^	6.7^h^ (26.1^i^)

a The *λ*_max_ was taken from UV-visible absorption spectra measured in MeCN.

b The isomer compositions were determined by ^1^H NMR in CD_3_CN.

c The half-lives were determined in DMSO at 25 °C or various temperatures.

d The isomer compositions of **4** could not be accurately measured due to rapid thermal *Z* → *E* isomerization.

e Irradiation wavelength: 495 nm.

f The *λ*_max_ was taken from UV-visible absorption spectra measured in H_2_O with 1.0‰ Et_3_N.

g The isomer composition was determined by ^1^H NMR in D_2_O.

h The half-life was measured in H_2_O with 1.0‰ Et_3_N at 37 °C.

i The half-life was measured in DMSO at 37 °C.

We subsequently investigated the structure–property relationship by introducing an electron-donating group (–OMe) at the 4-, 5-, 6- and 7-positions of indole. Among the derivatives, only *E*-6 (*λ*_max_ = 389 nm) exhibited a 26-nm redshift compared to *E*-2, along with significant broadening of both the π → π* and *n* → π* absorption bands (Fig. 2c). As a result, the *E* ↔ *Z* isomerization efficiency (>90%) was significantly improved under 400 nm and 549 nm irradiation. Given the substantial spectral modulation observed with 4-position substitution on indole, we further investigated electron-withdrawing group (EWG) substitutions, including –F and –NO_2_ (molecules 10 and 11). Compared to *E*-2, *λ*_max_ (π → π*) of *ortho*-fluorinated *E*-10 remained largely unchanged, enabling efficient bidirectional isomerization under visible-light irradiation (*E* → *Z*_400nm_: 87.1%; *Z* → *E*_549nm_: 96.2%). With increased electron-withdrawing strength and steric hindrance, *E*-11 exhibited a redshifted *λ*_max_ (π → π*) to 394 nm, accompanied by a reduction in *n* → π* absorption intensity of *Z*-11. Consequently, although the *E* → *Z* isomerization reached 94.1%, the reverse *Z* → *E* isomerization under 495 nm irradiation was limited to 37.0%. The effect of other *ortho*-substitution of the N=N bond was investigated by designing derivative 12, which features an acetyl group at the 2-position of indole. Similar to compound 11, the electron-withdrawing acetyl substitution induced a redshift in *λ*_max_ (π → π*) of the *E*-isomer to 395 nm, leading to spectral overlap with the *n* → π* band of *Z*-12. Based on previous work,^56^ we recognized that substitution of an EWG at the *N*-position of indole causes a blueshift in *λ*_max_ (π → π*) of the *E*-isomer owing to an increased HOMO–LUMO gap. Consequently, the *E* → *Z* isomerization efficiency of *E*-13 under 400 nm irradiation decreased to 65.4%.

The thermal *Z* → *E* isomerization of indoleazopyrazole/indazoles was determined in DMSO and followed first-order kinetics (Fig. S21–S30 and Tables S6–S15). *Z*-1 exhibited a *t*_1/2_ of approximately 15.6 h, while *Z*-2 displayed an extended *t*_1/2_ of 55.3 h (2.3 d), attributed to the exceptional properties of the 4-pyrazole unit. With increasing conjugation (compounds 3 and 4), *t*_1/2_ decreased significantly (3.2 h and 0.5 h, respectively). Notably, the introduction of the ester substitution at the *ortho* position (relative to the azo group) significantly extended the thermal half-life of *Z*-5 to 112.2 h (4.7 d), despite a further redshift of the π → π* absorption of *E*-5 (Fig. 2d). Methoxy substitution (EDG) at different positions reduced the thermal stability of *Z*-isomers. However, introducing an EWG (–F and –NO_2_) at the 4-position increased the half-life of the *Z*-isomer by approximately two-to fourfold compared to *Z*-2. Ketocarbonyl substitution led to substantial variations in half-lives. The half-life decreased to 6.8 h with substitution at the 2-position of indole (*Z*-12), whereas substitution at the *N*-position of indole significantly enhanced thermal stability (*Z*-13 : 206.7 h/8.6 d). Combined with spectral analysis, only compounds 5 and 11 exhibited a notable increase in half-life while simultaneously achieving a redshift of *λ*_max_ (π → π*) (Fig. 2d).

### Theoretical calculations

**Fig. 3:**
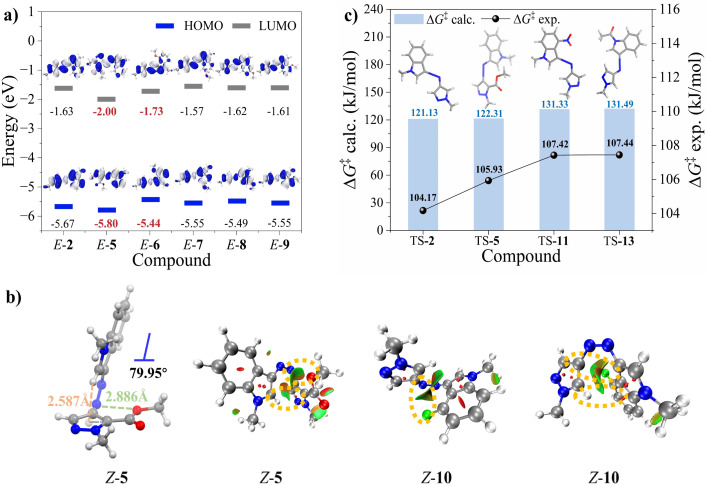
(a) FMOs for the predominant conformers of *E*-2 and *E*-5–9 (isosurface value = 0.02). (b) Geometries of the dominant *Z*-isomer of 5, and noncovalent index (NCI) surfaces of *Z*-5 and *Z*-10. The C–H⋯π interaction in *Z*-5 is shown by a light pink dashed line and the O⋯N distance is indicated by a green dashed line. (c) The Δ*G*_calc._^‡^ and Δ*G*_exp._^‡^ for *Z*-2, 5, 11 and 13 in DMSO. Computational TS structures of 2, 5, 11 and 13 at the PBE0-D3(BJ)/6-311G** level of theory using the SMD solvation model (DMSO).

To further investigate the dual enhancement of spectral redshift and thermal half-life, we conducted DFT calculations using MeCN or DMSO as solvent models. For molecule 5, frontier molecular orbital (FMO) analysis of *E*-5 revealed stabilization of both the HOMO and LUMO, with a more pronounced effect on the LUMO (Fig. 3a). The LUMO energy decreased from −1.63 eV (*E*-2) to −2.00 eV (*E*-5), reducing the HOMO–LUMO gap and causing a corresponding spectral redshift. π-Electron population analysis and the corresponding density map for *E*-5 showed a reduction in π-electron density on the N=N bond, with a shift toward the indole ring. This shift was attributed to the electron-withdrawing conjugation effect (–C) of the ester group and the electron–rich properties of the indole nitrogen (Fig. S39 and Table S25). Consequently, the electronic redistribution lowered the excitation energy, leading to a redshift in the absorption wavelength. Additionally, the ester substitution at the *ortho* position (relative to the azo group) induced minor structural distortion in the *E*-isomer, increasing the dihedral angle (C–C–N=N: *φ*1/N=N–C–C: *φ*2) from 0.00°/0.00° (*E*-2) to 4.10°/0.13° (*E*-5) (Fig. S44). This distortion enhanced the intensity of the *n* → π* absorption and increased the oscillator intensity (*f*) from 0.000 to 0.002 (Table S24). Unlike the ester substitution at the *ortho* position (relative to the azo group), which simultaneously stabilized both HOMO and LUMO orbitals, the 4-OMe substitution selectively enhanced the HOMO energy (from −5.67 eV to −5.44 eV) and decreased the LUMO energy (from −1.63 eV to −1.73 eV) (Fig. 3a). This dual modulation significantly reduced the π → π* excitation energy (from 3.47 eV to 3.10 eV) (Table S24), resulting in a redshifted absorption band. To elucidate the relationship between spectral redshift and electronic transition characteristics of compound 11, the π → π*’, *n* → π* and π → π* transitions were analyzed by FMO and natural transition orbital calculations (Fig. S43). A charge-transfer (CT) transition was observed in *E*-11, creating a lower energy pathway that induced a redshifted absorption band. Due to the presence of the nitro group, the HOMO exhibited higher electron density in the indole moiety than in pyrazole, while the LUMO displayed an even higher concentration of electron density in indole (96.76%) as a result of intramolecular CT.

To investigate the effect of substituent modifications on half-life enhancement, the NCI of *Z*-5/10, along with the minimum energy structures and the transition states (TSs) of *Z*-2/5/11/13 were analyzed.^57,58^ As shown in Fig. 3b, *Z*-5 exhibited a nearly T-shaped conformation, with the H atom at the 2-position of indole oriented towards the pyrazole, forming a C–H⋯π interaction. Furthermore, NCI analysis revealed that the O(sp^3^) of the ester group formed an attractive interaction with the nitrogen atom of the N=N bond, further stabilizing *Z*-5. In *Z*-10, the fluorine atom interacted with the carbon at the 4-position of the pyrazole ring. Despite increased distortion of the T-configuration, the H atom at the 5-position of pyrazole remained attracted to the indole plane *via* a C–H⋯π interaction, thereby contributing to enhanced conformational stability of *Z*-10. To understand the thermal *Z* → *E* isomerization of 2, 5, 11 and 13, we investigated the isomerization process and demonstrated that the inversion process involves a lower energy barrier than the rotation process, indicating that it is the energetically favored pathway (Fig. 3c). Furthermore, the introduction of the ester group on the pyrazole ring induced a conformational deviation in TS-5 compared to TS-2/11/13, with the N=N bond preferentially adopting an orientation of approximately 180° relative to the pyrazole ring, possibly due to the electron-withdrawing interaction of the ester group. Theoretical free energy barriers (Δ*G*_calc._^‡^) were obtained by calculating the Gibbs free energies of *Z*-isomers (Δ*G*_*Z*_^‡^) and TSs (Δ*G*_TS_^‡^), while experimental free energy barriers (Δ*G*_exp._^‡^) were determined by the Arrhenius and Eyring plots of the *Z* → *E* thermal isomerization kinetics in DMSO solution at different temperatures. Compared to 2, both Δ*G*_calc._^‡^ and Δ*G*_exp._^‡^ increased for 5 (Δ*G*_calc._^‡^: 121.1 kJ mol^−1^*vs.* 122.3 kJ mol^−1^ and Δ*G*_exp._^‡^: 104.2 kJ mol^−1^*vs.* 105.9 kJ mol^−1^), correlating with the observed twofold increase in the half-life of *Z*-5. Moreover, the Δ*G*^‡^ values were significantly higher in *Z*-11 and *Z*-13, resulting in markedly prolonged half-lives for the thermal *Z* → *E* isomerization.

Based on the experimental findings and DFT calculations, we proposed a scenario in which spectral redshift—achieved either by stabilizing the HOMO to reduce the HOMO–LUMO gap or by introducing new excitation bands (*e.g.*, CT transition) through *ortho* modifications—might also enhance the thermal half-life of the *Z*-isomers, thereby enabling the simultaneous dual enhancement of spectral redshift and thermal half-life.

### Photopharmacology

**Fig. 4:**
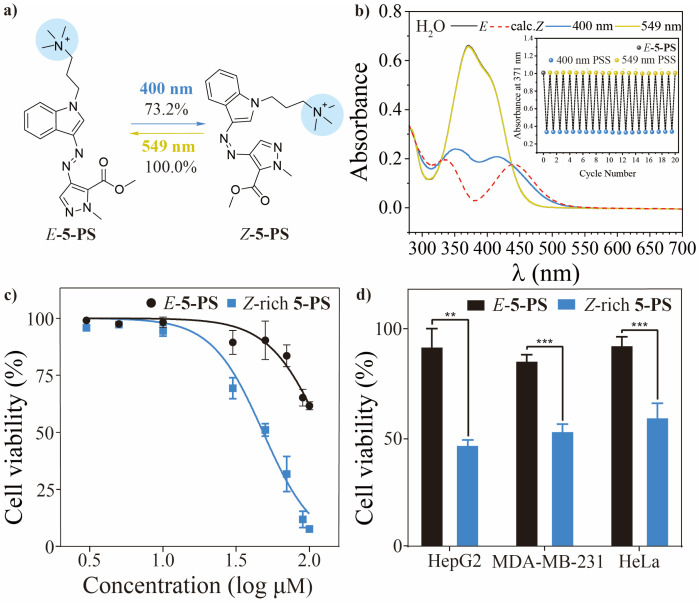
(a) Molecular structure and photochemical properties of 5-PS. (b) UV-visible absorption spectra of 5-PS in H_2_O with 1‰ Et_3_N (inset: cyclic stability of 5-PS in H_2_O with 1‰ Et_3_N). (c) Antiproliferative activity on HepG2, using both *E*-5-PS and *Z*-rich 5-PS. (d) Effect of 5-PS (50 μM) against HepG2, MDA-MB-231 and HeLa cells under either dark or 400 nm irradiation conditions, measured by MTT assays.

Acknowledging the biological potential of indole and pyrazole moieties, we proceeded with photopharmacological studies. Molecule 5 emerged as the optimal candidate for further development based on its excellent bidirectional isomerization efficiency, prolonged thermal half-life, and significant conformational switching capacity. The compound demonstrated exceptional cycling stability, maintaining its photoresponsive properties through ten complete isomerization cycles (400 nm/549 nm irradiation) in DMSO with no signs of photofatigue (Fig. S31). Addressing the critical challenge of aqueous solubility^59,60^—a key determinant in drug development—we employed rational molecular design guided by structure–property relationship analysis and DFT calculations. Quaternization of the indole nitrogen yielded the water-soluble derivative 5-PS while preserving the core photoswitching characteristics. In organic solvents, 5-PS maintained photophysical behavior comparable to that of the parent compound 5. However, aqueous environments induced a redshifted absorption profile and accelerated thermal relaxation, primarily due to intermolecular hydrogen bonding with H_2_O. To mitigate this effect, 1‰ Et_3_N was added to disrupt hydrogen bonding, facilitating efficient visible-light-induced isomerization in water (*E* → *Z* under 400 nm irradiation: 73.2% and *Z* → *E* under 549 nm irradiation: 100.0%). For biological relevance, we characterized the system under physiological conditions (37 °C), observing a thermal half-life of 6.7 h – adequate for cytotoxicity assessments. Importantly, 5-PS retained excellent cyclic photostability in aqueous solution, exhibiting no detectable degradation after 20 irradiation cycles (Fig. 4b, inset), thus validating its suitability for photopharmacological applications.

The photocontrolled anticancer potential of 5-PS*in vitro* was systematically evaluated against three human cancer cell lines (HepG2, MDA-MB-231 and HeLa) using MTT assays. Control experiments confirmed that neither the presence of 1‰ Et_3_N nor 400-nm LED irradiation alone affected cell proliferation. The *E*-isomer of 5-PS demonstrated mild cytotoxicity. In HepG2 cells, cytotoxic effects became apparent at an *E*-5-PS concentration of 60 μM. In MDA-MB-231 and HeLa cells, a gradual decrease in viability was observed only at concentrations above 100 μM, exhibiting varying IC_50(*E*-__5-PS__)_ values: HepG2, >140.2 μM; MDA-MB-231, >500.4 μM; HeLa, >150.1 μM (Fig. 4c and S32). Conversely, the 400 nm PSS group (*Z*-rich isomer of 5-PS) exhibited significant antiproliferative activity. In HepG2 cells, cell survival declined significantly at 10 μM, with only ∼10% viability remaining at 100 μM. Similar trends were observed in MDA-MB-231 cells and HeLa cells, where the *Z*-rich isomer exhibited higher cytotoxicity even at low concentrations, with only ∼40% viability at 100 μM. These findings contrasted sharply with the dark group, in which *E*-5-PS exhibited negligible toxicity at concentrations below 100 μM (Fig. S32 and Tables S16–S18). The IC_50_ values of *Z*-rich isomers were generally 3- to 10-fold lower than those of *E*-5-PS (HepG2, ≈46.3 μM; MDA-MB-231, ≈55.6 μM; HeLa, ≈83.2 μM). At 50 μM, *E*-5-PS showed no significant toxicity in any of the three cell lines, whereas only ∼50% of the cells remained viable in the 400 nm PSS group (Fig. 4d). This significant light-controlled therapeutic window highlights the potential for precise spatiotemporal control of anticancer activity.

To preliminarily investigate the underlying causes of the high toxicity of the *Z*-isomer, we examined the aggregation behavior and reactive oxygen species (ROS) production before and after light irradiation. Transmission electron microscopy characterization revealed that 5-PS was well dispersed in solution before irradiation, whereas microspheres of various sizes formed after light exposure (Fig. S45–46). We hypothesized that the enhanced toxicity may result from the aggregates being more readily adsorbed onto cell membranes while exhibiting reduced susceptibility to metabolic degradation and elimination. Moreover, no significant ROS production was detected after irradiation (Fig. S47), thereby preliminarily ruling out ROS-mediated enhancement of cytotoxicity.

## Conclusions

In summary, a series of indoleazopyrazoles were identified as efficient bidirectional visible-light-responsive photoswitches. Heteroaryl engineering and substituent modifications were employed to investigate the relationship between molecular structure, spectral property and half-life. The ester substitution at the *ortho* position of the azo bond effectively induced a spectral redshift while maintaining half-life through modulation of molecular geometry and electron density distribution. This approach offers insights toward achieving the dual enhancement of *λ*_max_ (π → π*) redshift and prolonged *Z*-isomer thermal half-life. For instance, 5 exhibited *λ*_max_ (π → π*) of 383 nm, representing a 20 nm redshift relative to 2, while the thermal half-life of *Z*-5 extended to 4.7 days, approximately twice that of *Z*-2. Our calculation studies corroborated the experimental observations of the *λ*_max_ (π → π*) redshift and the prolonged half-life, and further proposed a potential strategy to achieve simultaneous enhancement of both properties. Furthermore, in HepG2, MDA-MB-231 and HeLa cells, the *Z*-rich water-soluble derivative 5-PS exhibited significantly enhanced cytotoxicity compared to *E*-5-PS, particularly in HepG2 cells, with an IC_50(*Z*-rich-__5-PS__)_ approximately three fold lower than IC_50(*E*-__5-PS__)_. These findings highlight the potential of indoleazopyrazoles as visible-light-responsive switches for photoselective anticancer activity modulation. This strategy provides a versatile platform for photocontrolled cancer therapy and establishes a structural foundation for the rational design of advanced light-responsive therapeutics.

## Author contributions

T. L., D. W. and B. L. conceived the study and supervised the project. X. Y. was primarily responsible for the experiments. C. Z. provided the photopharmacological experiments. D. D. helped synthesize the compounds. All authors contributed to the writing of the manuscript.

## Conflicts of interest

There are no conflicts to declare.

## Supplementary Material

SC-016-D5SC03275J-s001

SC-016-D5SC03275J-s002

## Data Availability

The data supporting this article have been included as part of the SI. CCDC 2424490, 2424491, 2424493 and 2424494 contain the supplementary crystallographic data for this paper.^61–64^ Supplementary information is available and includes experimental procedures, characterization data of the compounds (NMR, MS, UV-vis, single-crystal) and computational data. See DOI: https://doi.org/10.1039/d5sc03275j.
