# Supplementary material for: Visible-light-responsive indoleazopyrazole photoswitches: dual enhancement of redshift and half-life by ester modification at the ortho position
Source: Chem Sci. 2025 Jul 21;16(34):15620–7. doi: 10.1039/d5sc03275j (PMC12315719; doi:10.1039/d5sc03275j)
Supplement: SC-016-D5SC03275J-s001 [file SC-016-D5SC03275J-s001.pdf]

Supplementary Information (SI) for Chemical Science.
This journal is © The Royal Society of Chemistry 2025

Supporting Information

Visible-Light Responsive Indoleazopyrazole Photoswitches: Dual-Enhancement of Redshift and Half-life by Ester Modification at the *Ortho* Position

Xuanchi Yu,[1, 2†] Chenyu Zhang,[3†] Dongfang Dong,[1, 2] Bing Liu,[3] Dali Wang[1, 4]* & Tao Li[1, 2]*

[1] School of Chemistry and Chemical Engineering, Shanghai Jiao Tong University, Shanghai 200240, [2] State Key Laboratory of Synergistic Chem-Bio Synthesis, Shanghai Key Laboratory of Electrical Insulation and Thermal Aging, Shanghai Jiao Tong University, Shanghai 200240, [3] Department of Oral and Maxillofacial Surgery, The First Affiliated Hospital of Harbin Medical University, Harbin 150001, [4] Zhang Jiang Institute for Advanced Study, Shanghai Jiao Tong University, Shanghai 200240.

Table of Contents

1. General methods 2
 1.1. Experimental methods 2
 1.2. Computational methods 3
2. Synthesis 4
 2.1. General Procedure for the synthesis of diazo compounds 4
 2.2. General Procedure for azo compounds 4
 2.3. NMR spectra 11
3. Photoswitching performance 29
 3.1. UV/Vis spectra 30
 3.2. Calculation of PSS composition by UV/Vis spectroscopy 33
 3.3. Qualification of PSS composition by NMR in CD_3CN or D_2O. 34
 3.4. Quantum yields of photoisomerization in acetonitrile 48
4. Thermal isomerization kinetics 51
5. Cycling stability and cytotoxicity 62
6. Single crystal X-ray structures for compounds 64
7. Computational details 68
8. TEM images and ROS detection 113
9. References 114

1. General methods

1.1. Experimental methods

All reagents and solvents were commercially available, and used directly without further purification. Reactions were monitored by thin layer chromatography (TLC) carried out on silica gel plates (HSGF254, Huanghai) using UV light (λ = 254 nm) for visualization. Silica gel (300–400 mesh, Huanghai) was used for column chromatography. **^{1}H NMR**, **^{13}C NMR** and **^{19}F NMR** spectra were recorded on AVANCE III HD 400 spectrometers (Bruker) with chemical shift of the residual solvent as an internal reference at 400 MHz, 101 MHz and 376 MHz, respectively. The following abbreviations (or combinations thereof) were used to explain multiplicities: s = singlet, d = doublet, t = triplet, q = quartet, m = multiplet, dd = double doublet, ddd = double double doublet, dt = double triplet, td = triple doublet. Coupling constants, *J*, were reported in Hertz unit (Hz). **HRMS** data were obtained on Bruker Impact II quadrupole time of flight mass spectrometry instrument and TRACE 1610/orbitrap exploris GC 240. **UV/Vis** absorption spectra were recorded on UV-2700 spectrophotometer (Shimadzu) with slit width of 2.0 nm. **Variable-temperature UV-Vis** absorbance for thermal $Z \rightarrow E$ isomerization kinetics was monitored by same spectrophotometer equipped with a programmable temperature controller (DCY-1006, Shanghai Sunny Hengping Scientific Instrument Ltd). **Melting point** (M.P.) were determined on SGW X-4B digital melting point apparatus (Shanghai INESA Physical Optics Instrument Co., Ltd). **Transmission electron microscopy** (TEM) were determined on Tecnai G2 spirit Biotwin (Thermofisher). **Cell culture and compound preparation**. All cells were purchased from American Type Culture Collection (ATCC) cell bank. HepG2 cells (hepatocellular carcinoma type G2), MDA-MB-231 cells (breast cancer cells) and HeLa cells (cervical cancer) were maintained in Dulbecco's modified eagle medium (DMEM, Gibco, USA), supplemented with 10% fetal bovine serum (FBS, Gibco, USA), 100 µg/ mL streptomycin and 100 U/ mL penicillin in a humidified incubator with 5% CO_2 at 37 °C. **5-PS** was dissolved in ddH_2O with 1‰ Et_3N. **Cell Viability.** The cytotoxicity of the tested compounds against HepG2 cells (hepatocellular carcinoma type G2), MDA-MB-231 cells (breast cancer cells) and HeLa cells (cervical cancer) was measured by Methyl Thiazol Tetrazolium (MTT) assay. Cells were seeded in 96-well plates and incubated for 24 h. Subsequently, cells were treated with the tested compounds at different concentrations for 48 h in dark. And for the light cytotoxicity they were irradiated with a 400 nm LED light (10 W) for 10 mins every 6 h. 20 µL MTT (5 mg/mL in 1×PBS) was added to each well and incubated for an another 4 h. Then the medium was carefully removed and 150 µL DMSO was added to each well. The absorbance at 595 nm was recorded using a microplate reader (Infinite M200 Pro, Tecan, Switzerland). The percentage of cell viability was calculated using the equation: (mean optical density of treated cells/mean optical density of control cells) × 100%. Data were reported as the mean ± standard deviation (n = 3). **Cellular ROS detection.** HepG2 cells were seeded in 6-well plates and incubated overnight. Upon treatment with **5-PS** (50 µM) for 15 min, these cells were irradiated with 400 nm LED irradiation (10 min) and incubated for 30 min. The cells were harvested and incubated with 10 µM dichloro-dihydro-fluorescein diacetate (DCFH-DA) in serum-free medium for 15 min at 37 °C in the dark. Then the cells were collected by centrifugation and washed twice with PBS in order to remove excess DCFH-DA. The samples were analyzed by flow cytometry, and the data were analyzed using FlowJo software. λ_{ex} = 488 nm, λ_{em} = 525 ± 20 nm. The Z hydrodynamic diameters of **5-PS** samples

dissolved in deionized water were measured by EliteSizer (Brookhaven Instruments; USA) at 25 °C.

Statistical analysis. All the experiments were replicated three times. The statistical significance in this study is determined by the unpaired, two-tailed Student's t-test and One-way ANOVA at $*p < 0.05$, $**p < 0.01$, $***p < 0.001$ and $****p < 0.0001$. Data were presented as means ± standard deviations (SD). IC_{50} curves were created using GraphPad Prism 8.0.1 software.

Abbreviations Used:

Ac = acetyl, aq. = aqueous, DCM = dichloromethane, DFT = density functional theory, DMAP = 4-dimethylaminopyridine, DMF = *N*,*N*-dimethylformamide, EA = ether acetate, equiv. = equivalents, h = hours, m/z = mass to charge ratio, NMR = nuclear magnetic resonance, PE = petroleum ether, Ph = phenyl, r.t. = room temperature, TBN = *tert*-butyl nitrite, THF = tetrahydrofuran, TLC = thin layer chromatography.

1.2. Computational methods

DFT calculations were performed on Gaussian 16 program.[1] The ground state geometries were optimized at PBE0-D3(BJ)/6-311G** level of theory[2] using SMD solvation model.[3] The gas-phase free energies (E_{sol}) and solution-phase free energies (E_{gas}) (using SMD solvation model) of each conformer was computed at M05-2X/6-31G*[2a, 5] level of theory. The high-precision single-point energy (E_{sp}) was computed at B2PLYP-D3/def2-TZVP[4] level of theory. The Gibbs free energy in solution ($G_{solution}$) = thermal correction to Gibbs free energy + high-precision single-point energy (E_{sp}) + solvation free energy (E_{sol} - E_{gas}) + 1.89 kcal/mol. Relative $G_{solution}$ = $G_{solution}$ for each geometry configuration - $G_{solution}$ for lowest energy geometry configuration. The Boltzmann distribution of all possible conformers were deduced by the relative $G_{solution}$ ($p_i = \frac{e^{-\Delta E_i/\mathrm{RT}}}{\sum_j e^{-\Delta E_j/\mathrm{RT}}} = \frac{Q_i}{Q}$; p_i = proportion of geometric configuration, E_i = relative $G_{solution}$ for each geometric configuration, R = ideal gas constant, T = temperature (K), Q_i = partition function) (Table S20). The vertical electronic excitation energies and oscillator strengths were calculated by time-dependent density functional theory (TD-DFT), and the 20 lowest-lying singlet excited states were considered at PBE0/6-311G** level of theory[2] with SMD solvation model in acetonitrile (Table S22).[3] The relaxed scans for C–N=N–C dihedral angle φ (using the broken symmetry (BS) approach[6]) and two C–N=N bond angles θ were carried out at the same level to explore the mechanism of thermal *Z*→*E* isomerization and obtain the initial geometries for transition states (TSs). The geometries of TSs were also optimized at the same level followed by vibrational frequency analysis. The inversion TSs were found at singlet states, and the rotational TSs were found at open-shell singlet states using BS approach.[6] The gas-phase free energies and solvation free energies (using SMD solvation model) of each TSs were computed at the same level.

The noncovalent interaction (NCI) analysis[7] were performed by Multiwfn 3.8 program[8] and the plots were rendered by VMD program.[9]

2. Synthesis

2.1. General Procedure for the synthesis of diazo compounds

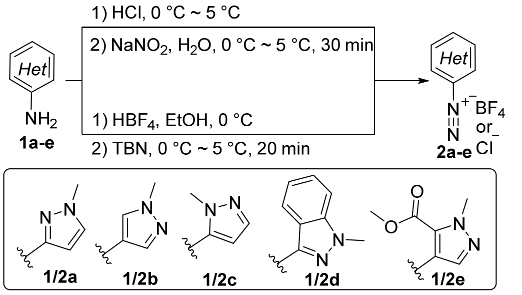

The heteroaryl amines (**1a-e**) (9.00 mmol) were dissolved in H_2O (15.0 mL), then 9.00 mL of concentrated HCl (12.2 mol/L, 12.0 eq.) was added. After cooling to 0 °C, a prechilled solution of $NaNO_2$ (2.24 g, 3.60 eq.) in 30.0 mL of water was added slowly, then stirred at 0 ~ 5 °C for 30 min to obtain an aqueous solution of diazonium chloride, used for further reactions. Similarly, the indicated heteroaryl amines (**1a-e**) (9.00 mmol) were dissolved in EtOH (15.0 mL) at 0 °C, then 3.00 mL of tetrafluoroboric acid was added. After 2 mins, TBN (1.50 eq.) was added slowly and stirred at 0 ~ 5 °C for 20 mins. Finally, the solid diazonium tetrafluoroborate was precipitated by adding 20.0 mL of ether, filtered and washed with ether (5.00 mL×3).

2.2. General Procedure for azo compounds

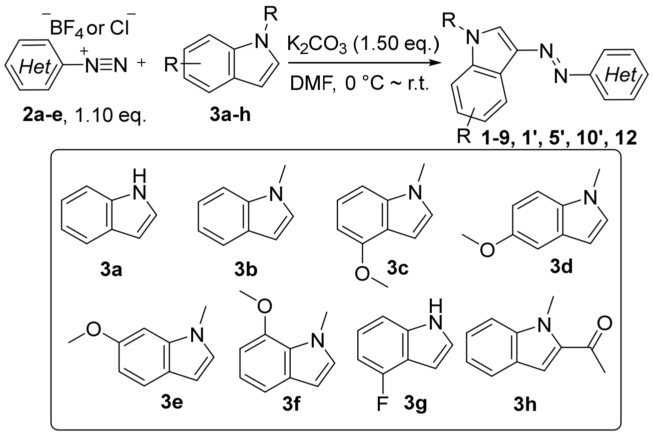

The indole derivatives **3a-h** and K_2CO_3 (1.50 eq.) were added to a round bottom flask and dissolved in DMF at 0 °C, stirred for 10 minutes and added aryldiazonium salt **2a-f** (1.10 eq.). The mixture was reacted at 0 °C for 15 minutes, then transferred to r.t. and continued reacting for 16 hours. After the reaction, the resulting mixture was quenched with $NaHCO_3$ or Na_2CO_3 (1.00 M) and washed with brine, most products precipitated in water and filtrated to obtain crude products, then recrystallized with DCM and *n*-hexane. In the case of the few compounds that don't precipitate, the aq. layer was extracted with EA, dried over Na_2SO_4, filtered, concentrated by vacuum evaporation and purified by flash column chromatography. Further products were recrystallized with DCM and *n*-hexane, if required.

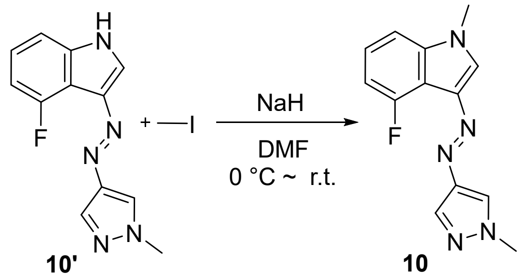

The purified (*E*)-4-fluoro-3-((1-methyl-1*H*-pyrazol-4-yl)diazenyl)-1*H*-indole **10'** was reacted with CH_3I (4.00 equiv.) and NaH (1.10 equiv.) in DMF at r.t.. After reacting for 8 h, the resulting mixture was washed with brine and extracted with EA, then the combined organic layers were washed with brine, dried over Na_2SO_4, filtered, concentrated by vacuum evaporation. Finally purification by column chromatography (PE:EA = 8:1) to afford the product **10**.

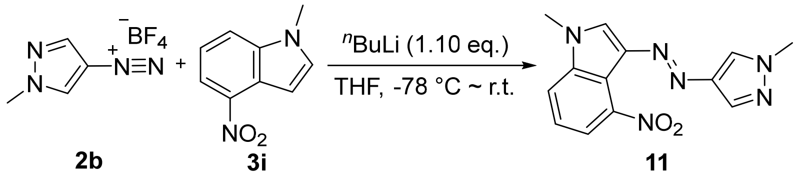

The indole derivatives **3i** were added to a round bottom flask and dissolved in dry THF at -78 °C, added ^{n}BuLi (1.10 eq.) and stirred for 20 minutes. Then rapidly added 1-methyl-1*H*-pyrazole-4-diazonium tetrafluoroborate **2b** (1.10 eq.). The mixture was reacted at -78 °C for 2 h, then transferred to r.t. and continued reacting for 2 h. After the reaction, the resulting mixture was quenched with NH_4Cl and washed with brine, the aq. layer was extracted with EA, dried over Na_2SO_4, filtered, concentrated by vacuum evaporation and purified by flash column chromatography to obtain products **11**. Further products were recrystallized with DCM and *n*-hexane, if required.

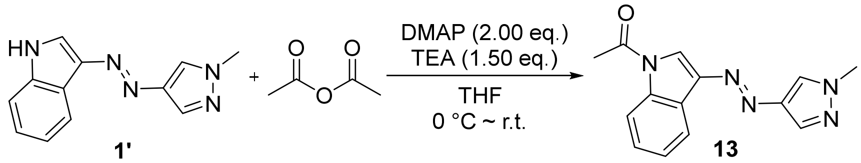

The purified (*E*)-3-((1-methyl-1*H*-pyrazol-4-yl)diazenyl)-1*H*-indole (**1'**) and DMAP (2.00 equiv.) were added in THF (20.0 mL) at 0 °C, and acetic anhydride (2.00 eq.), TEA (1.50 eq.) were slowly added. Then the reaction was stirred at r.t. for 8 h. After the reaction, the mixture was treated with brine and stirred for 5 minutes, the aq. layer was extracted with EA, and the combined organic layers were dried over Na_2SO_4, filtered, and concentrated by vacuum evaporation The resulting crude material was purified by flash column chromatography to afford (*E*)-1-(3-((1-methyl-1*H*-pyrazol-4-yl)diazenyl)-1*H*-indol-1-yl)ethan-1-one **13** as an orange solid.

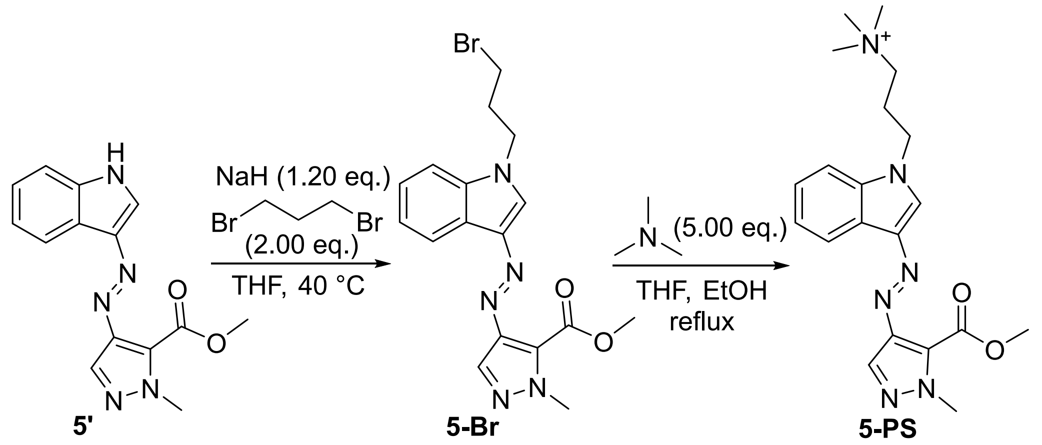

The purified methyl (*E*)-4-((1*H*-indol-3-yl)diazenyl)-1-methyl-1*H*-pyrazole-5-carboxylate **5'** was added to a round bottom flask and dissolved in dry THF at 0 °C, added NaH (1.20 eq.) and stirred for 30 minutes. Then, it reacted with 1,3-dibromopropane under the basic conditions of NaH for 8 h. The solution was dried with Na_2SO_4, distilled under reduced pressure and purified by column chromatography (PE/EA = 2:1) to obtain yellow methyl (*E*)-4-((1-(3-bromopropyl)-1*H*-indol-3-yl)diazenyl)-1-methyl-1*H*-pyrazole-5-carboxylate **5-Br**. Then, **5-Br** was dissolved in THF, and trimethylamine in EtOH was added. The reaction was refluxed overnight, and a yellow solid was precipitated by the addition of EA, which was washed with EA/hexanes to give the product (*E*)-3-(3-((5-(methoxycarbonyl)-1-methyl-1*H*-pyrazol-4-yl)diazenyl)-1*H*-indol-1-yl)-*N*,*N*,*N*-trimethylpropan-1-aminium **5-PS** as a yellow solid.

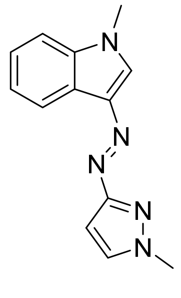

(*E*)-1-methyl-3-((1-methyl-1*H*-pyrazol-3-yl)diazenyl)-1*H*-indole (**1**). The 1-methyl-1*H*-indole **3b** (1.31 g, 10.0 mmol) was reacted with 1-methyl-1*H*-pyrazole-3-diazonium tetrafluoroborate **2a** (2.16 g, 1.10 eq.) according to the procedure detailed above to afford **1** as a yellow solid (1.96 g, 82.0%). **^{1}H NMR** (400 MHz, $CDCl_3$) δ 8.60 (d, *J* = 8.0 Hz, 1H), 7.84 (s, 1H), 7.36 (d, *J* = 2.8 Hz, 1H), 7.34 – 7.27 (m, 3H), 6.58 (d, *J* = 2.4 Hz, 1H), 4.00 (s, 3H), 3.87 (s, 3H); **^{13}C NMR** (101 MHz, $CDCl_3$) δ 164.7, 137.4, 135.6, 134.7, 131.5, 124.0, 123.7, 122.9, 119.5, 109.3, 95.6, 39.4, 33.5. **HRMS** calculated for $[C_{13}H_{13}N_5+H]^+$: 240.1244, found: 240.1242. **m.p.** 172.0 – 172.1 °C.

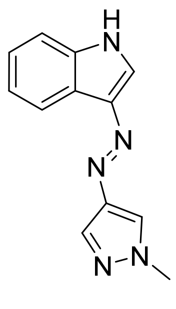

(*E*)-3-((1-methyl-1*H*-pyrazol-4-yl)diazenyl)-1*H*-indole (**2'**). The indole **3a** (1.17 g, 10.0 mmol) was reacted with 1-methyl-1*H*-pyrazole-4-diazonium tetrafluoroborate **2b** (2.16 g, 1.10 eq.) according to the procedure detailed above to afford **1'** as a yellow solid (1.73 g, 76.7%). **^{1}H NMR** (400 MHz, DMSO) δ 11.86 (s, 1H), 8.27 (dd, *J* = 8.0, 1.2 Hz, 1H), 8.24 (s, 1H), 8.15 (s, 1H), 7.85 (s, 1H), 7.46 (d, *J* = 8.0 Hz, 1H), 7.24 (td, *J* = 7.2, 1.6 Hz, 1H), 7.18 (td, *J* = 7.6, 0.8 Hz, 1H), 3.90 (s, 3H); **^{13}C NMR** (101 MHz, DMSO) one carbon signal was overlapped (~39.1) δ 142.3, 136.9, 135.6, 131.7, 131.2, 126.3, 123.9, 122.5, 122.4, 118.8, 112.6.

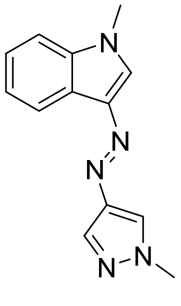

(*E*)-1-methyl-3-((1-methyl-1*H*-pyrazol-4-yl)diazenyl)-1*H*-indole (**2**). The 1-methyl-1*H*-indole **3b** (1.31 g, 10.0 mmol) was reacted with 1-methyl-1*H*-pyrazole-4-diazonium tetrafluoroborate **2b** (2.16 g, 1.10 eq.) according to the procedure detailed to afford **2** as yellow solid (1.93 g, 80.6%). **^{1}H NMR** (400 MHz, $CDCl_3$) δ 8.44 (d, *J* = 4.0 Hz, 1H), 7.97 (s, 1H), 7.86 (s, 1H), 7.71 (s, 1H), 7.34 – 7.31 (m, 2H), 7.30 – 7.27 (m, 1H), 3.95 (s, 3H), 3.85 (s, 3H); **^{13}C NMR** (101 MHz, $CDCl_3$) δ 142.6, 137.2, 135.2, 132.7, 132.5, 124.8, 123.7, 122.9, 122.4, 119.6, 109.3, 39.4, 33.3. **HRMS**

calculated for $[C_{13}H_{13}N_5+H]^+$: 240.1244, found: 240.1243. **m.p.** 155.7 – 156.1 °C.

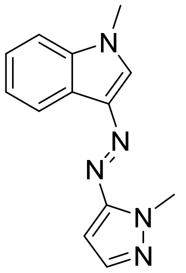

(*E*)-1-methyl-3-((1-methyl-1*H*-pyrazol-5-yl)diazenyl)-1*H*-indole (3). The 1-methyl-1*H*-indole **3b** (1.31 g, 10.0 mmol) reacted with 1-methyl-1*H*-pyrazole-5-diazonium tetrafluoroborate **2c** (2.16 g, 1.10 eq.) according to the procedure detailed above to afford **3** as yellow solid (1.78 g, 74.5%). **^{1}H NMR** (400 MHz, $CDCl_3$) δ 8.41 (dt, *J* = 8.0, 1.4 Hz, 1H), 7.88 (s, 1H), 7.52 (d, *J* = 2.0 Hz, 1H), 7.41 – 7.31 (m, 3H), 6.45 (d, *J* = 2.0 Hz, 1H), 4.23 (s, 3H), 3.90 (s, 3H); **^{13}C NMR** (101 MHz, $CDCl_3$) δ 154. 5, 138.9, 137.6, 136.2, 136.1, 124.4, 123.4, 123.0, 119.0, 109.8, 91.9, 35.9, 33.6. **HRMS** calculated for $[C_{13}H_{13}N_5+H]^+$: 240.1244, found: 240.1243. **m.p.** 139.9 – 141.2 °C.

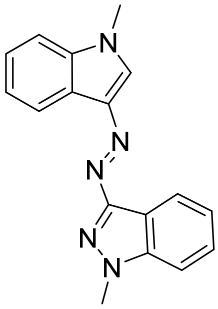

(*E*)-1-methyl-3-((1-methyl-1*H*-indol-3-yl)diazenyl)-1*H*-indazole (**4**). The 1-methyl-1*H*-indole **3b** (1.31 g, 10.0 mmol) reacted with 1-methyl-1*H*-indazol-3-diazonium tetrafluoroborate **2d** (2.71 g, 1.10 eq.) according to the procedure detailed to afford **4** as yellow solid (1.89 g, 65.3%). **^{1}H NMR** (400 MHz, $CDCl_3$) δ 8.69 – 8.67 (m, 1H), 8.48 (dt, *J* = 8.0, 1.2 Hz, 1H), 7.92 (s, 1H), 7.47 – 7.43 (m, 1H), 7.39 (dt, *J* = 8.0, 0.8 Hz, 1H), 7.36 – 7.31 (m, 3H), 7.30 – 7.27 (m, 1H), 4.17 (s, 3H), 3.89 (s, 3H); **^{13}C NMR** (101 MHz, $CDCl_3$) δ 155.4, 141.6, 137.4, 136.2, 134.0, 127.1, 124.0, 123.9, 123.6, 122.9, 122.5, 119.7, 115.6, 109.4, 108.8, 35.9, 33.5. **HRMS** calculated for $[C_{17}H_{15}N_5+H]^+$: 290.1400, found: 290.1400. **m.p.** 98.1 – 98.2 °C.

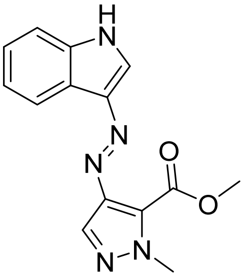

methyl (*E*)-4-((1*H*-indol-3-yl)diazenyl)-1-methyl-1*H*-pyrazole-5-carboxylate (5'). The indole (1.17 g, 10.0 mmol) was reacted with 5-(methoxycarbonyl)-1-methyl-1*H*-pyrazole-4-diazonium tetrafluoroborate **2e** (2.72 g, 1.10 eq.) according to the procedure detailed above and then purification by column chromatography (PE:EA = 1:1) to afford **5'** as a dark red solid (849.87 mg, 30.0%). **^{1}H NMR** (400 MHz, DMSO) δ 12.06 (s, 1H), 8.46 – 8.43 (m, 1H), 8.31 (d, *J* = 4.0 Hz, 1H), 7.79 (s, 1H), 7.49 – 7.46 (m, 1H), 7.29 – 7.23 (m, 2H), 4.12 (s, 3H), 4.02 (s, 3H); **^{13}C NMR** (101 MHz, DMSO) δ 160.1, 142.3, 136.6, 136.2, 133.1, 128.2, 126.0, 123.9, 122.6, 122.4, 118.1, 112.1, 52.4, 40.0; **HRMS** calculated for $[C_{14}H_{13}N_5O_2+H]^+$: 284.1142, Found: 284.1148. **m.p.** 214.3 – 215.1 °C.

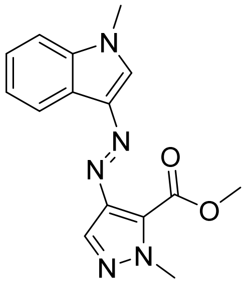

methyl (*E*)-1-methyl-4-((1-methyl-1*H*-indol-3-yl)diazenyl)-1*H*-pyrazole-5-carboxylate (**5**). The 1-methyl-1*H*-indole **3b** (0.656 g, 5.00 mmol) was reacted with 5-(methoxycarbonyl)-1-methyl-1*H*-pyrazole-4-diazonium tetrafluoroborate **2e** (1.36 g, 1.10 eq.) according to the procedure detailed above to afford **5** as a red solid (0.736 g, 52.3%). **^{1}H NMR** (400 MHz, $CDCl_3$) δ 8.58 (d, *J* = 8.0 Hz, 1H), 7.84 (d, *J* = 16.0 Hz, 2H), 7.35 – 7.31 (m, 3H), 4.23 (s, 3H), 4.10 (s, 3H), 3.88 (s, 3H); **^{13}C NMR** (101 MHz, $CDCl_3$) δ 160.9, 143.0, 137.4, 136.2, 134.9, 128.4, 126.7, 124.1, 123.2, 123.0, 119.3, 109.5, 52.3, 40.3, 33.5. **HRMS** calculated for $[C_{15}H_{15}N_5O_2+H]^+$: 298.1299, Found: 298.1296. **m.p.** 185.7 – 186.5 °C.

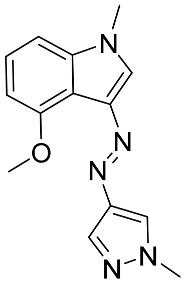

(*E*)-4-methoxy-1-methyl-3-((1-methyl-1*H*-pyrazol-4-yl)diazenyl)-1*H*-indole (**6**). The 4-methoxy-1-methyl-1*H*-indole **3c** (1.61 g, 10.0 mmol) was reacted with 1-methyl-1*H*-pyrazole-4-diazonium tetrafluoroborate **2b** (2.16 g, 1.10 eq.) according to the procedure detailed above to afford **6** as a yellow solid (1.96 g, 72.6%). **^{1}H NMR** (400 MHz, $CDCl_3$) δ 8.00 (s, 1H), 7.88 (s, 1H), 7.46 (s, 1H), 7.22 (t, *J* = 8.0 Hz, 1H), 6.98 (d, *J* = 8.0 Hz, 1H), 6.72 (d, *J* = 8.0 Hz, 1H), 4.04 (s, 3H), 3.95 (s, 3H), 3.81 (s, 3H); **^{13}C NMR** (101 MHz, $CDCl_3$) δ 154.5, 142.5, 138.4, 135.8, 133.6, 124.7, 123.5,

116.4, 114.8, 103.2, 102.5, 56.0, 39.5, 33.7. **HRMS** calculated for $[C_{14}H_{15}N_5O+H]^+$: 270.1349, found: 270.1348. **m.p.** 117.2 – 117.4 °C.

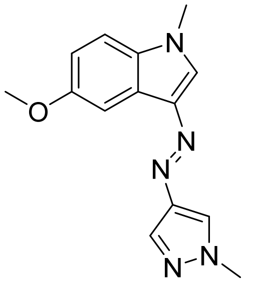

(*E*)-5-methoxy-1-methyl-3-((1-methyl-1*H*-pyrazol-4-yl)diazenyl)-1*H*-indole (7). The 5-methoxy-1-methyl-1*H*-indole **3d** (1.61 g, 10.0 mmol) was reacted with reacted with 1-methyl-1*H*-pyrazole-4-diazonium tetrafluoroborate **2b** (2.16 g, 1.10 eq.) according to the procedure detailed above to afford **7** as a yellow solid (2.03 g, 75.4%). **^{1}H NMR** (400 MHz, $CDCl_3$) δ 7.95 (d, *J* = 4.0 Hz, 2H), 7.84 (s, 1H), 7.66 (s, 1H), 7.21 (d, *J* = 8.0 Hz, 1H), 6.96 (dd, *J* = 8.0, 2.8 Hz, 1H), 3.94 (s, 3H), 3.89 (s, 3H), 3.81 (s, 3H); **^{13}C NMR** (101 MHz, $CDCl_3$) δ 156.3, 142.6, 134.9, 132.9, 132.6, 132.4, 124.5, 120.0, 113.4, 110.2, 104.7, 55.8, 39.4, 33.5. **HRMS** calculated for $[C_{14}H_{15}N_5O+H]^+$: 270.1249, Found: 270.1350. **m.p.** 151.5 – 151.7 °C.

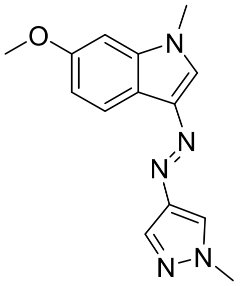

(*E*)-6-methoxy-1-methyl-3-((1-methyl-1*H*-pyrazol-4-yl)diazenyl)-1*H*-indole (8). The 6-methoxy-1-methyl-1*H*-indole **3e** (1.61 g, 10.0 mmol) was reacted with 1-methyl-1*H*-pyrazole-4-diazonium tetrafluoroborate **2b** (2.16 g, 1.10 eq.) according to the procedure detailed above to afford **8** as a yellow solid (1.85 g, 68.7%). **^{1}H NMR** (400 MHz, $CDCl_3$) δ 8.31 (d, *J* = 8.0 Hz, 1H), 7.95 (s, 1H), 7.83 (s, 1H), 7.60 (s, 1H), 6.91 (dd, *J* = 8.0, 4.0 Hz, 1H), 6.75 (s, 1H), 3.93 (s, 3H), 3.88 (s, 3H), 3.77 (s, 3H); **^{13}C NMR** (101 MHz, $CDCl_3$) δ 157.5, 142.6, 138.3, 135.3, 132.6, 132.3, 124.7, 123.8, 113.8, 111.4, 93.2, 55.6, 39.4, 33.3. **HRMS** calculated for $[C_{14}H_{15}N_5O+H]^+$: 270.1349, Found: 270.1346. **m.p.** 190.9 – 191.8 °C.

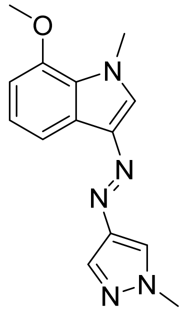

(*E*)-7-methoxy-1-methyl-3-((1-methyl-1*H*-pyrazol-4-yl)diazenyl)-1*H*-indole (9). The 7-methoxy-1-methyl-1*H*-indole **3f** (1.61 g, 10.0 mmol) was reacted with 1-methyl-1*H*-pyrazole-4-diazonium tetrafluoroborate **2b** (2.16 g, 1.10 eq.) according to the procedure detailed above to afford **9** as a yellow solid (1.77 g, 65.7%). **^{1}H NMR** (400 MHz, $CDCl_3$) δ 8.02 (d, *J* = 8.0 Hz, 1H), 7.95 (s, 1H), 7.84 (s, 1H), 7.58 (s, 1H), 7.14 (t, *J* = 8.0 Hz, 1H), 6.72 (d, *J* = 8.0 Hz, 1H), 4.11 (s, 3H), 3.94 (d, *J* = 4.8 Hz, 6H); **^{13}C NMR** (101 MHz, $CDCl_3$) δ 147.5, 142.7, 135.0, 133.3, 132.7, 126.8, 124.7, 123.1, 122.0, 115.3, 104.7, 55.5, 39.5, 37.5. **HRMS** calculated for $[C_{14}H_{15}N_5O+H]^+$: 270.1349, Found: 270.1350. **m.p.** 190.8 – 191.2 °C.

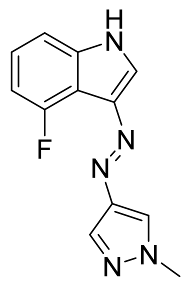

(*E*)-4-fluoro-3-((1-methyl-1*H*-pyrazol-4-yl)diazenyl)-1*H*-indole (10'). The 4-fluoro-1*H*-indole **3g** (1.35 g, 10.0 mmol) was reacted with 1-methyl-1*H*-pyrazole-4-diazonium tetrafluoroborate **2b** (2.16 g, 1.10 eq.) according to the procedure detailed above and then purification by column chromatography (PE:EA = 1:1) to afford **10'** as a dark red solid (0.615 g, 25.3%). **^{1}H NMR** (400 MHz, DMSO) δ 12.09 (s, 1H), 8.24 (s, 1H), 7.94 (s, 1H), 7.83 (s, 1H), 7.29 (d, *J* = 8.0 Hz, 1H), 7.21 – 7.17 (m, 1H), 6.93 (dd, *J* = 12.0, 8.0 Hz, 1H), 3.90 (s, 3H). **^{13}C NMR** (101 MHz, DMSO) one carbon signal was overlapped δ 155.8 (d, *J* = 240.0 Hz), 141.6 (d, *J* = 2.0 Hz), 139.1 (d, *J* = 12.3 Hz), 134.6 (d, *J* = 6.0 Hz), 131.5, 126.1, 123.2 (d, *J* = 8.0 Hz), 121.0, 110.1 (d, *J* = 2.0 Hz, 1H), 108.9 (d, *J* = 5.0 Hz), 106.7 (d, *J* = 30.0 Hz); **^{19}F NMR** (377 MHz, DMSO) δ -112.93. **HRMS** calculated for $[C_{12}H_{10}FN_5+H]^+$: 224.0993, Found: 224.0993. **m.p.** 179.5 – 180.1 °C.

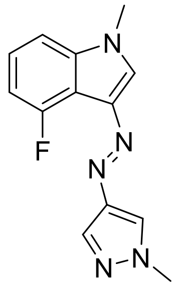

(*E*)-4-fluoro-1-methyl-3-((1-methyl-1*H*-pyrazol-4-yl)diazenyl)-1*H*-indole (**10**). The compound **10'** (0.489 g, 2.00 mmol) was reacted with CH_3I (0.341 g, 1.20 eq.) according to the procedure detailed above and then purification by column chromatography (PE:EA = 2:1) to afford **10** as a yellow solid (0.949 g, 36.9%). **^{1}H NMR** (400 MHz, $CDCl_3$) δ 7.99 (s, 1H), 7.88 (s, 1H), 7.55 (s, 1H), 7.23 – 7.18 (m, 1H), 7.10 (d, *J* = 8.0 Hz, 1H), 6.95 (dd, *J* = 12.0, 8.0 Hz, 1H), 3.94 (s, 3H), 3.82 (s, 3H); **^{13}C NMR** (101 MHz, $CDCl_3$) δ 157.6 (d, *J* = 50.0 Hz), 142.4 (d, *J* = 1.0 Hz), 139.4 (d, *J* = 20.0 Hz), 134.5 (d, *J* = 10.0 Hz), 133.3, 124.8, 123.4 (d, *J* = 6.0 Hz), 122.0, 111.9 (d, *J* = 20.0 Hz), 107.4 (d, *J* = 20.0 Hz), 105.8 (d, *J* = 4.0 Hz, 1H), 39.5, 33.7; **^{19}F NMR** (377 MHz, CD_3CN) δ -113.88. **HRMS** calculated for $[C_{13}H_{12}FN_5+H]^+$: 258.1150, Found: 258.1147. **m.p.** 143.2 – 144.1 °C.

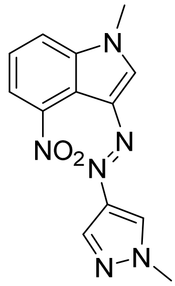

(*E*)-1-methyl-3-((1-methyl-1*H*-pyrazol-4-yl)diazenyl)-4-nitro-1*H*-indole (**11**). The 1-methyl-4-nitro-1*H*-indole **3i** (0.881 g, 5.00 mmol) was reacted with 1-methyl-1*H*-pyrazole-4-diazonium tetrafluoroborate **2b** (1.08 g, 1.10 eq.) according to the procedure detailed above and then purification by column chromatography (PE:EA = 5:1) to afford **11** as a red solid (0.381 g, 26.8%). **^{1}H NMR** (400 MHz, CD_3CN) δ 8.09 (s, 1H), 7.94 (s, 1H), 7.43 (d, *J* = 8.0 Hz, 1H), 7.18 (d, *J* = 4.0 Hz, 1H), 7.13 (d, *J* = 4.0 Hz, 1H), 7.04 (d, *J* = 8.0 Hz, 1H), 4.02 (s, 3H), 3.90 (s, 3H). **^{13}C NMR** (101 MHz, CD_3CN) δ 144.3, 143.3, 136.9, 134.4, 132.7, 130.9, 127.6, 124.4, 124.3, 117.4, 101.5, 39.8, 32.8. **HRMS** calculated for $[C_{13}H_{12}N_6O_2]$: 284.1022, Found: 284.1043. **m.p.** 88.5 – 88.8 °C.

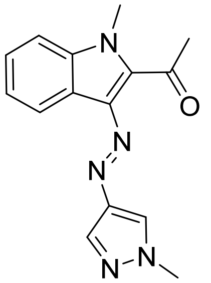

(*E*)-1-(1-methyl-3-((1-methyl-1*H*-pyrazol-4-yl)diazenyl)-1*H*-indol-2-yl)ethan-1-one (**12**). The 1-(1-methyl-1*H*-indol-2-yl)ethan-1-one **3h** (0.866 g, 5.00 mmol) was reacted with 1-methyl-1*H*-pyrazole-4-diazonium tetrafluoroborate **2b** (1.08 g, 1.10 eq.) according to the procedure detailed above to afford **12** as a red solid (0.736 g, 52.3%). **^{1}H NMR** (400 MHz, $CDCl_3$) δ 8.50 (d, *J* = 8.0 Hz, 1H), 7.94 (d, *J* = 16.0 Hz, 2H), 7.43 – 7.39 (m, 2H), 7.29 (ddd, *J* = 8.0, 4.0, 1.6 Hz, 1H), 4.03 (s, 3H), 3.99 (s, 3H), 2.96 (s, 3H); **^{13}C NMR** (101 MHz, $CDCl_3$) δ 194.1, 143.1, 138.4, 137.3, 135.0, 132.7, 126.7, 126.0, 125.0, 123.7, 116.9, 110.1, 39.6, 32.9, 32.4. **HRMS** calculated for $[C_{15}H_{15}N_5O+H]^+$: 282.1349, Found: 282.1351. **m.p.** 166.3 – 166.8 °C.

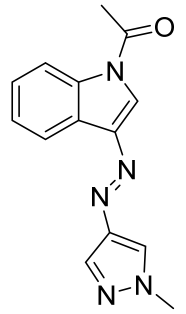

(*E*)-1-(3-((1-methyl-1*H*-pyrazol-4-yl)diazenyl)-1*H*-indol-1-yl)ethan-1-one (**13**). The compound **1'** (1.13 g, 5.00 mmol) was reacted with acetyl chloride (0.589 g, 1.50 eq.) according to the procedure detailed above and then purification by column chromatography (PE:EA = 2:1) to afford **13** as an orange solid (1.15 g, 86.4%). **^{1}H NMR** (400 MHz, $CDCl_3$) δ 8.49 (d, *J* = 8.0 Hz, 1H), 8.38 (d, *J* = 8.0 Hz, 1H), 8.05 (s, 1H), 8.00 (s, 1H), 7.94 (s, 1H), 7.45 – 7.35 (m, 2H), 3.98 (s, 3H), 2.71 (s, 3H); **^{13}C NMR** (101 MHz, $CDCl_3$) δ 168.7, 142.5, 138.9, 136.2, 132.8, 127.4, 126.7, 126.4, 125.2, 122.8, 122.7, 116.5, 39.6, 24.0. **HRMS** calculated for $[C_{14}H_{13}N_5O+H]^+$: 268.1193, Found: 268.1195. **m.p.** 222.6 – 222.9 °C.

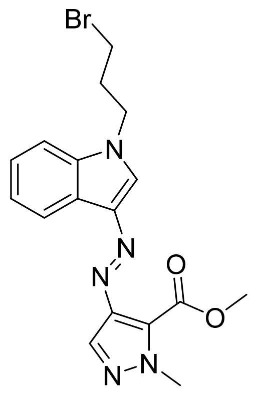

methyl (*E*)-4-((1-(3-bromopropyl)-1*H*-indol-3-yl)diazenyl)-1-methyl-1*H*-pyrazole-5-carboxylate (5-Br). The compound **5'** (0.425 g, 1.50 mmol) was reacted with 1,3-dibromopropane (0.606 g, 2.00 eq.) according to the procedure detailed above and then purification by column chromatography (PE:EA = 2:1) to afford **5-Br** as a yellow solid (0.337 g, 55.6%). **^{1}H NMR** (400 MHz, $CDCl_3$) δ 8.60 (d, *J* = 8.0 Hz, 1H), 7.92 (s, 1H), 7.86 (s, 1H), 7.42 – 7.39 (m, 1H), 7.35 – 7.30 (m, 2H), 4.41 (t, *J* = 8.0 Hz, 2H), 4.23 (s, 3H), 4.10 (s, 3H), 3.36 (t, *J* = 6.0 Hz, 2H), 2.44 – 2.38 (m, 2H); **^{13}C NMR** (101 MHz, $CDCl_3$) δ 160.8, 142.9, 136.5, 136.4, 133.4, 128.6, 126.6, 124.3, 123.4, 123.1, 119.5, 109.5, 52.3, 44.5, 40.3, 32.2, 29.8. **HRMS** calculated for $[C_{17}H_{18}BrN_5O_2+H]^+$: 404.0717, Found: 404.0719. **m.p.** 153.7 – 154.2 °C.

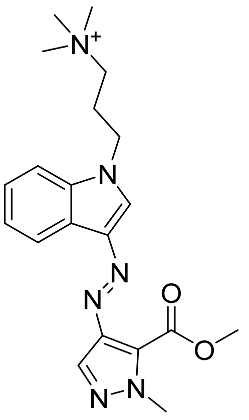

(*E*)-3-(3-((5-(methoxycarbonyl)-1-methyl-1*H*-pyrazol-4-yl)diazenyl)-1*H*-indol-1-yl)-*N*,*N*,*N*-trimethylpropan-1-aminium (5-PS). The compound **5-Br** (0.404 g, 1.00 mmol) was reacted with trimethylamine (0.250 g, 5.00 eq.) according to the procedure detailed above and then purification by crystallization (EA) to afford **5-PS** as a yellow solid (0.328 g, 85.6%). **^{1}H NMR** (400 MHz, DMSO) δ 8.48 (d, *J* = 8.0 Hz, 1H), 8.45 (s, 1H), 7.80 (s, 1H), 7.68 (d, *J* = 8.0 Hz, 1H), 7.39 – 7.31 (m, 2H), 4.39 (t, *J* = 6.0 Hz, 2H), 4.13 (s, 3H), 4.03 (s, 3H), 3.43 – 3.39 (m, 2H), 3.07 (s, 9H), 2.38 – 2.30 (m, 2H); **^{13}C NMR** (101 MHz, DMSO) δ 160.0, 142.2, 136.4, 135.44, 135.41, 128.4, 125.9, 124.1, 123.1, 122.7, 118.7, 110.6, 62.8, 52.4, 52.3, 43.2, 40.1, 23.1. **HRMS** calculated for $[C_{20}H_{27}N_6O_2]^+$: 383.2190, Found: 383.2190. **m.p.** 241.1 – 241.6 °C.

2.3. NMR spectra

(*E*)-1-methyl-3-((1-methyl-1*H*-pyrazol-3-yl)diazenyl)-1*H*-indole (1)

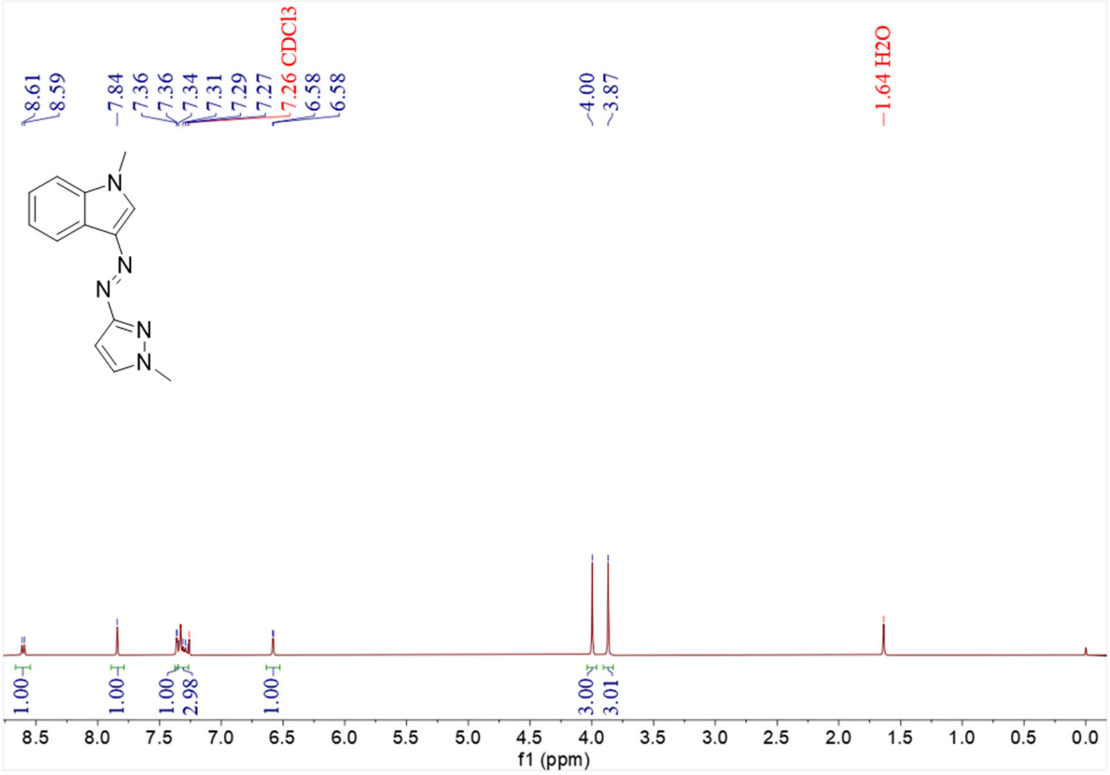

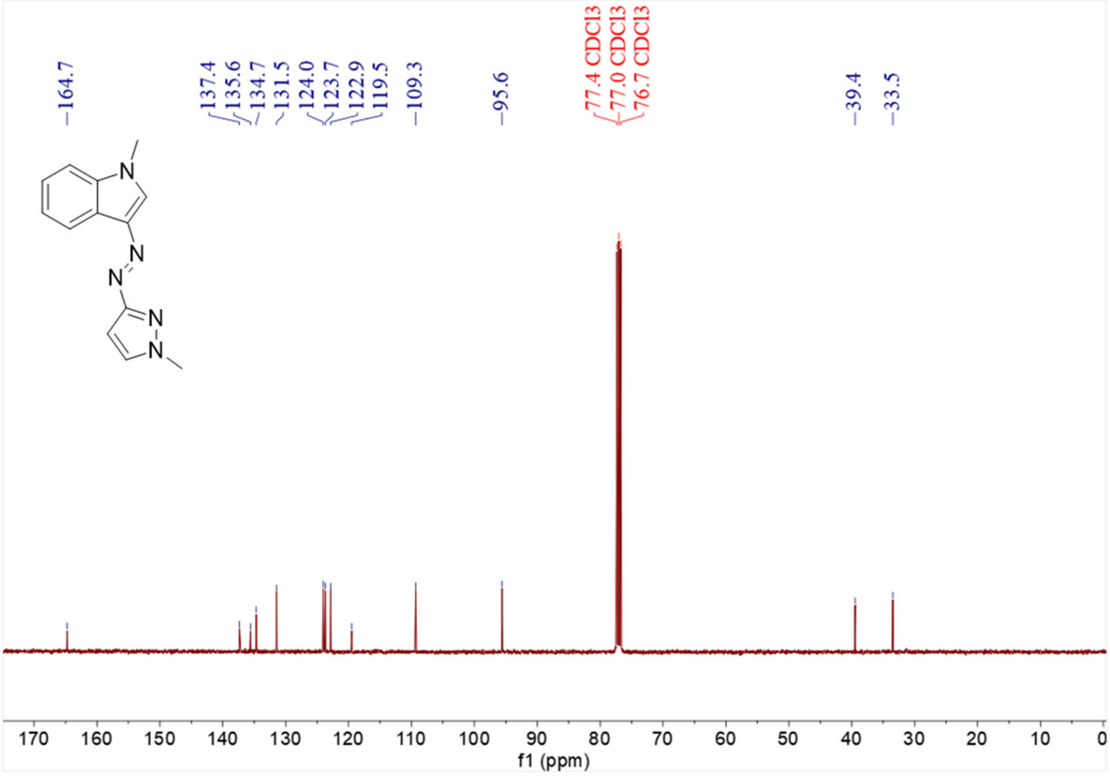

(*E*)-3-((1-methyl-1*H*-pyrazol-4-yl)diazenyl)-1*H*-indole (2’)

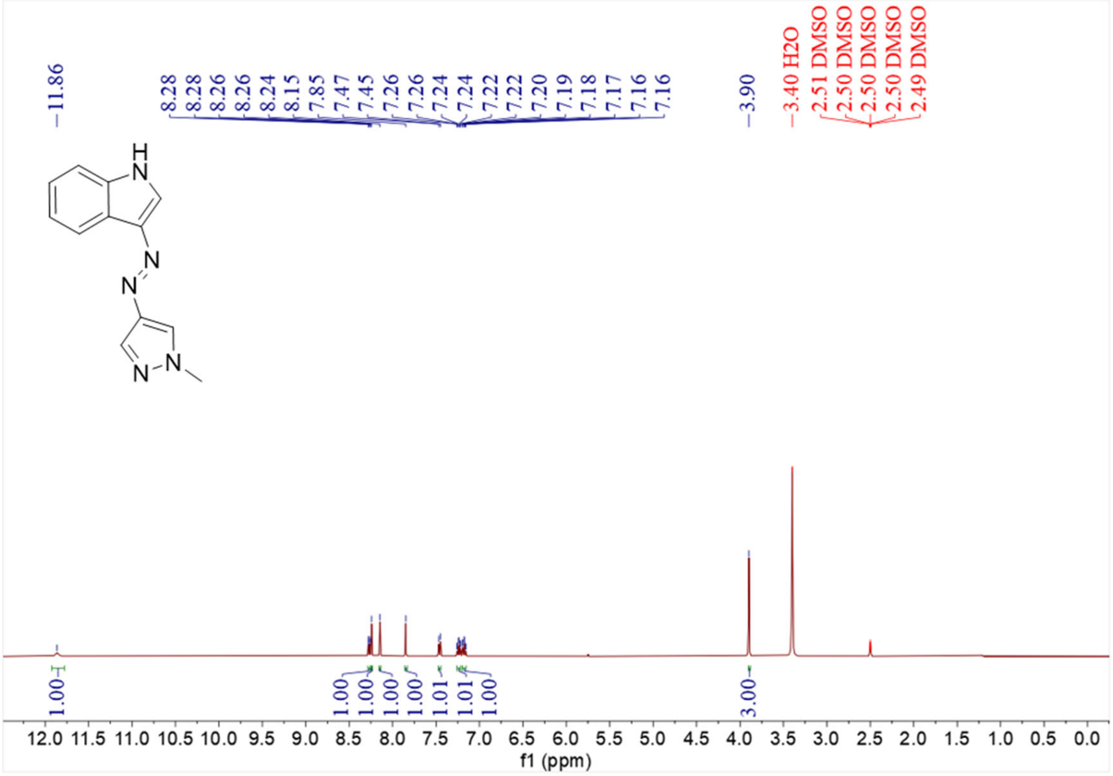

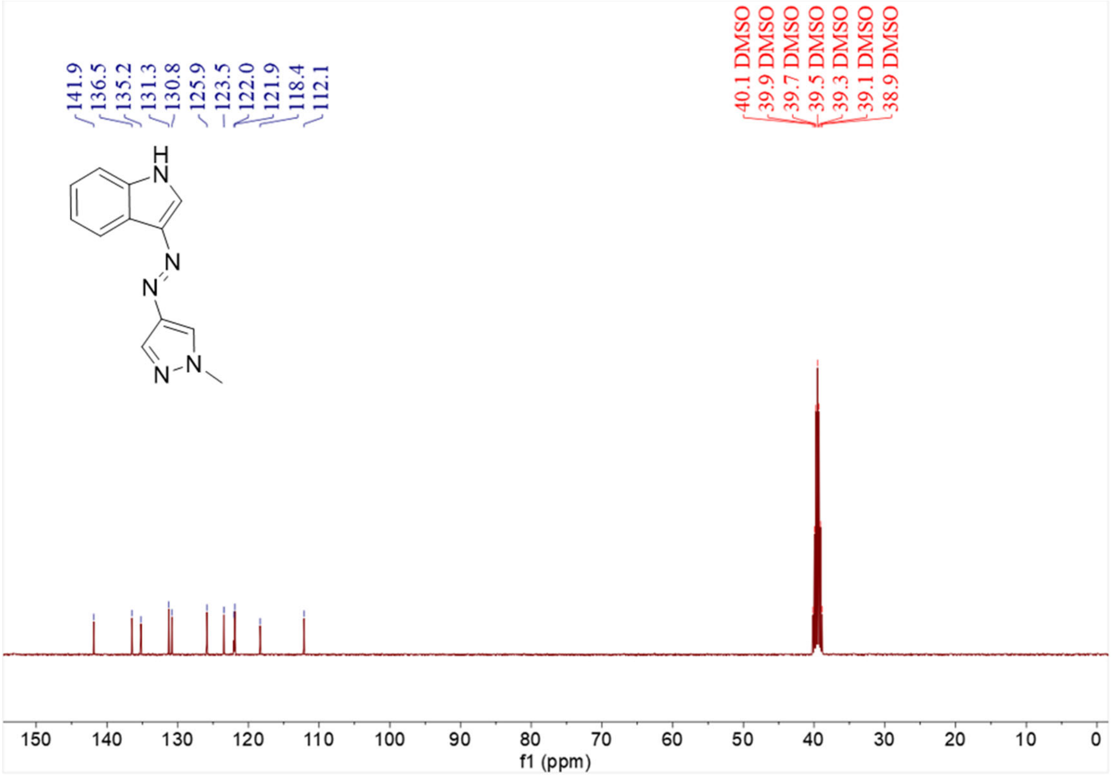

(*E*)-1-methyl-3-((1-methyl-1*H*-pyrazol-4-yl)diazenyl)-1*H*-indole (2)

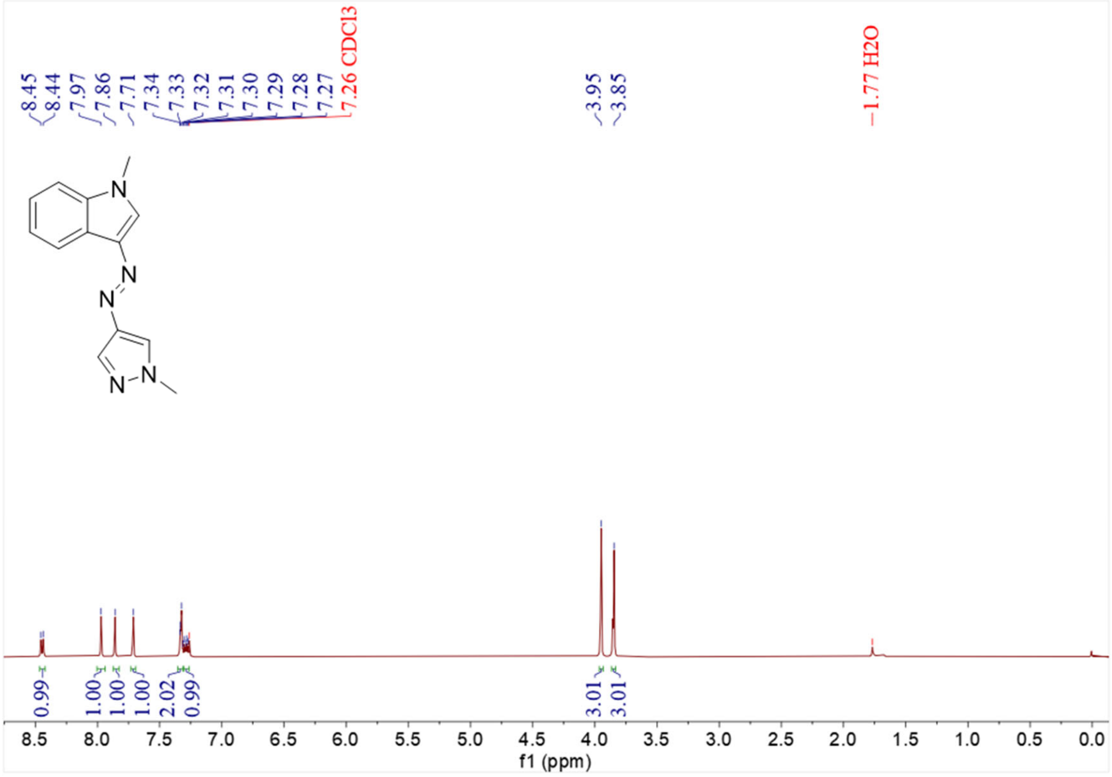

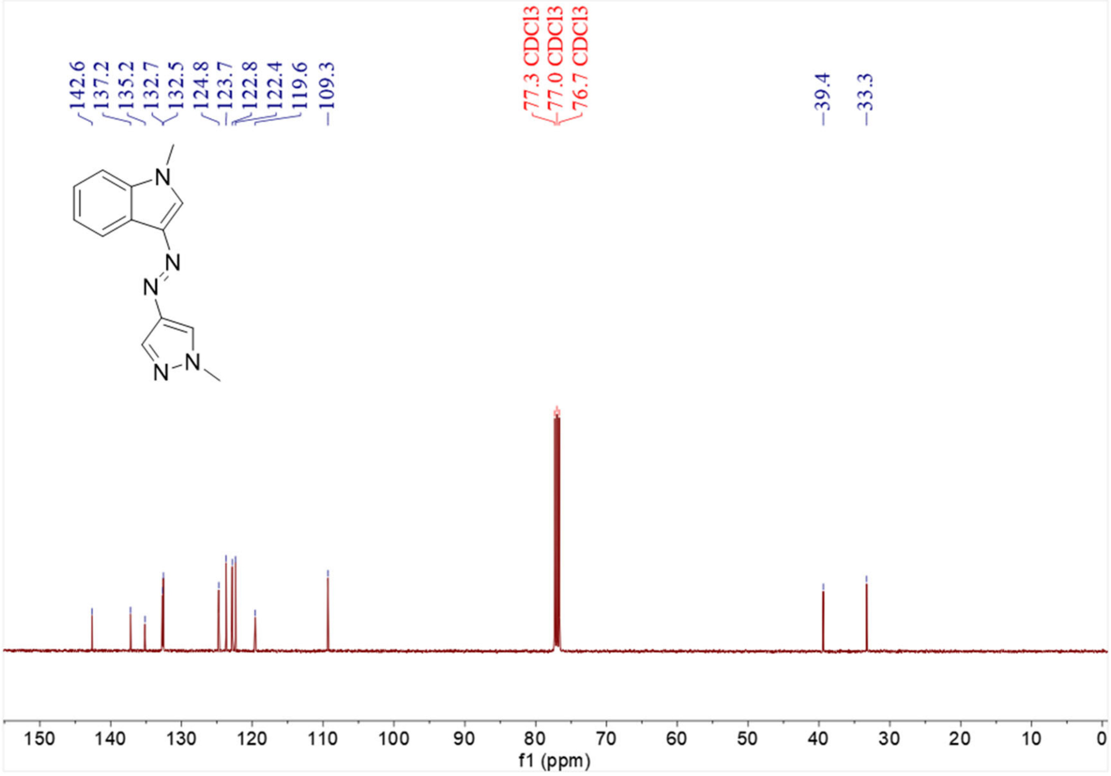

(*E*)-1-methyl-3-((1-methyl-1*H*-pyrazol-5-yl)diazenyl)-1*H*-indole (3)

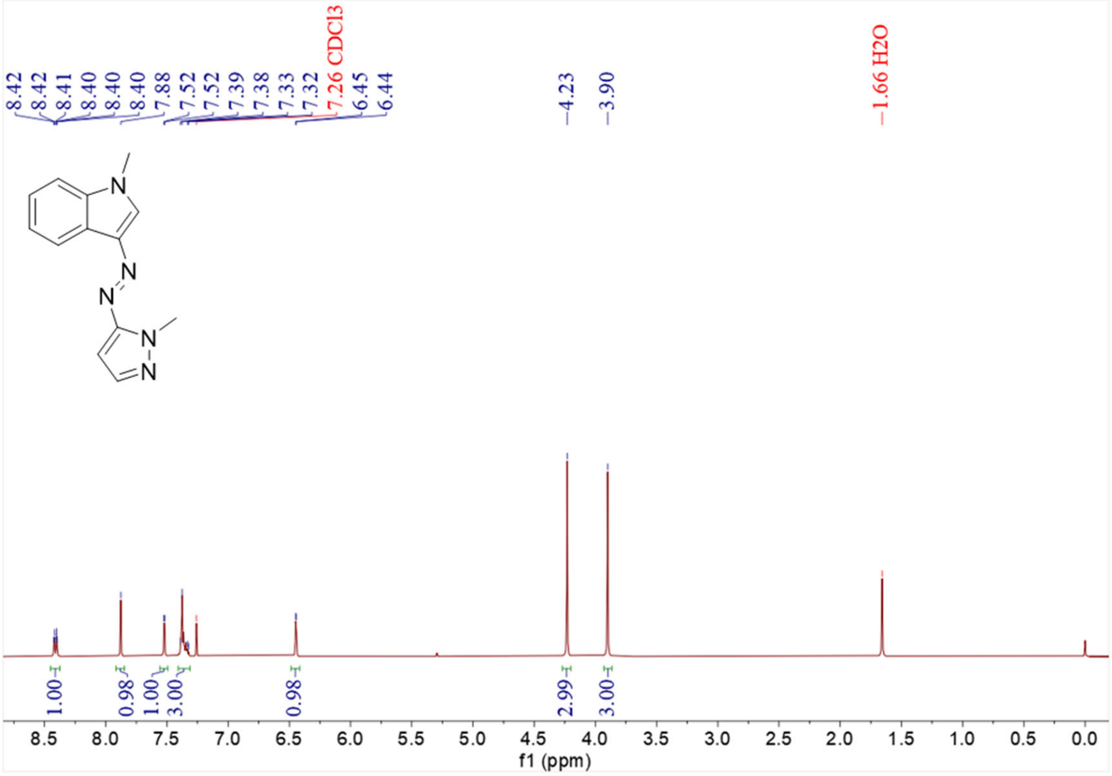

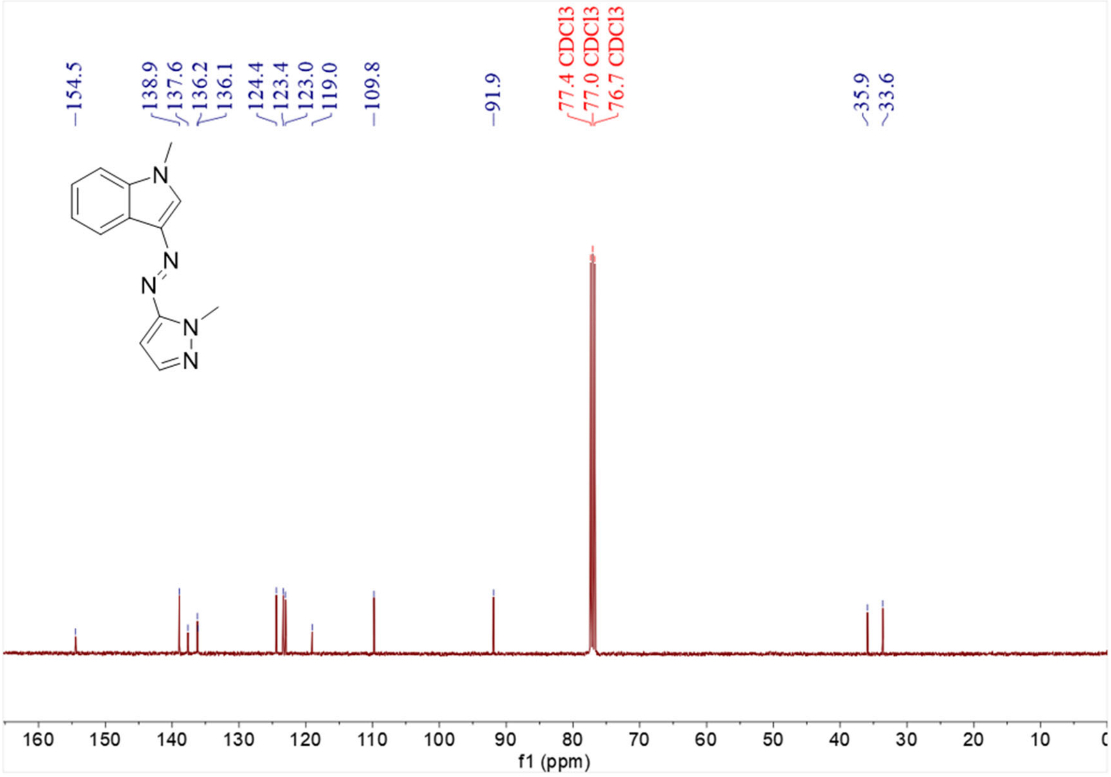

(*E*)-1-methyl-3-((1-methyl-1*H*-indol-3-yl)diazenyl)-1*H*-indazole (4)

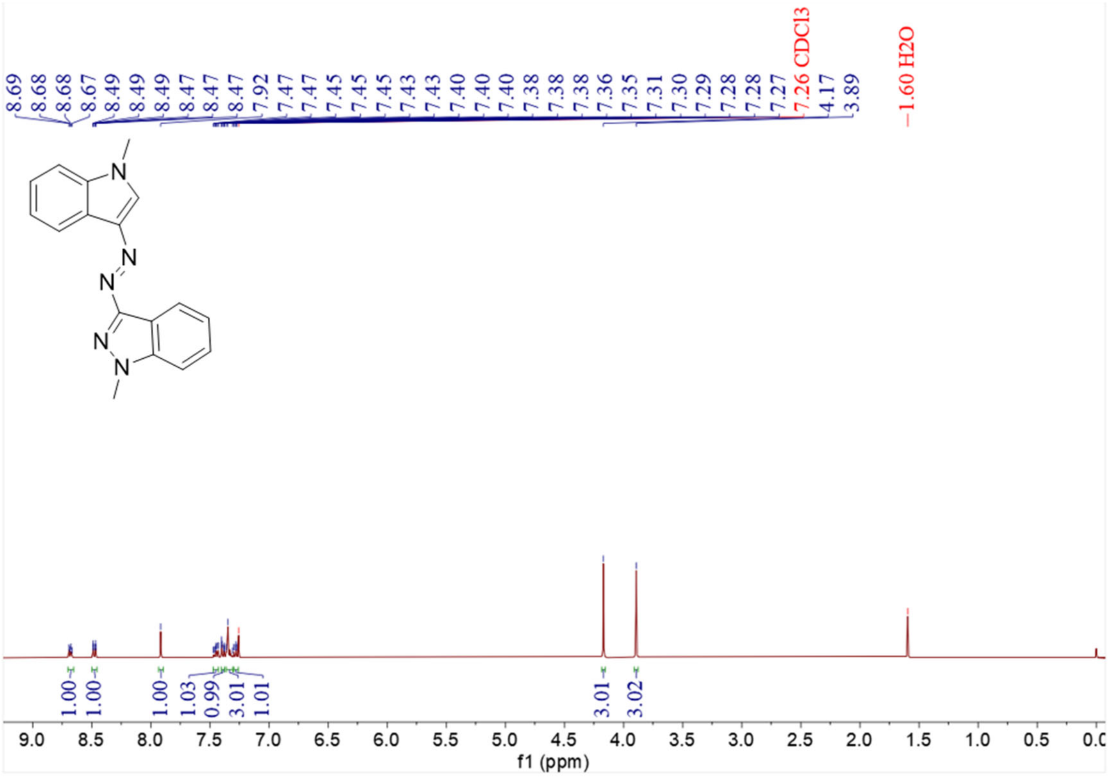

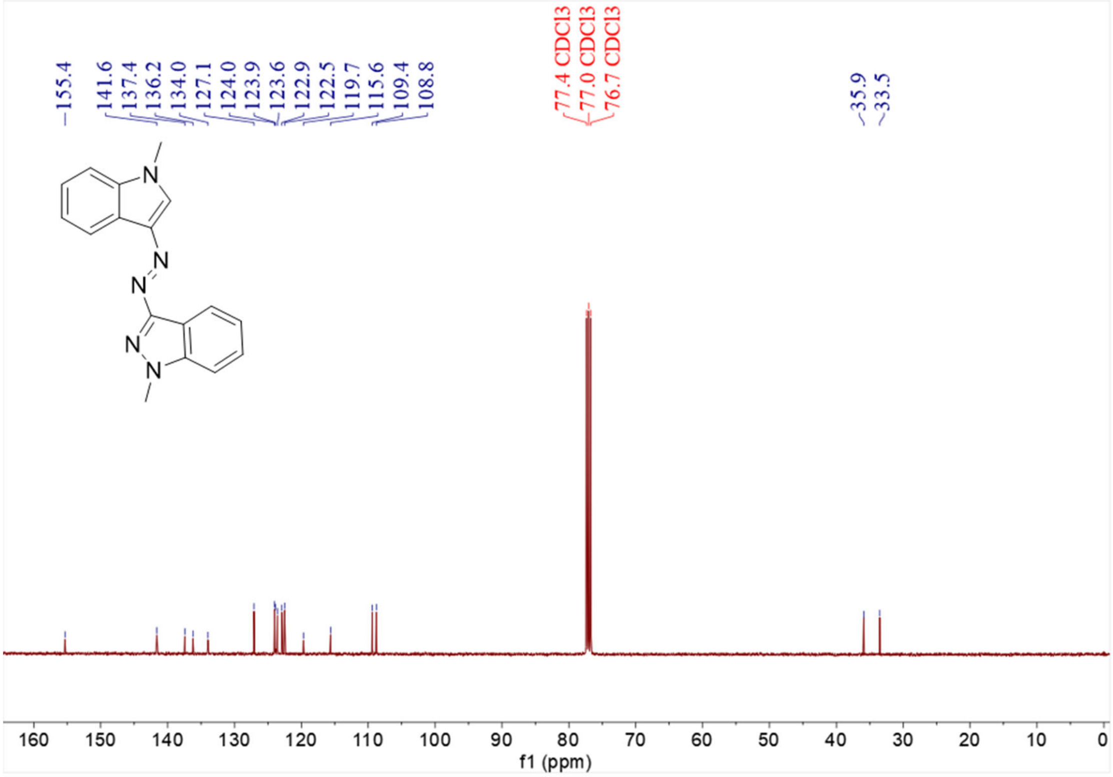

methyl (*E*)-4-((1*H*-indol-3-yl)diazenyl)-1-methyl-1*H*-pyrazole-5-carboxylate (5')

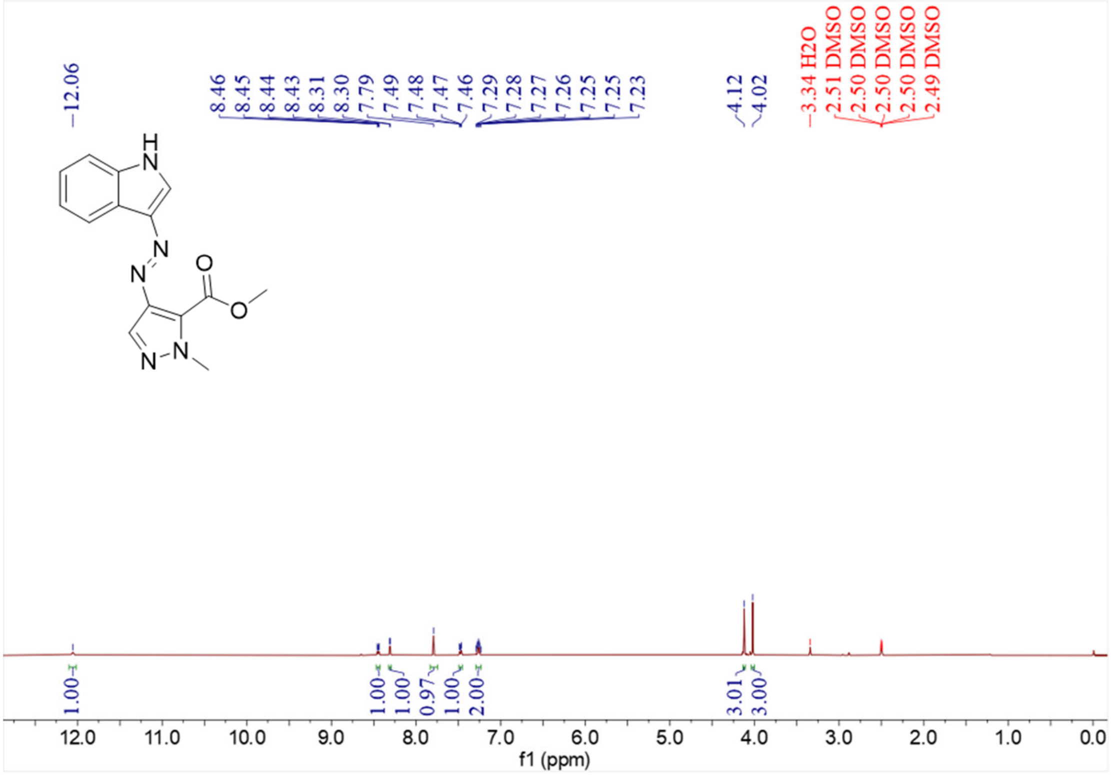

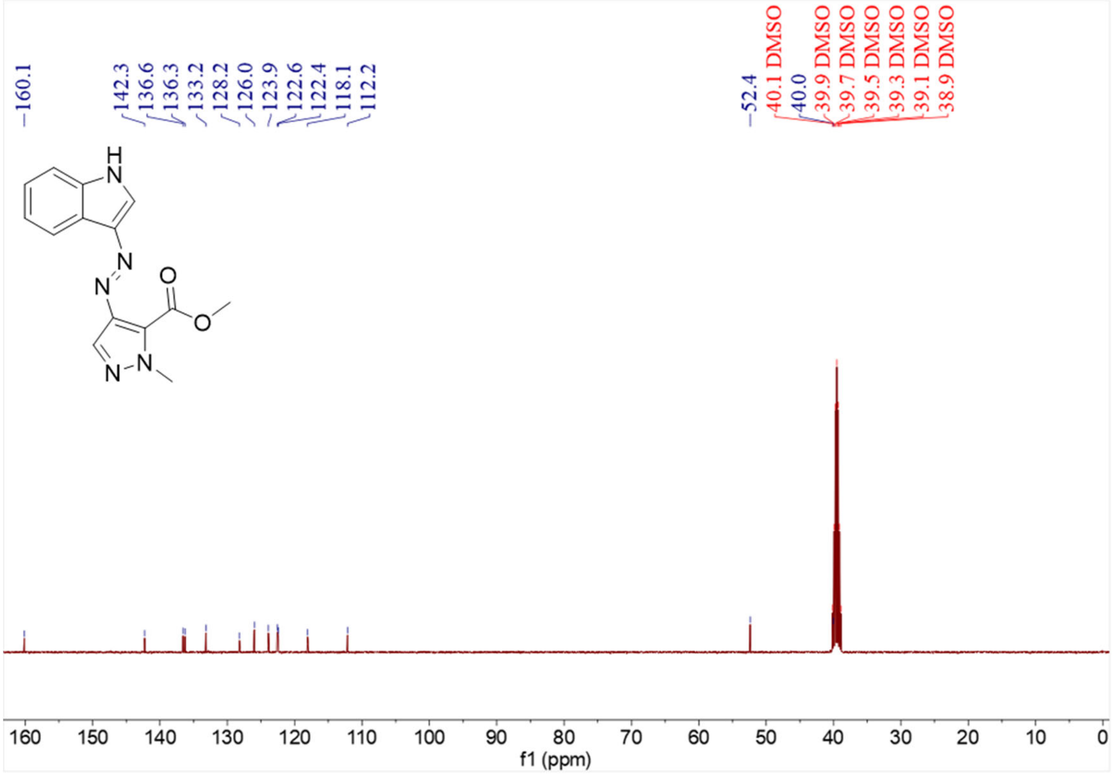

methyl (*E*)-1-methyl-4-((1-methyl-1*H*-indol-3-yl)diazenyl)-1*H*-pyrazole-5-carboxylate (5)

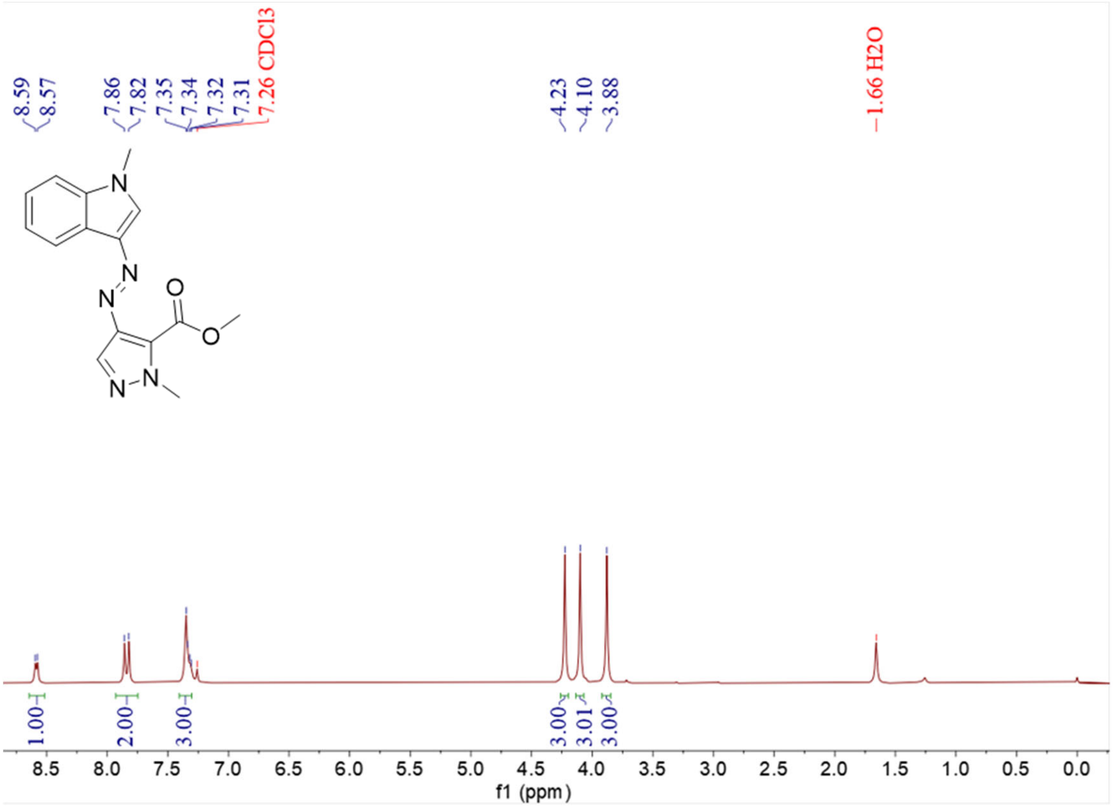

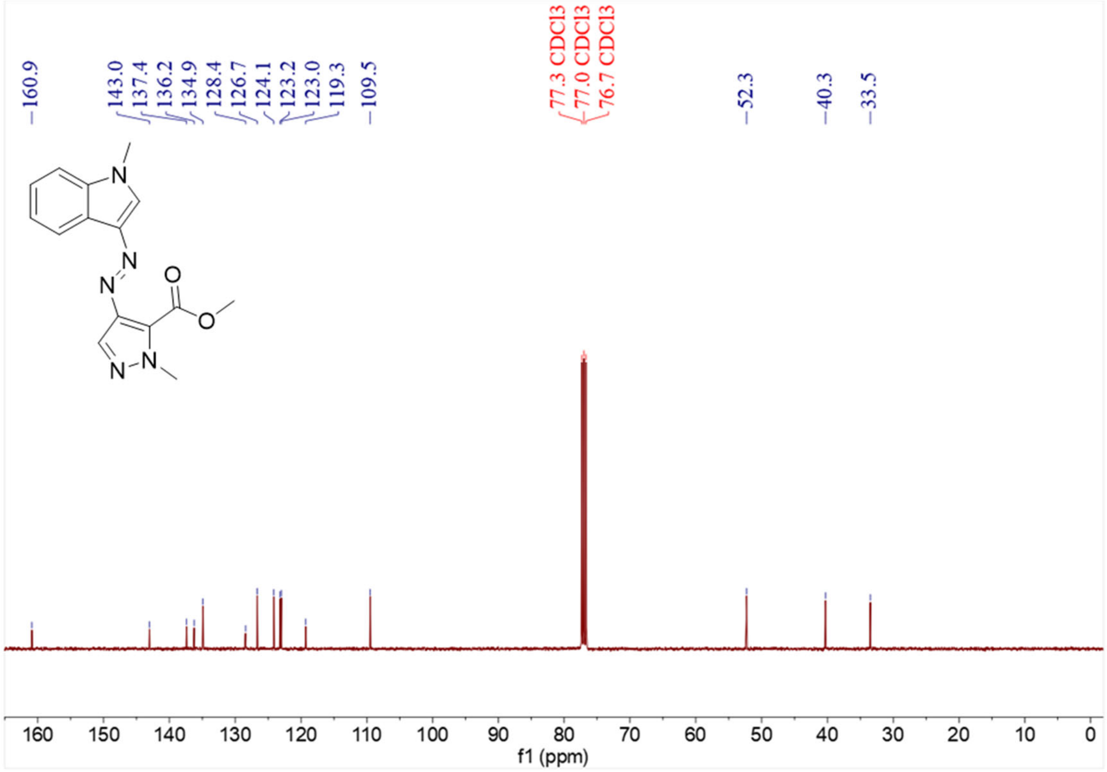

(*E*)-4-methoxy-1-methyl-3-((1-methyl-1*H*-pyrazol-4-yl)diazenyl)-1*H*-indole (6)

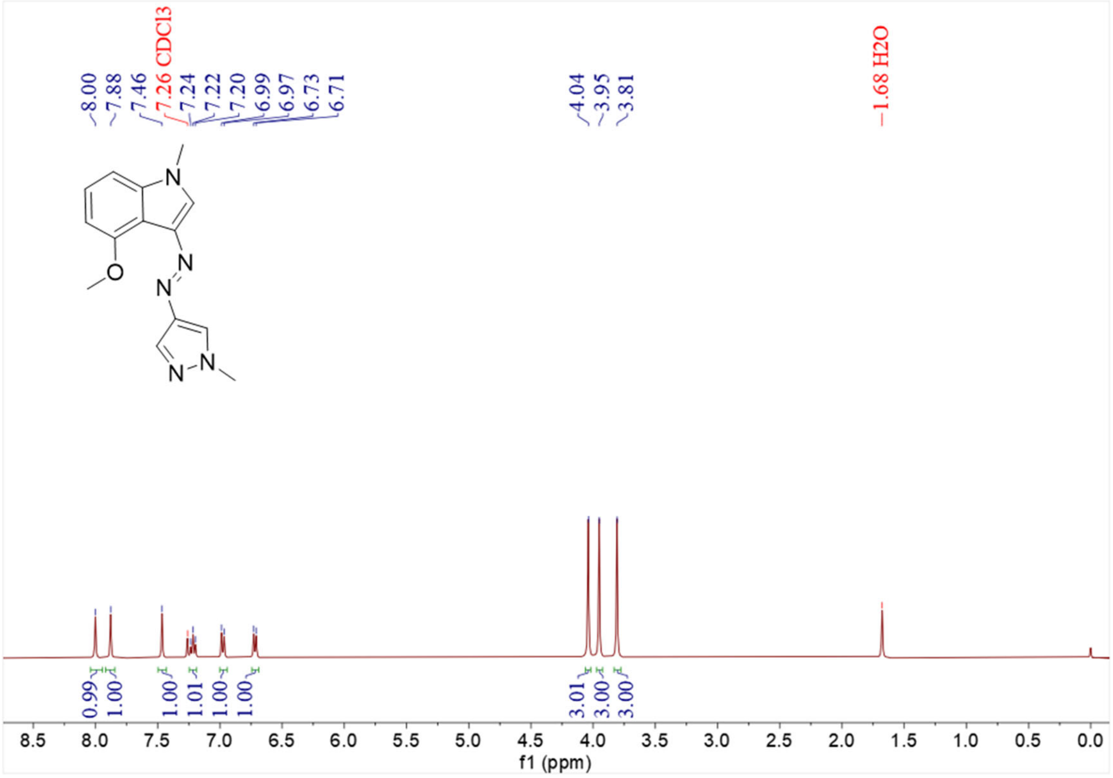

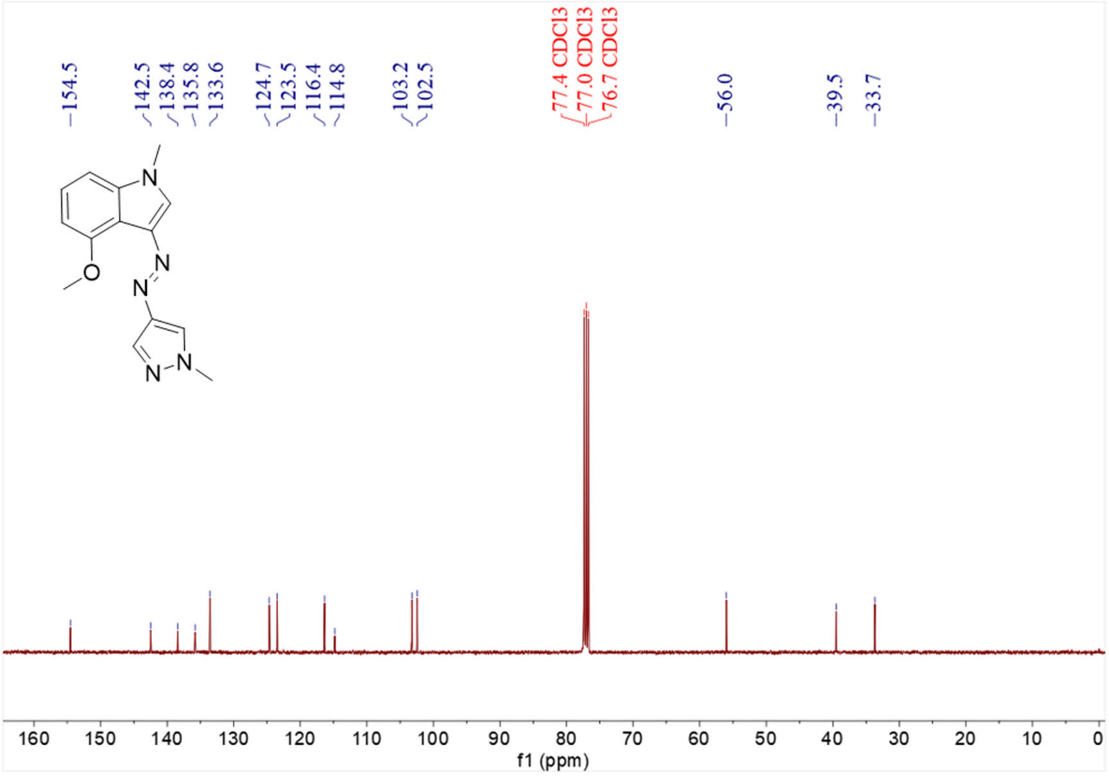

(*E*)-5-methoxy-1-methyl-3-((1-methyl-1*H*-pyrazol-4-yl)diazenyl)-1*H*-indo-le (7)

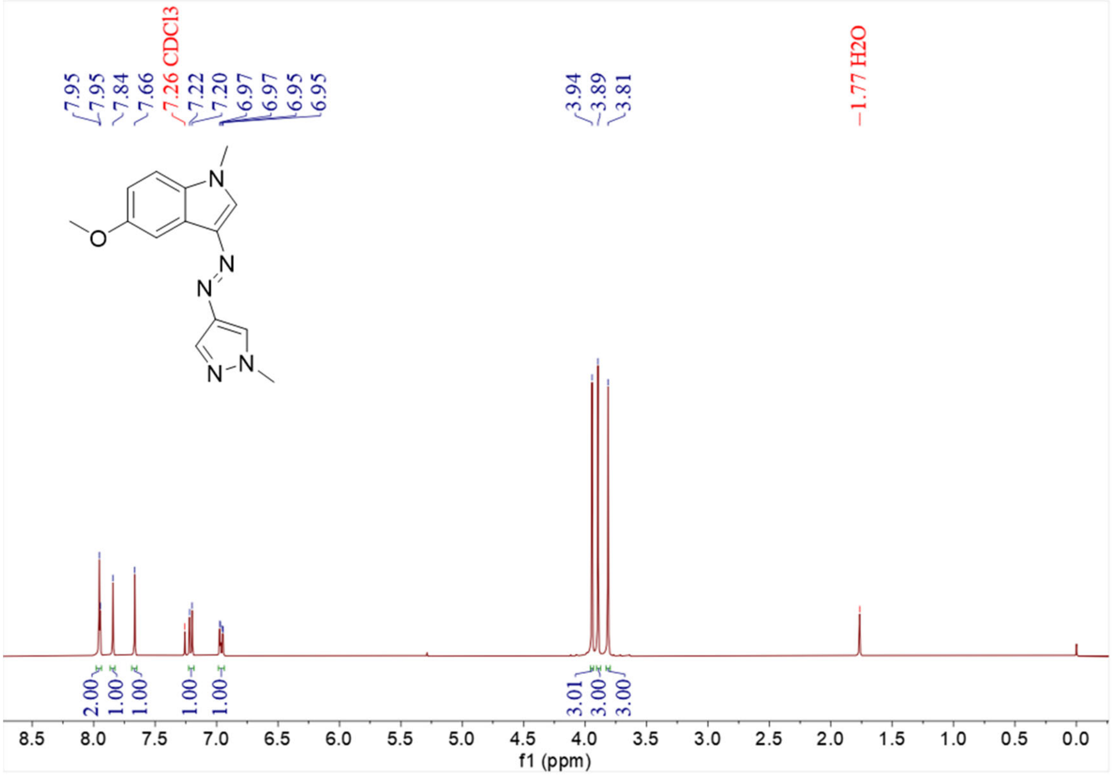

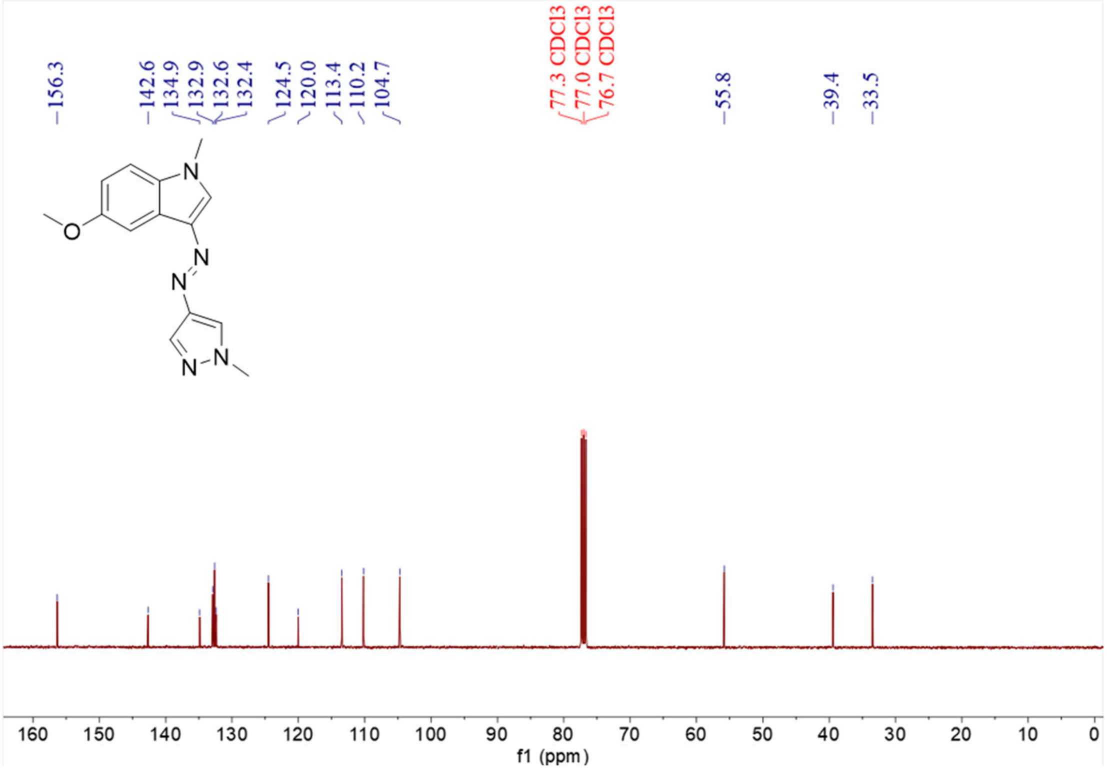

(*E*)-6-methoxy-1-methyl-3-((1-methyl-1*H*-pyrazol-4-yl)diazenyl)-1*H*-indo-le (8)

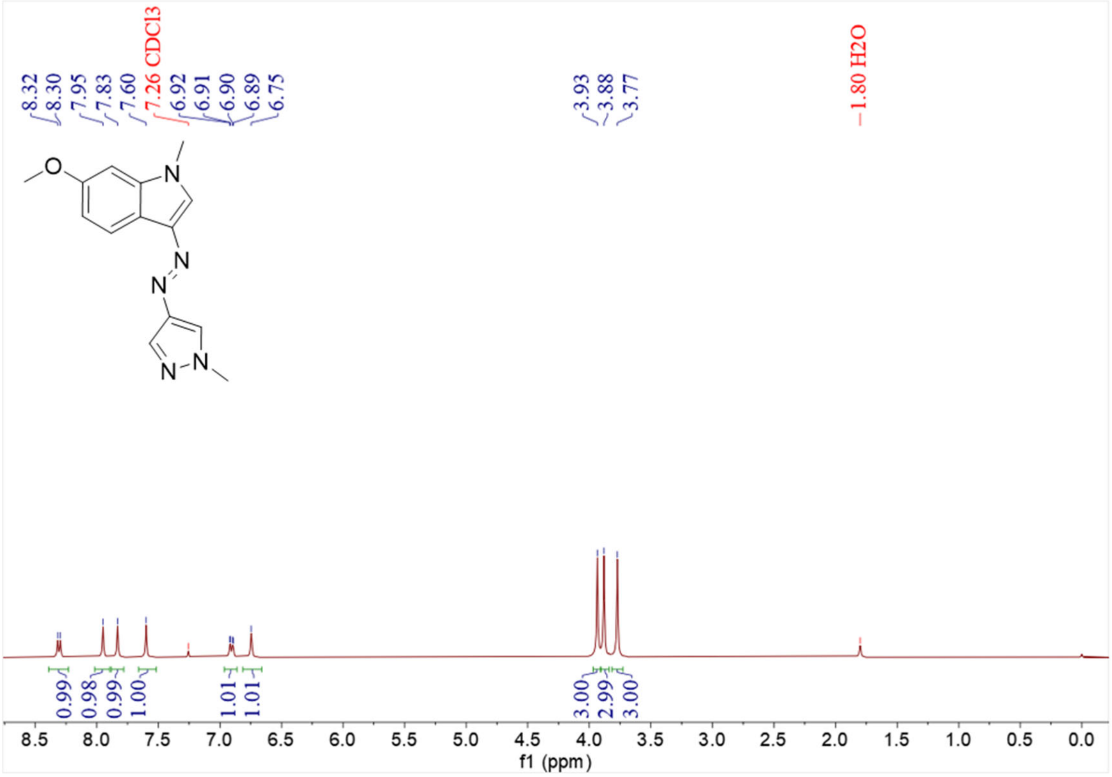

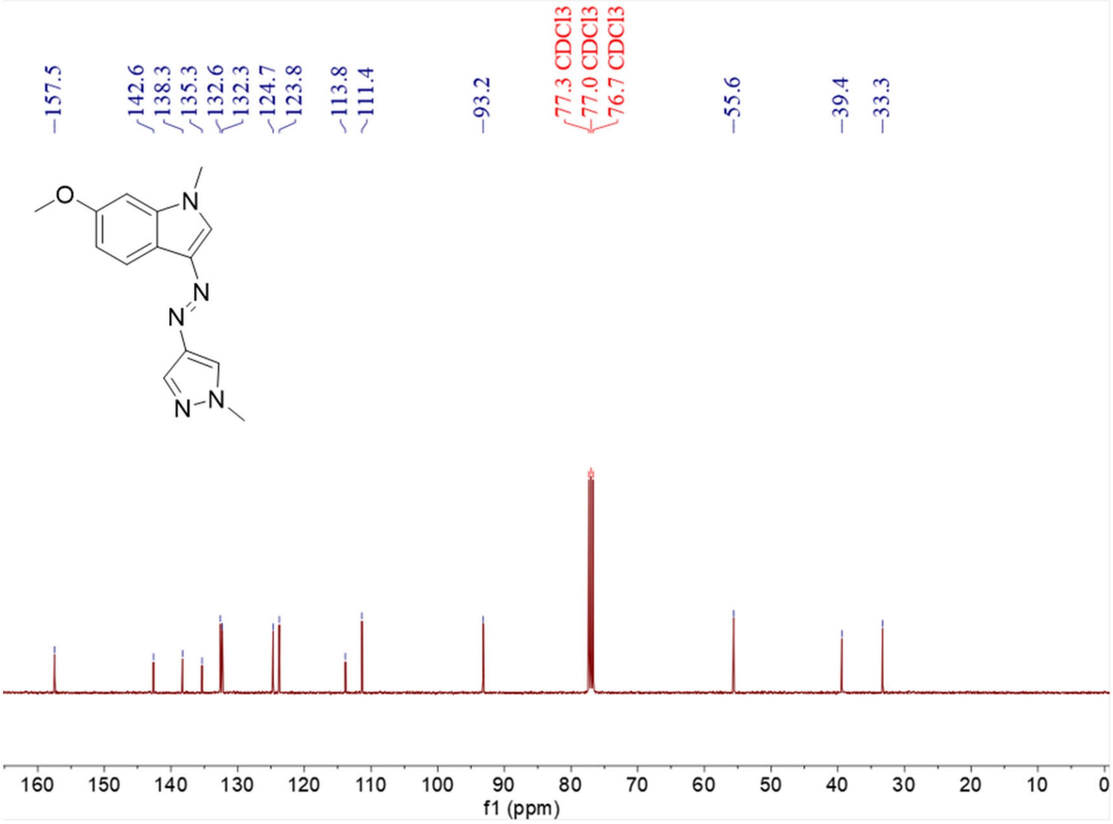

(*E*)-7-methoxy-1-methyl-3-((1-methyl-1*H*-pyrazol-4-yl)diazenyl)-1*H*-indole (9)

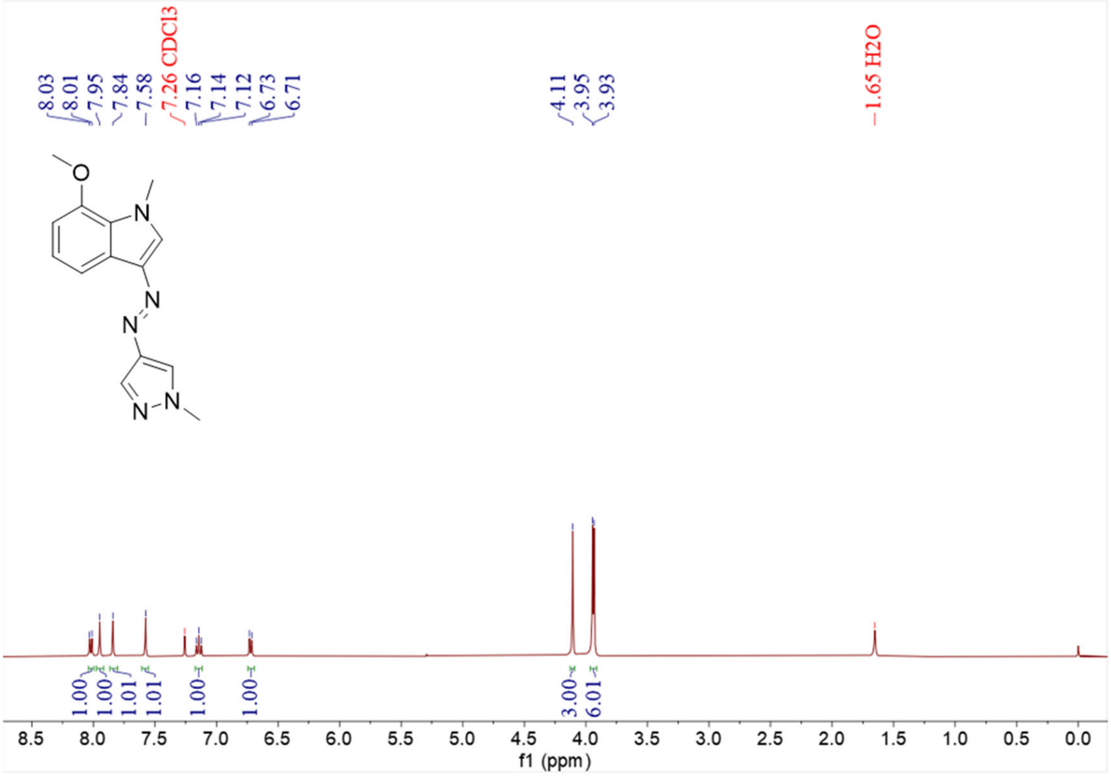

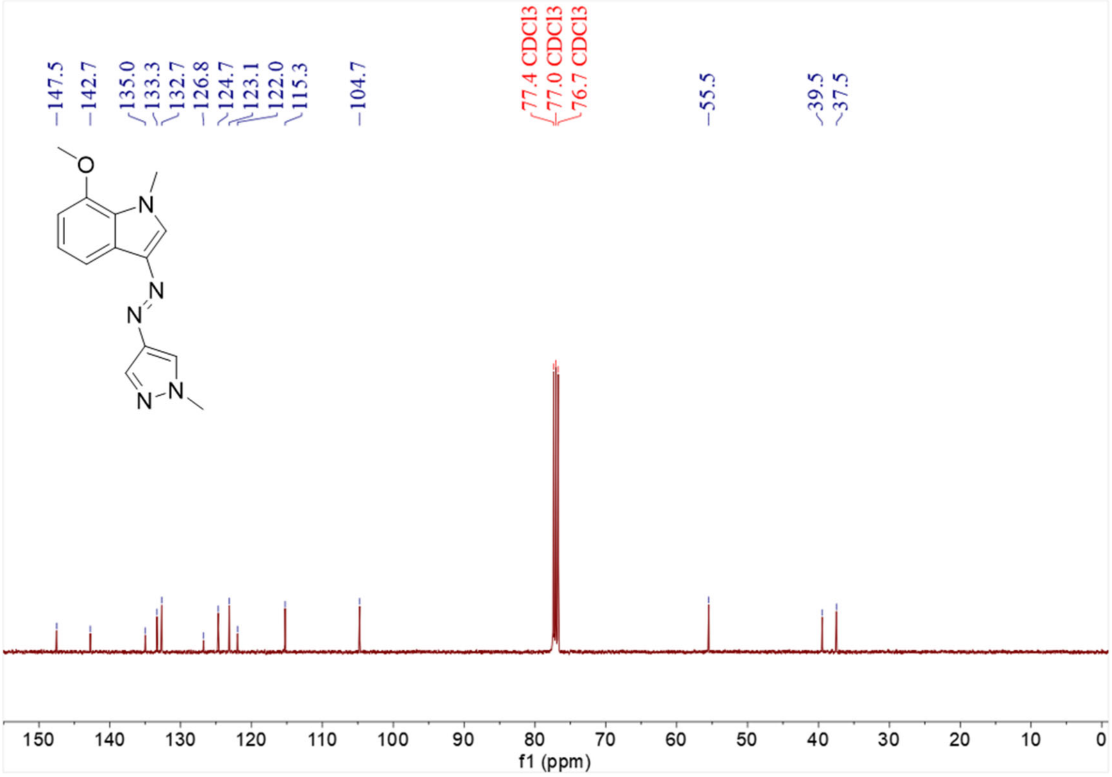

(*E*)-4-fluoro-3-((1-methyl-1*H*-pyrazol-4-yl)diazenyl)-1*H*-indole (10’)

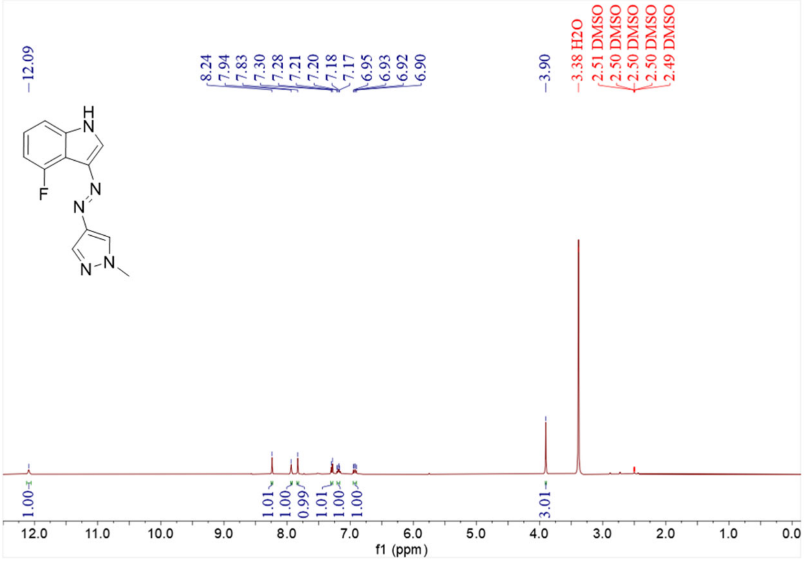

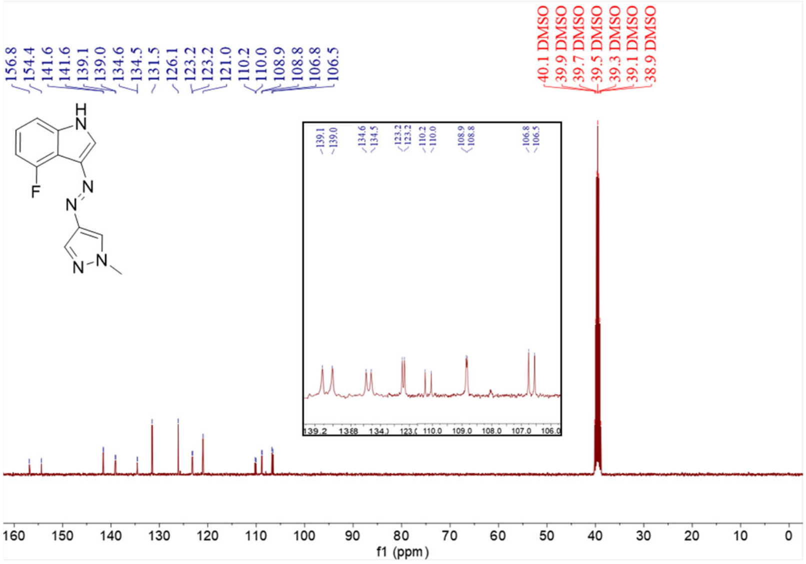

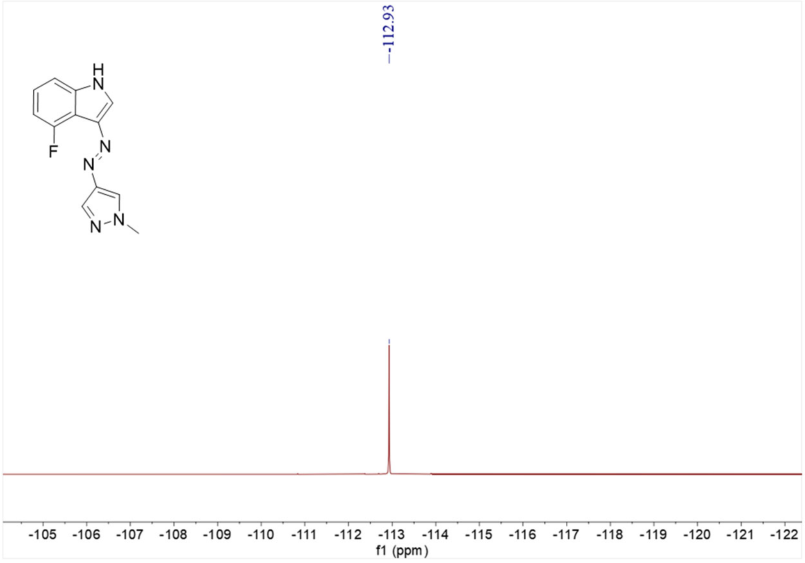

(*E*)-4-fluoro-1-methyl-3-((1-methyl-1*H*-pyrazol-4-yl)diazenyl)-1*H*-indole (10)

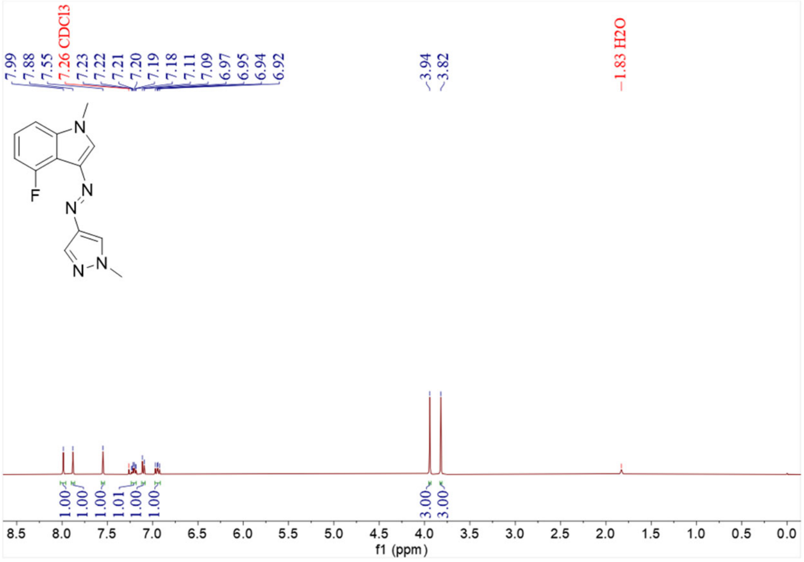

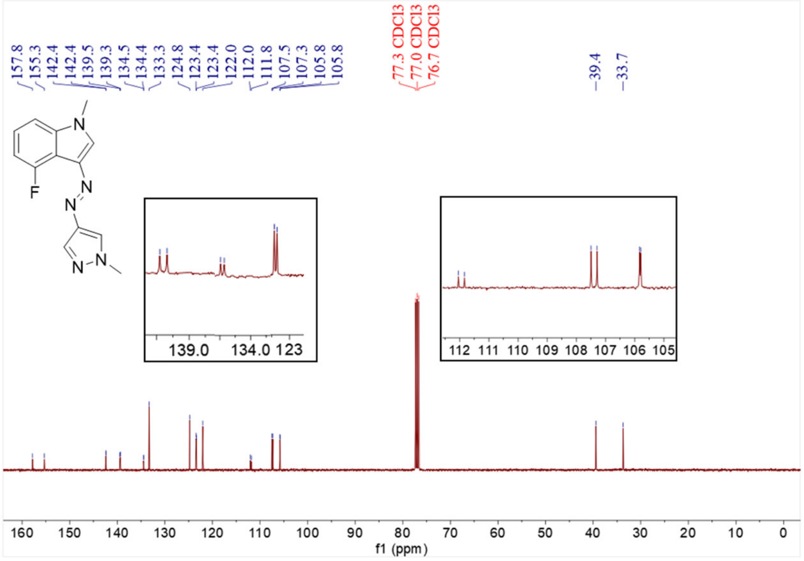

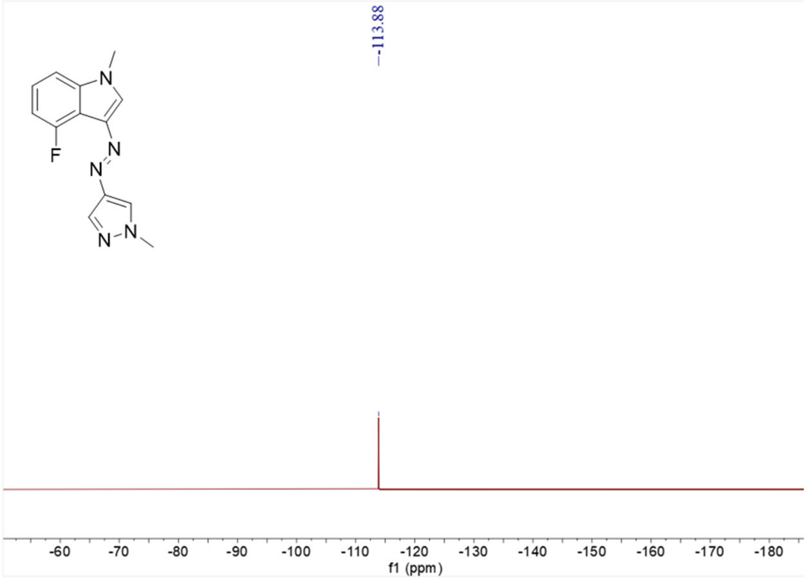

(*E*)-1-methyl-3-((1-methyl-1*H*-pyrazol-4-yl)diazenyl)-4-nitro-1*H*-indole (11)

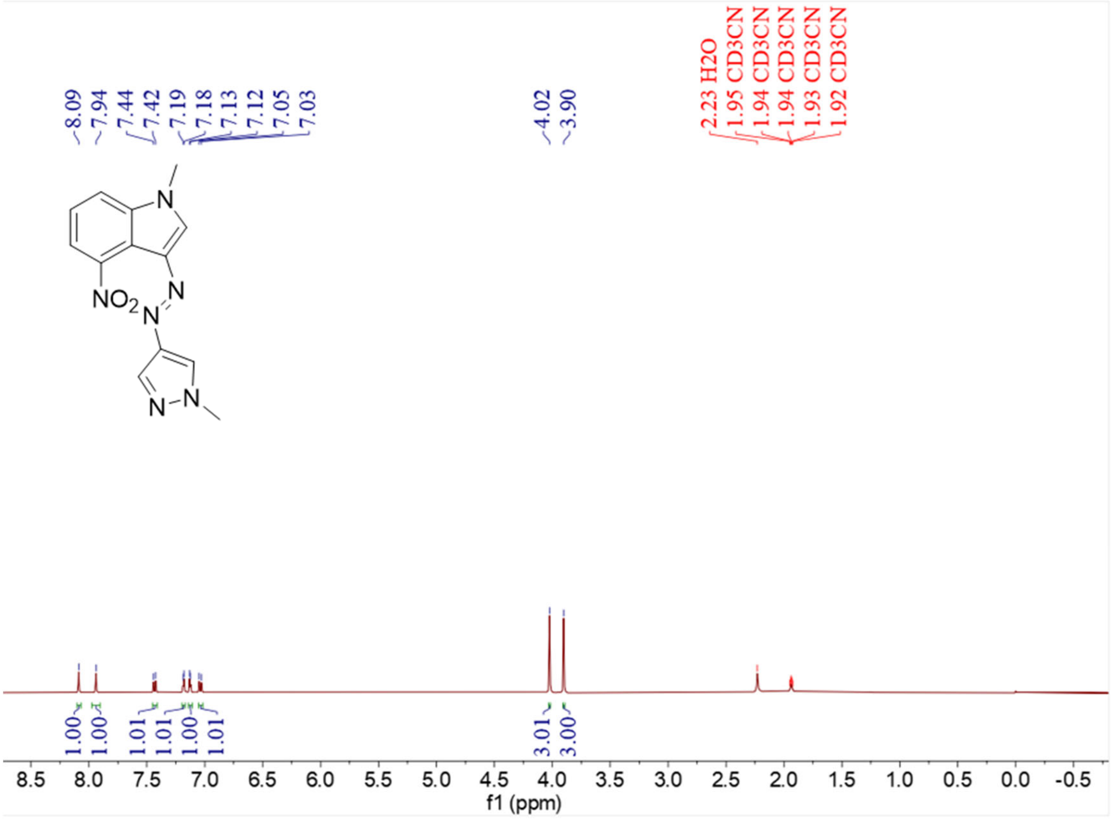

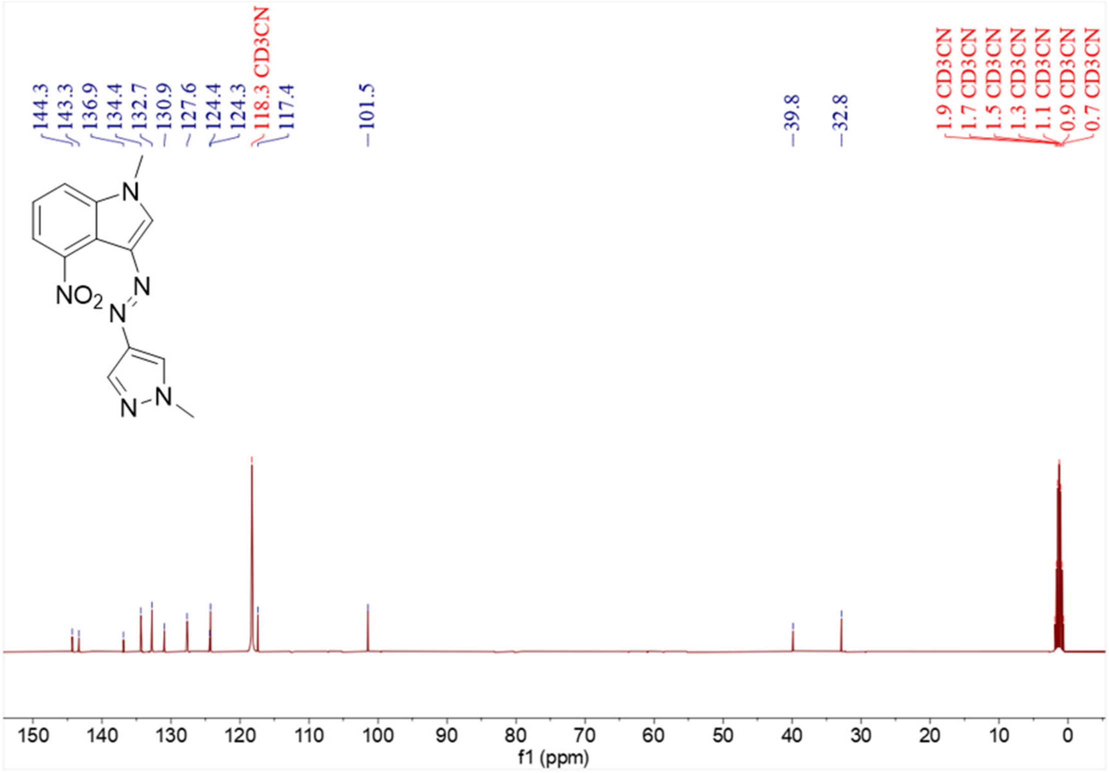

(*E*)-1-(1-methyl-3-((1-methyl-1*H*-pyrazol-4-yl)diazenyl)-1*H*-indol-2-yl)ethan-1-one (12)

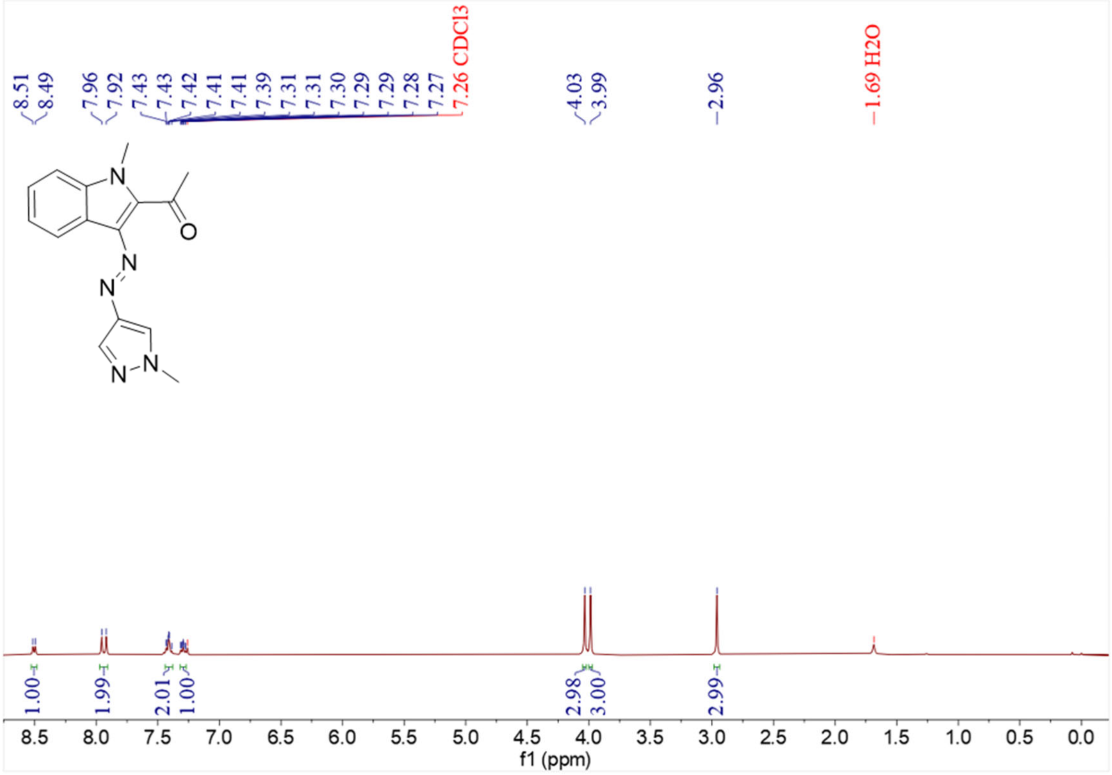

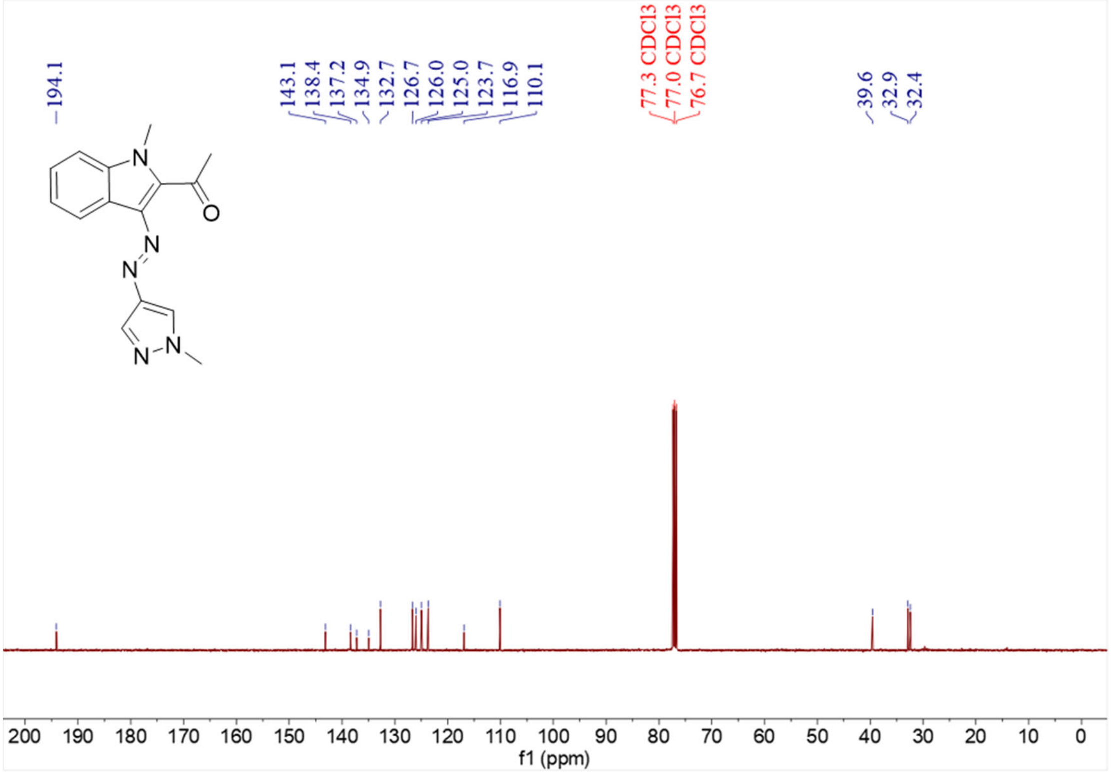

(*E*)-1-(3-((1-methyl-1*H*-pyrazol-4-yl)diazenyl)-1*H*-indol-1-yl)ethan-1-one (16)

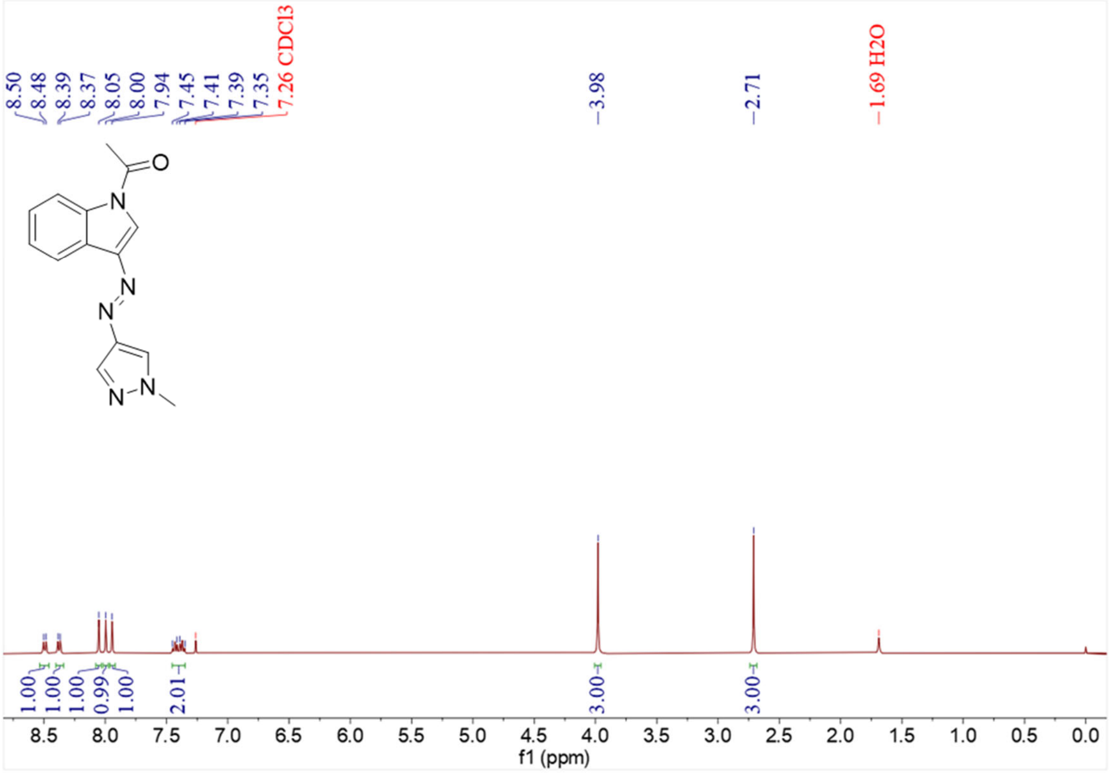

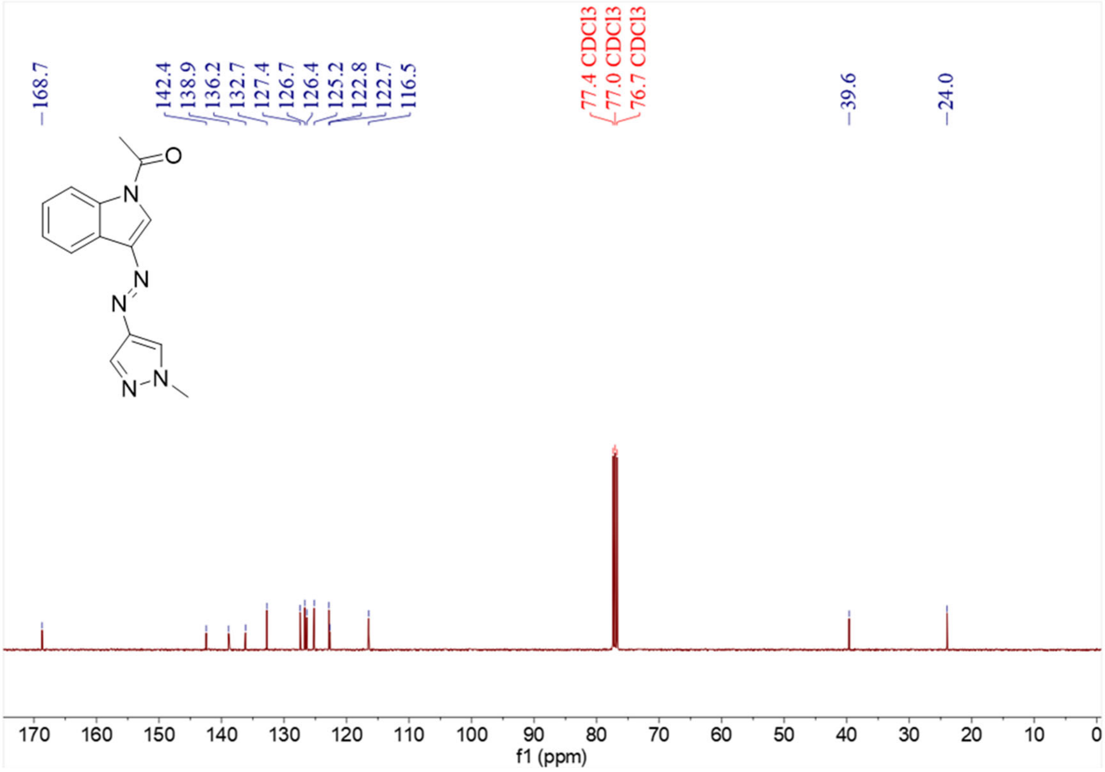

methyl (*E*)-4-((1-(3-bromopropyl)-1*H*-indol-3-yl)diazenyl)-1-methyl-1*H*-pyrazole-5-carboxylate (5-Br)

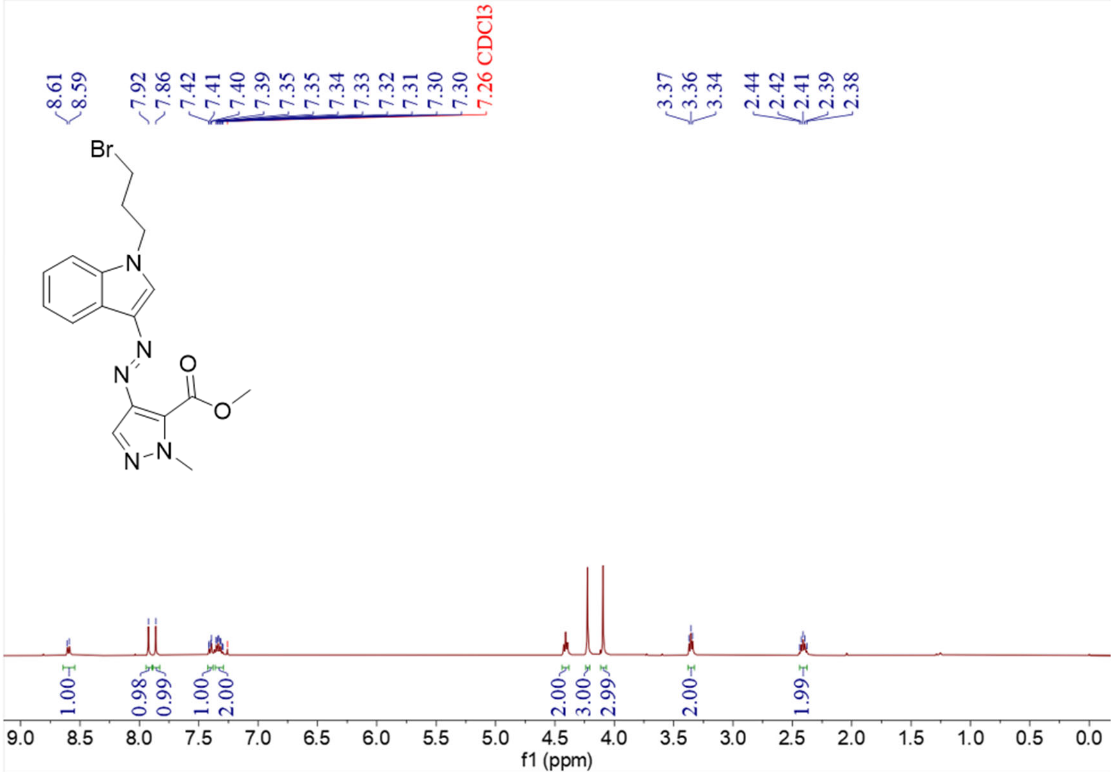

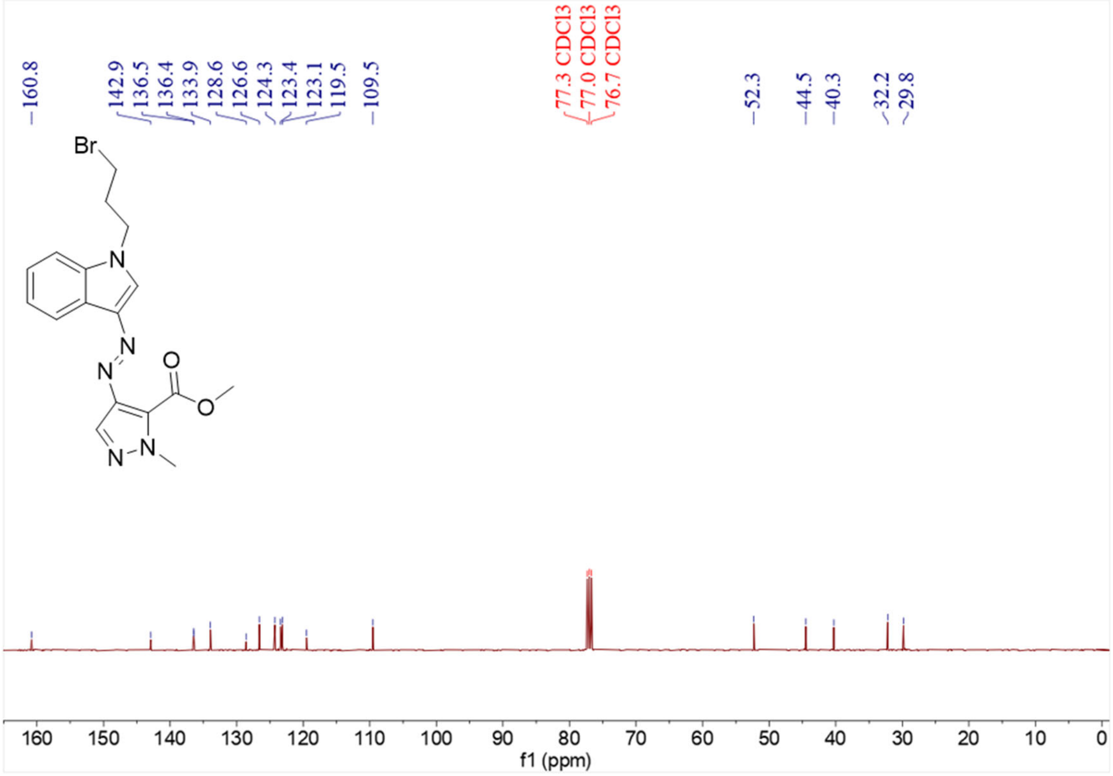

(*E*)-3-(3-((5-(methoxycarbonyl)-1-methyl-1*H*-pyrazol-4-yl)diazenyl)-1*H*-indol-1-yl)-*N*,*N*,*N*-trimethylpropan-1-aminium (5-PS)

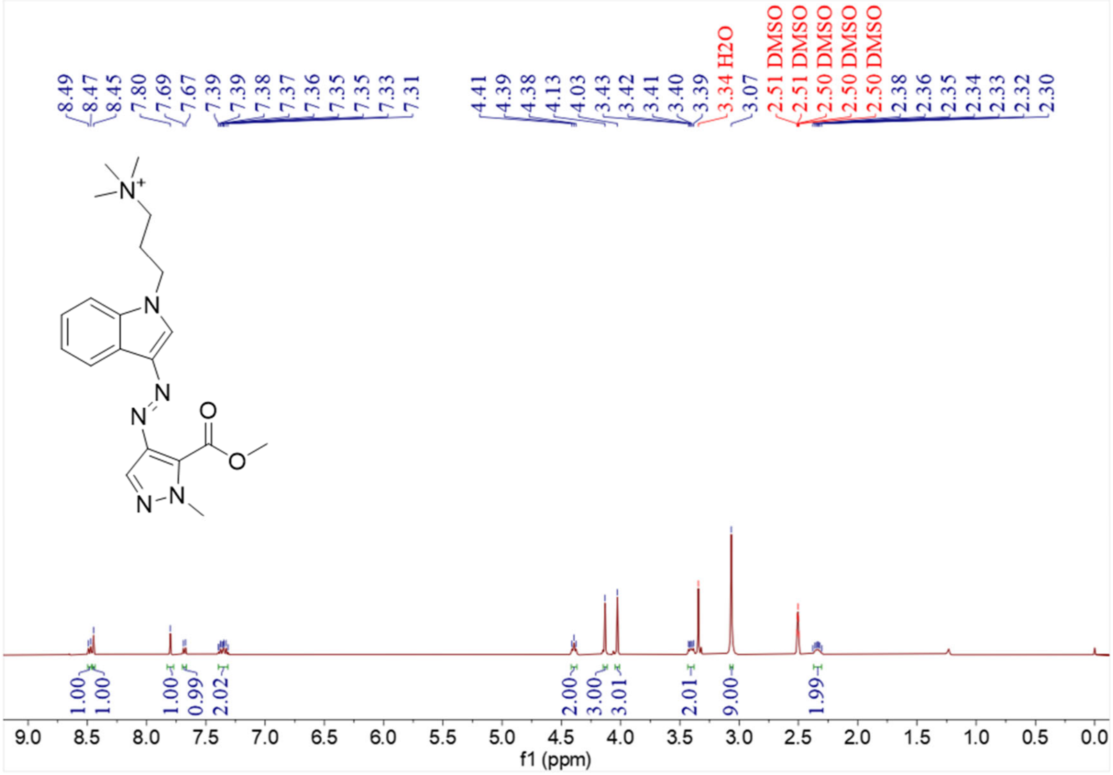

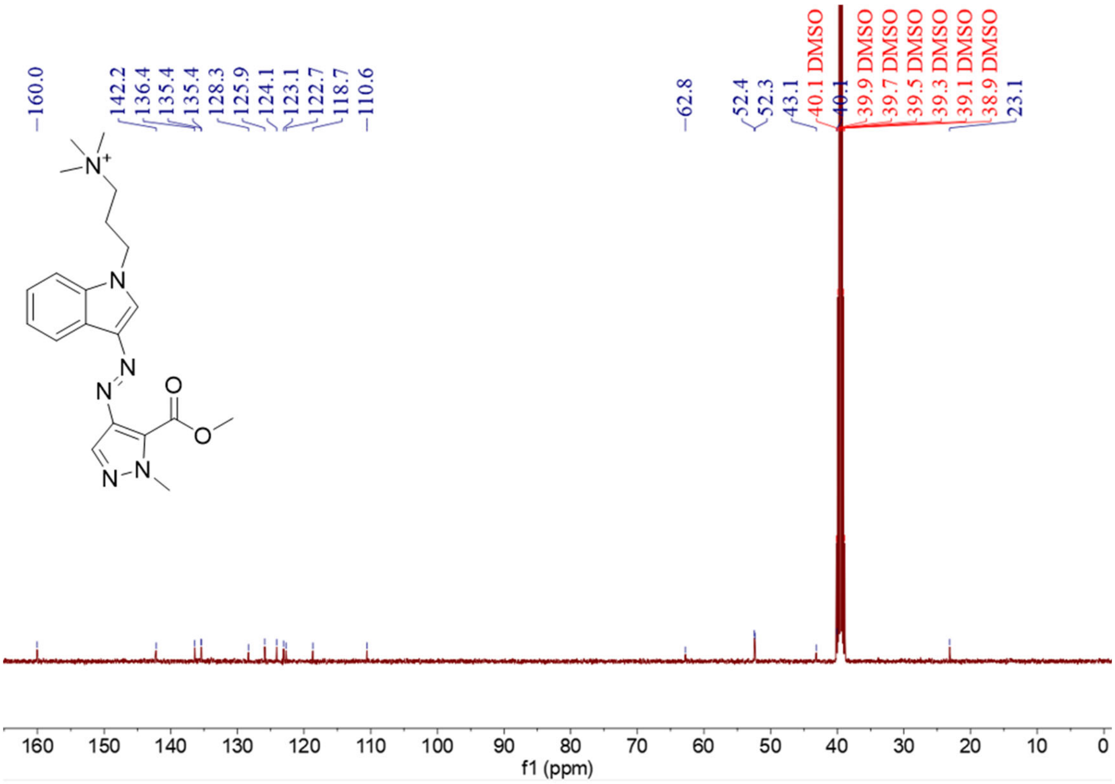

3. Photoswitching performance

All spectral tests were performed with LED light sources of varied power.

Table S1. The wavelength and power of LED light sources.

Wavelength (nm)	350	365	396	400	410
Power (W/m^2)	5.3	3.7	6.0	9.7	6.5
Wavelength (nm)	420	430	440	450	460
Power (W/m^2)	4.0	2.5	38.8	35.6	12.6
Wavelength (nm)	470	495	523	549	585
Power (W/m^2)	33.6	26.5	52.0	28.6	37.0

3.1. UV/Vis spectra

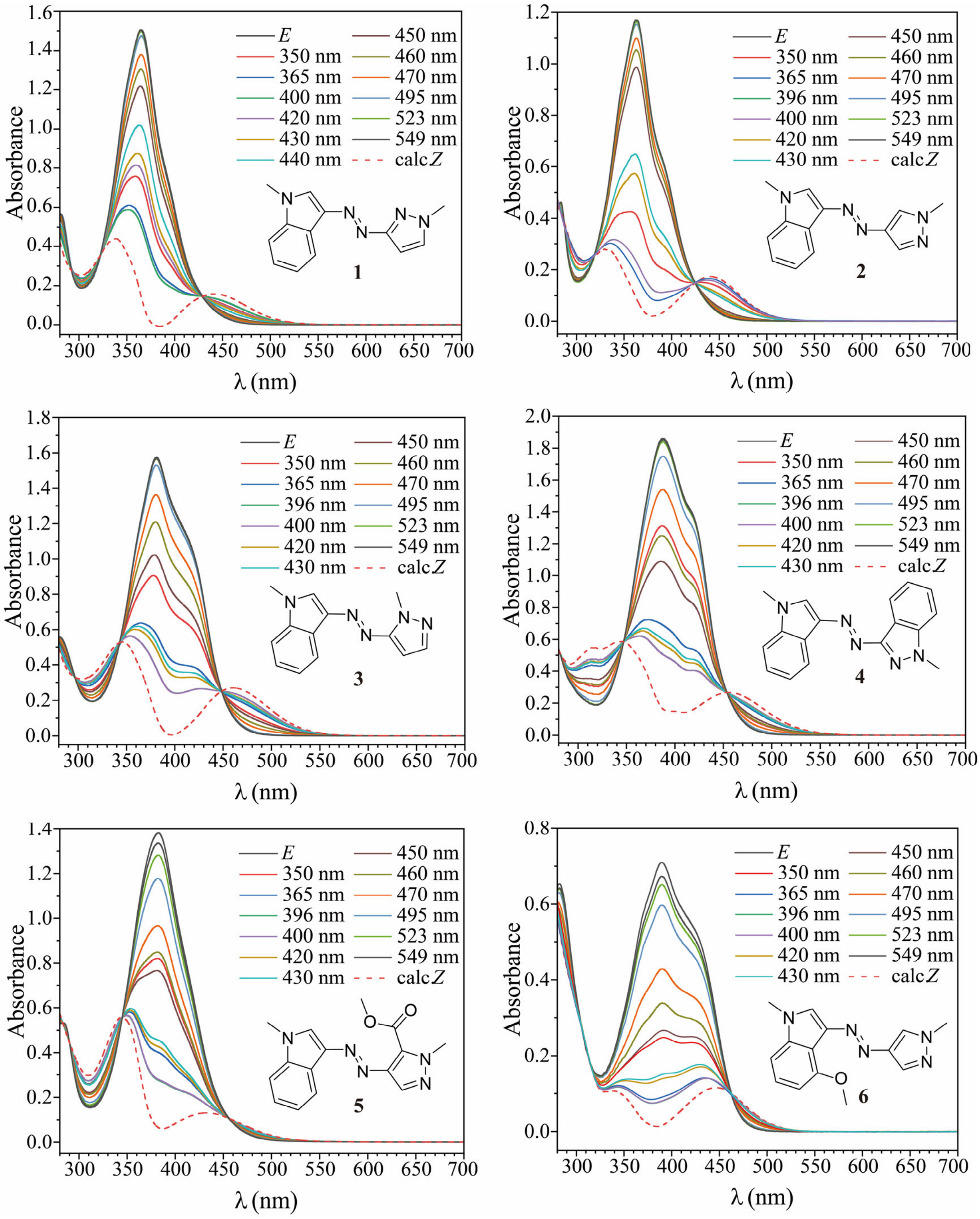

Figure S1. UV-Vis absorption spectra of **1-6** in MeCN at different PSS states.

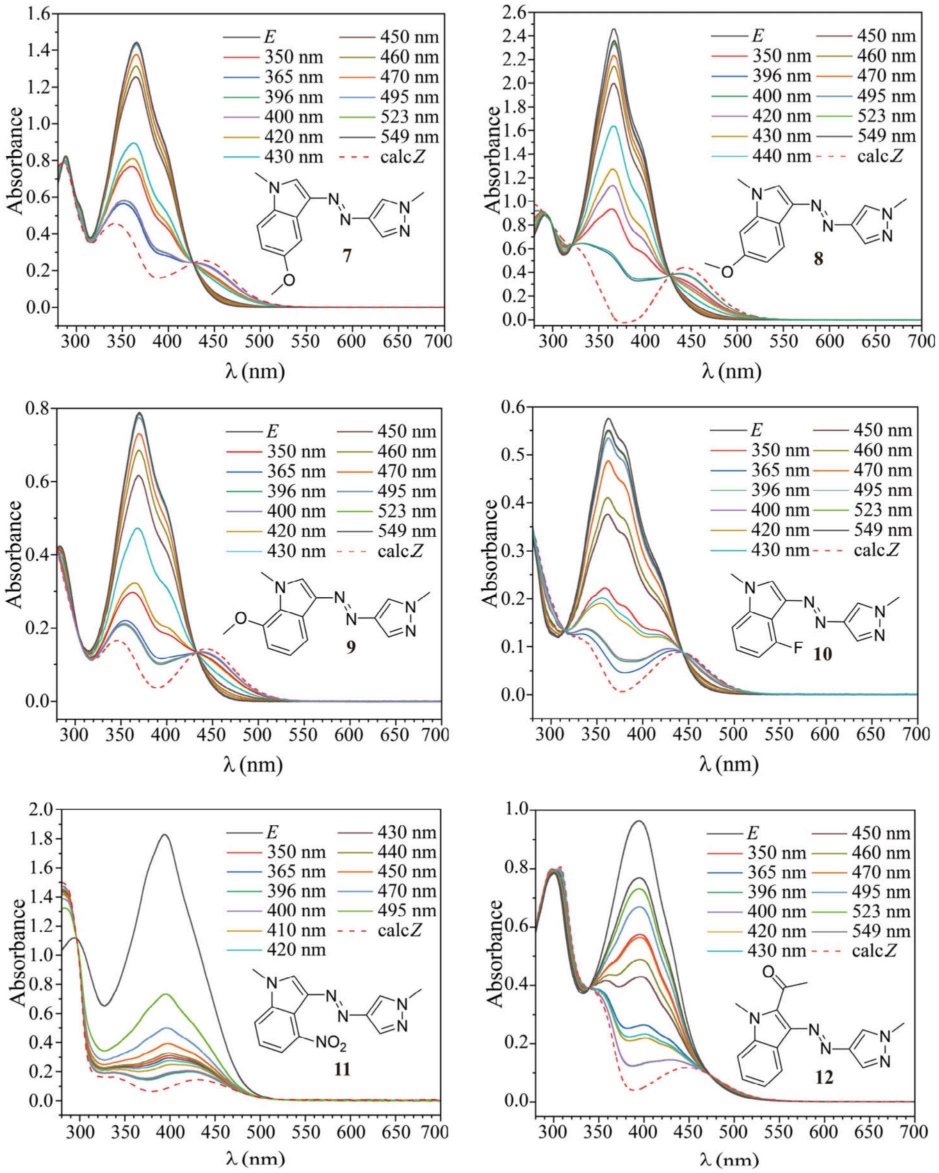

Figure S2 UV-Vis absorption spectra of **7-12** in MeCN at different PSS states.

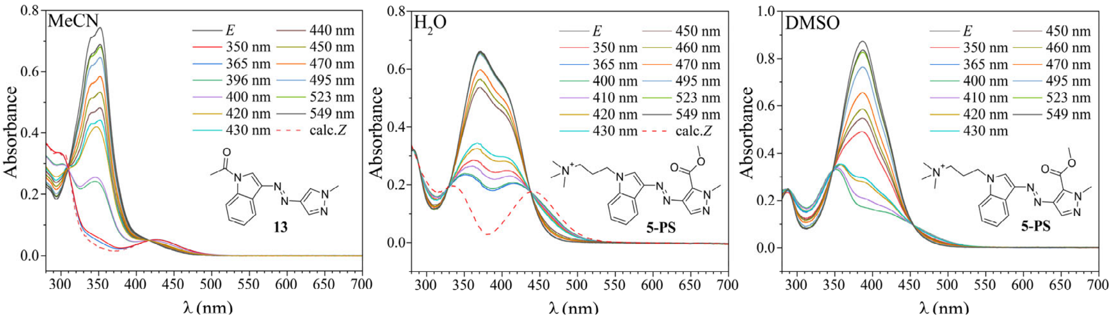

Figure S3 UV-Vis absorption spectra of **13** in MeCN, **5-PS** in H_2O (1‰ Et_3N) and DMSO at different PSS states.

Table S2. A summary of spectroscopic data, *E*⇄*Z* photoisomerization and half-lives of the azoindoles.

	E isomer λ_{max} [a]	*Z* isomer λ_{max} [a]	Photoisomerization [b]		$t_{1/2}$ (h) [c]
	π→π* (nm)	n→π* (nm)	*E*→*Z* (%)	*Z*→*E* (%)	
			400 nm	549 nm	
**1**	365	442	73.5	100.0	15.6
**2**	363	441	85.7	100.0	55.3
**3**	381	460	81.6	100.0	3.2
**4**	387	456	> 65.9 [d]	100.0	0.5
**5**	383	433	86.0	96.2	112.2
**6**	389	447	91.1	98.0	20.5
**7**	365	440	81.3	100.0	14.5
**8**	367	443	78.5	96.2	7.4
**9**	370	444	87.5	99.0	14.9
**10**	362	427	87.1	96.2	106.9
**11**	394	429	94.1	37.0 [e]	204.3
**12**	395	448	90.8	79.4	6.8
**13**	352	426	68.7	88.5 [e]	206.6
**5-PS** [f]	371	440	73.2 [g]	100.0 [g]	6.7 [h] (26.1 [i])

[a] The λ_{max} was taken from UV-Vis absorption spectra measured in MeCN. [b] The isomer compositions were determined by ^{1}H NMR in CD_3CN. [c] The half-lives were measured or calculated by Arrhenius equation in DMSO at 25 °C. [d] The isomer compositions of **4** could be accurately measured due to fast thermal *Z*→*E* isomerization. [e] Irradiation wavelength: 495 nm. [f] The λ_{max} were taken from UV-Vis absorption spectra measured in H_2O with 1.0‰ Et_3N. [g] The isomer compositions were determined by ^{1}H NMR in D_2O. [h] The half-life was measured in H_2O with 1.0‰ Et_3N at 37 °C. [i] The half-life was measured in DMSO at 37 °C.

3.2. Calculation of PSS composition by UV/Vis spectroscopy

First, the composition of specific wavelength PSSs were determined by ^{1}H NMR. Then the pure *Z*-isomer UV/Vis absorption spectral were deduced by those of PSSs and pure *E* isomer by spectral fitting. The PSS composition at the other PSSs were calculated according to the spectra of pure *E* and *Z* isomers. Spectral fitting method was performed according to our previous work.[10]

$$\alpha \times \mathrm{Abs.}_E(\lambda) + (1-\alpha) \times \mathrm{Abs.}_Z(\lambda) = \mathrm{Abs.}_{PSS}(\lambda)$$

where α is the fraction of *E*-isomer in a PSS, $\mathrm{Abs.}_E(\lambda)$ is the spectra of pure *E*-isomer, $\mathrm{Abs.}_Z(\lambda)$ is the spectra of pure *Z*-isomer and $\mathrm{Abs.}_{PSS}$ is the spectra of the PSS (at the same concentration).

Table S3. Calculated fractions of *Z* isomers of azoindoles at different PSSs in MeCN or H_2O.

Fractions (%) [a]	350/365 nm	396/400 nm	410/420 nm	430/440 nm	450 nm	460 nm	470 nm	495 nm	523 nm	549 nm
**1**	57.1/71.0	–/73.5	–/52.2	47.2/36.0	21.1	14.5	9.0	2.2	0.3	0.0
**2**	70.2/91.6	86.7/85.7	–/55.5	48.3/–	16.8	15.0	6.3	1.3	0.4	0.0
**3**	46.3/68.3	81.5/81.6	–/74.2	71.5/–	38.0	25.1	14.6	3.0	0.6	0.0
**4**	–	–	–	–	–	–	–	–	–	–
**5**	42.6/74.4	87.2/86.0	–/72.5	70.3/–	46.7	40.5	31.5	15.3	7.5	3.8
**6**	66.2/89.2	90.8/91.1	–/82.0	80.5/–	63.5	53.5	40.4	16.3	8.5	2.0
**7**	61.4/83.	81.7/81.3	–/57.5	49.5/–	16.7	11.4	5.8	0.9	0.2	0.0
**8**	62.7/–	79.1/78.5	–/54.3	48.6/34.0	18.9	12.9	9.3	5.2	4.7	3.8
**9**	71.5/85.7	88.1/87.5	–/67.8	45.6/–	24.8	14.8	8.6	2.1	1.3	1.0
**10**	65.6/92.5	87.9/87.1	–/71.7	69.3/–	36.7	30.2	16.3	7.4	4.9	3.8
**11**	87.2/94.7	94.8/94.1	90.8/89.2	88.1/86.2	82.4	–	76.2	63.0	–	–
**12**	42.3/76.4	91.0/90.8	–/81.4	79.8/–	58.2	51.8	43.5	32.1	25.4	20.6
**13**	95.7/97.3	70.6/65.4	–/45.4	45.9/36.4	29.3	–	22.3	11.5	9.7	8.7
**5-PS** [b]	62.8/74.2	–/73.2	67.2/55.6	52.2/–	20.5	45.7	10.3	1.5	0.6	0.0

[a] The isomer compositions were calculated in MeCN. [b] The isomer compositions were calculated in H_2O with 1‰ Et_3N.

3.3. Qualification of PSS composition by NMR in CD_3CN or D_2O.

E

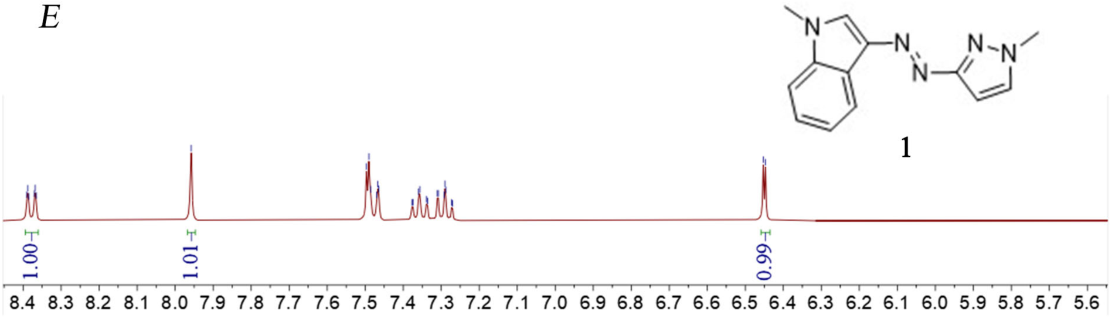

400 nm PSS $Z\% = 2.78/(1.00+2.78) = 73.54\%$

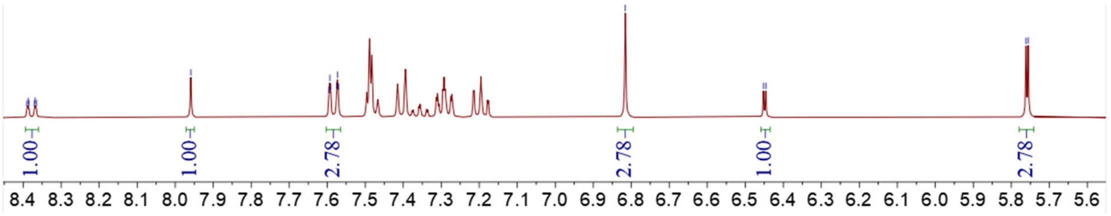

549 nm PSS $E\% = 1.00/(1.00+0.00) = 100.00\%$

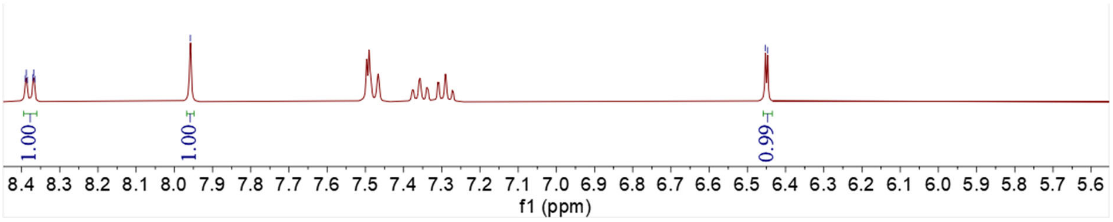

Figure S4. ^{1}H NMR spectra of *E*-**1** and its PSSs in CD_3CN.

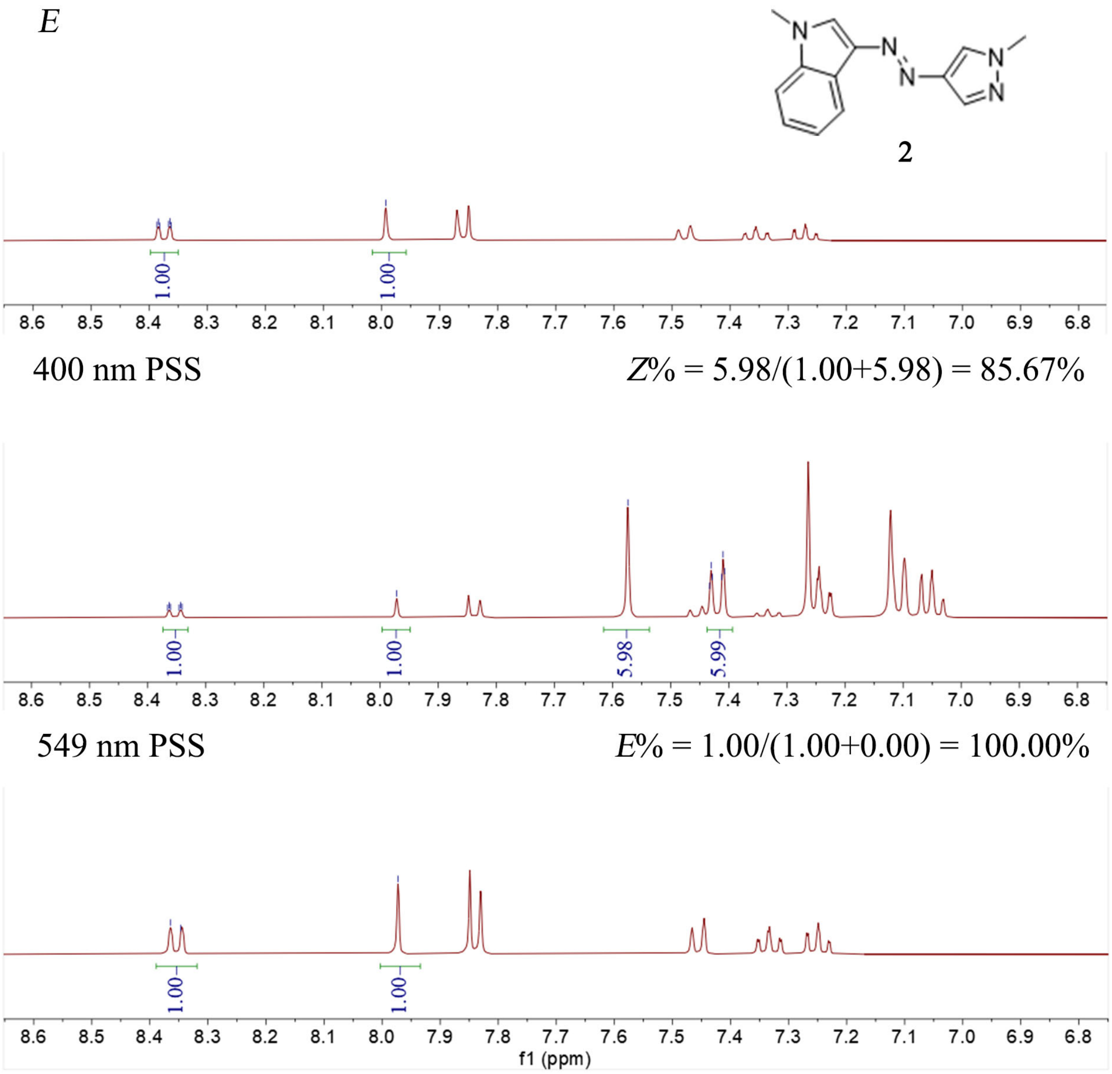

Figure S5. ^{1}H NMR spectra of *E*-**2** and its PSSs in CD_3CN.

E

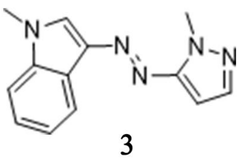

3

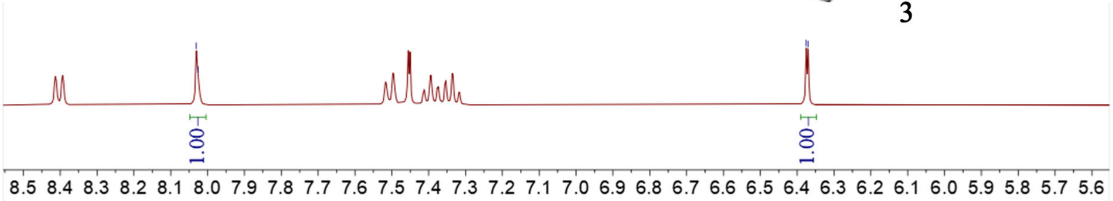

400 nm PSS $Z\% = 4.44/(1.00+4.44) = 81.62\%$

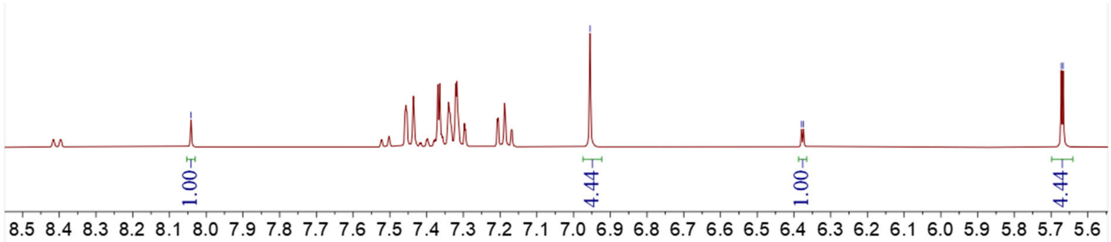

549 nm PSS $E\% = 1.00/(1.00+0.00) = 100.00\%$

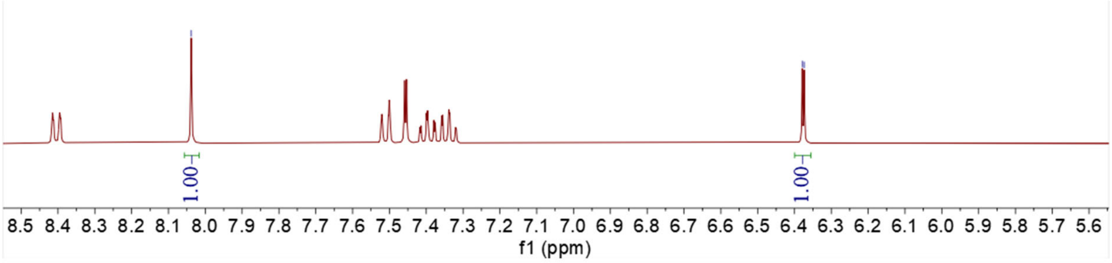

Figure S6. ^1H NMR spectra of *E*-**3** and its PSSs in CD_3CN.

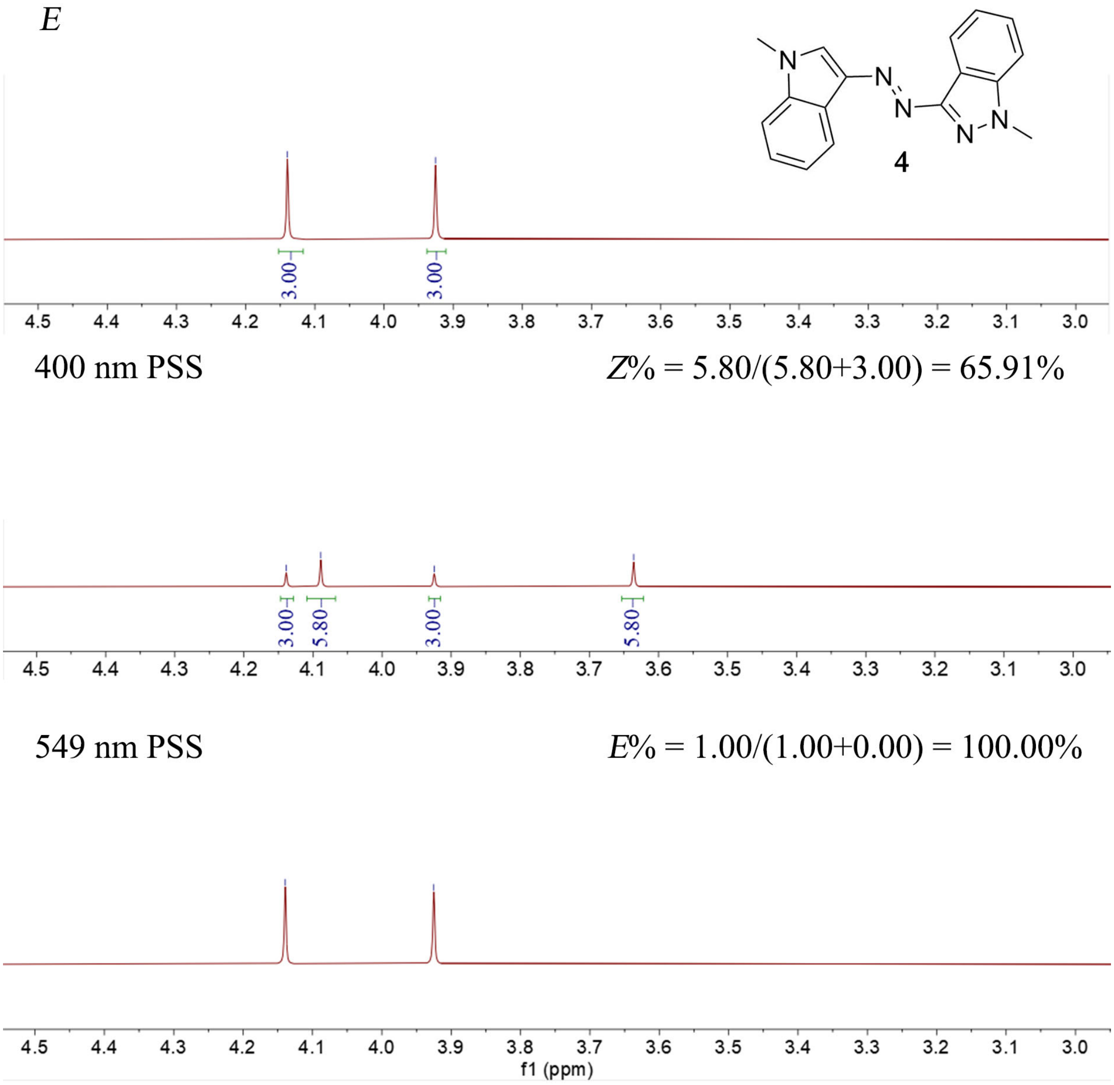

Figure S7. ^{1}H NMR spectra of *E*-**4** and its PSSs in CD_3CN.

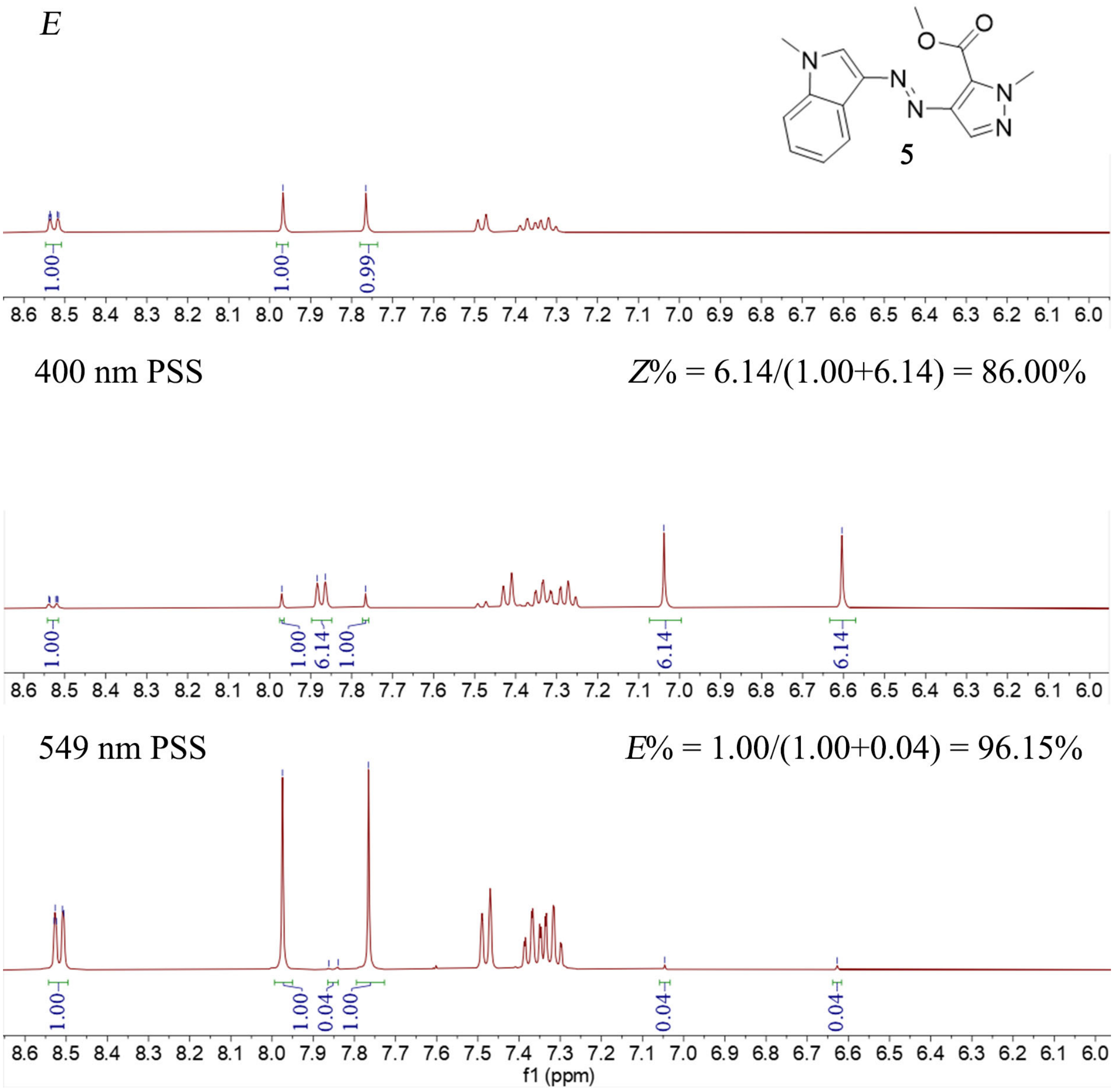

Figure S8. ^{1}H NMR spectra of *E*-**5** and its PSSs in CD_3CN.

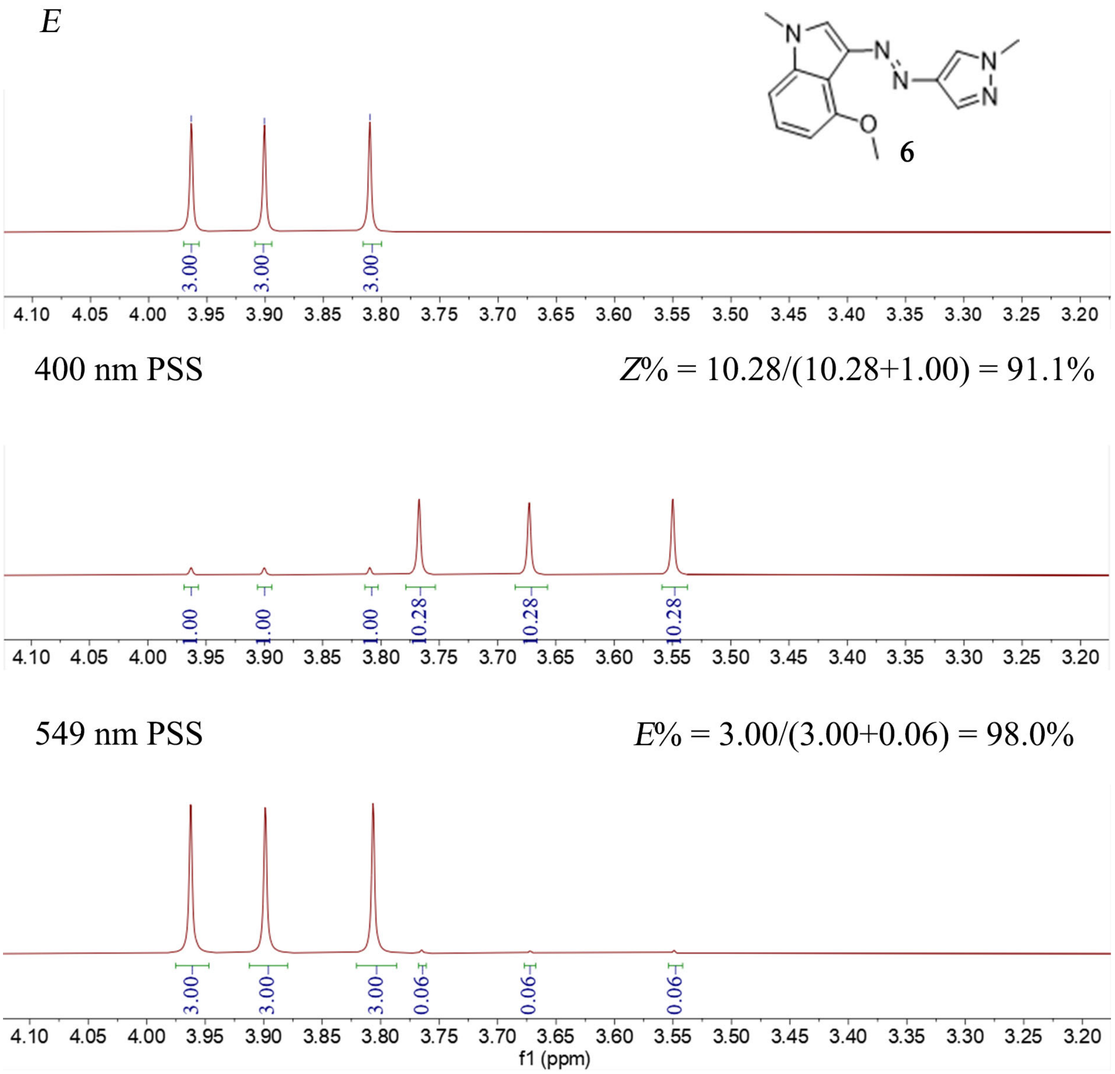

Figure S9. ^{1}H NMR spectra of *E*-**6** and its PSSs in CD_3CN.

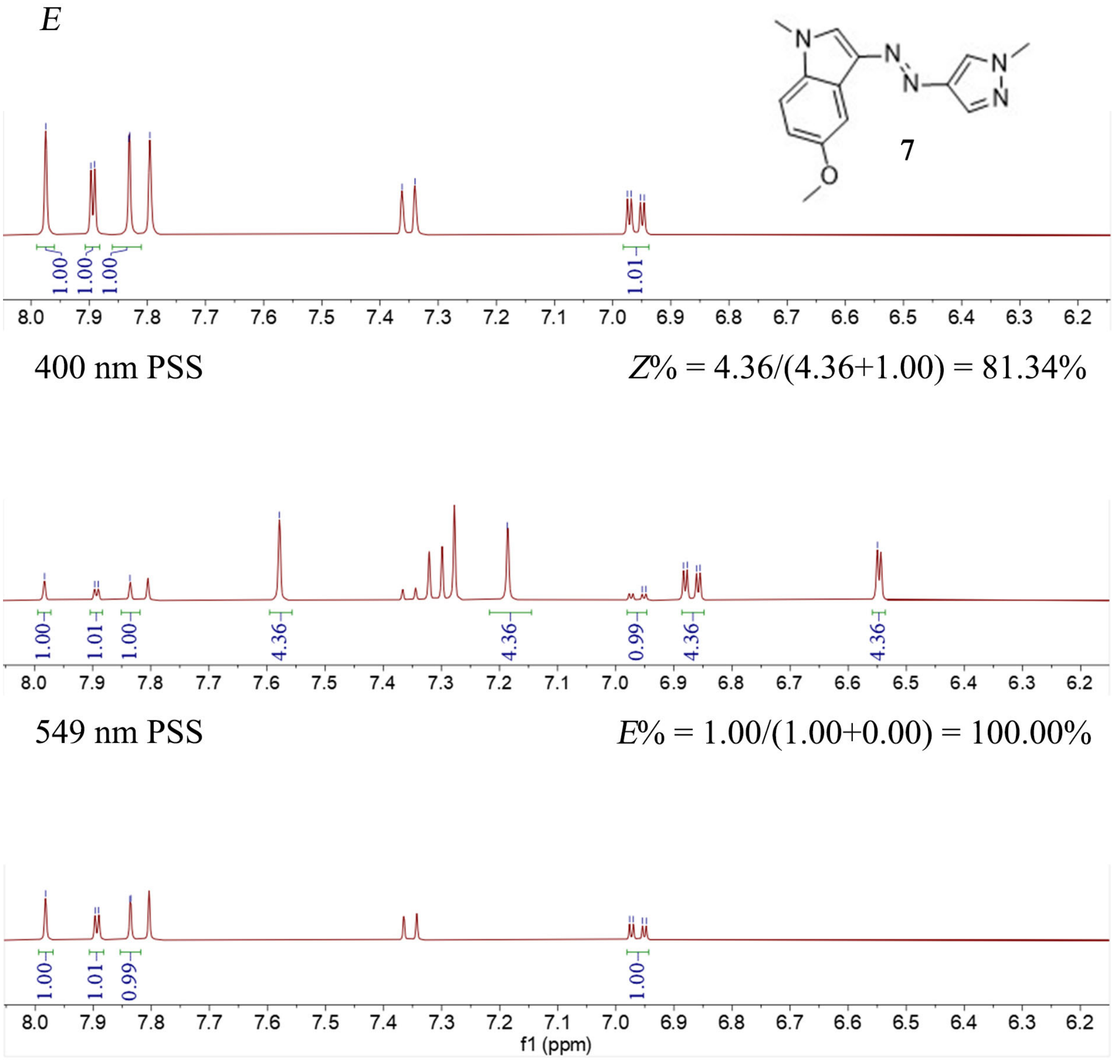

Figure S10. ^{1}H NMR spectra of *E*-**7** and its PSSs in CD_3CN.

E

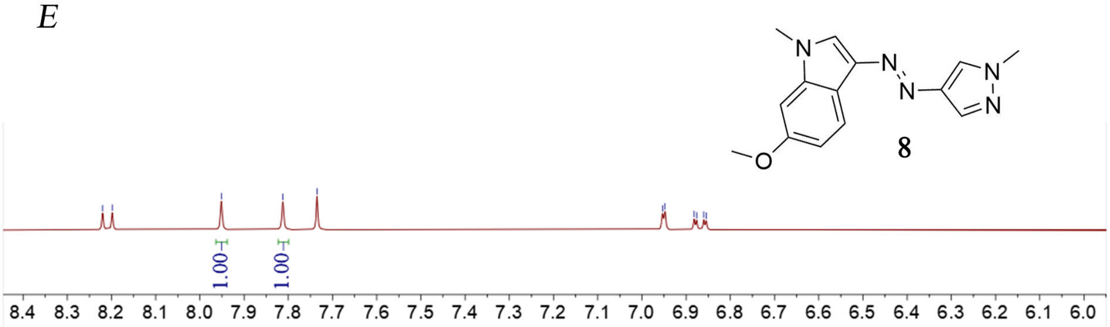

400 nm PSS $Z\% = 3.66/(3.66+1.00) = 78.54\%$

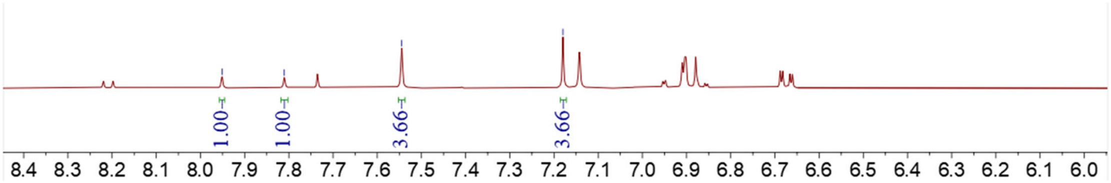

549 nm PSS $E\% = 1.00/(1.00+0.04) = 96.15\%$

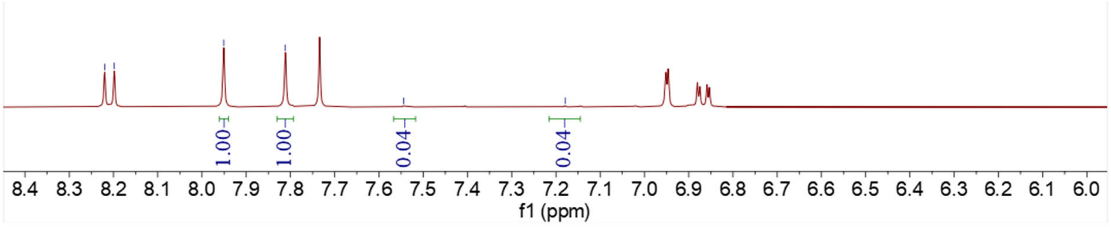

Figure S11. ^{1}H NMR spectra of *E*-**8** and its PSSs in CD_3CN.

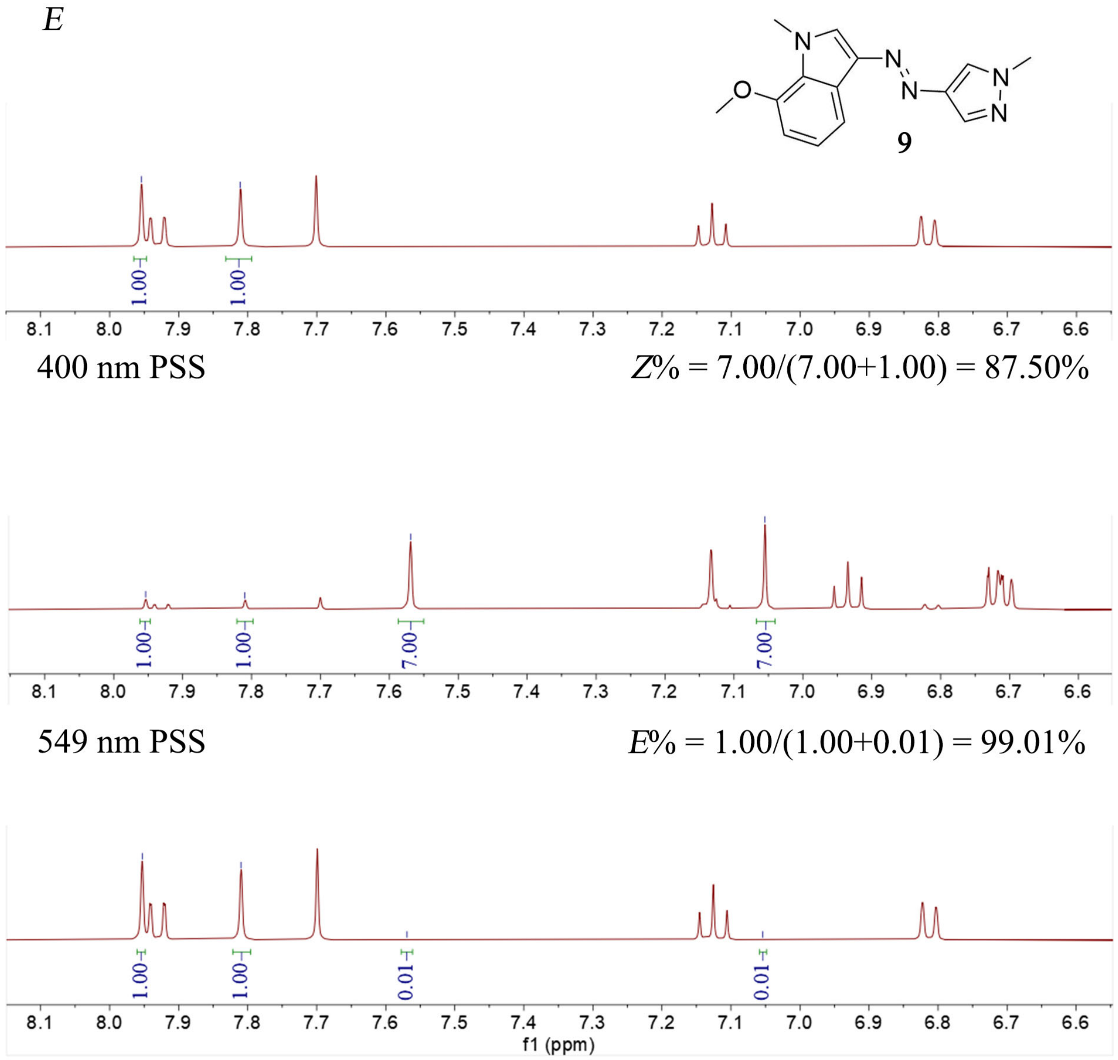

Figure S12. ^{1}H NMR spectra of *E*-**9** and its PSSs in CD_3CN.

E

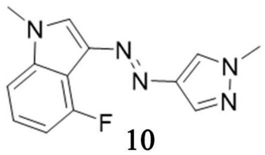

10

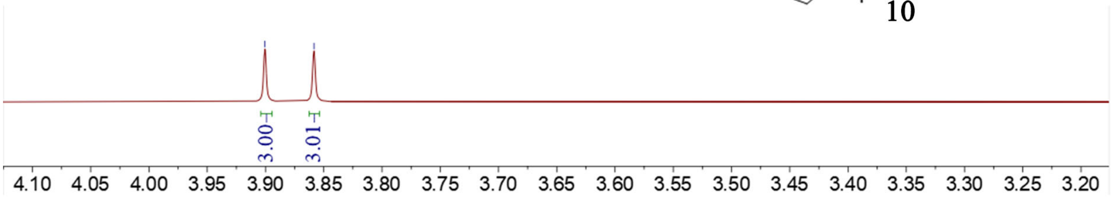

400 nm PSS $Z\% = 20.18/(20.18+3.00) = 87.06\%$

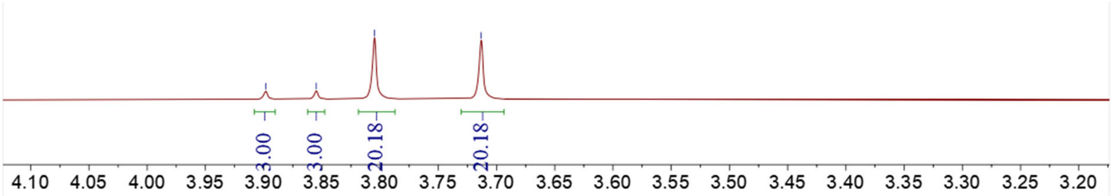

549 nm PSS $E\% = 3.00/(3.00+0.12) = 96.15\%$

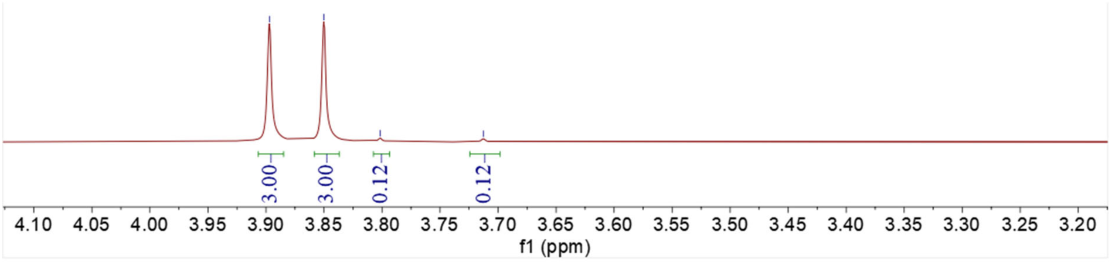

Figure S13. ^{1}H NMR spectra of *E*-**10** and its PSSs in CD_3CN.

E

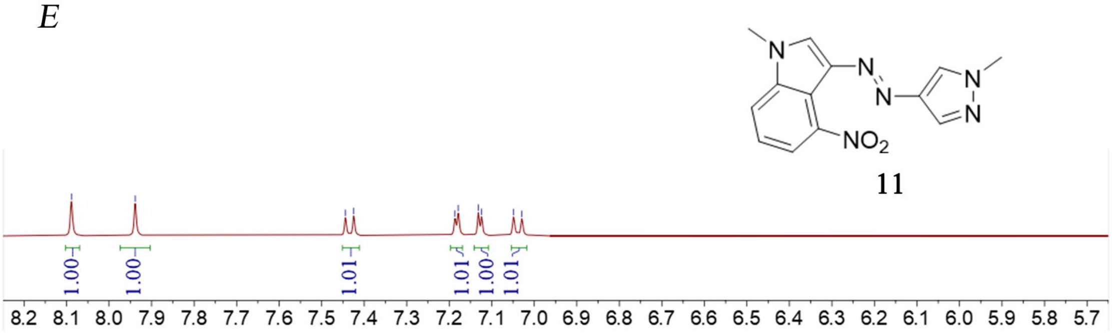

400 nm PSS $Z\% = 15.99/(15.99+1.00) = 94.11\%$

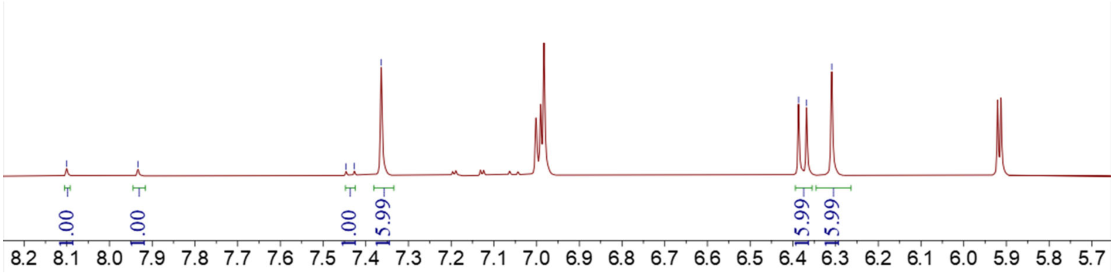

495 nm PSS $E\% = 1.00/(1.00+1.70) = 37.04\%$

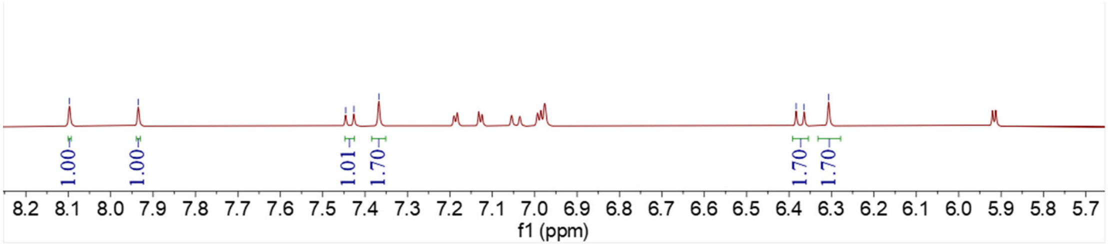

Figure S14. ^1H NMR spectra of *E*-**11** and its PSSs in CD_3CN.

E

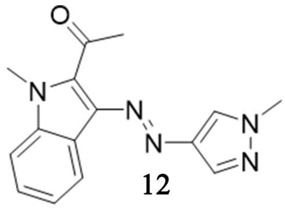

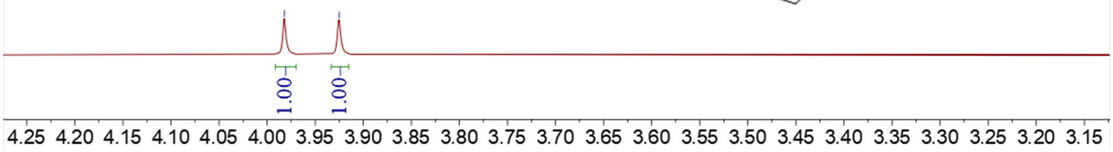

400 nm PSS $Z\% = 9.87/(9.87+1.00) = 90.80\%$

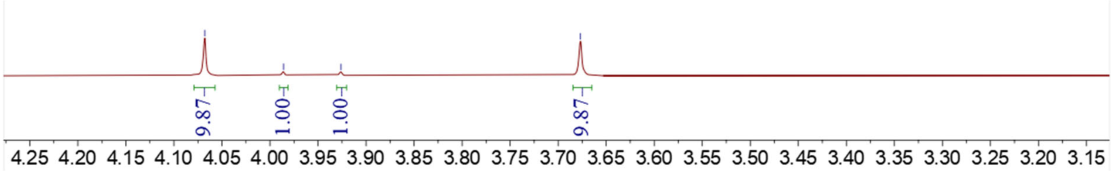

549 nm PSS $E\% = 1.00/(1.00+0.26) = 79.37\%$

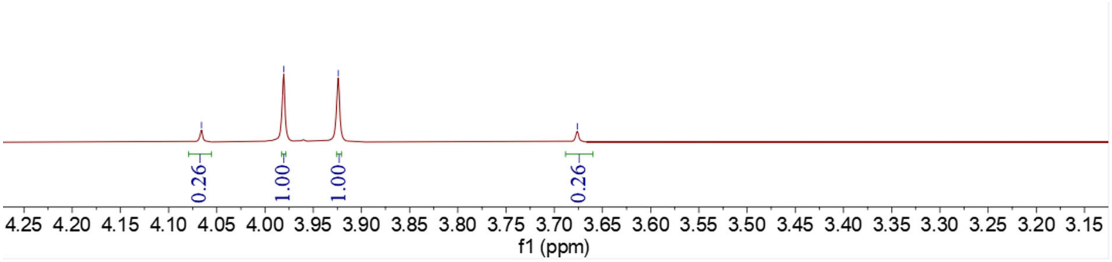

Figure S15. ^{1}H NMR spectra of *E*-**12** and its PSSs in CD_3CN.

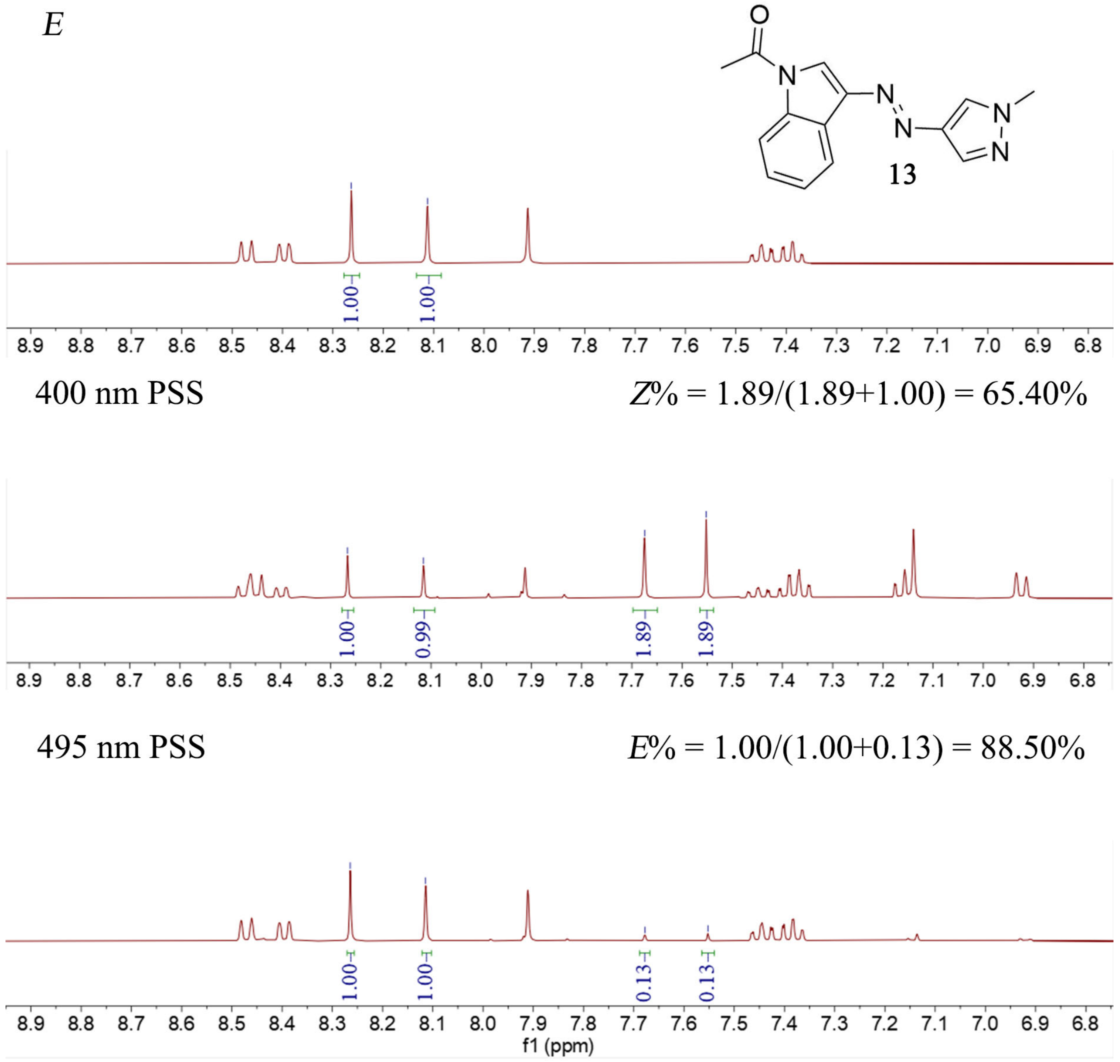

Figure S16. ^{1}H NMR spectra of *E*-**13**and its PSSs in CD_3CN.

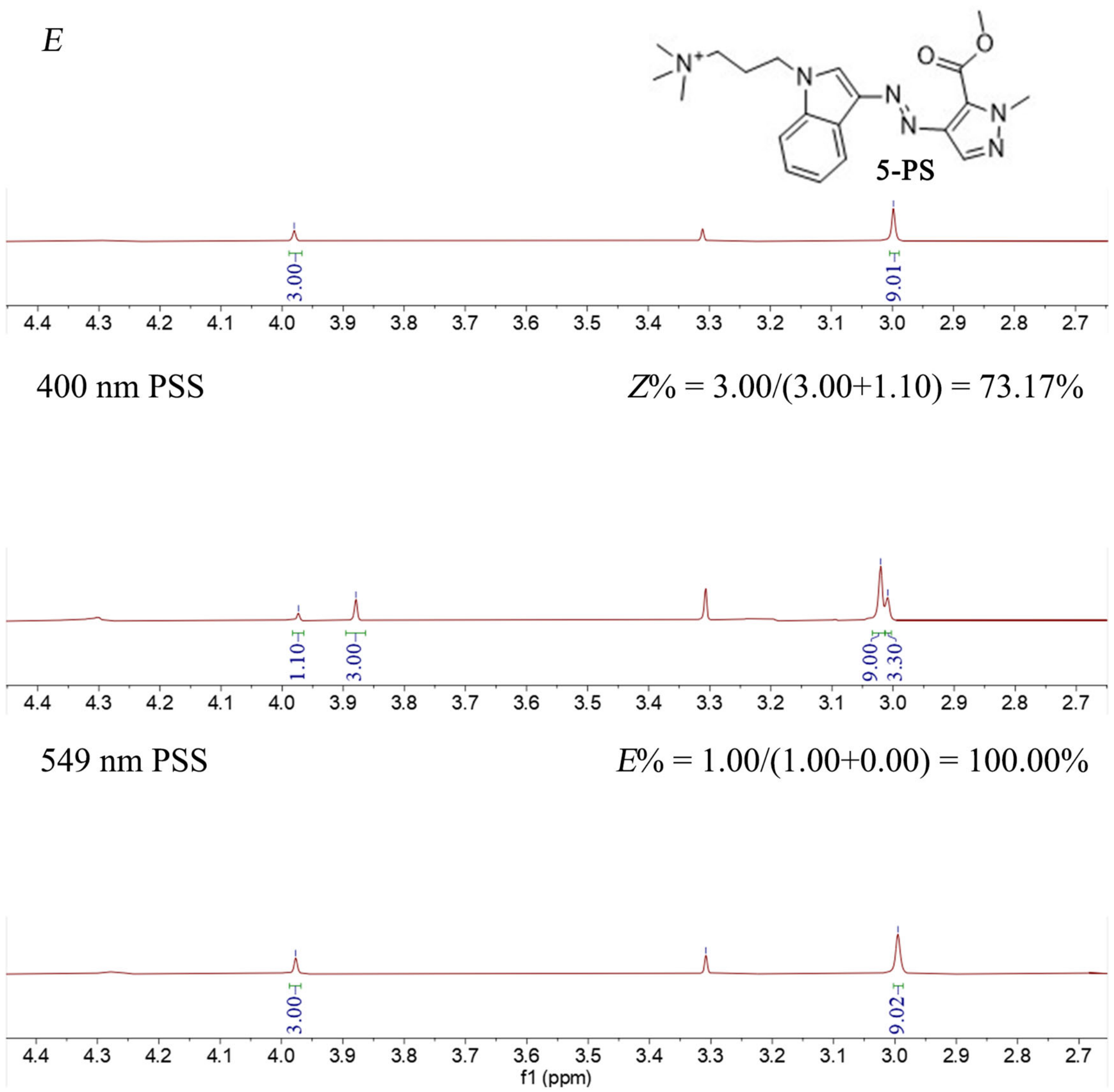

Figure S17. ^{1}H NMR spectra of *E*-**5-PS** and its PSSs in D_2O.

3.4. Quantum yields of photoisomerization in acetonitrile

The differential rate equation of $E \to Z$ photoisomerization is as following:[10b]

$$\frac{dc_t}{dt} = k(c_0 - c_t) - k_{-1}c_t$$

$$k_1 = \frac{I \cdot l \cdot (1-10^{-A(t)}) \cdot \Phi_{E\to Z} \cdot \varepsilon_E}{V \cdot A(t)} \qquad k_{-1} = \frac{I \cdot l \cdot (1-10^{-A(t)}) \cdot \Phi_{Z\to E} \cdot \varepsilon_Z}{V \cdot A(t)}$$

k_1 and k_{-1} are the rate constants at the excitation wavelength of $E \to Z$ and $Z \to E$ isomerization, respectively; $c_0 = [E]_0$ is the initial concentration of E-isomer, $c_t = [Z]_t$ is the concentration of Z-isomer at time t; I is the photon flux of the excitation light; l is the beam path length through quartz cuvette; A(t) is the absorbance with time t at the excitation wavelength; $\Phi_{E\to Z}$ and $\Phi_{Z\to E}$ are quantum yields at the excitation wavelength of $E \to Z$ and $Z \to E$ isomerization, respectively; ε_E and ε_Z are the absorption coefficients at the excitation wavelength of the E and Z isomer, respectively; V is the volume of irradiated solution.

At the PSS, the concentrations of both isomers do not change any more, i.e. $\frac{dc_t}{dt} = 0$.

Thus, the equation $\frac{\Phi_{E\to Z}}{\Phi_{Z\to E}} = \frac{c_\infty \cdot \varepsilon_Z}{(c_0 - c_\infty) \cdot \varepsilon_E}$ is obtained, $c_\infty = [Z]_\infty$ is the concentration of Z-isomer at PSS, Defining the fraction of Z-isomer as $y = c_t / c_0$ (calculated from UV-Vis spectra), the equations depicted above are combined as $\ln\frac{y_\infty - y}{y_\infty - y_0} = -\frac{I \cdot l \cdot \Phi_{E\to Z} \cdot \varepsilon_E}{V \cdot y_\infty}\int_{t0}^{t}\frac{1-10^{-A(t)}}{A(t)}dt$ ($y_\infty = c_\infty / c_0$ is the fraction of Z-isomer at the PSS). Let the parameter $B = \frac{I \cdot l \cdot \Phi_{E\to Z} \cdot \varepsilon_E}{V \cdot y_\infty}$ and integrated photokinetic factor $x(t) = \int_{t0}^{t}\frac{1-10^{-A(t)}}{A(t)}dt$, then $y = (y_0 - y_\infty) \cdot e^{-B\cdot x(t)} + y_\infty$, Plotting y against $x(t)$, followed by exponential fitting, the B and y_∞ are obtained, The quantum yield is allowed to be calculated as $\Phi_{E\to Z} = \frac{B \cdot V \cdot y_\infty}{I \cdot l \cdot \varepsilon_E}$, Similarly, the quantum yield of $Z \to E$ photoisomerization can be calculated by exchanging "E" and "Z" in the equations shown above.

Determination of photon flux

The photon flux of excitation light sources was determined by chemical actinometry using potassium ferrioxalate as standard actinometer.[11] All the processes were performed in a dark environment or under dark red light. 2.5 mL (V_1) of ferrioxalate solution (0.03 M $K_3[Fe(C_2O_4)_3]\cdot 3H_2O$ in 0.1 M H_2SO_4 for 395 nm light source, 0.15 M $K_3[Fe(C_2O_4)_3]\cdot 3H_2O$ in 0.1 M H_2SO_4 for 500 nm light source) was irradiated at the particular wavelength under stirring for a time period (depending on the light sources), providing less than 10% conversion of Fe(III) → Fe(II). The absorbance of the sample at the excitation wavelength

($A(\lambda)$) was recorded before and after irradiation, and there should be no significant change. 0.5 mL (V_2) of the irradiated sample together with 1 mL of buffered solution (1.2 M NaOAc in 0.36 M H_2SO_4) and 2 mL of ligand solution (0.06 M 1,10-phenanthroline in H_2O) were diluted to 25 mL (V_3) with water. After stirred for about 2 h, 2.5 mL of the solution was taken to measure the absorbance at 510 nm ($A_{510\ nm}$). This procedure was repeated using different irradiation time to create a plot of the $A_{510\ nm}$ with irradiation time, and the slope was used to calculate the photon flux (I) through the following equation: $I(\lambda) = slope \cdot \frac{V_1 \cdot V_3}{V_2 \cdot \varepsilon_{510nm} \cdot l \cdot \Phi_\lambda \cdot (1-10^{-A(\lambda)})}$ (ε_{510nm} =11100 M^{-1} cm^{-1}), l is the beam path length through quartz cuvette, and Φ_λ is the photochemical quantum yield of the actinometer (potassium ferrioxalate), the results were listed in Table S4.

Table S4. Photon flux of the excitation light sources.

Wavelength (nm)	395 nm	500 nm
Photon flux (mol s^{-1})	0.0000189	0.0000636

Measurement of quantum yields

Samples of either pure *E* isomers or *Z*-rich mixtures in acetonitrile were prepared for quantum yield experiments. A solution of 3.0 mL was irradiated under magnetic stirring, and the beam path length through quartz cuvette was 1 cm. The change of absorbance with time *t* was recorded on Shimadzu UV-2700 spectrophotometer with slit width of 2.0 nm. The fractions of *Z* or *E* isomer were plotted against the integrated photokinetic factor x(t), followed by exponential fitting (Figure S17–S20).

Table S5. Photoisomerization quantum yields (Φ) of **2, 5** and **10** in acetonitrile.

Azo switch	Quantum yield	
	E→*Z*	*Z*→*E*
	π–π* excitation[a]	n–π* excitation[b]
**2**	0.441	0.537
**5**	0.426	0.424
**10**	0.311	0.535

The excitation wavelength [a] 395 nm; [b] 500 nm.

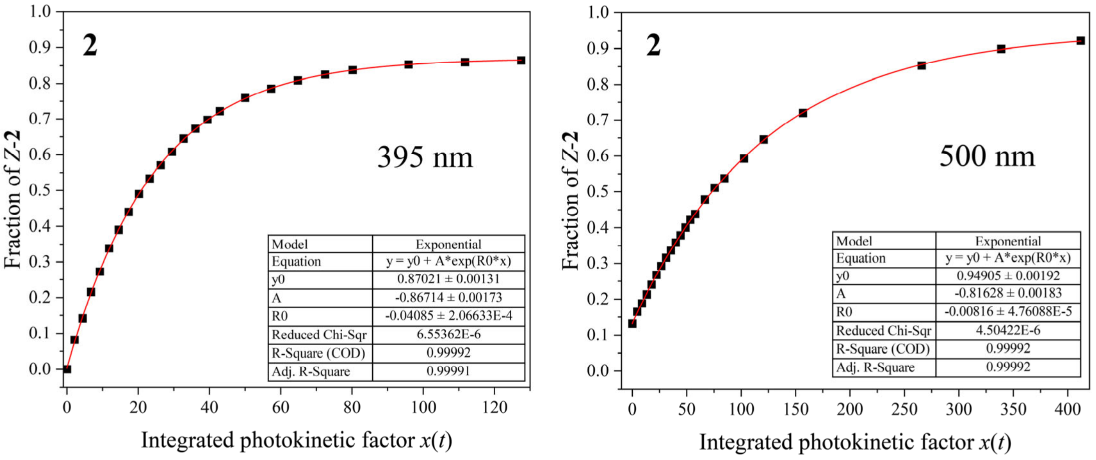

Figure S18. Exponential fitting of the fractions of *Z* or *E* isomer against the integrated photokinetic factors x(t) for **2** in MeCN.

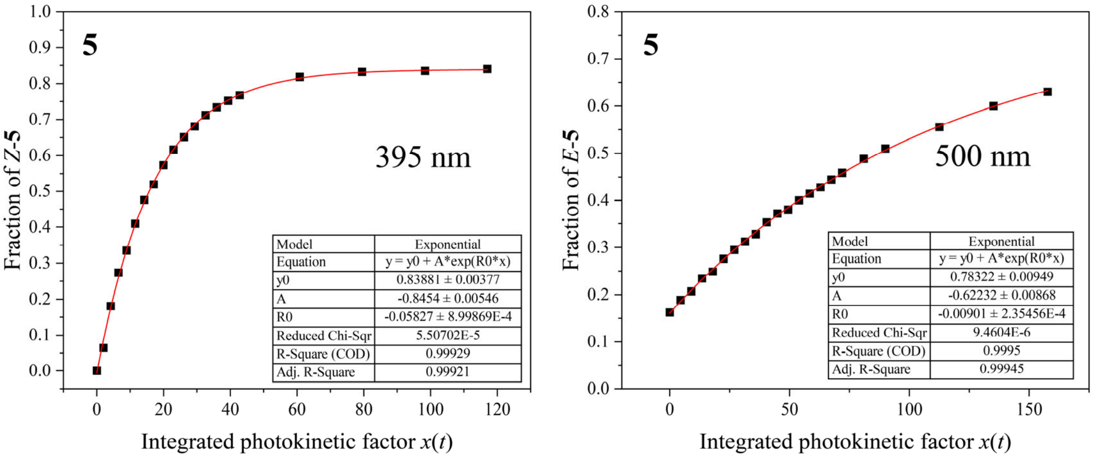

Figure S19. Exponential fitting of the fractions of *Z* or *E* isomer against the integrated photokinetic factors x(t) for **5** in MeCN.

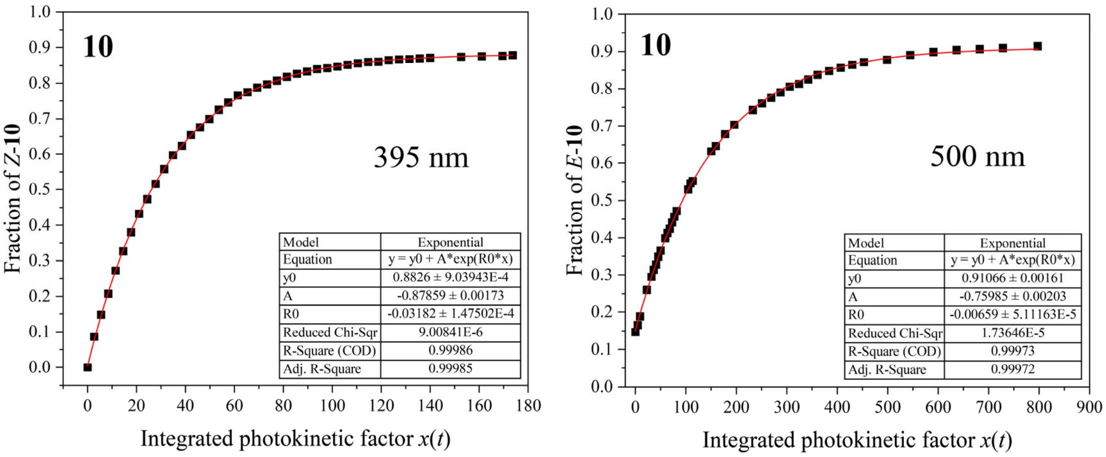

Figure S20. Exponential fitting of the fractions of *Z* or *E* isomer against the integrated photokinetic factors x(t) for **10** in MeCN.

4. Thermal isomerization kinetics

The measurement was performed in DMSO at elevated temperatures. DMSO has been a favored solvent when $t_{1/2}$ need to be measured at high temperatures. The thermal *Z*→*E* isomerization of azo follows first order kinetics and Arrhenius equation is used to calculate the thermal half-life.

$$\ln k = -\frac{E_a}{RT} + \ln A \text{ (Arrhenius equation)}$$

where k = the rate constant, E_a = activation energy, R = gas constant, T = absolute temperature, A = pre-exponential factor;

Meanwhile, the enthalpy of activation ($\Delta H^{\ddagger}$) and entropy of activation ($\Delta S^{\ddagger}$) are calculated according to Eyring equation to further obtain the free energy of activation ($\Delta G^{\ddagger}$).

$$\ln \frac{kh}{k_B T} = -\frac{\Delta H^{\ddagger}}{RT} + \frac{\Delta S^{\ddagger}}{R} \text{ (Eyring equation)}$$

$$\Delta G^{\ddagger} = \Delta H^{\ddagger} - T\Delta S^{\ddagger} \text{ (Free energy of activation (free energy barrier))}$$

where k = the rate constant, h = Planck's constant, k_B = Boltzmann constant, T = absolute temperature, R = gas constant, $\Delta H^{\ddagger}$ = enthalpy of activation, $\Delta S^{\ddagger}$ = entropy of activation.

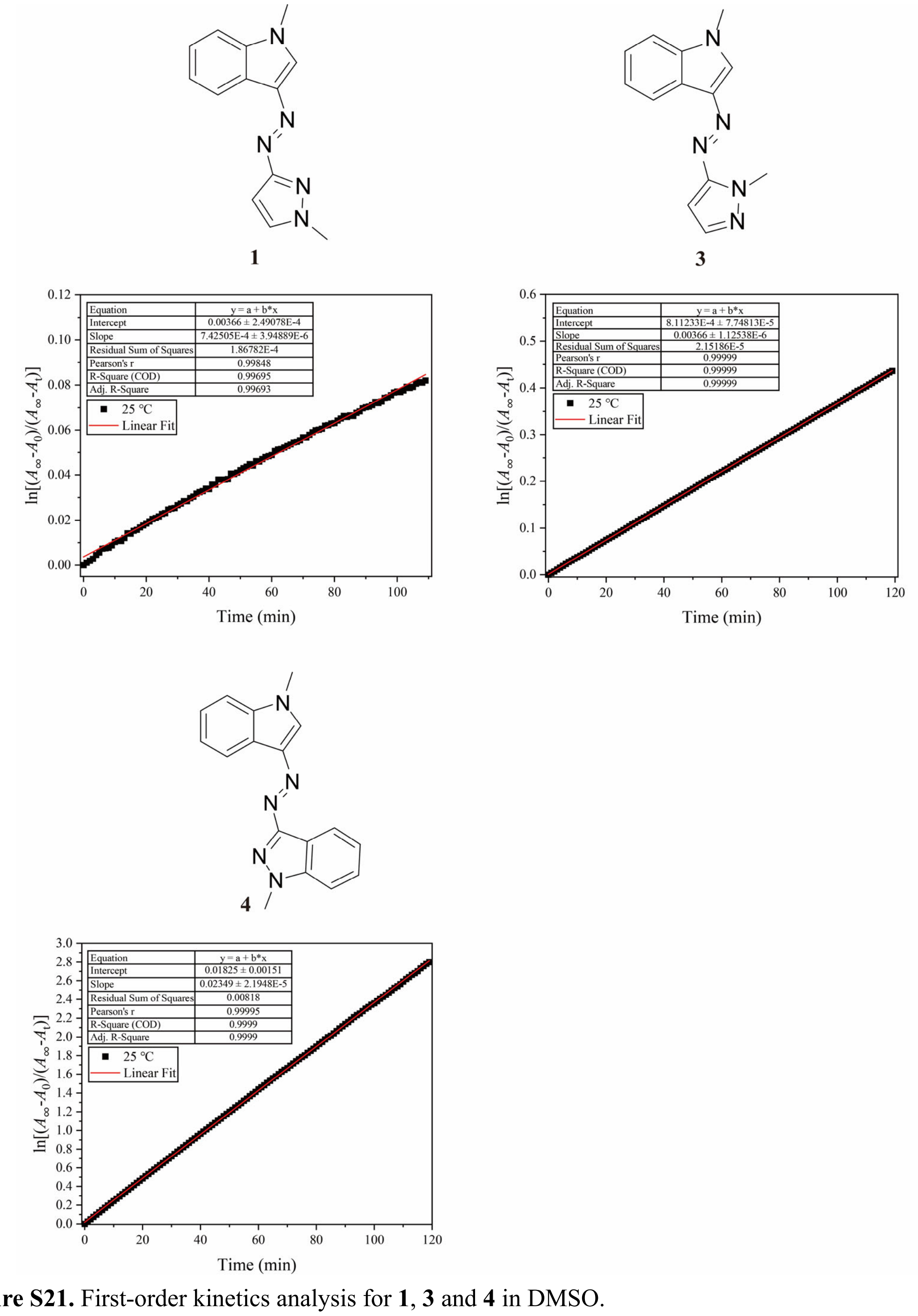

Figure S21. First-order kinetics analysis for **1**, **3** and **4** in DMSO.

Table S6. Thermal isomerization kinetics results of **1**, **3** and **4** in DMSO.

Experimental			
Compound	**1**	**3**	**4**
k (min^{-1})	7.43×10^{-4}	3.66×10^{-3}	2.35×10^{-2}
$t_{1/2}$ (h)	15.55	3.16	0.49

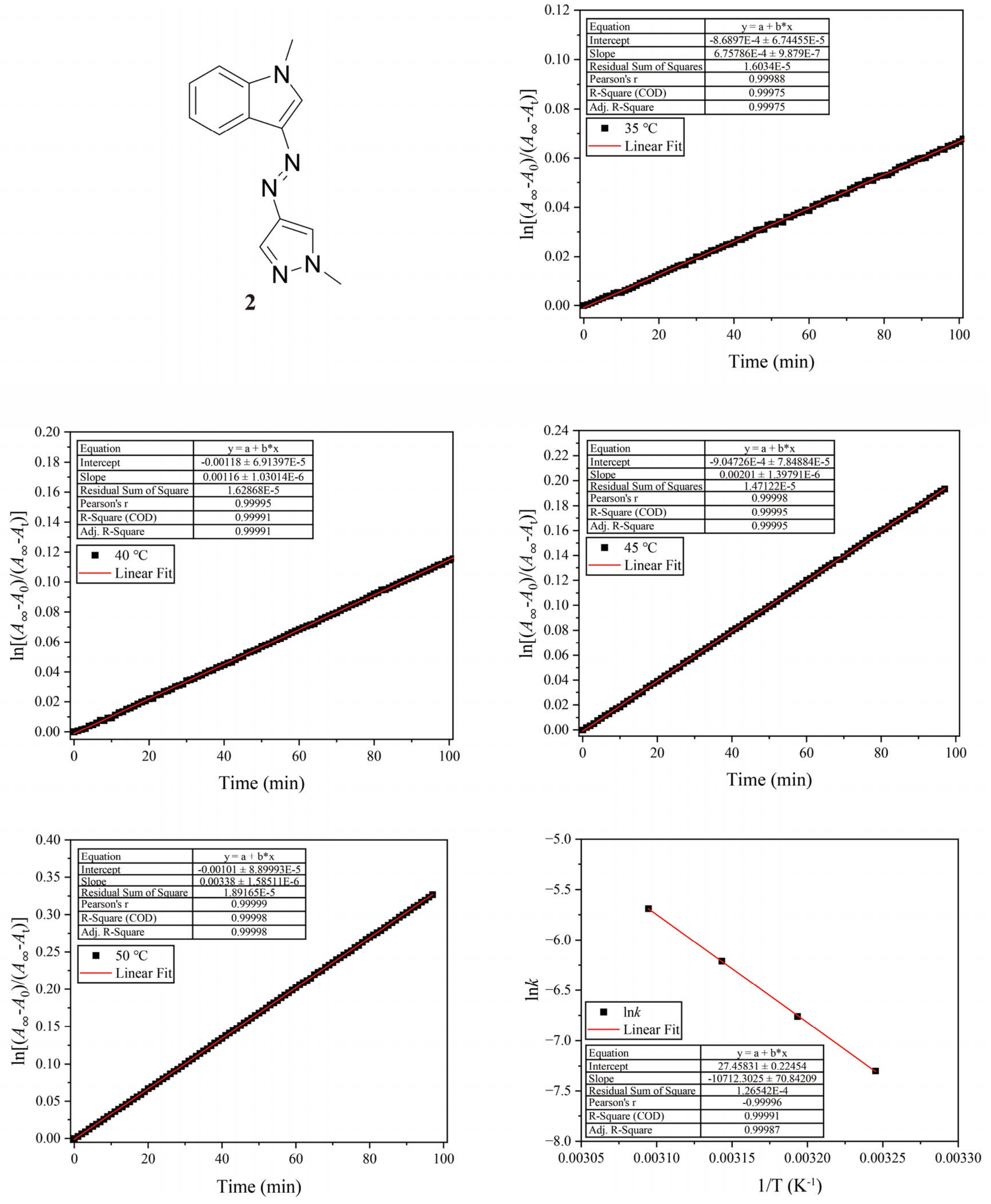

Figure S22. First-order kinetics analysis for **2** in DMSO.

Table S7. Thermal isomerization kinetics results of **2** in DMSO.

	Experimental				Calculated
Temp. (°C)	35	40	45	50	25
k (min^{-1})	6.76×10^{-4}	1.16×10^{-3}	2.01×10^{-3}	3.38×10^{-3}	2.09×10^{-4}
$t_{1/2}$ (h)	17.09	9.96	5.75	3.42	55.27

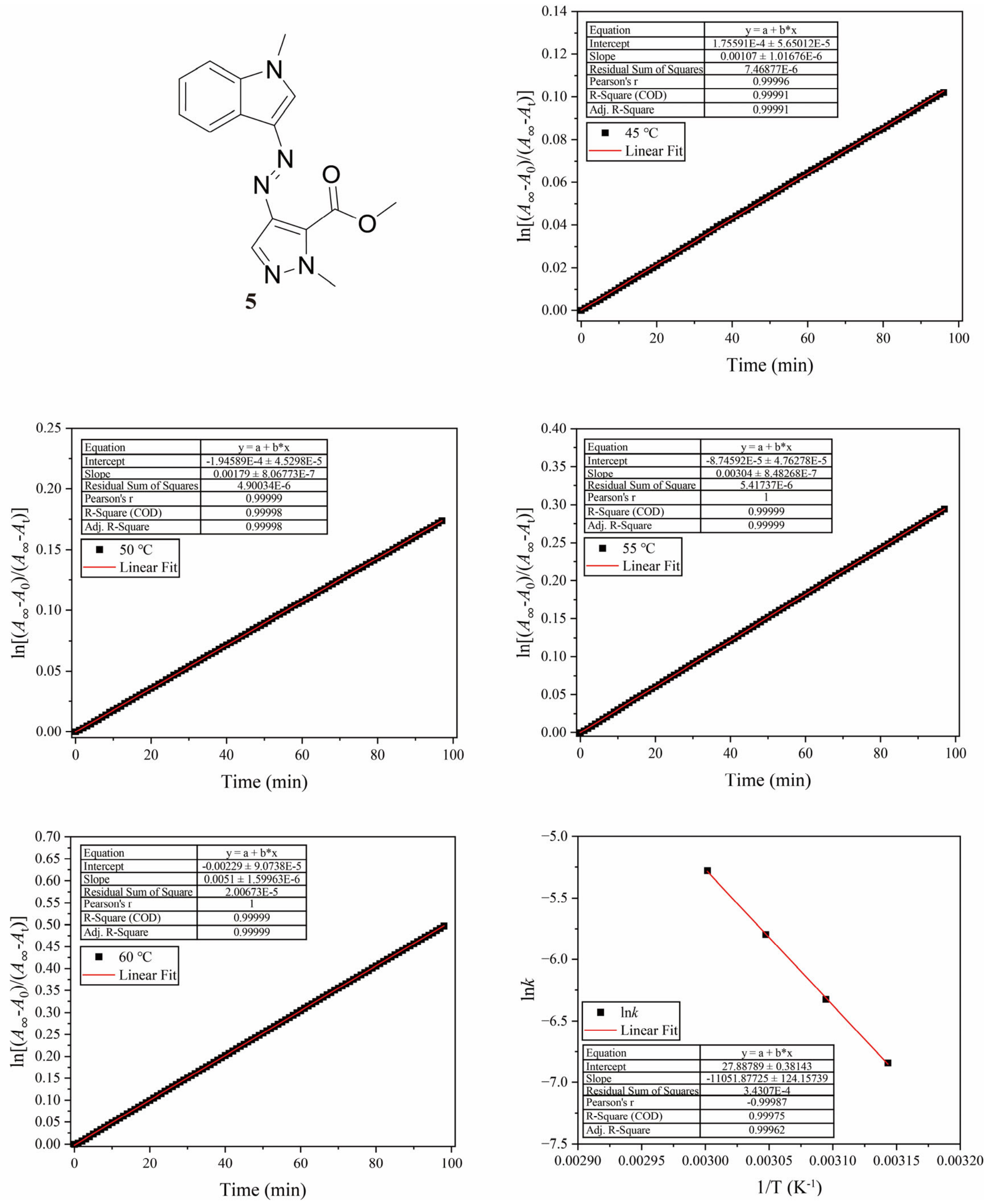

Figure S23. First-order kinetics analysis for **5** in DMSO.

Table S8. Thermal isomerization kinetics results of **5** in DMSO.

	Experimental				Calculated
Temp. (°C)	45	50	55	60	25
k (min^{-1})	1.07×10^{-3}	1.79×10^{-3}	3.04×10^{-3}	5.10×10^{-3}	1.03×10^{-4}
$t_{1/2}$ (h)	10.80	6.45	3.80	2.27	112.16

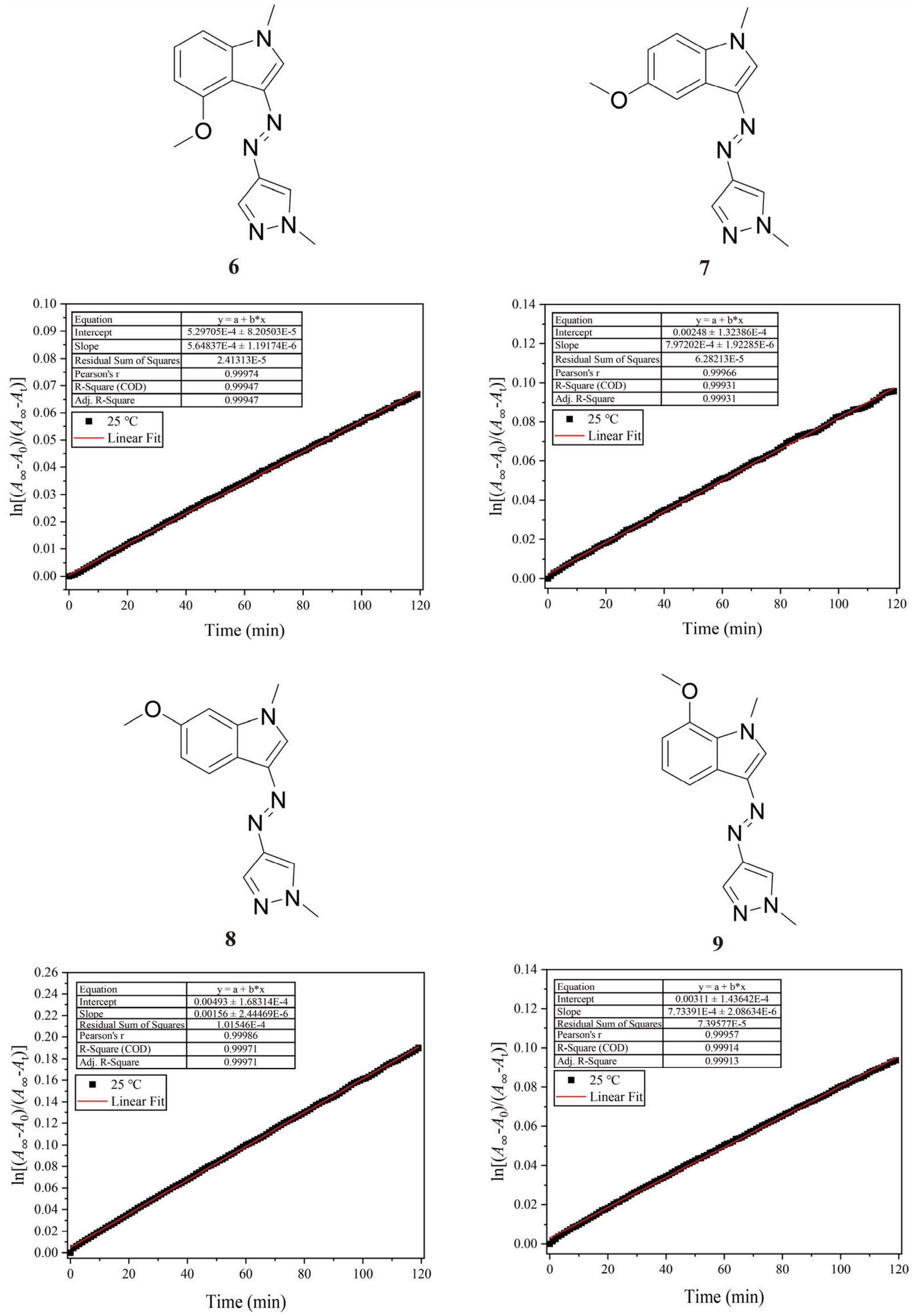

Figure S24. First-order kinetics analysis for **6-9** in DMSO.

Table S9. Thermal isomerization kinetics results of **6-9** in DMSO.

	Experimental			
Compound	**6**	**7**	**8**	**9**
k (min^{-1})	5.65×10^{-4}	7.97×10^{-4}	1.56×10^{-3}	7.73×10^{-4}
$t_{1/2}$ (h)	20.45	14.49	7.41	14.94

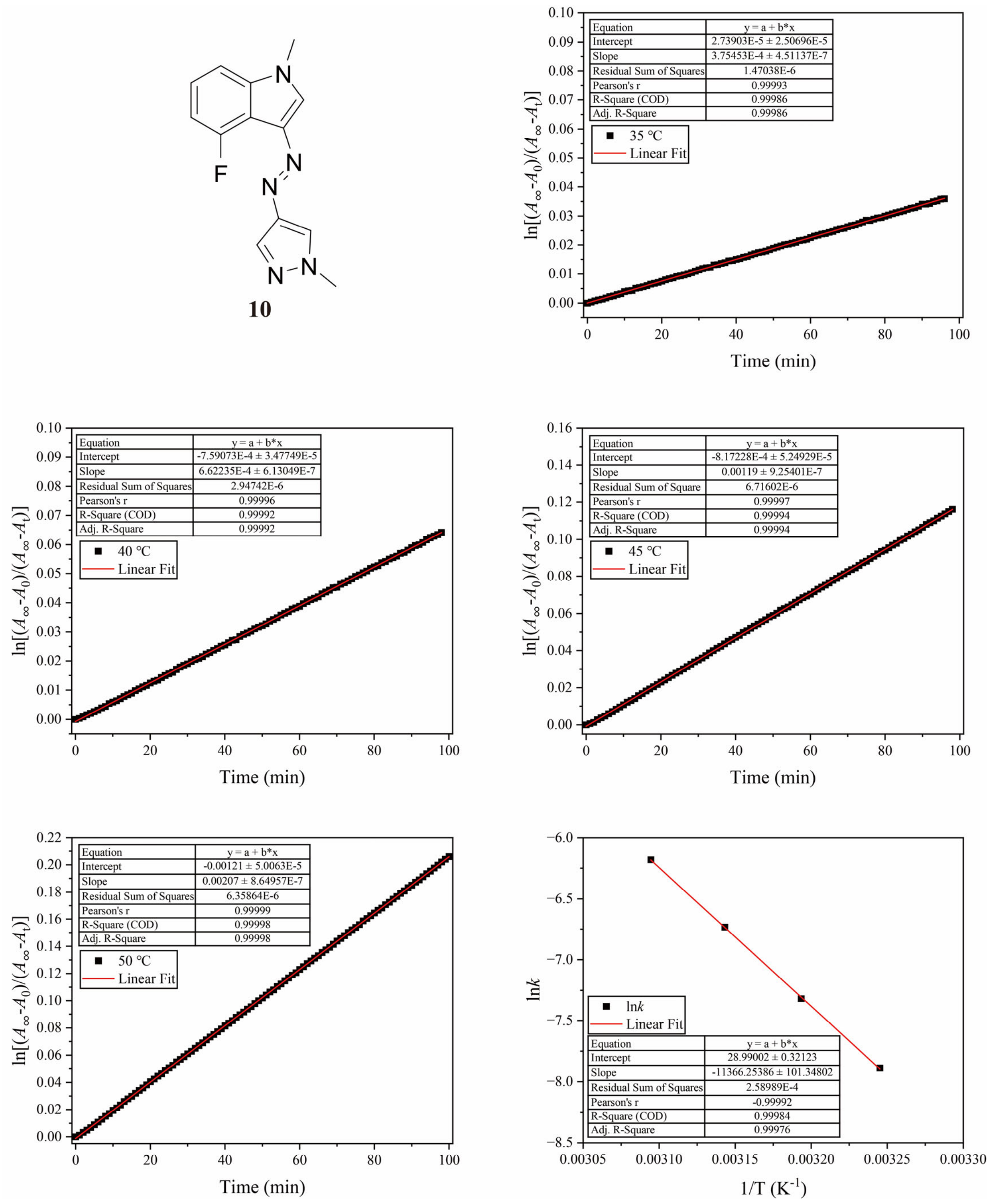

Figure S25. First-order kinetics analysis for **10** in DMSO.

Table S10. Thermal isomerization kinetics results of **10** in DMSO.

	Experimental				Calculated
Temp. (℃)	35	40	45	50	25
k (min^{-1})	3.75×10^{-4}	6.62×10^{-4}	1.19×10^{-3}	2.07×10^{-3}	1.08×10^{-4}
$t_{1/2}$ (h)	30.80	17.45	9.71	5.58	106.88

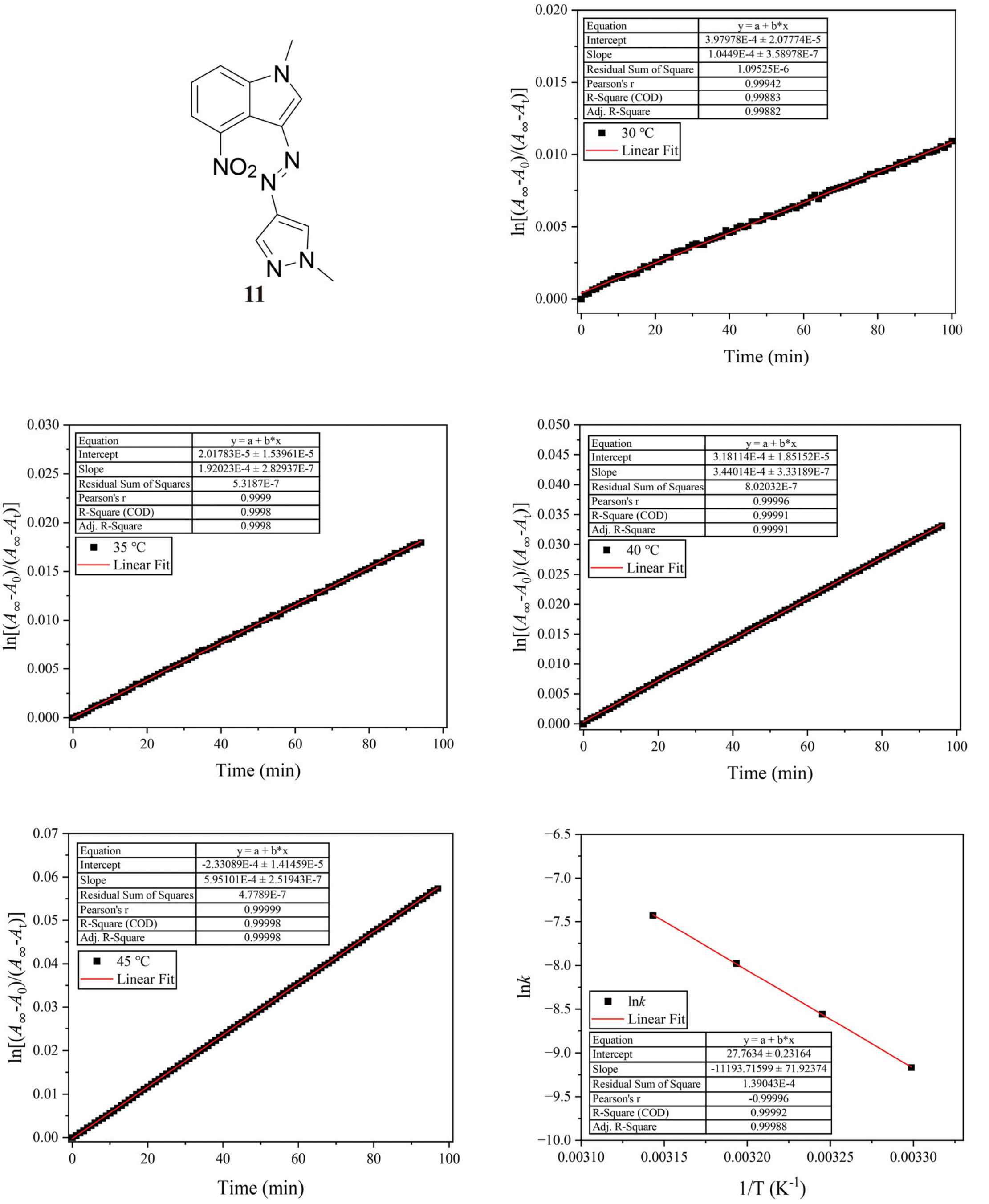

Figure S26. First-order kinetics analysis for **11** in DMSO.

Table S11. Thermal isomerization kinetics results of **11** in DMSO.

	Experimental				Calculated
Temp. (°C)	30	35	40	45	25
k (min^{-1})	1.05×10^{-4}	1.92×10^{-4}	3.44×10^{-4}	5.95×10^{-3}	5.65×10^{-5}
$t_{1/2}$ (h)	110.02	60.17	33.58	19.42	204.46

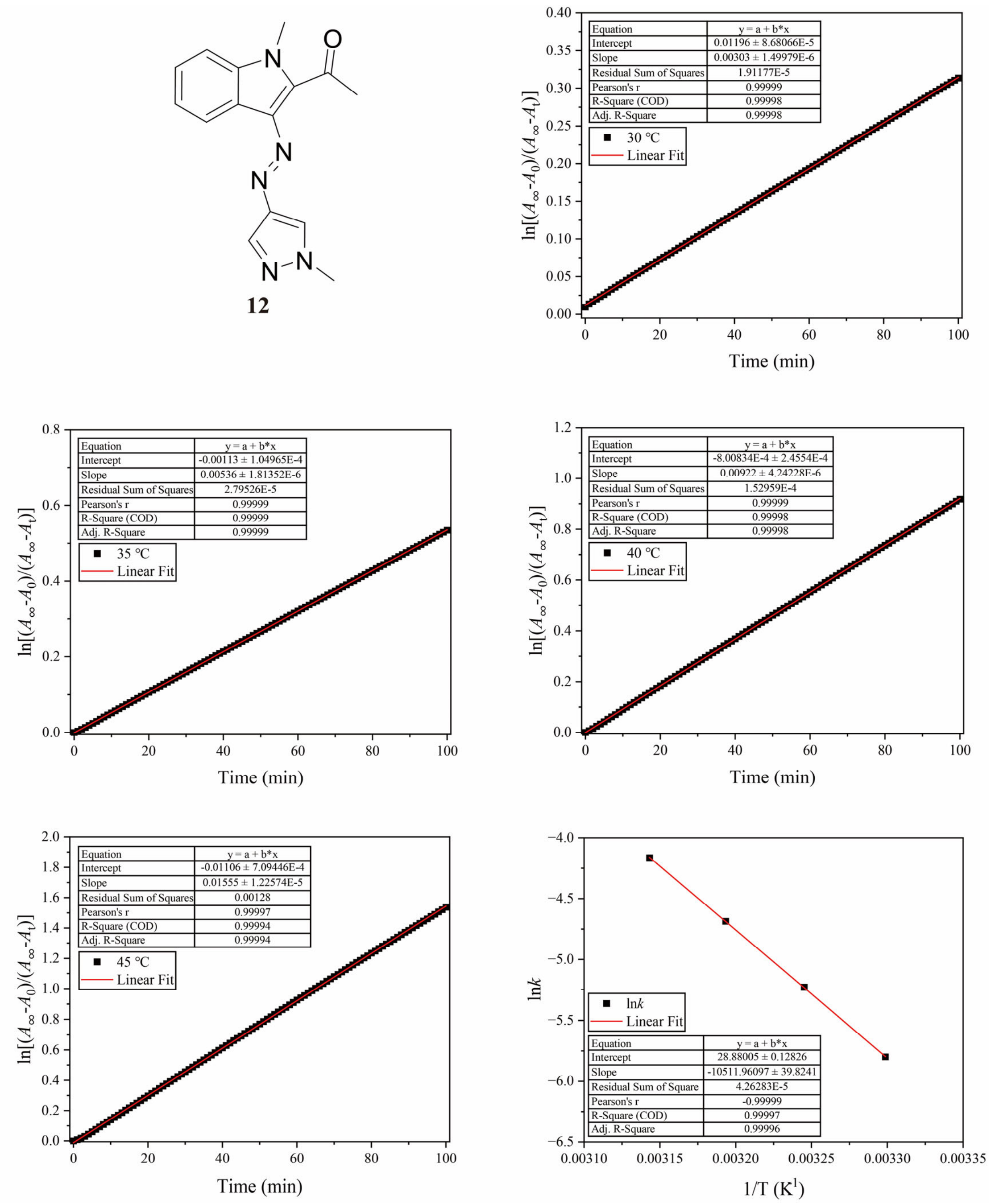

Figure S27. First-order kinetics analysis for **12** in DMSO.

Table S12. Thermal isomerization kinetics results of **12** in DMSO.

	Experimental				Calculated
Temp. (°C)	30	35	40	45	25
k (min^{-1})	3.03×10^{-3}	5.36×10^{-3}	9.22×10^{-3}	1.56×10^{-2}	1.70×10^{-3}
$t_{1/2}$ (h)	3.81	2.16	1.25	0.74	6.80

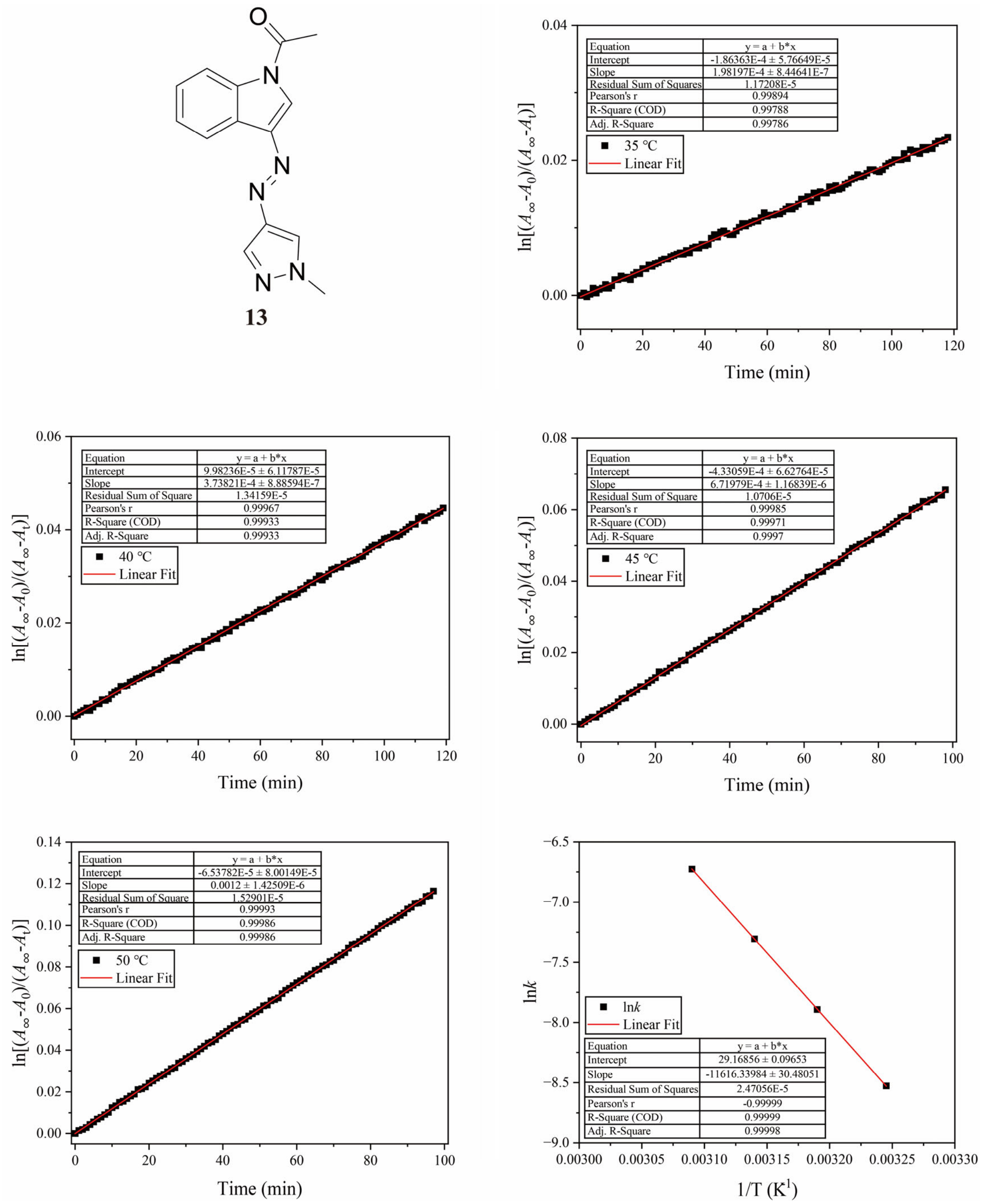

Figure S28. First-order kinetics analysis for **13** in DMSO.

Table S13. Thermal isomerization kinetics results of **13** in DMSO.

	Experimental				Calculated
Temp. (°C)	35	40	45	50	25
k (min^{-1})	1.98×10^{-4}	3.74×10^{-4}	6.72×10^{-4}	1.20×10^{-3}	5.59×10^{-5}
$t_{1/2}$ (h)	58.35	30.89	17.19	9.63	206.66

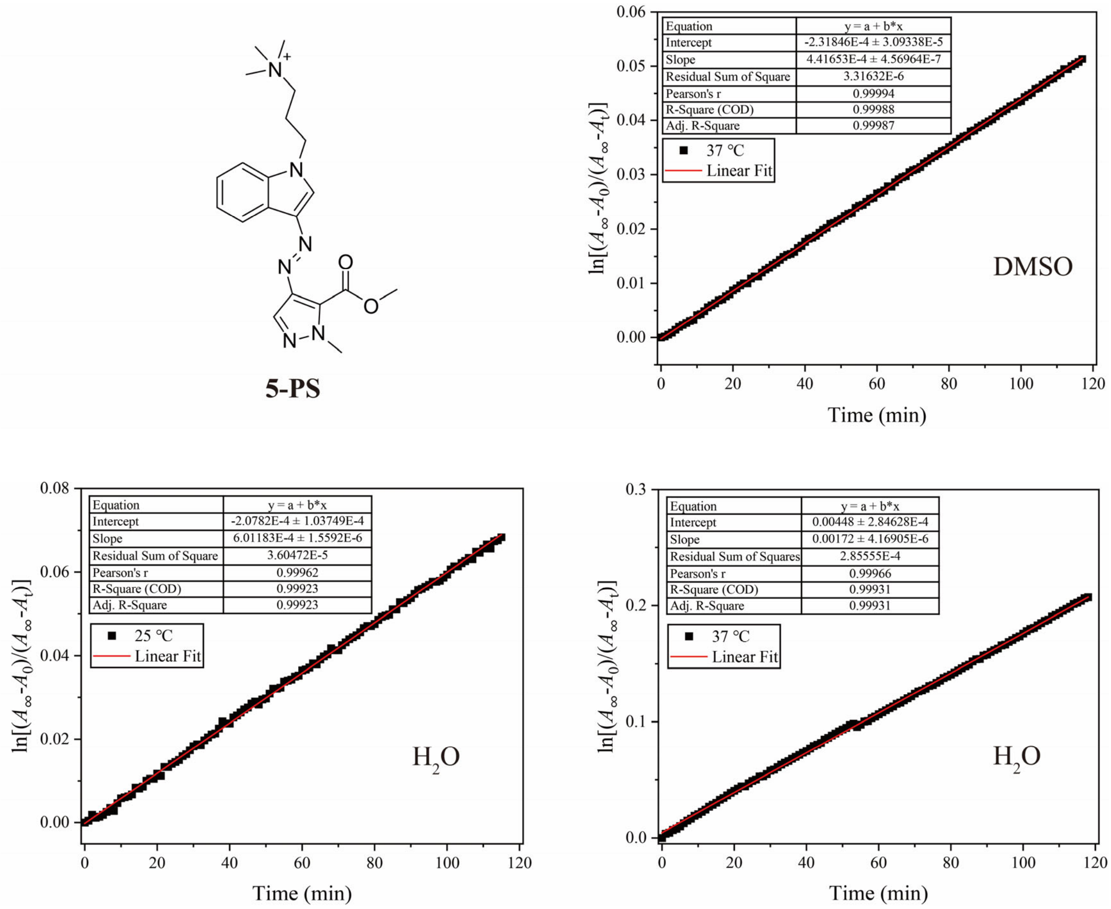

Figure S29. First-order kinetics analysis for **5-PS** in H_2O or DMSO.

Table S14. Thermal isomerization kinetics results of **5-PS** in H_2O or DMSO.

Experimental			
solvent	DMSO	H_2O	H_2O
Temp. (°C)	37 °C	25 °C	37 °C
k (min^{-1})	4.42×10^{-4}	6.01×10^{-4}	1.72×10^{-3}
$t_{1/2}$ (h)	26.14	19.22	6.72

Table S15. Thermal parameters of the thermal isomerization of **2**, **5**, **10**–**13** in DMSO.

Compound	$t_{1/2}$ (25 °C/h)	E_a (kJ/mol)	$\Delta S^{\ddagger}$ (J/(mol·K))	$\Delta H^{\ddagger}$ (kJ/mol)	$\Delta G^{\ddagger}$ (kJ/mol)	lnA
**2**	55.27	89.06	-59.00	86.58	104.17	27.45
**5**	112.16	91.89	-55.41	89.41	105.93	27.89
**10**	106.88	94.50	-46.25	92.02	105.81	28.99
**11**	204.46	93.06	-56.45	90.59	107.42	27.76
**12**	6.80	87.40	-47.16	84.92	98.98	28.88
**13**	206.66	96.58	-44.75	94.10	107.44	29.17

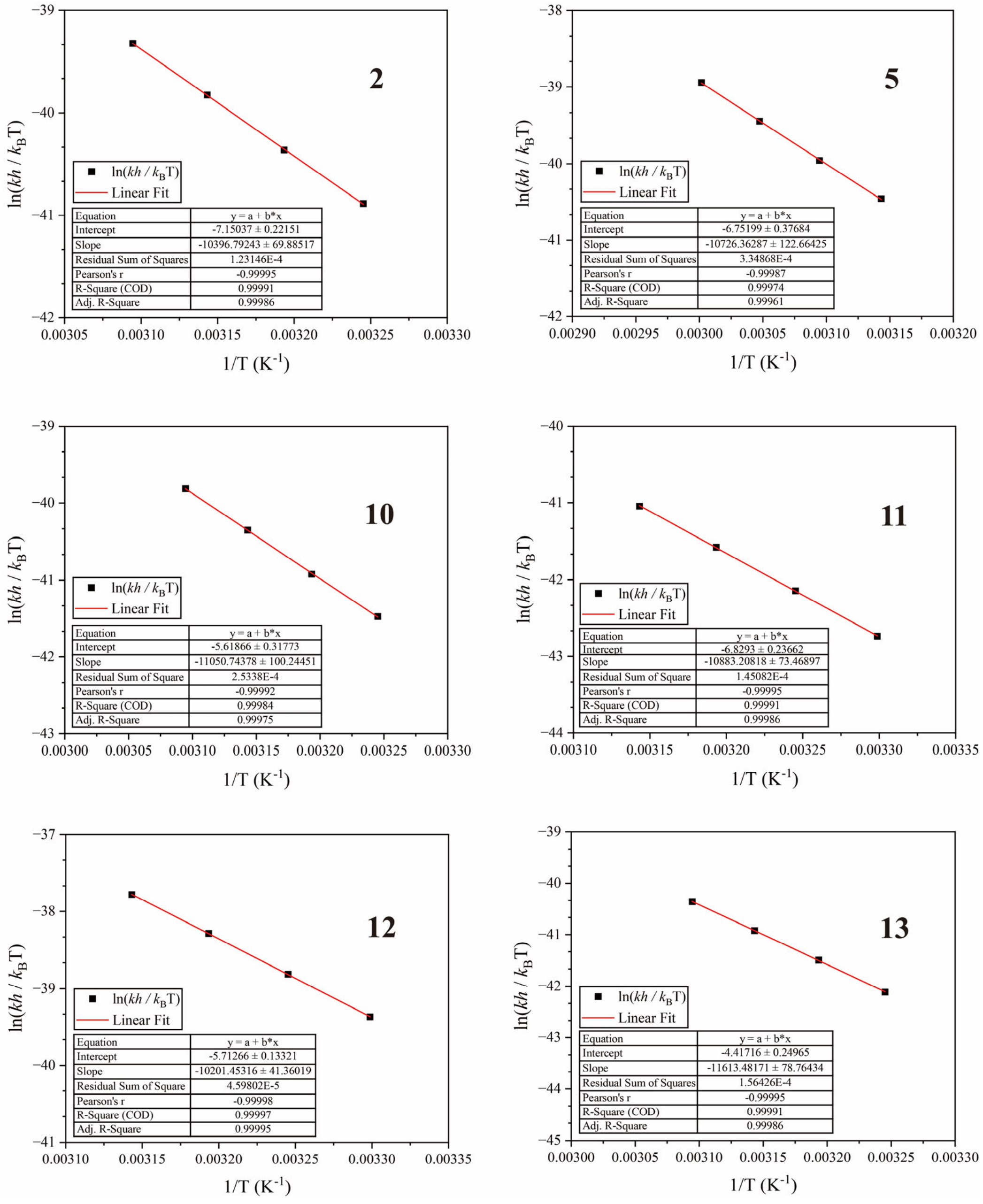

Figure S30. Eyring plot of thermal $Z \rightarrow E$ isomerization for azoindoles in DMSO.

5. Cycling stability and cytotoxicity

The cycling stability of **5, 10** and **5-PS** were measured *via* UV-Vis spectrophotometry (Shimadzu UV-2700), the samples were irradiated alternately by violet light (400 nm) (1 min) and green light (549 nm) (30-60 min).

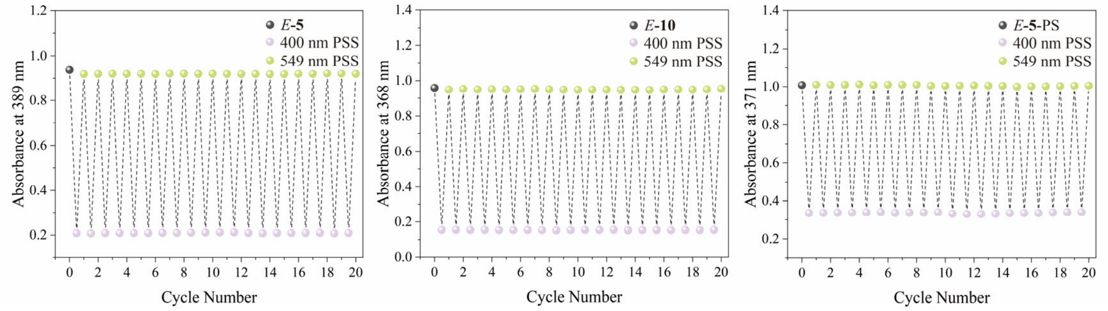

Figure S31. Cycling stability measures. The gray circle represents the absorbance before irradiation, violet circle represents the absorbance after 400 nm light irradiation and green circle represents the absorbance after 549 nm light irradiation.

HeLa and MDA-MB-231 cells were treated with *E*-**5-PS** and 400 nm PSS group, cultured for 48 h and characterized by MTT method, then by fitting a diagram.

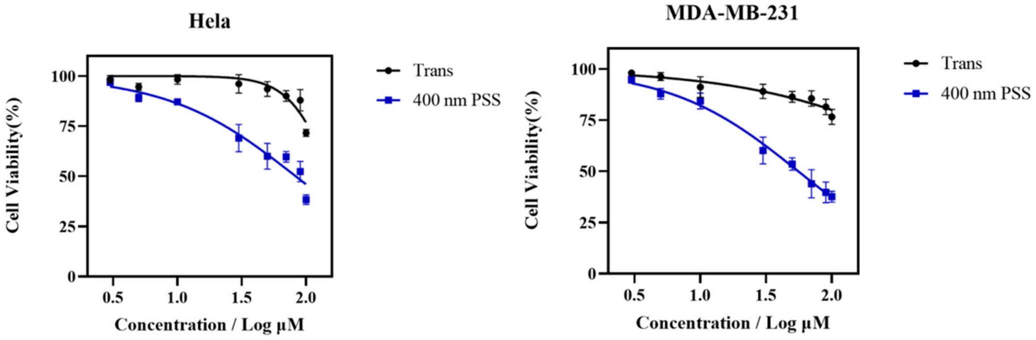

Figure S32. Antiproliferative activity on Hela and MDA-MB-231, using both *E*-**5-PS** and 400 nm PSS (*Z*-rich-**5-PS**).

Table S16. HepG2 cell viability in response to varying concentrations of (*E*/*Z*-rich)**-5-PS**.

Concentration (μM)	3.0	5.0	10.0	30.0	50.0	70.0	90.0	100.0
	100.0	97.5	100.9	90.8	82.5	78.5	65.0	63.2
trans<br>Cell viability (%)	97.4	96.4	96.6	93.9	85.0	84.0	69.0	61.9
	99.8	98.6	97.2	83.6	92.4	88.0	61.7	59.8
(Table S20. continued)								
400 nm PSS<br>Cell viability (%)	96.8	96.7	96.1	65.4	46.5	37.1	8.0	8.7

	95.6	98.8	92.9	68.2	42.1	23.5	15.0	7.4
	95.0	96.0	94.3	74.3	50.3	39.4	12.6	6.9

Table S17. HeLa cell viability in response to varying concentrations of (*E*/*Z*-rich)**-5-PS**.

Concentration (μM)	3.0	5.0	10.0	30.0	50.0	70.0	90.0	100.0
trans Cell viability (%)	100.0	96.5	100.0	97.3	94.1	92.7	92.0	73.2
	96.4	94.4	97.8	99.3	94.5	90.2	89.9	71.9
	98.8	93.0	96.6	98.7	88.4	87.4	81.9	69.8
400 nm PSS Cell viability (%)	97.8	90.7	87.8	74.3	66.4	59.8	57.9	40.7
	98.6	89.8	86.5	61.4	60.1	62.3	51.2	38.4
	95.0	87.0	88.2	71.4	53.5	57.0	48.0	35.9

Table S18. MDA-MB-231 cell viability in response to varying concentrations of (*E*/*Z*-rich)**-5-PS**.

Concentration (μM)	3.0	5.0	10.0	30.0	50.0	70.0	90.0	100.0
trans Cell viability (%)	99.0	96.5	95.8	91.5	93.3	82.1	78.7	76.7
	97.4	94.4	91.8	90.7	88.3	85.1	84.1	79.2
	97.8	93.0	85.8	85.0	87.5	89.6	81.2	73.9
400 nm PSS Cell viability (%)	92.8	86.7	87.1	60.3	54.1	45.8	45.6	36.9
	95.6	90.8	81.7	64.8	50.3	39.1	36.9	35.4
	96.0	86.0	84.9	55.5	56.4	48.9	36.7	40.7

6. Single crystal X-ray structures for compounds

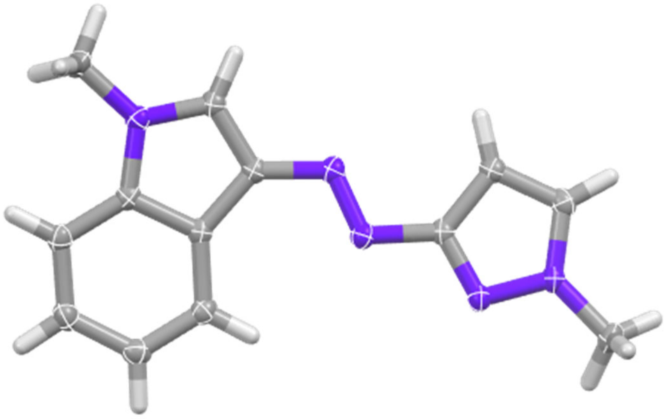

Figure S33. Single crystal X-ray structure of **1.** The crystals were grown from a concentrated solution of **1** in *n*-hexanes/DCM, using a slow evaporation. X-ray crystallographic data have been deposited in the Cambridge Crystallographic Data Centre database (http://www.ccdc.cam.ac.uk/) under accession code CCDC 2424490.

Table 19. Crystal data and structure refinement for **1**

Empirical formula	$C_{13}H_{13}N_5$
Formula weight	239.28
Temperature	100(2) K
Wavelength	1.54184 A
Crystal system, space group	*Monoclinic, P2(1)/n*
	a = 6.41810(10) A α = 90 deg.
Unit cell dimensions	b = 14.1610(3) A β = 101.423(2) deg.
	c = 13.2275(3) A γ = 90 deg.
Volume	1178.39(4) A^3
Z, Calculated density	4, 1.349 Mg/m^3
Absorption coefficient	0.693 mm^{-1}
F(000)	504
Crystal size	0.160 x 0.140 x 0.120 mm
Theta range for data collection	4.624 to 77.561 deg.
Limiting indices	$-4 \leq h \leq 7$, $-17 \leq k \leq 17$, $-16 \leq l \leq 16$
Reflections collected / unique	13047 / 2436 [*R(int)* = 0.0513]
Completeness to theta = 67.684	99.8 %
Absorption correction	Semi-empirical from equivalents
Refinement method	Full-matrix least-squares on F^2
Data / restraints / parameters	2436 / 0 / 165
Goodness-of-fit on F^2	1.091
Final R indices [*I>2sigma(I)*]	R_1 = 0.0436, wR_2 = 0.1249
R indices (all data)	R_1 = 0.0490, wR_2 = 0.1286
Extinction coefficient	*n/a*
Largest diff. peak and hole	0.311 and -0.298 $e.A^{-3}$

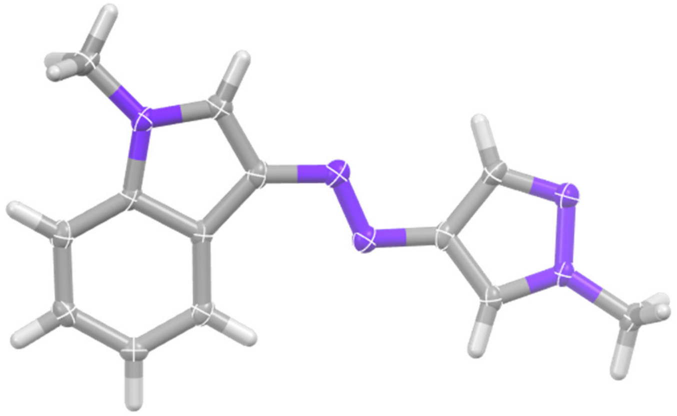

Figure S34. Single crystal X-ray structure of **2.** The crystals were grown from a concentrated solution of **2** in *n*-hexanes/DCM, using a slow evaporation. X-ray crystallographic data have been deposited in the Cambridge Crystallographic Data Centre database (http://www.ccdc.cam.ac.uk/) under accession code CCDC 2424491.

Table 20. Crystal data and structure refinement for **2**

Empirical formula	$C_{13}H_{13}N_5$
Formula weight	239.28
Temperature	100(2) K
Wavelength	1.54184 A
Crystal system, space group	*Monoclinic, P2(1)*
	a = 7.07950(10) A α = 90 deg.
Unit cell dimensions	b = 13.8474(3) A β = 101.205(2) deg.
	c = 12.6001(3) A γ = 90 deg.
Volume	1211.68(4) A^3
Z, Calculated density	4, 1.312 Mg/m^3
Absorption coefficient	0.674 mm^{-1}
F(000)	504
Crystal size	0.180 x 0.160 x 0.140 mm
Theta range for data collection	3.576 to 77.230 deg.
Limiting indices	$-8 \leq h \leq 7, -17 \leq k \leq 17, -15 \leq l \leq 15$
Reflections collected / unique	13680 / 4743 [*R(int)* = 0.0476]
Completeness to theta = 67.684	99.8 %
Absorption correction	Semi-empirical from equivalents
Refinement method	Full-matrix least-squares on F^2
Data / restraints / parameters	4743 / 0 / 330
Goodness-of-fit on F^2	0.859
Final R indices [*I>2sigma(I)*]	R_1 = 0.0423, wR_2 = 0.1002
R indices (all data)	R_1 = 0.0458, wR_2 = 0.1025
Extinction coefficient	*n/a*
Largest diff. peak and hole	0.266 and -0.244 $e.A^{-3}$

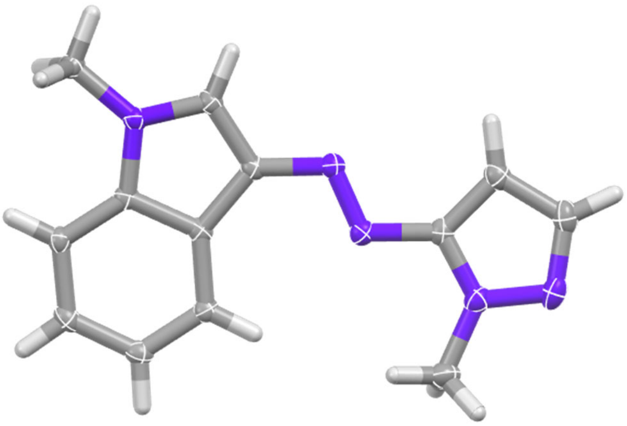

Figure S35. Single crystal X-ray structure of **3.** The crystals were grown from a concentrated solution of **3** in *n*-hexanes/DCM, using a slow evaporation. X-ray crystallographic data have been deposited in the Cambridge Crystallographic Data Centre database (http://www.ccdc.cam.ac.uk/) under accession code CCDC 2424493.

Table 21. Crystal data and structure refinement for **3**

Empirical formula	$C_{13}H_{13}N_5$
Formula weight	239.28
Temperature	100(2) K
Wavelength	1.54184 A
Crystal system, space group	*Orthorhombic, P2(1)2(1)2(1)*
	a = 5.60170(10) A α = 90 deg.
Unit cell dimensions	b = 8.7826(2) A β = 90 deg.
	c = 23.5685(5) A γ = 90 deg.
Volume	1159.51(4) A^3
Z, Calculated density	4, 1.371 Mg/m^3
Absorption coefficient	0.705 mm^{-1}
F(000)	504
Crystal size	0.150 x 0.140 x 0.120 mm
Theta range for data collection	3.571 to 77.387 deg.
Limiting indices	$-4 \leq h \leq 6$, $-11 \leq k \leq 10$, $-29 \leq l \leq 29$
Reflections collected / unique	5535 / 2331 [*R(int)* = 0.0311]
Completeness to theta = 67.684	100.0 %
Absorption correction	Semi-empirical from equivalents
Refinement method	Full-matrix least-squares on F^2
Data / restraints / parameters	2331 / 0 / 165
Goodness-of-fit on F^2	1.071
Final R indices [*I*>*2sigma(I)*]	R_1 = 0.0379, wR_2 = 0.1021
R indices (all data)	R_1 = 0.0407, wR_2 = 0.1039
Extinction coefficient	*n/a*
Largest diff. peak and hole	0.265 and -0.229 e.A^{-3}

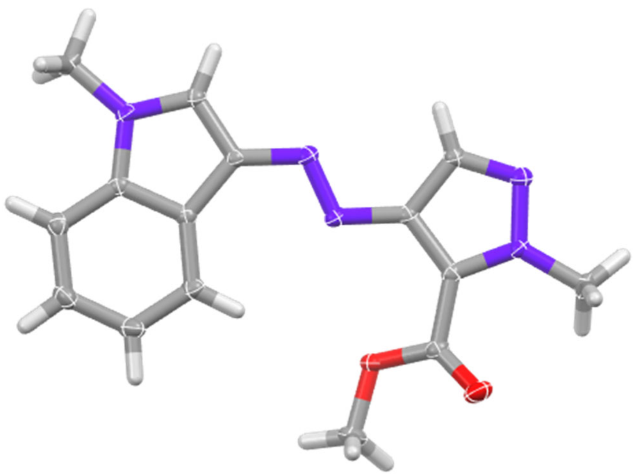

Figure S36. Single crystal X-ray structure of **5.** The crystals were grown from a concentrated solution of **5** in *n*-hexanes/DCM, using a slow evaporation. X-ray crystallographic data have been deposited in the Cambridge Crystallographic Data Centre database (http://www.ccdc.cam.ac.uk/) under accession code CCDC 2424494.

Table 22. Crystal data and structure refinement for **5**

Empirical formula	$C_{15}H_{15}N_5O_2$
Formula weight	297.32
Temperature	100(2) K
Wavelength	1.54184 A
Crystal system, space group	*Triclinic, P-1*
	a = 6.30080(10) A α = 88.117(2) deg.
Unit cell dimensions	b = 7.7125(2) A β = 80.529(2) deg.
	c = 15.2437(3) A γ = 83.044(2) deg.
Volume	725.23(3) A^3
Z, Calculated density	2, 1.362 Mg/m^3
Absorption coefficient	0.781 mm^{-1}
F(000)	312
Crystal size	0.180 x 0.160 x 0.140 mm
Theta range for data collection	2.939 to 75.881 deg.
Limiting indices	$-6 \leq h \leq 7$, $-9 \leq k \leq 9$, $-18 \leq l \leq 19$
Reflections collected / unique	11599 / 2915 [*R(int)* = 0.0134]
Completeness to theta = 67.684	98.4 %
Absorption correction	Semi-empirical from equivalents
Refinement method	Full-matrix least-squares on F^2
Data / restraints / parameters	2915 / 0 / 203
Goodness-of-fit on F^2	1.044
Final R indices [*I>2sigma(I)*]	R_1 = 0.0321, wR_2 = 0.0844
R indices (all data)	R_1 = 0.0330, wR_2 = 0.0852
Extinction coefficient	0.0164(14)
Largest diff. peak and hole	0.331 and -0.154 $e.A^{-3}$

7. Computational details

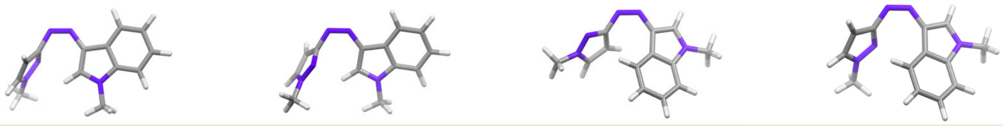

Z-**1** (1) *Z*-**1** (2) *Z*-**1** (3) *Z*-**1** (4)

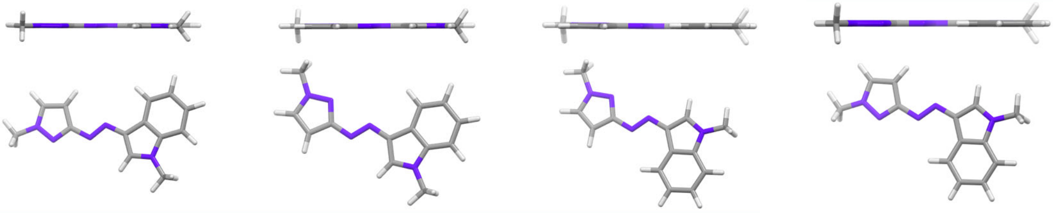

E-**1** (1) *E*-**1** (2) *E*-**1** (3) *E*-**1** (4)

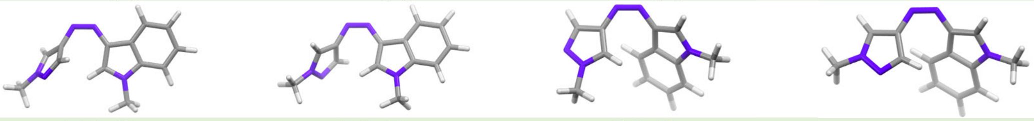

Z-**2** (1) *Z*-**2** (2) *Z*-**2** (3) *Z*-**2** (4)

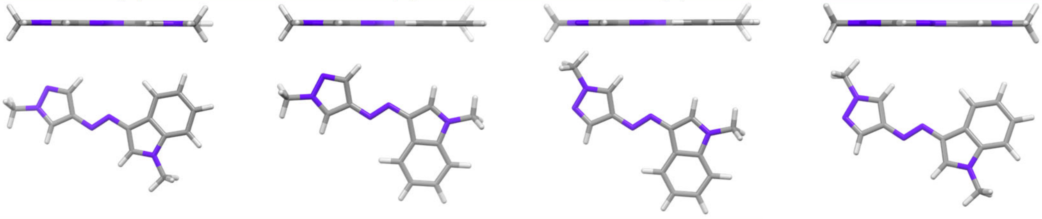

E-**2** (1) *E*-**2** (2) *E*-**2** (3) *E*-**2** (4)

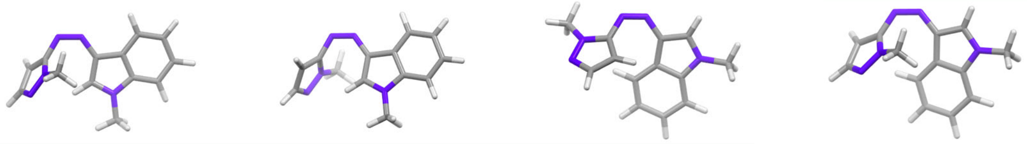

Z-**3** (1) *Z*-**3** (2) *Z*-**3** (3) *Z*-**3** (4)

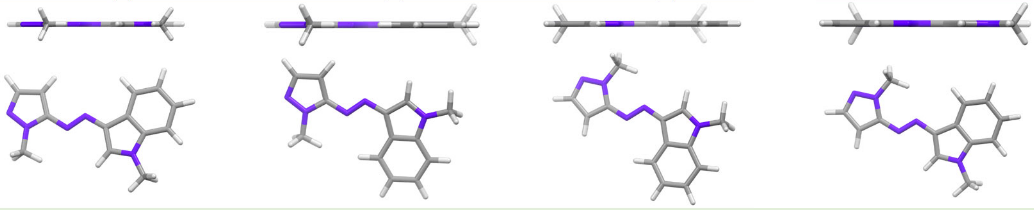

E-**3** (1) *E*-**3** (2) *E*-**3** (3) *E*-**3** (4)

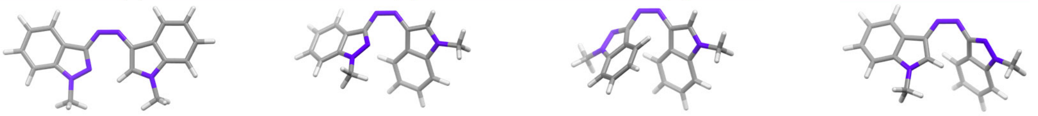

Z-**4** (1) *Z*-**4** (2) *Z*-**4** (3) *Z*-**4** (4)

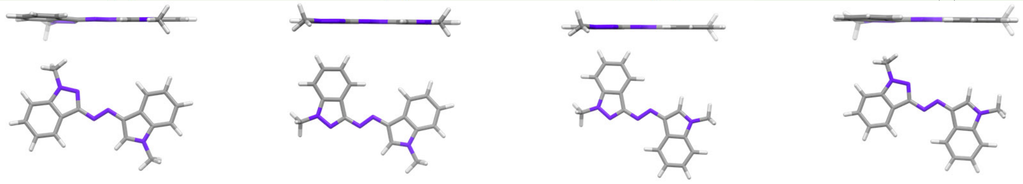

E-**4** (1) *E*-**4** (2) *E*-**4** (3) *E*-**4** (4)

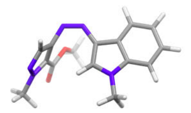
Z-**5** (**1**)

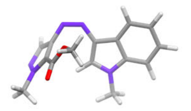
Z-**5** (2)

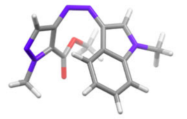
Z-**5** (3)

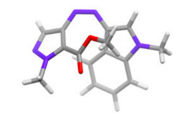
Z-**5** (4)

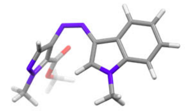
Z-**5** (5)

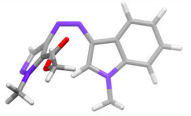
Z-**5** (6)

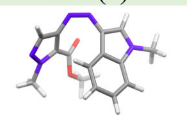
Z-**5** (7)

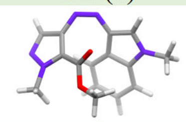
Z-**5** (8)

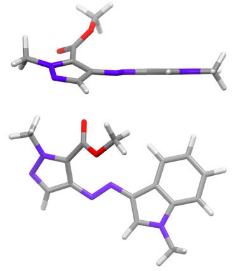
E-**5** (1)

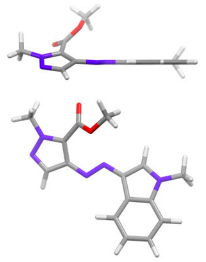
E-**5** (2)

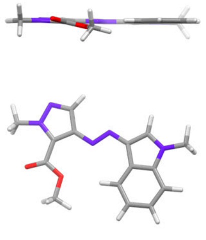
E-**5** (**3**)

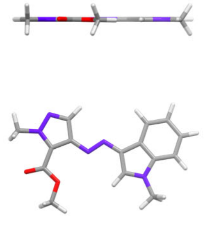
E-**5** (4)

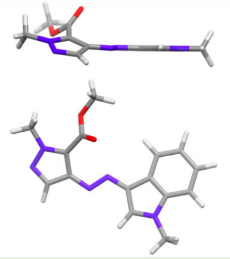
E-**5** (5)

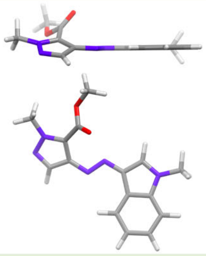
E-**5** (6)

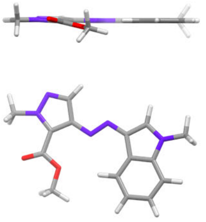
E-**5** (7)

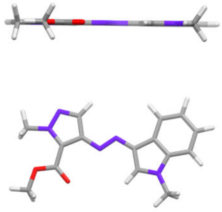
E-**5** (8)

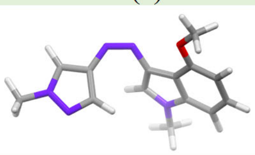
Z-**6** (1)

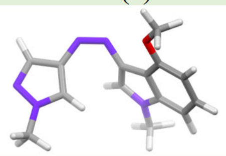
Z-**6** (2)

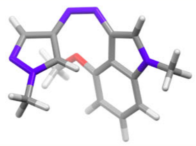
Z-**6** (**3**)

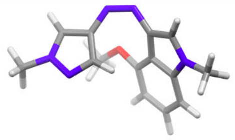
Z-**6** (4)

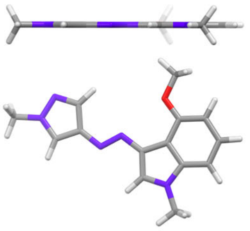
E-**6** (**1**)

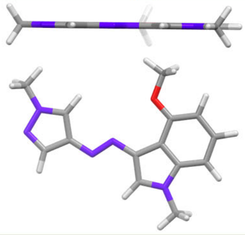
E-**6** (2)

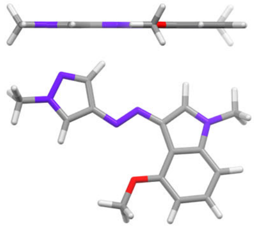
E-**6** (3)

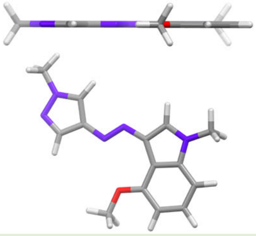
E-**6** (4)

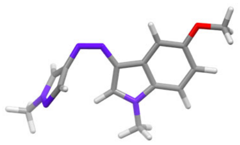
Z-**7** (1)

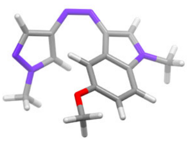
Z-**7** (2)

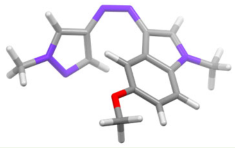
Z-**7** (**3**)

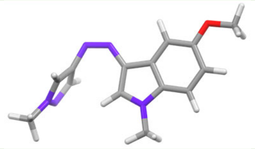
Z-**7** (4)

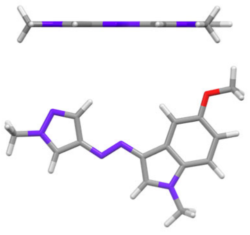
E-**7** (1)

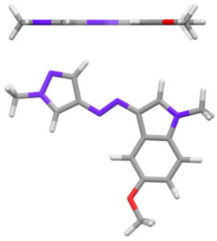
E-**7** (**2**)

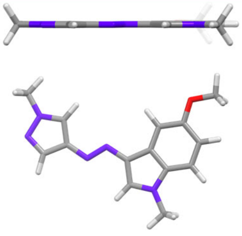
E-**7** (3)

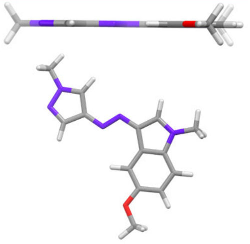
E-**7** (4)

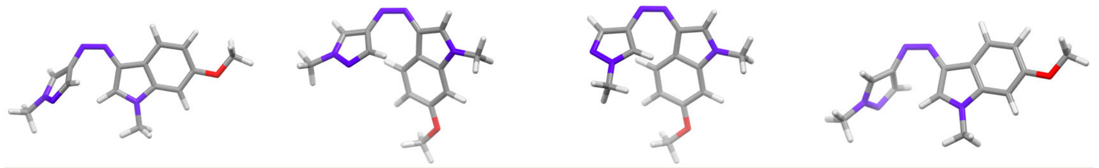

Z-**8** (1) *Z*-**8** (2) *Z*-**8** (**3**) *Z*-**8** (4)

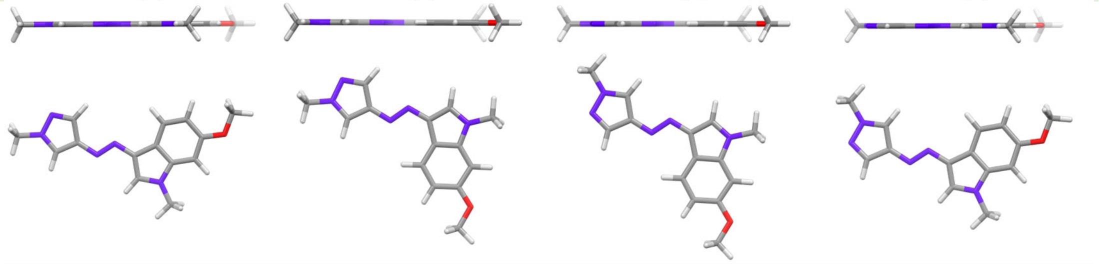

E-**8** (1) *E*-**8** (**2**) *E*-**8** (3) *E*-**8** (4)

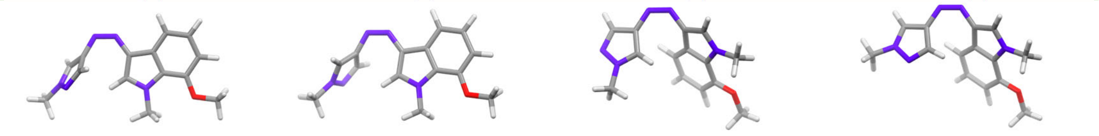

Z-**9** (1) *Z*-**9** (2) *Z*-**9** (3) *Z*-**9** (**4**)

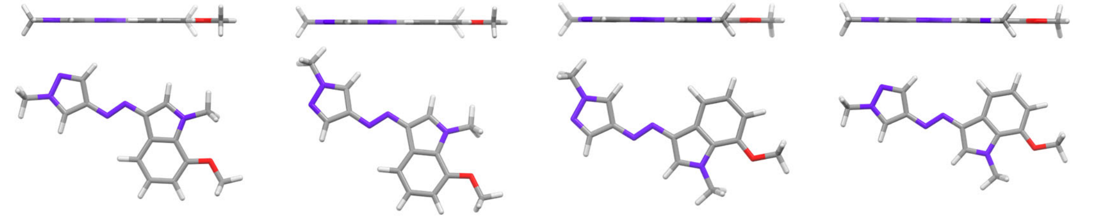

E-**9** (1) *E*-**9** (2) *E*-**9** (3) *E*-**9** (**4**)

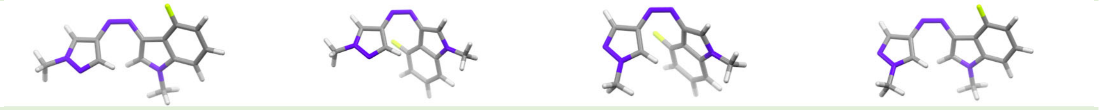

Z-**10** (1) *Z*-**10** (2) *Z*-**10** (**3**) *Z*-**10** (4)

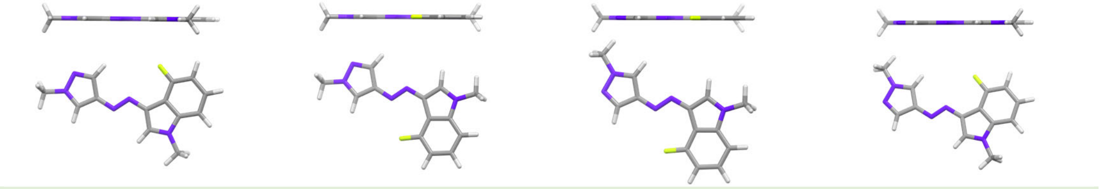

E-**10** (1) *E*-**10** (**2**) *E*-**10** (3) *E*-**10** (4)

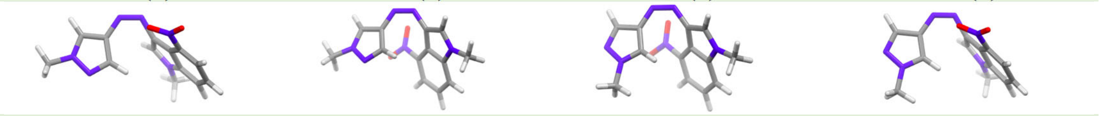

Z-**11** (1) *Z*-**11** (2) *Z*-**11** (**3**) *Z*-**11** (4)

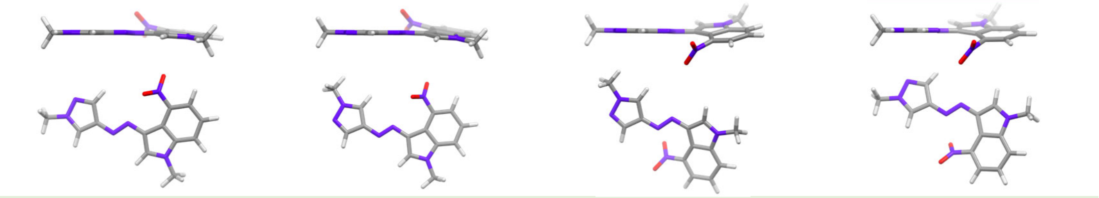

E-**11** (1) *E*-**11** (2) *E*-**11** (3) *E*-**11** (**4**)

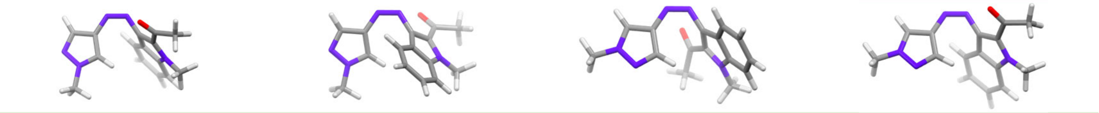

Z-**12** (1) *Z*-**12** (2) *Z*-**12** (3) *Z*-**12** (4)

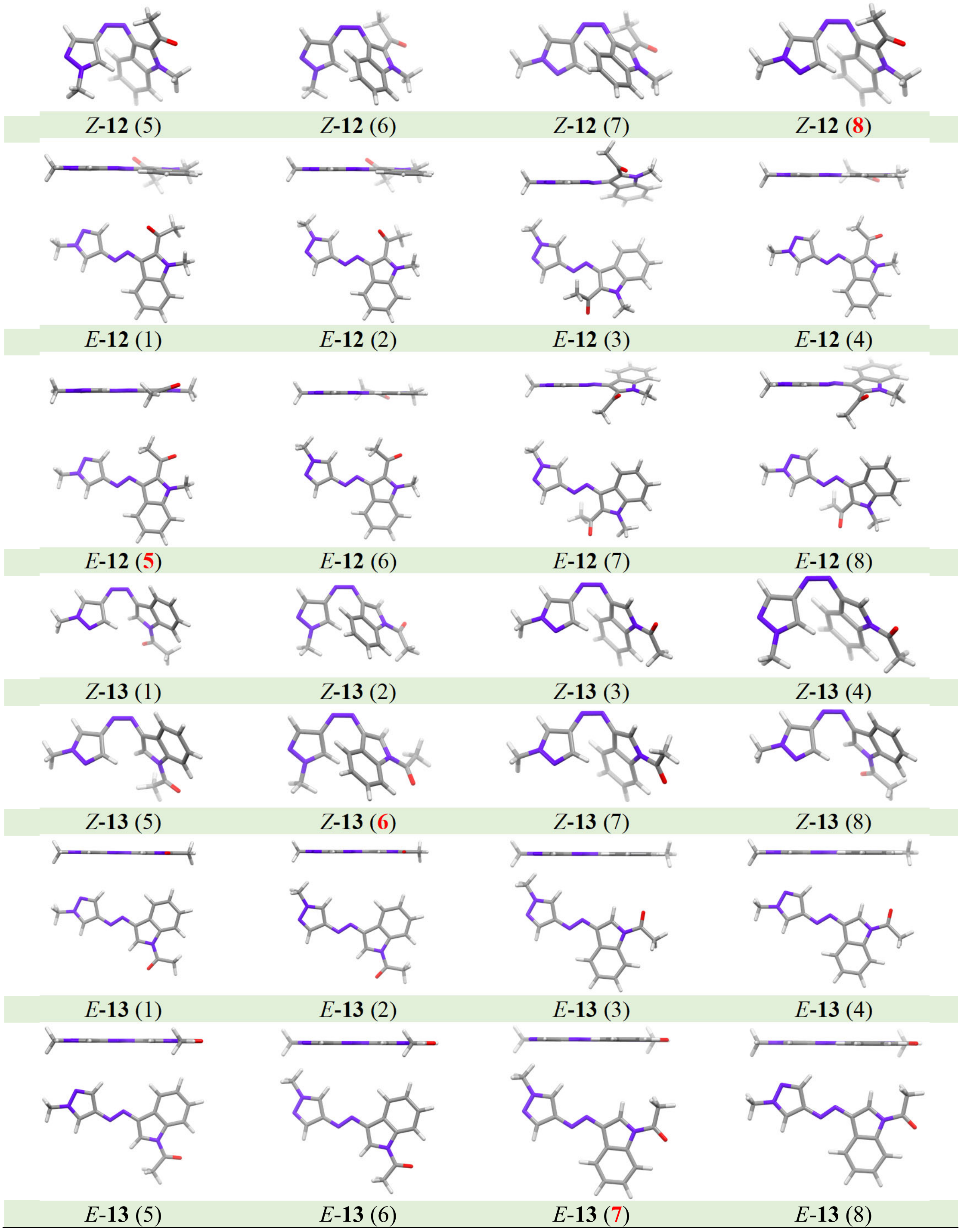

Figure S37. Optimized geometries of the conformation for **1-13**. (Exhibit four low-energy optimal conformations; atomic color: dark gray–carbon, light gray–hydrogen, red–oxygen, blue–nitrogen, yellowish green–fluorine)

Table S23. Relative $G_{solution}$ and Boltzmann distributions of **1**–**13** conformers calculated in MeCN (The detailed computational methods were described in section 1.2.).

Compound	Isomer	Conformer	Relative $G_{solution}$ (kcal/mol)	Population (%)
**1**	*E*-**1**	1	1.41	6.76
		2	2.97	0.49
		3	0.77	19.94
		**4**	**0.00**	**72.81**
	Z-**1**	**1**	**0.01**	**31.81**
		**2**	**0.00**	**32.18**
		3	0.62	11.24
		4	0.15	24.77
**2**	*E*-**2**	1	1.68	3.79
		**2**	**0.00**	**65.36**
		3	0.49	28.59
		4	1.99	2.27
	Z-**2**	1	0.87	9.56
		2	0.87	9.50
		**3**	**0.00**	**41.22**
		**4**	**0.02**	**39.72**
**3**	*E*-**3**	1	2.08	2.98
		**2**	**0.00**	**97.02**
		3	4.16	0.09
		4	6.11	0.00(3)
	Z-**3**	**1**	**0.00**	**35.45**
		**2**	**0.00(4)**	**35.19**
		3	0.13	28.50
		4	2.19	0.87
**4**	*E*-**4**	1	4.19	0.08
		2	1.57	6.28
		**3**	**0.00**	**89.47**
		4	1.81	4.18
	Z-**4**	1	0.95	12.26
		2	0.80	15.77

(Table S23. continued)				
**4**	*Z*-**4**	**3**	**0.00**	**60.89**
		4	1.00	11.08
**5**	*E*-**5**	1	2.61	0.53
		2	0.86	10.11
		**3**	**0.00**	**43.46**
		4	1.98	1.54
		5	4.08	0.04
		6	2.39	0.76
		7	0.00(1)	43.42
		8	3.42	0.13
	Z-**5**	**1**	**0.00**	**27.94**
		2	0.01	27.54
		3	0.56	10.90
		4	0.55	10.95
		5	0.58	10.54
		6	0.83	6.88
		7	1.40	2.62
		8	1.40	2.62
**6**	*E*-**6**	**1**	**0.00**	**86.93**
		2	1.22	10.99
		3	2.96	0.58
		4	2.41	1.49
	Z-**6**	1	1.49	4.45
		2	2.12	1.52
		**3**	**0.00**	**54.98**
		4	0.20	39.04
**7**	*E*-**7**	1	2.07	2.48
		**2**	**0.00**	**82.52**
		3	2.75	0.79
		4	1.04	14.21
	Z-**7**	1	1.33	6.33
		2	0.45	27.89
		**3**	**0.00**	**59.51**

(Table S23. continued)				
**7**	*Z*-**7**	4	1.33	6.28
**8**	*E*-**8**	1	2.69	0.96
		**2**	**0.00**	**89.54**
		3	1.34	9.26
		4	3.50	0.25
	Z-**8**	1	1.04	8.03
		2	0.13	37.26
		**3**	**0.00**	**46.80**
		4	1.05	7.91
**9**	*E*-**9**	**1**	**0.00**	**62.18**
		2	0.43	30.05
		3	1.68	3.64
		4	1.61	4.12
	Z-**9**	1	1.19	6.62
		2	1.24	6.15
		3	0.17	37.44
		**4**	**0.00**	**49.79**
**10**	*E*-**10**	1	0.45	18.50
		**2**	**0.00**	**39.26**
		3	0.23	26.83
		4	0.55	15.41
	Z-**10**	1	1.43	5.58
		2	0.45	28.88
		**3**	**0.00**	**62.20**
		4	1.73	3.34
**11**	*E*-**11**	1	0.47	22.77
		2	1.53	3.80
		3	0.45	2345
		**4**	**0.00**	**49.98**
	Z-**11**	1	0.72	14.84
		2	0.27	32.06
		**3**	**0.00**	**50.41**
		4	1.73	2.70

(Table S23. continued)				
**12**	*E*-**12**	1	2.16	1.32
		2	3.42	0.16
		3	2.53	0.70
		4	2.27	1.09
		**5**	**0.00**	**50.54**
		6	0.09	43.20
		7	2.33	0.99
		8	1.91	1.99
	Z-**12**	1	3.07	0.17
		2	3.13	0.15
		3	4.00	0.04
		4	2.97	0.20
		5	0.27	19.40
		6	0.26	19.56
		**7**	**0.01**	**30.05**
		**8**	**0.00**	**30.43**
**13**	*E*-**13**	1	3.67	0.11
		2	2.72	0.53
		3	2.07	1.59
		4	1.87	2.22
		5	1.01	9.49
		6	1.52	4.03
		**7**	**0.00**	**52.08**
		8	0.33	29.95
	Z-**13**	1	4.06	0.05
		2	2.01	1.60
		3	2.04	1.52
		4	2.02	1.60
		5	0.84	11.66
		**6**	**0.00**	**48.05**
		7	0.18	35.48
		8	4.14	0.04

Conformation	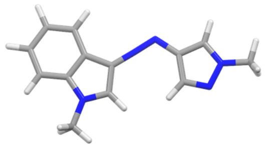	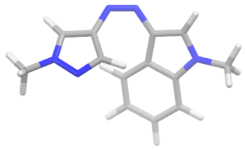
Gibbs free energy (kcal/mol)	-487144.84	-487173.82
Compound	TS-**2**	*Z*-**2**
Conformation	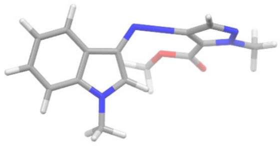	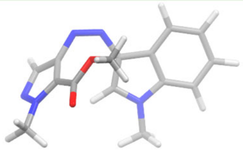
Gibbs free energy (kcal/mol)	-630079.57	-630108.83
Compound	TS-**5**	*Z*-**5**
Conformation	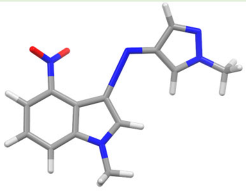	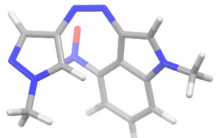
Gibbs free energy (kcal/mol)	-615451.99	-615483.41
Compound	TS-**11**	*Z*-**11**
Conformation	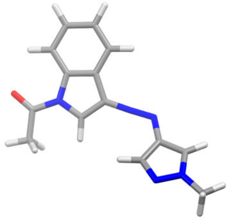	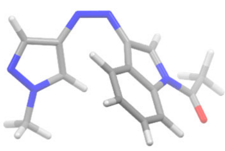
Gibbs free energy (kcal/mol)	-558246.89	-558278.35
Compound	TS-**13**	*Z*-**13**

Figure S38. Gibbs free energies for the optimal conformations of **2**, **5**, **11** and **13** in DMSO.

Table S24. Calculated excitation energies, excitation wavelengths (λ) and oscillator strengths (f) of **1-13** (the lowest energy structures) in solution (MeCN) at PBE0/6-311G** level of theory by TD-DFT.

Conformation		$S_0 \rightarrow S_1$ Excitation (n-π*)			$S_0 \rightarrow S_2$ Excitation (π-π*)		
		Energy (eV)	λ (nm)	*f*	Energy (eV)	λ (nm)	*f*
**1**	*E* (4)	3.0656	404.44	0.0000	3.4543	358.93	0.9437
	Z (2)	2.9793	416.16	0.0139	3.7707	328.81	0.2493
**2**	*E* (2)	3.1009	399.84	0.0000	3.4686	357.45	0.9378
	Z (3)	2.7567	449.75	0.1129	3.8429	322.63	0.2284

(Table S24. continued)							
**3**	*E* (2)	3.0363	408.33	0.0000	3.3075	374.86	0.9232
	Z (1)	2.9256	423.79	0.0312	3.6725	337.61	0.2670
**4**	*E* (3)	3.0150	411.23	0.0007	3.1694	391.19	1.0330
	Z (3)	2.6607	465.98	0.1229	3.7058	334.57	0.1674
**5**	*E* (3)	2.8766	431.01	0.0022	3.2343	383.35	0.8703
	Z (1)	2.9656	418.07	0.0294	3.6946	335.58	0.2050
**6**	*E* (1)	2.9412	421.54	0.0000	3.1021	399.67	0.6157
	Z (3)	2.7077	457.89	0.0984	3.4975	354.49	0.0624
**7**	*E* (2)	3.1295	396.17	0.0000	3.3623	368.74	0.7651
	Z (3)	2.7725	447.19	0.1297	3.6013	344.28	0.1048
**8**	*E* (2)	3.1140	398.15	0.0000	3.3522	369.85	0.8507
	Z (3)	2.7577	449.59	0.1286	3.6052	343.91	0.1263
**9**	*E* (1)	3.1032	399.54	0.0000	3.3320	372.10	0.8017
	Z (4)	2.7395	452.57	0.1295	3.5714	347.16	0.1322
**10**	*E* (2)	3.0772	402.92	0.0000	3.4809	356.18	0.9277
	Z (3)	2.7383	452.77	0.0991	3.6911	335.90	0.1141
**11** [a]	*E* (4)	3.0341	408.63	0.0341	3.1093	398.76	0.0487
	Z (3)	2.7417	452.22	0.0619	2.8658	432.64	0.0506
**12**	*E* (5)	2.7372	452.96	0.0006	3.0320	408.92	0.6563
	Z (8)	2.7297	454.20	0.0784	3.5606	348.21	0.0936
**13**	*E* (7)	2.9844	415.44	0.0000	3.5033	353.90	0.9354
	Z (6)	2.8175	440.05	0.0674	3.7714	328.75	0.0731

[a] The $S_0 \rightarrow S_1$ excitation of **11** is CT, $S_0 \rightarrow S_2$ excitation is n-π*.

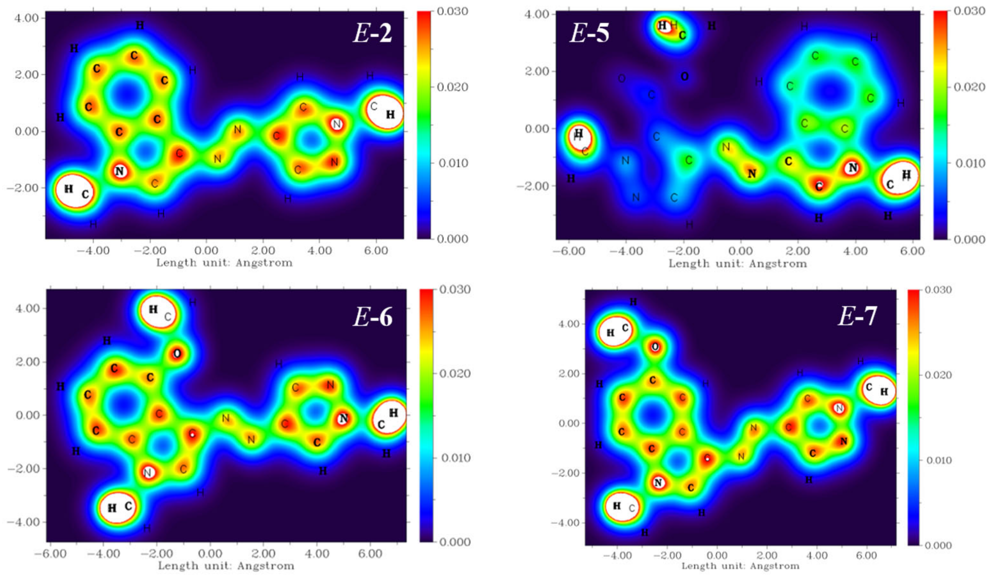

Figure S39. π-Electron density profile of *E*-**2**, **5-7** (Regions with a higher electron cloud density are represented by redder colors, while regions with a lower electron cloud density are depicted in blue).

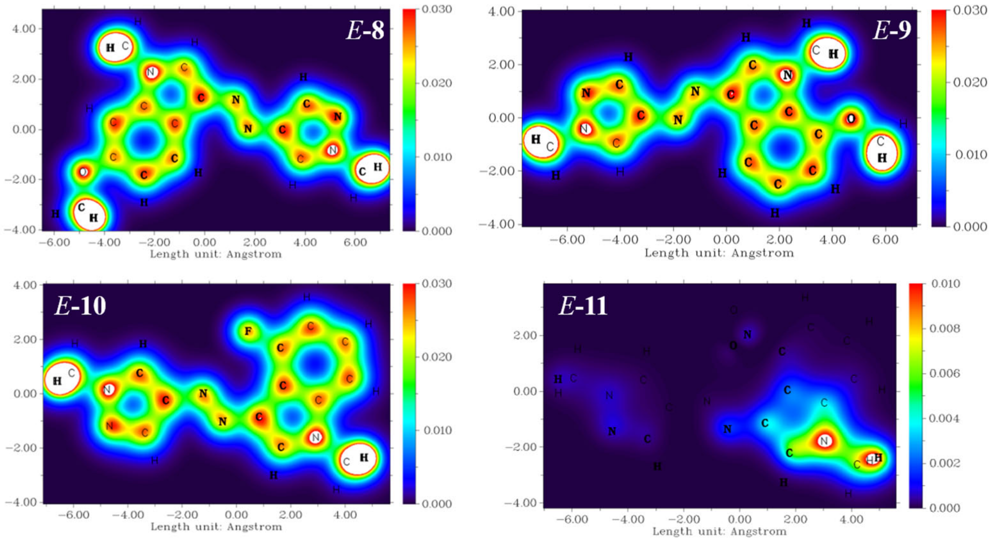

Figure S40. π-Electron density profile of *E*-**8-11** (Regions with a higher electron cloud density are represented by redder colors, while regions with a lower electron cloud density are depicted in blue).

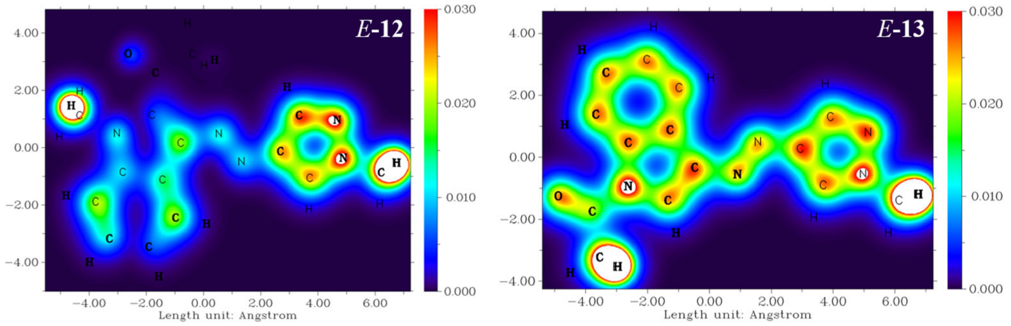

Figure S41. π-Electron density profile of *E*-**12** and **13** (Regions with a higher electron cloud density are represented by redder colors, while regions with a lower electron cloud density are depicted in blue).

Table S25. π-Electron population of N=N fragment (*E*-**2**, **5-13**) (The numerical values represent the proportion of the π-electron cloud contribution on the N=N fragment relative to the total electron cloud distribution).

Compound	*E*-**2**	*E*-**5**	*E*-**6**	*E*-**7**	*E*-**8**
Population	2.13	1.74	2.11	2.14	2.13
Compound	*E*-**9**	*E*-**10**	*E*-**11**	*E*-**12**	*E*-**13**
Population	2.13	2.12	0.03	1.38	2.10

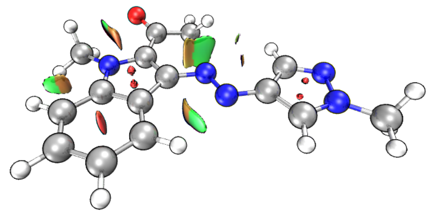

Figure S42. NCI surfaces of *E*–**12**.

a) FMO analysis

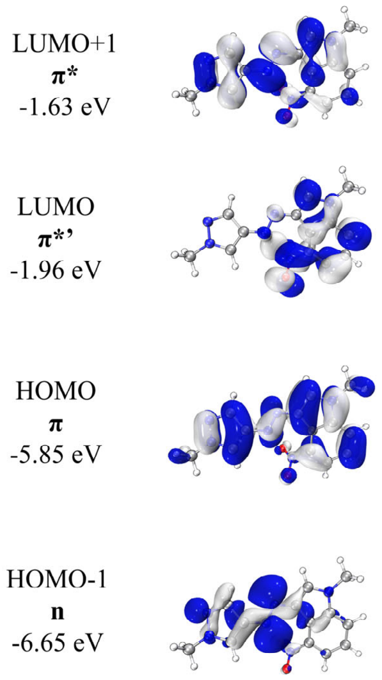

b) NTO analysis

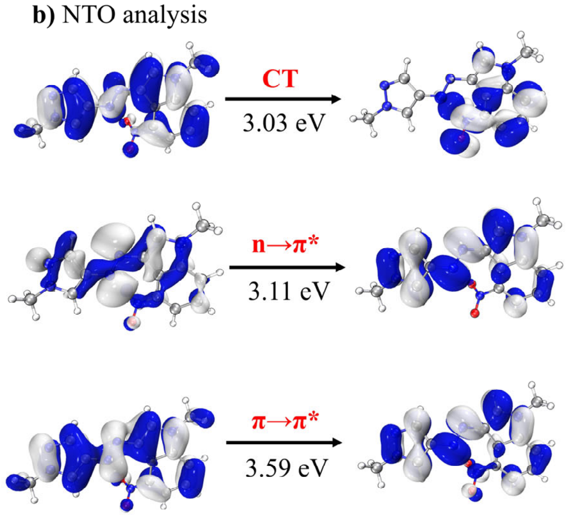

c) Fragment contribution of orbitals

$S_0 \rightarrow S_1$	Indole (%)	N=N (%)	Pyrazole (%)
LUMO	96.76	2.20	1.04
HOMO	57.65	14.55	27.80

Figure S43. a) FMOs and b) NTOs for the predominant conformers of *E*–**11** (isosurface value = 0.02). c) HOMO and LUMO electron contributions at each group in *E*–**11**.

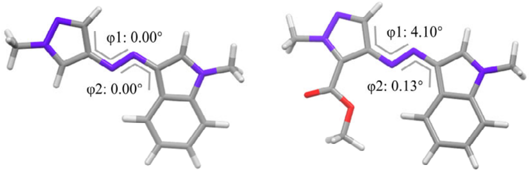

Figure S44. The dihedral angle of *E*–**2** and *E*–**5** in MeCN (the lowest energy configuration).

Table S26. Cartesian coordinates for the lowest energy configuration of *E*-**1** in MeCN.

E-**1** (MeCN)			
C	1.79467946	-1.84808922	-0.00001333
C	0.89885931	-0.78719753	-0.00002901
C	1.68808848	0.42248641	-0.00002962
C	3.03840551	-0.00168865	-0.00001598
C	1.40820909	1.79322626	-0.00003358
C	2.46806142	2.68848796	-0.00001508
C	3.79848462	2.24500859	0.00000797
C	4.10438317	0.89180518	0.00001116
H	0.38271391	2.13897969	-0.00005636
H	2.26513616	3.75454102	-0.00002033
H	4.60408043	2.97188389	0.00002358
H	5.13194985	0.54525247	0.00004361
N	3.06206329	-1.38585869	-0.00002141
C	4.26215631	-2.19228374	0.00008891
H	4.85906113	-1.98208091	0.89083567
H	3.97644871	-3.24307913	-0.00043765
H	4.85966072	-1.98136592	-0.89008111
H	1.58442254	-2.90812975	-0.00000769
N	-0.45108922	-1.00747942	-0.00004114
N	-1.16970271	0.03133396	-0.00001093
C	-2.53184862	-0.23839646	-0.00001375
C	-3.24777758	-1.45833477	-0.00013259
N	-4.58138162	0.28526688	0.00009515
C	-4.56634384	-1.06807572	0.00006181
H	-2.85022563	-2.46001461	-0.00021157
H	-5.48486795	-1.63613444	0.00004927
N	-3.35894202	0.81020031	0.00006430
C	-5.73841915	1.14922506	-0.00000418
H	-5.73197901	1.77888694	-0.89191550
H	-6.63397047	0.52937392	0.00121811
H	-5.73069358	1.78060531	0.89066187

Table S27. Cartesian coordinates for the lowest energy configuration of *Z*-**1** in MeCN.

Z-**1** (MeCN)			
C	0.02957855	0.57470743	0.20852677
C	0.49243437	-0.73274268	0.01585798
C	1.92971467	-0.63485558	-0.08021271
C	2.25642625	0.72787305	0.06293953
C	2.95250840	-1.56784613	-0.26922207
C	4.26069332	-1.11318894	-0.31175378
C	4.56553407	0.25073530	-0.16853196
C	3.56792962	1.19435989	0.02149615
H	2.72084994	-2.62163712	-0.38034734
H	5.06880456	-1.82245517	-0.45858061
H	5.60110765	0.57189733	-0.20713171
H	3.79979974	2.24778992	0.13337316
N	1.07474219	1.42551462	0.23777408
C	0.98347685	2.85811769	0.41909995
H	1.41274169	3.37341467	-0.44310647
H	-0.06568522	3.13210174	0.51680102
H	1.52137076	3.15744203	1.32162223
N	-0.09542088	-1.97906127	-0.04993577
N	-1.32212438	-2.23059922	0.04519963
H	-0.96656792	0.96853863	0.32475942
C	-2.25717332	-1.17858048	0.18924731
C	-2.91153948	-0.77871282	1.36850948
N	-3.55439688	0.39291031	-0.37353902
C	-3.73902374	0.24668266	0.95275846
H	-2.78387051	-1.16891868	2.36607501
H	-4.43713742	0.86841394	1.49358442
N	-2.65709340	-0.47505924	-0.86495949
C	-4.24989278	1.28799103	-1.26840528
H	-4.91652854	0.72366163	-1.92457224
H	-4.83367533	1.98996862	-0.67427815
H	-3.5251567	1.83660357	-1.87183380

Table S28. Cartesian coordinates for the lowest energy configuration of *E*-**2** in MeCN.

E-**2** (MeCN)			
C	-1.79431725	-1.84659997	-0.00010781
C	-0.91530091	-0.77419146	-0.00018242
C	-1.72269543	0.42324156	0.00004454
C	-3.06770383	-0.02083908	0.00020986
C	-1.46521249	1.79895550	0.00008777
C	-2.53777456	2.67841089	0.00035602
C	-3.86185732	2.21509448	0.00059006
C	-4.14681910	0.85772892	0.00053242
H	-0.44544973	2.16129726	-0.00006769
H	-2.35060748	3.74740537	0.00040210
H	-4.67825754	2.92985838	0.00083357
H	-5.16910249	0.49568626	0.00075007
N	-3.07158306	-1.40288903	0.00002216
C	-4.25894441	-2.22738350	0.00080313
H	-4.86188378	-2.02529995	-0.88789738
H	-3.95718371	-3.27369058	-0.00218909
H	-4.85821720	-2.02924079	0.89289557
H	-1.56903253	-2.90349433	-0.00024104
N	0.44194294	-0.97482017	-0.00048773
N	1.14329251	0.07579058	-0.00050951
C	2.50962573	-0.15716715	-0.00037473
C	3.27192231	-1.35071434	-0.00028438
N	4.65984796	0.25896844	-0.00037797
H	2.92212457	-2.37248543	-0.00031332
C	5.95935510	0.88929608	-0.00001887
H	5.82213764	1.96972832	-0.00178829
H	6.51418776	0.59322947	0.89233248
H	6.51581175	0.59052019	-0.89043296
N	4.56796801	-1.08180569	-0.00034444
C	3.45911981	0.85727999	-0.00033049
H	3.34880815	1.93110531	-0.00035206

Table S29. Cartesian coordinates for the lowest energy configuration of *E*-**2** in DMSO.

E-**2** (DMSO)			
C	-1.79426910	-1.84669282	0.00010109
C	-0.91508564	-0.77437102	0.00016928
C	-1.72219781	0.42309369	0.00018203
C	-3.06716885	-0.02091013	0.00015503
C	-1.46466577	1.79867761	0.00021025
C	-2.53722599	2.67796057	0.00026346
C	-3.86113890	2.21471855	0.00029563
C	-4.14620406	0.85753286	0.00025488
H	-0.44501832	2.16135079	0.00020983
H	-2.3501406	3.74697127	0.00029545
H	-4.67751868	2.92951770	0.00036714
H	-5.16856366	0.49566695	0.00031339
N	-3.07111465	-1.40275058	0.00003972
C	-4.25855473	-2.22647276	0.00055540
H	-4.86096599	-2.02444582	-0.88852571
H	-3.95747289	-3.27299252	-0.00185347
H	-4.85816649	-2.02765978	0.89227544
H	-1.56925842	-2.90366066	0.00005189
N	0.44193819	-0.97472207	0.00015601
N	1.14296299	0.07617545	0.00022716
C	2.50912888	-0.15667724	-0.00012922
C	3.27098649	-1.35041163	-0.00062356
N	4.65922358	0.25881988	-0.00057586
H	2.92092622	-2.37214293	-0.00081516
C	5.95857948	0.88860034	-0.00036902
H	5.82190025	1.96910270	-0.00169650
H	6.51365334	0.59211246	0.89171830
H	6.5147562	0.59006606	-0.89106671
N	4.56701864	-1.08183156	-0.00083025
C	3.45891357	0.85744401	-0.00014967
H	3.34908212	1.93132366	0.00011519

Table S30. Cartesian coordinates for the lowest energy configuration of *Z*-**2** in MeCN.

Z-**2** (MeCN)			
C	-1.97047997	-1.79548033	0.35554337
C	-0.80787951	-1.40900165	-0.29161055
C	-0.94229080	0.00631643	-0.55497101
C	-2.18634995	0.38762132	0.00887857
C	-0.20112531	0.97194391	-1.24957244
C	-0.68769867	2.26775105	-1.32485345
C	-1.90649403	2.62683232	-0.73014198
C	-2.67646502	1.68889525	-0.06066738
H	0.73658866	0.71583171	-1.72710856
H	-0.11419113	3.02027374	-1.85623529
H	-2.25737636	3.65078805	-0.80435988
H	-3.62967505	1.95423264	0.38366539
N	-2.77279068	-0.72697239	0.57021681
C	-4.04641226	-0.74373400	1.25351205
H	-4.84183497	-0.40708230	0.58400456
H	-4.25726213	-1.76330601	1.57281261
H	-4.01731984	-0.09253002	2.13062087
N	0.11336545	-2.38288290	-0.67250734
N	1.35398281	-2.19922666	-0.76739971
H	-2.25157024	-2.78217472	0.69538182
C	2.02416330	-1.07388368	-0.29128425
C	3.32429918	-0.73511018	-0.73017494
N	2.87040274	0.58914508	0.85707393
H	3.89236546	-1.19962030	-1.52466769
C	3.08577232	1.67860059	1.78075130
H	2.25448844	1.70815572	2.48364995
H	4.01976329	1.51951874	2.32184075
H	3.13607153	2.62314482	1.23486265
N	3.81762023	0.28378070	-0.04627353
C	1.77621328	-0.17751018	0.75096992
H	0.94037312	-0.05358392	1.41948642

Table S31. Cartesian coordinates for the lowest energy configuration of *Z*-**2** in DMSO.

Z-**2** (DMSO)			
C	-2.12607415	-1.77248763	0.09715407
C	-0.93034427	-1.31120997	-0.42694732
C	-1.04072547	0.12864350	-0.48536236
C	-2.31022695	0.44127500	0.06341440
C	-0.25759054	1.17252725	-0.99533091
C	-0.73129957	2.47172696	-0.90908317
C	-1.97737906	2.75869949	-0.33143808
C	-2.78753581	1.74620479	0.15665584
H	0.70343833	0.97288592	-1.45359822
H	-0.12569395	3.28501741	-1.29560208
H	-2.31692757	3.78765761	-0.27527715
H	-3.76041257	1.95945284	0.58616915
N	-2.93196242	-0.73467580	0.42459143
C	-4.23773523	-0.83339232	1.03553824
H	-4.99279350	-0.37173174	0.39442575
H	-4.48113048	-1.88646304	1.17022651
H	-4.24300964	-0.33696877	2.00912691
N	0.00676472	-2.22270082	-0.91118301
N	1.25008497	-2.03657472	-0.90677735
H	-2.43136949	-2.79400264	0.27374863
C	1.88498691	-1.01257117	-0.20337401
C	1.58823184	-0.30843526	0.99390450
N	3.55641882	0.26092395	0.42581853
H	0.69867798	-0.34510982	1.60387040
C	4.80037184	0.98915682	0.51217275
H	5.45504309	0.65909368	-0.29303451
H	4.61174359	2.06015029	0.41420344
H	5.27488324	0.78963495	1.47448753
N	2.61057201	0.44545177	1.36285886
C	3.18167699	-0.61518767	-0.51437904
H	3.82626499	-0.89628603	-1.33345505

Table S32. Cartesian coordinates for the lowest energy configuration of *E*-**3** in MeCN.

E-**3** (MeCN)			
C	-1.62835668	-1.92092671	-0.00001163
C	-0.72099685	-0.86696681	-0.00001135
C	-1.49938621	0.35104623	-0.00000044
C	-2.85262018	-0.06171299	0.00002197
C	-1.20867432	1.71889247	-0.00000808
C	-2.26137229	2.62283460	0.00002865
C	-3.59486022	2.19027804	0.00007536
C	-3.91146120	0.83939420	0.00007642
H	-0.18100313	2.05846182	-0.00003438
H	-2.05012006	3.68722848	0.00002832
H	-4.39452486	2.92359885	0.00011632
H	-4.94158936	0.50065765	0.00012455
N	-2.88861115	-1.44713420	-0.00000781
C	-4.09653524	-2.24214722	0.00025156
H	-4.69140776	-2.02407043	-0.88978974
H	-3.82065587	-3.29551611	-0.00063970
H	-4.69030767	-2.02526094	0.89132774
H	-1.42693332	-2.98270517	-0.0000268
N	0.62062470	-1.10537475	-0.00003843
N	1.35762102	-0.07491897	-0.00002359
C	2.70571800	-0.36451082	-0.00006275
C	3.45379774	-1.53989577	-0.00010226
N	3.59764602	0.66196822	-0.00005038
C	4.77860695	-1.10025151	-0.00011936
H	3.07169412	-2.54796903	-0.00011527
H	5.68517482	-1.69006434	-0.00014960
N	4.85923853	0.23272449	-0.00008255
C	3.31662974	2.07874444	-0.00003018
H	2.23741663	2.21851779	0.00011809
H	3.74696822	2.53880475	0.89158618
H	3.74671905	2.53879426	-0.89177371

Table S33. Cartesian coordinates for the lowest energy configuration of *Z*-**3** in MeCN.

Z-**3** (MeCN)			
C	0.06818982	0.74013745	-0.27595144
C	-0.28015600	-0.61982437	-0.27458946
C	-1.71734215	-0.66146199	-0.13234876
C	-2.15643381	0.67358413	-0.05268545
C	-2.65259630	-1.69619052	-0.06412301
C	-3.99056723	-1.36573153	0.08168318
C	-4.40831161	-0.02728012	0.16230640
C	-3.49746105	1.01587462	0.09616480
H	-2.33366573	-2.73104708	-0.12468234
H	-4.73318554	-2.15511144	0.13595645
H	-5.46387908	0.19494823	0.27785790
H	-3.81622103	2.05040239	0.15733797
N	-1.04102895	1.48953945	-0.14920822
C	-1.07407783	2.93611719	-0.11766600
H	-1.52683718	3.27774015	0.81568446
H	-0.05367527	3.31026685	-0.18255504
H	-1.65556075	3.31618579	-0.96063070
N	0.41452800	-1.79199289	-0.43623020
N	1.66636812	-1.90817836	-0.54386019
H	1.02257691	1.23394286	-0.37121991
C	2.49679328	-0.78223239	-0.41434650
C	3.25692957	-0.12451587	-1.36582942
N	2.74213438	-0.17987937	0.77599200
C	3.90785710	0.88691279	-0.64112937
H	3.30754759	-0.34582693	-2.42051287
H	4.59701669	1.63682818	-1.00529726
N	3.59843329	0.84586983	0.65042940
C	2.18669677	-0.50731322	2.06754201
H	2.9756954	-0.43258261	2.81587011
H	1.37302233	0.17685543	2.32244853
H	1.80699834	-1.52856955	2.04572130

Table S34. Cartesian coordinates for the lowest energy configuration of *E*-**4** in MeCN.

E-**4** (MeCN)			
N	-0.23707310	-0.56473004	0.00241568
N	0.27232592	0.59457886	-0.00316847
C	1.65231001	0.59710503	-0.00308545
C	2.62596481	-0.45677188	-0.00817342
C	3.85927004	0.23047777	-0.01072531
C	2.61666708	-1.85905264	-0.01341753
C	5.09020186	-0.43419479	-0.00952009
C	3.83006075	-2.51987217	-0.01533141
H	1.67994728	-2.40097228	-0.01409249
C	5.05072335	-1.81438569	-0.01268827
H	6.02805633	0.10944474	-0.00520099
H	3.84935604	-3.60463655	-0.01783969
H	5.98197938	-2.37140865	-0.01180247
N	2.25032562	1.78266982	-0.00215412
N	3.56542102	1.56302742	-0.01655827
C	4.49321838	2.66446815	0.05868577
H	5.36614216	2.44847774	-0.55898183
H	3.99405064	3.55478109	-0.32067737
H	4.81301715	2.83548305	1.09081953
C	-1.60412095	-0.59855437	0.00371757
C	-2.60241053	0.44577038	-0.00399750
C	-2.29013864	-1.80592316	0.01442146
C	-3.85202412	-0.21898014	0.00292015
C	-2.57877776	1.84456623	-0.01693441
H	-1.89014889	-2.80984432	0.02257437
C	-5.06398269	0.46386868	-0.00199631
C	-3.78483831	2.53015398	-0.02220783
H	-1.63359500	2.37188528	-0.02250056
C	-5.01144566	1.85015964	-0.01487511
H	-6.01060422	-0.06527153	0.00363500
H	-3.78100856	3.61531159	-0.03230534
H	-5.93645929	2.41724336	-0.01922304
N	-3.62121926	-1.58371209	0.01418752
C	-4.65270917	-2.59657161	0.02587105
H	-4.17983945	-3.57742420	0.03100906
H	-5.27490359	-2.49327330	0.91809118
H	-5.28226206	-2.50621442	-0.86254189

Table S35. Cartesian coordinates for the lowest energy configuration of *Z*-**4** in MeCN.

Z-**4** (MeCN)			
C	2.64679431	1.55606735	0.45137429
C	1.41035259	1.47517604	-0.17809023
C	1.32357534	0.13705390	-0.73301953
C	2.52146240	-0.51279998	-0.34462185
C	0.43532576	-0.55853116	-1.56362312
C	0.73075840	-1.86279877	-1.92631113
C	1.90506375	-2.49536447	-1.49389475
C	2.82291888	-1.82334823	-0.70317242
H	-0.47069322	-0.09174780	-1.92882942
H	0.03763941	-2.40524380	-2.56098270
H	2.10440171	-3.51913377	-1.79241170
H	3.74537190	-2.29500925	-0.38272498
N	3.28850127	0.37431885	0.38776743
C	4.57947644	0.07540012	0.96644935
H	5.28079268	-0.23072964	0.18669308
H	4.95714897	0.97151284	1.45638618
H	4.48804321	-0.72680350	1.70251076
N	0.63493543	2.62060138	-0.20662745
N	-0.61474125	2.65396990	-0.35602042
H	3.07716756	2.40311647	0.96633084
C	-1.40940140	1.50794553	-0.24273221
C	-1.42523985	0.49027635	0.76372280
C	-2.54276320	-0.30126909	0.42022357
C	-0.67052776	0.17087736	1.90214066
C	-2.91076709	-1.43378102	1.15771852
C	-1.03333404	-0.94125231	2.63299262
H	0.17885526	0.77664891	2.19572279
C	-2.13799747	-1.73700659	2.25918402
H	-3.76495130	-2.03930059	0.87647727
H	-0.46035189	-1.21565254	3.51231503
H	-2.38682003	-2.60707536	2.85798500
N	-2.42916511	1.35915398	-1.07428030
N	-3.10007812	0.26563396	-0.68403096
C	-4.20683262	-0.22921500	-1.46441420
H	-4.96151793	-0.65059296	-0.79866776
H	-4.63800021	0.60732463	-2.01248114
H	-3.87643837	-0.99764025	-2.16953976

Table S36. Cartesian coordinates for the lowest energy configuration of *E*-**5** in MeCN.

E-**5** (MeCN)			
N	0.39345074	-1.55674375	0.04491836
N	-0.46537185	-0.62751606	-0.00170895
C	-1.77260065	-1.08312892	-0.00102553
C	-2.28707271	-2.39456002	-0.03393159
N	-3.97820108	-1.10019002	-0.03946717
H	-1.74490543	-3.32765174	-0.04875345
C	-5.39158299	-0.77976487	-0.04523338
H	-5.66183953	-0.25853129	-0.96352582
H	-5.92223442	-1.72770901	0.01921501
H	-5.64246349	-0.15187706	0.80959381
N	-3.61190350	-2.38065871	-0.05271134
C	-2.90879426	-0.26221611	-0.00572234
C	-3.07499958	1.19652648	-0.00271161
O	-1.93909428	1.82927541	0.27258074
C	-2.01060657	3.25866333	0.26293953
O	-4.12398686	1.75921883	-0.22204140
H	-2.73570653	3.61246823	0.99830292
H	-1.01147647	3.60192340	0.52488679
H	-2.28792944	3.62254751	-0.72850721
C	1.69087477	-1.12798853	0.02899950
C	2.26426053	0.19595623	-0.03399719
C	2.75264792	-2.02213495	0.07638466
C	3.66609904	0.00740111	-0.01993802
C	1.75142078	1.49574569	-0.09985782
C	4.56355365	1.06895369	-0.06849839
C	2.64307007	2.55782935	-0.14917442
H	0.68013129	1.65322048	-0.11064345
C	4.02978190	2.34839918	-0.13318049
H	5.63548745	0.90478026	-0.05650302
H	2.26155975	3.57258918	-0.20123176
H	4.69924212	3.20142703	-0.17217157
H	2.72480797	-3.10115855	0.12946158
N	3.92319575	-1.35205759	0.04744896
C	5.24250640	-1.94246849	0.08103170
H	5.79243185	-1.58955011	0.95674440
H	5.13986400	-3.02514367	0.13494133
H	5.79813982	-1.67840460	-0.82199098

Table S37. Cartesian coordinates for the lowest energy configuration of *Z*-**5** in MeCN.

Z-**5** (MeCN)			
C	0.53681833	-1.07359973	0.06720215
C	0.95089505	-0.00263842	0.86725339
C	2.35098618	0.19259504	0.57679799
C	2.70438280	-0.76881776	-0.39066072
C	3.31908252	1.08826605	1.03839237
C	4.60204417	0.99559687	0.52352280
C	4.93395865	0.03101700	-0.44268592
C	3.99031352	-0.86712840	-0.91630482
H	3.06572499	1.83580717	1.78233766
H	5.36845271	1.68093871	0.87070920
H	5.94824597	-0.01190377	-0.82538371
H	4.24268610	-1.61248787	-1.66235337
N	1.57692669	-1.52057452	-0.66594697
C	1.52974578	-2.62537015	-1.59907055
H	2.22331590	-3.40997179	-1.28811506
H	0.51717642	-3.02525899	-1.61701542
H	1.80030095	-2.28244353	-2.60032115
N	0.34639153	0.78852744	1.81991389
N	-0.87422873	0.80080463	2.10644300
H	-0.41618643	-1.56846017	-0.02079655
C	-1.80209094	-0.01572599	1.42871517
C	-2.42486195	-1.15425385	1.95538373
N	-3.30681155	-0.84220824	0.04504450
H	-2.24542650	-1.63531981	2.90596691
C	-4.21204760	-1.10331566	-1.05674388
H	-3.65188815	-1.28417191	-1.97386433
H	-4.78388483	-1.98818465	-0.78404340
H	-4.88168392	-0.25617603	-1.20675968
N	-3.32284032	-1.64187174	1.10006145
C	-2.40618260	0.17246045	0.18455595
C	-2.16491213	1.16801334	-0.85900004
O	-1.26177511	2.06059876	-0.46065899
C	-0.90702794	3.06097013	-1.41964229
O	-2.70140305	1.18137126	-1.94328748
H	-1.77814755	3.66141582	-1.68864857
H	-0.15827911	3.67997608	-0.92927352
H	-0.48766771	2.59932406	-2.31577037

Table S38. Cartesian coordinates for the lowest energy configuration of *Z*-**5** in DMSO.

Z-**5** (DMSO)			
C	-0.53028757	-1.07496046	0.07647554
C	-0.94572536	-0.00006586	0.87072604
C	-2.34545129	0.19296815	0.57787882
C	-2.69738750	-0.77333929	-0.38508475
C	-3.31424312	1.09043603	1.03403273
C	-4.59654389	0.99454606	0.51848190
C	-4.92703363	0.02506853	-0.44307251
C	-3.98267664	-0.87492189	-0.91136193
H	-3.06204701	1.84197774	1.77437481
H	-5.36362291	1.68125077	0.86148853
H	-5.94096788	-0.02025402	-0.82644147
H	-4.23416856	-1.62414947	-1.65383234
N	-1.56952898	-1.52587499	-0.65526288
C	-1.52151857	-2.63523186	-1.58238986
H	-1.79310724	-2.29782424	-2.58523972
H	-0.50848995	-3.03403277	-1.59905348
H	-2.21394402	-3.41909104	-1.26695141
N	-0.34402918	0.79543826	1.82145941
N	0.87645754	0.81218248	2.10829232
H	0.42256712	-1.57085735	-0.00796795
C	1.80543040	-0.00354051	1.43160598
C	2.43305714	-1.13728992	1.96241616
N	3.30266863	-0.83717454	0.04474671
H	2.25979008	-1.61210452	2.91731417
C	4.20034625	-1.10689853	-1.06071950
H	4.85072592	-0.25070443	-1.23960035
H	4.79348313	-1.97285996	-0.77310481
H	3.63466610	-1.32295221	-1.9668340
N	3.32690324	-1.62906420	1.10550731
C	2.40143566	0.17714208	0.18243421
C	2.14922949	1.16167512	-0.86959345
O	1.26882884	2.07380581	-0.46352100
C	0.90128075	3.05912205	-1.43329068
O	2.66007757	1.15084717	-1.96585807
H	0.46206095	2.58519367	-2.31333559
H	0.16540541	3.69152816	-0.94041117
H	1.76962613	3.65084865	-1.72980484

Table S39. Cartesian coordinates for the lowest energy configuration of *E*-**6** in MeCN.

E-**6** (MeCN)			
C	-0.96903650	-2.01830476	-0.00047450
C	-0.63061009	-0.67142686	-0.00024647
C	-1.87100312	0.06062365	-0.00000456
C	-2.90281003	-0.90289322	-0.00003627
C	-2.22408624	1.42542208	0.00016899
C	-3.57345537	1.76179873	0.00034173
C	-4.56970876	0.77016334	0.00041582
C	-4.26084819	-0.57614011	0.00028774
H	-3.87393098	2.80199078	0.00048389
H	-5.60929350	1.08112763	0.00059777
N	-2.31553955	-2.15090876	-0.00035544
C	-3.03972795	-3.40236580	0.00005198
H	-3.67161191	-3.47499002	-0.88852878
H	-2.32223956	-4.22166940	-0.00308025
H	-3.66677431	-3.47745687	0.89187538
H	-0.32628914	-2.88510529	-0.00075713
N	0.62440371	-0.09832997	-0.00032906
N	1.58643246	-0.91465174	-0.00003914
C	2.83377431	-0.31292997	-0.00004108
C	3.23077000	1.04690242	-0.00030828
N	5.01413113	-0.10949417	0.00031984
H	2.60749212	1.92896584	-0.00062457
C	6.43831644	-0.34827061	0.00024485
H	6.61159961	-1.42341225	0.00264442
H	6.88725232	0.09197948	-0.89214603
H	6.88783394	0.09604424	0.89030182
N	4.54968645	1.15201520	-0.00022882
C	4.02978071	-1.02077084	0.00044645
H	4.22583617	-2.08226327	0.00083328
H	-5.03019433	-1.33931793	0.00040912
O	-1.21508999	2.31839450	0.00001539
C	-1.55180125	3.69465671	-0.00027616
H	-0.60443422	4.23307986	-0.00063268
H	-2.12300524	3.96709943	-0.89455726
H	-2.12264434	3.96756928	0.89406477

Table S40. Cartesian coordinates for the lowest energy configuration of *Z*-**6** in MeCN.

Z-**6** (MeCN)			
C	-2.07216806	-1.87083664	-0.42844852
C	-0.94938628	-1.23159314	-0.91042838
C	-1.06789086	0.14958944	-0.51897520
C	-2.25639295	0.24780893	0.24019504
C	-0.32537053	1.31848344	-0.77070974
C	-0.73107884	2.50755262	-0.17626613
C	-1.89757000	2.55815616	0.60631870
C	-2.68906745	1.44485658	0.81748358
H	-0.16332066	3.41623235	-0.33352683
H	-2.18706580	3.50886369	1.04221279
N	-2.83679873	-0.99753554	0.28576125
C	-4.06256866	-1.31823822	0.98036004
H	-4.89445125	-0.73317216	0.57972000
H	-4.27524425	-2.37746658	0.84234929
H	-3.96257778	-1.11138669	2.04897958
N	0.00608542	-1.90651579	-1.68523622
N	1.24063821	-1.80466099	-1.48398016
H	-2.35058401	-2.91096958	-0.51710683
C	1.81762693	-1.16524742	-0.39028875
C	3.19769887	-0.86047011	-0.37620992
N	2.45667350	-0.33914024	1.53536200
H	3.90571439	-0.99098408	-1.18332610
C	2.51529551	0.14622241	2.89459168
H	1.52074310	0.07553066	3.33264382
H	3.21599631	-0.45860581	3.47317131
H	2.84321522	1.18734882	2.89840352
N	3.57144807	-0.34520475	0.78168614
C	1.38050702	-0.80737397	0.88898455
H	0.42479768	-0.87180026	1.38103625
H	-3.6012501	1.49678920	1.40047715
O	0.72709014	1.19290860	-1.60107373
C	1.63747640	2.27398195	-1.69136572
H	2.44523266	1.93177156	-2.33770075
H	1.16969166	3.15756934	-2.13830131
H	2.04338994	2.53105968	-0.70704054

Table S41. Cartesian coordinates for the lowest energy configuration of *E*-**7** in MeCN.

E-**7** (MeCN)			
C	-1.03272203	-2.58994974	0.00004916
C	-0.35914969	-1.37525648	-0.00002989
C	-1.36854790	-0.34158070	-0.00000956
C	-2.61107574	-1.02119221	0.00004878
C	-1.35084033	1.05234120	-0.00002050
C	-2.56292509	1.73949016	0.00001391
C	-3.78784387	1.04908075	0.00004681
C	-3.81891031	-0.33978868	0.00006147
H	-0.41987272	1.60306188	-0.00005860
H	-4.76653993	-0.86730471	0.00007989
N	-2.36600920	-2.38523582	0.00009296
C	-3.38554770	-3.40854262	-0.00002532
H	-4.01282703	-3.32006602	-0.89060998
H	-2.90210396	-4.38438569	0.00072846
H	-4.01381756	-3.31920344	0.88976612
H	-0.61900138	-3.58834190	0.00006901
N	1.00943773	-1.32695993	-0.00007350
N	1.51166702	-0.16660933	-0.00019507
C	2.89840198	-0.15667845	-0.00007019
C	3.85469254	-1.20109209	0.00027675
N	4.94564211	0.62240758	-0.00025879
H	3.68523081	-2.26769830	0.00051976
C	6.11832539	1.46546627	0.00012462
H	5.79870815	2.50645950	-0.00301652
H	6.71498317	1.26937854	0.89308675
H	6.71836297	1.26505125	-0.88957380
N	5.08558267	-0.71367905	0.00020692
C	3.65928795	1.00596444	-0.00036701
H	3.36715784	2.04520697	-0.00069365
H	-4.72340146	1.59378923	0.00005775
O	-2.46810460	3.09258986	0.00000530
C	-3.66732317	3.84396136	0.00006156
H	-3.36442865	4.89091970	0.00006447
H	-4.26842966	3.64481677	-0.89424164
H	-4.26835809	3.64479394	0.89440850

Table S42. Cartesian coordinates for the lowest energy configuration of *Z*-**7** in MeCN.

Z-**7** (MeCN)			
C	-2.13544401	-2.26882514	0.14594274
C	-0.91643139	-1.73594897	0.54376452
C	-1.02031379	-0.30414781	0.35717969
C	-2.30684774	-0.08199248	-0.19624411
C	-0.21927854	0.79875755	0.65824635
C	-0.68641771	2.07749897	0.36811434
C	-1.95268254	2.27447426	-0.21020974
C	-2.77399939	1.19095663	-0.48983104
H	-2.30467482	3.27327347	-0.43482676
N	-2.94693836	-1.30073701	-0.32828899
C	-4.27464244	-1.48815477	-0.86580779
H	-4.31711955	-1.15083334	-1.90447959
H	-4.52177006	-2.54801958	-0.82400423
H	-5.00536537	-0.92691530	-0.27781767
N	0.02017110	-2.57392400	1.13855034
N	1.26572522	-2.39474756	1.11835967
H	-2.44727087	-3.30351510	0.15982892
C	1.90436291	-1.47362227	0.28640063
C	1.62591735	-0.98025486	-1.01520346
N	3.56110969	-0.26908376	-0.49909486
H	0.75528194	-1.14272422	-1.63218301
C	4.78378017	0.48159940	-0.66315903
H	5.44208598	0.26423824	0.17668473
H	5.26971238	0.18668219	-1.59453001
H	4.56549665	1.55137980	-0.68927158
N	2.63956155	-0.26478509	-1.47626405
C	3.18049848	-0.99396279	0.56183577
H	3.80817979	-1.12002103	1.43101979
H	-3.75897186	1.34454939	-0.91707569
O	0.15997254	3.09110758	0.67976585
H	0.76006043	0.69499084	1.10715115
C	-0.25472063	4.41471489	0.39951834
H	-1.16060950	4.68089567	0.95584802
H	0.56354477	5.05874387	0.72147636
H	-0.42844897	4.56480066	-0.67206530

Table S43. Cartesian coordinates for the lowest energy configuration of *E*-**8** in MeCN.

E-**8** (MeCN)			
C	-0.77782239	2.47718068	-0.00036529
C	-0.12455987	1.25614223	0.00003952
C	-1.15063006	0.23710887	-0.00004957
C	-2.38440247	0.93299624	-0.00047711
C	-1.17919084	-1.15763866	0.00024937
C	-2.39977391	-1.81969015	0.00019973
C	-3.61001098	-1.10282913	-0.00014586
C	-3.61370520	0.29065584	-0.00047869
H	-0.25518381	-1.72140403	0.00054066
H	-2.41027445	-2.90240151	0.00045092
H	-4.55377475	0.83021386	-0.00067945
N	-2.11980564	2.28918330	-0.00079600
C	-3.12371924	3.32812240	-0.00057227
H	-3.75076338	3.25153326	0.89137912
H	-2.62534668	4.29641631	-0.00426586
H	-3.75525867	3.24718407	-0.88892218
H	-0.35255576	3.47047759	-0.00047011
N	1.24381961	1.18952584	0.00035853
N	1.72820510	0.02169037	0.00029626
C	3.11432350	-0.00961142	0.00028824
C	4.08630118	1.02036678	0.00053843
N	5.14950384	-0.81951384	-0.00043943
H	3.93290982	2.08938762	0.00100101
C	6.30959915	-1.67984708	0.00084435
H	5.97499445	-2.71608918	-0.00867309
H	6.91604586	-1.48381136	-0.88543328
H	6.90548655	-1.49672080	0.89708723
N	5.30952898	0.51445559	0.00094370
C	3.85770135	-1.18361709	-0.00082868
H	3.55017224	-2.21836879	-0.00167930
O	-4.83016077	-1.68908966	-0.00017089
C	-4.89097047	-3.10422150	0.00029751
H	-4.42095694	-3.52716945	0.89490711
H	-5.95111247	-3.35684484	0.00022932
H	-4.42069770	-3.52778248	-0.89388457

Table S44. Cartesian coordinates for the lowest energy configuration of *Z*-**8** in MeCN.

Z-**8** (MeCN)			
C	-0.63055142	-2.88022046	0.13426550
C	0.24643158	-1.96241475	-0.41944889
C	-0.46274128	-0.69800164	-0.45652950
C	-1.72691506	-0.94716481	0.13616978
C	-0.22845682	0.58054203	-0.96936182
C	-1.19840912	1.56586547	-0.84716224
C	-2.42846037	1.29627547	-0.22150937
C	-2.70850175	0.02424047	0.27010595
N	-1.78618397	-2.27582672	0.50097619
C	-2.91264850	-2.91153369	1.14442914
H	-3.80404153	-2.83279242	0.51669710
H	-2.67803039	-3.96372207	1.29982803
H	-3.11401833	-2.44276025	2.11099346
N	1.46320458	-2.42538047	-0.90833831
N	2.51641416	-1.74240347	-1.00108029
H	-0.47221367	-3.93503830	0.30792824
C	2.69023230	-0.48936566	-0.41468528
C	3.66036935	0.43370872	-0.86506289
N	2.84770685	1.24740795	0.91228293
H	4.28656080	0.35664036	-1.74347864
C	2.65500282	2.22383318	1.95900606
H	2.00056783	1.79805423	2.71820126
H	3.61859697	2.47560672	2.40426897
H	2.19631242	3.12557147	1.54708618
N	3.73035214	1.49002258	-0.07061654
C	2.19059420	0.08864302	0.75336367
H	1.45978894	-0.24434149	1.47182039
H	-3.66953422	-0.18115297	0.72744428
H	0.70154245	0.81908463	-1.46992416
H	-0.99519168	2.55184349	-1.24569528
O	-3.40969405	2.21515581	-0.06447364
C	-3.18230838	3.52339213	-0.55803598
H	-4.08434252	4.08977086	-0.32737294
H	-2.32517597	3.99564002	-0.06554781
H	-3.02555037	3.52281315	-1.64229244

Table S45. Cartesian coordinates for the lowest energy configuration of *E*-**9** in MeCN.

E-**9** (MeCN)			
C	1.01518605	1.98897868	0.00043031
C	0.19552909	0.87104168	0.00063225
C	1.06421930	-0.28121110	0.00073206
C	2.38020010	0.23402788	0.00071008
C	0.85480875	-1.66897132	0.00070116
C	1.96366797	-2.49269571	0.00055015
C	3.27152968	-1.98030691	0.00024404
C	3.50222533	-0.61054357	0.00025285
H	-0.14927325	-2.07019885	0.00093360
H	1.83250026	-3.57009106	0.00056390
N	2.31348777	1.61889268	0.00074336
C	3.42670860	2.54859435	-0.00155136
H	4.03949645	2.40920718	-0.89277870
H	3.02026356	3.55934705	0.00330998
H	4.04746361	2.40377105	0.88312568
H	0.73339211	3.03212738	0.00019079
N	-1.16982272	1.00233468	0.00061799
N	-1.82315972	-0.07890996	0.00057473
C	-3.19854199	0.09682456	0.00027755
C	-4.00703409	1.25952669	0.00035544
N	-5.33091917	-0.40285997	-0.00011974
H	-3.69760897	2.29420133	0.00089939
C	-6.60527091	-1.08222254	-0.00167817
H	-6.42724134	-2.15666212	0.00250451
H	-7.17435156	-0.80300440	0.88710673
H	-7.16938508	-0.80912415	-0.89557485
N	-5.29158046	0.94022923	0.00044284
C	-4.10756323	-0.95413373	-0.00041556
H	-3.95608026	-2.02293679	-0.00083938
H	4.10881646	-2.66698284	0.00021989
O	4.71991922	-0.02821513	0.00014359
C	5.85533467	-0.87824137	-0.00141512
H	5.87892587	-1.50822843	-0.89687828
H	6.72151946	-0.21755091	-0.00148762
H	5.88017319	-1.50996547	0.89278744

Table S46. Cartesian coordinates for the lowest energy configuration of *Z*-**9** in MeCN.

Z-**9** (MeCN)			
C	1.13125346	2.37462075	0.13836392
C	0.06726782	1.6996052	-0.43198506
C	0.4995666	0.33614574	-0.61781026
C	1.81057464	0.27296315	-0.09093706
C	-0.06031712	-0.79813732	-1.2296352
C	0.68665212	-1.95771979	-1.26181475
C	1.97731387	-2.02859712	-0.7091234
C	2.56097445	-0.91546848	-0.12076928
N	2.15847691	1.52501496	0.37911925
C	3.40032641	1.9101525	1.02116356
H	4.24763582	1.72015234	0.36182287
H	3.34740625	2.97557222	1.24253283
H	3.54143676	1.35584084	1.94998414
N	-1.06854218	2.39484344	-0.84983728
N	-2.23554203	1.92875676	-0.81230147
H	1.19897381	3.41776645	0.41205898
C	-2.59615108	0.76414058	-0.13539343
C	-2.07428298	0.05808404	0.98252375
N	-3.94521431	-0.839065	0.51543431
H	-1.1586922	0.22416709	1.52860608
C	-5.02169426	-1.79406666	0.63719491
H	-5.80067558	-1.53455252	-0.07796768
H	-4.65291225	-2.80024168	0.42768198
H	-5.42722461	-1.75867873	1.64956532
N	-2.9052	-0.89618981	1.36633924
C	-3.81828085	0.14453786	-0.38230907
H	-4.57023882	0.3475974	-1.1297833
H	-1.05033101	-0.7649496	-1.66643718
H	0.27354783	-2.84902598	-1.72302228
H	2.52138818	-2.96362507	-0.75484172
O	3.79775371	-0.87973958	0.41913803
C	4.56648718	-2.07079623	0.38178849
H	4.74971656	-2.39339219	-0.64856363
H	5.51515615	-1.82760573	0.85906745
H	4.07679308	-2.87741598	0.93737088

Table S47. Cartesian coordinates for the lowest energy configuration of *E*-**10** in MeCN.

E-**10** (MeCN)			
N	-0.51229962	-1.02751102	0.00026778
N	-1.22467268	0.01116192	0.00031716
C	-2.58744957	-0.24032237	0.00010847
C	-3.33816427	-1.44129513	-0.00013021
N	-4.74131356	0.15558510	-0.00007617
H	-2.97906591	-2.45986494	-0.00020914
C	-6.04710311	0.77278907	-0.00004921
H	-5.92072860	1.85451055	-0.00010580
H	-6.59968322	0.46967320	-0.89140965
H	-6.59964078	0.46976002	0.89137026
N	-4.63649763	-1.18466652	-0.00028701
C	-3.54657919	0.76503093	0.00010437
H	-3.44404206	1.83956743	0.00025558
C	0.84997609	-0.84756325	0.00013892
C	1.73636908	0.30119831	0.00003607
C	1.66549316	-1.96718229	0.00007904
C	3.05464268	-0.23151555	-0.00005435
C	1.64265800	1.69590554	0.00001736
H	1.36837129	-3.00584897	0.00012179
C	4.20808739	0.54920739	-0.00015675
C	2.76713211	2.49505898	-0.00008649
C	4.04470886	1.92274426	-0.00017306
H	5.19218947	0.09565000	-0.00022219
H	2.63619361	3.57132255	-0.00009668
H	4.91405065	2.57097637	-0.00025256
N	2.96621624	-1.60756824	-0.00002117
C	4.09751146	-2.50830637	-0.00008404
H	4.70971644	-2.34787071	0.89054579
H	3.72665435	-3.53211612	-0.00004208
H	4.70959170	-2.34789879	-0.89080451
F	0.44918530	2.31095929	0.00010494

Table S48. Cartesian coordinates for the lowest energy configuration of *Z*-**10** in MeCN.

Z-**10** (MeCN)			
C	-1.81107279	-1.92589370	0.31576888
C	-0.77651399	-1.43285489	-0.45066581
C	-1.01582362	-0.02015147	-0.58780519
C	-2.18510153	0.26496237	0.16339736
C	-0.44158230	1.03808626	-1.29007229
C	-0.94515386	2.31594470	-1.20313517
C	-2.08343059	2.56463480	-0.41999245
C	-2.72550539	1.54731909	0.26408697
H	-0.46124895	3.11220119	-1.75727677
H	-2.47092904	3.57599846	-0.36544189
N	-2.63202468	-0.91352647	0.70979510
C	-3.78843083	-1.04684138	1.56723374
H	-4.68241199	-0.67782779	1.05878346
H	-3.92539651	-2.10059032	1.80570902
H	-3.64518737	-0.48556670	2.49403619
N	0.18962622	-2.26969205	-1.02676515
N	1.41709859	-2.00815814	-1.00331079
H	-1.99141037	-2.94312863	0.63203748
C	2.00079276	-0.98349776	-0.26545728
C	3.35711518	-0.63703246	-0.46760570
N	2.69354345	0.50465645	1.18461045
H	4.02511543	-1.02339638	-1.22533288
C	2.80121145	1.44629615	2.27490090
H	1.83635525	1.51143087	2.77521026
H	3.56077224	1.10694236	2.98154987
H	3.07841894	2.42696883	1.88427926
N	3.76223987	0.27116596	0.40014317
C	1.61492845	-0.20504481	0.83259053
H	0.69623352	-0.12700603	1.38921554
H	-3.61836721	1.73675992	0.84860905
F	0.61956380	0.80450362	-2.08579500

Table S49. Cartesian coordinates for the lowest energy configuration of *E*-**11** in MeCN.

E-**11** (MeCN)			
C	1.72992249	0.04751607	0.05190891
C	3.06454371	-0.40446393	-0.12645419
C	4.13583448	0.45236391	-0.35544357
C	3.87455173	1.81129805	-0.42412369
C	2.58175407	2.30425321	-0.22510672
C	1.53991087	1.43449978	0.04245569
C	0.92668613	-1.13974151	0.19190579
C	1.80391785	-2.21190779	0.15415399
H	5.14188318	0.06793852	-0.47946889
H	4.68389664	2.50823569	-0.60831312
H	2.39187203	3.37095675	-0.23259193
H	1.57458603	-3.26564937	0.22009627
N	3.06850136	-1.77968978	-0.04250812
N	-0.43418117	-1.35084074	0.22924077
N	-1.12866217	-0.35554374	-0.10797067
N	0.27648275	2.03550684	0.44977436
O	-0.18568585	2.92039350	-0.24560987
O	-0.20845366	1.64652893	1.49161645
C	4.24109073	-2.61709087	-0.17395398
H	4.70094737	-2.47263674	-1.15423892
H	4.96778124	-2.37624337	0.60510979
H	3.93621092	-3.65714934	-0.07062238
C	-2.49555868	-0.56858740	-0.07576371
C	-3.27970747	-1.70349343	0.24381841
C	-3.42351366	0.41615613	-0.39197651
H	-2.95017814	-2.68692056	0.54528270
N	-4.63402152	-0.14221714	-0.25541865
H	-3.28849979	1.44601272	-0.68597204
N	-4.56882192	-1.42995738	0.12861526
C	-5.92144817	0.47655243	-0.46939576
H	-6.45809121	-0.05033667	-1.26061197
H	-6.50546088	0.44196718	0.45208368
H	-5.76481726	1.51351158	-0.76308446

Table S50. Cartesian coordinates for the lowest energy configuration of *E*-**11** in DMSO.

E-**11** (DMSO)			
C	1.72871374	0.04748053	0.05158088
C	3.06390372	-0.40185157	-0.12825126
C	4.13315520	0.45706050	-0.35859039
C	3.86860996	1.81519119	-0.42713098
C	2.57511081	2.30538476	-0.22619234
C	1.53529485	1.43392051	0.04295568
C	0.92827783	-1.14134948	0.19302480
C	1.80747780	-2.21175379	0.15487696
H	5.13996837	0.07496032	-0.48368632
H	4.67614419	2.51392018	-0.61258877
H	2.38353965	3.37178250	-0.23394479
H	1.58064240	-3.26601032	0.22140107
N	3.07077239	-1.77682519	-0.04352894
N	-0.43239063	-1.35311568	0.23109918
N	-1.12595076	-0.35839972	-0.10960110
N	0.27149717	2.03240305	0.45404548
O	-0.19313537	2.91800233	-0.23893846
O	-0.20954707	1.64066879	1.49685326
C	4.24502080	-2.61113606	-0.17548339
H	4.70346519	-2.46655522	-1.15643528
H	4.97208805	-2.36770778	0.60246680
H	3.94322101	-3.65198507	-0.07074054
C	-2.49279166	-0.56996599	-0.07620920
C	-3.27768249	-1.70301218	0.24773991
C	-3.42003482	0.41407830	-0.39629767
H	-2.94890848	-2.68555428	0.55306203
N	-4.63077582	-0.14290454	-0.25777741
H	-3.28451436	1.44257460	-0.69454468
N	-4.56657867	-1.42908001	0.13128053
C	-5.91737610	0.47563411	-0.47478868
H	-6.45397813	-0.05385506	-1.26433789
H	-6.50208600	0.44502801	0.44641820
H	-5.76021607	1.51140274	-0.77241839

Table S51. Cartesian coordinates for the lowest energy configuration of *Z*-**11** in MeCN.

Z-**11** (MeCN)			
C	-1.09971847	0.02029570	-0.42985301
C	-2.33984389	0.33260295	0.19515535
C	-2.76144671	1.63108052	0.45228138
C	-1.92707278	2.67388135	0.07967626
C	-0.71615911	2.41997728	-0.56430738
C	-0.32603528	1.11921450	-0.83636496
C	-1.01642372	-1.41515448	-0.44725006
C	-2.21269829	-1.87534474	0.08102351
H	-3.71537059	1.82114371	0.93130713
H	-2.22517607	3.69879799	0.26665354
H	-0.08138682	3.23410902	-0.89078326
H	-2.52877663	-2.90015726	0.21330865
N	-2.98478842	-0.84532881	0.48991284
N	-0.08475074	-2.35633175	-0.90204839
N	1.15331940	-2.26520241	-0.72922148
N	0.86251031	0.94169211	-1.64965132
O	1.75927039	1.76095143	-1.55315636
O	0.87426153	-0.00428179	-2.41689463
C	1.78367613	-1.30172643	0.05490037
C	1.39580153	-0.50137397	1.13423504
C	3.17210236	-1.06171950	-0.06531811
N	2.50400200	0.12318924	1.55479416
H	3.85034525	-1.49188364	-0.78923614
N	3.59258285	-0.18625275	0.82871618
C	2.60772060	1.08657668	2.62585147
H	3.40855472	0.79113937	3.30501736
H	2.82307305	2.07480373	2.21369809
H	1.66100894	1.11410702	3.16310351
H	0.45569794	-0.36273731	1.64158689
C	-4.26654049	-0.95337884	1.15302278
H	-4.20783463	-0.54287651	2.16368244
H	-5.03027245	-0.41538627	0.58747031
H	-4.53841703	-2.00636230	1.20876956

Table S52. Cartesian coordinates for the lowest energy configuration of *Z*-**11** in DMSO.

Z-**11** (DMSO)			
C	-1.09949538	0.01921385	-0.42956315
C	-2.33994766	0.33364015	0.19356442
C	-2.76118715	1.63274531	0.44758527
C	-1.92587091	2.67393778	0.07355290
C	-0.71443817	2.41790911	-0.56848712
C	-0.32454419	1.11659219	-0.83741776
C	-1.01745603	-1.41642916	-0.44362639
C	-2.21462686	-1.87439090	0.08440167
H	-3.71540966	1.82443251	0.92538982
H	-2.22337385	3.69945373	0.25830449
H	-0.07865613	3.23128046	-0.89494009
H	-2.53168536	-2.89861243	0.21893110
N	-2.98603621	-0.84294214	0.49016617
N	-0.08647163	-2.35993354	-0.89448658
N	1.15154699	-2.26965016	-0.72119062
N	0.86541694	0.93716704	-1.64899989
O	1.76281972	1.75548996	-1.55017200
O	0.87591774	-0.00826940	-2.41718899
C	1.78246761	-1.30350363	0.05878644
C	1.39475739	-0.49656353	1.13328420
C	3.17141289	-1.06683810	-0.06081852
N	2.50350080	0.12817614	1.55154884
H	3.84981897	-1.50223204	-0.78148747
N	3.59245269	-0.18746510	0.82888153
C	2.60735776	1.09719463	2.61687390
H	3.40660974	0.80445435	3.29911556
H	2.82506632	2.08275472	2.19950381
H	1.65997322	1.12973113	3.15268351
H	0.45448795	-0.35348041	1.63915123
C	-4.26804585	-0.94798966	1.15238160
H	-4.20965756	-0.53522506	2.16215320
H	-5.03081199	-0.41041988	0.58507982
H	-4.54142903	-2.00047546	1.21046189

Table S53. Cartesian coordinates for the lowest energy configuration of *E*-**12** in MeCN.

E-**12** (MeCN)			
C	-1.76487956	1.14517394	-0.02299729
C	-0.75534326	0.17272853	-0.02126006
C	-1.40004706	-1.10945864	-0.00983772
C	-2.79036013	-0.84454833	-0.01326034
C	-0.95314197	-2.43964476	0.00629372
C	-1.89113914	-3.45486045	0.01584369
C	-3.26846787	-3.17228077	0.01253541
C	-3.73773060	-1.87200866	-0.00200949
H	0.10683041	-2.65371963	0.00903047
H	-1.56203303	-4.48864500	0.02765407
H	-3.97990851	-3.99148250	0.02611223
H	-4.80159439	-1.66617886	0.00713550
N	-2.98537961	0.51619617	-0.02467289
C	-4.29791102	1.13145169	-0.09155595
H	-4.62229525	1.47185703	0.89345747
H	-4.28034886	1.98148773	-0.76940423
H	-5.00246910	0.39252930	-0.47060667
N	0.57875491	0.50422295	-0.0002627
N	1.37236582	-0.47842327	-0.01253263
C	-1.65430357	2.61835341	0.04970190
O	-2.61716806	3.28311683	0.39189918
C	-0.35207852	3.27519741	-0.29553752
H	0.38448412	3.07035503	0.48548239
H	0.06085520	2.88840426	-1.22905670
H	-0.52372623	4.34998523	-0.36591809
C	2.70782659	-0.12605207	0.00531098
C	3.36241375	1.13138789	0.02943940
C	3.74387515	-1.05470175	-0.00240839
H	2.92691545	2.11978730	0.04102538
N	4.88437067	-0.35381551	0.01645993
H	3.72875184	-2.13403274	-0.02007952
N	4.67462030	0.97606683	0.03590561
C	6.23604775	-0.86307838	0.01815828
H	6.75348457	-0.53267826	0.92070201
H	6.19832355	-1.95105717	-0.00379092
H	6.76838667	-0.49723091	-0.86171767

Table S54. Cartesian coordinates for the lowest energy configuration of *Z*-**12** in MeCN.

Z-**12** (MeCN)			
C	-1.80919216	-0.85299368	-0.07431451
C	-0.70883590	-0.41257589	-0.80329922
C	-0.66621154	1.00841954	-0.69804736
C	-1.75230489	1.37753562	0.13360737
C	0.17863862	1.99057521	-1.23626666
C	-0.06494490	3.31093840	-0.92213730
C	-1.14151114	3.66748185	-0.08418515
C	-1.99432477	2.72134915	0.44874128
H	1.00509556	1.71254691	-1.88178803
H	0.57686028	4.08888545	-1.32163368
H	-1.30814207	4.71506461	0.14461009
H	-2.82195324	3.01831019	1.08220974
N	-2.42302102	0.24668296	0.50844945
C	-3.57076400	0.23483620	1.39473699
H	-4.46652032	-0.09589973	0.86816338
H	-3.39570409	-0.42961690	2.24015319
H	-3.72586083	1.24671020	1.76501203
N	0.09267584	-1.1802509	-1.66511047
N	1.33754633	-1.23454227	-1.54401166
C	2.04383625	-0.72759547	-0.46110447
C	1.73201784	-0.24804957	0.84417372
N	3.85625722	-0.26912630	0.68209028
H	0.77291882	-0.10519133	1.31635372
C	5.21638847	-0.05819256	1.12191748
H	5.89088009	-0.36364747	0.32365060
H	5.36868353	0.99850862	1.34849539
H	5.40801114	-0.65481105	2.01521594
C	-2.28659033	-2.22721246	0.11653033
O	-3.32523014	-2.46289749	0.71287041
C	-1.44878139	-3.33343272	-0.45408424
H	-1.26927424	-3.17461727	-1.52155531
H	-0.46836929	-3.36066551	0.03169675
H	-1.96438884	-4.28048714	-0.29400672
N	2.83503506	0.01526890	1.51727884
C	3.43812301	-0.72501417	-0.49714789
H	4.11689164	-1.01455315	-1.28513767

Table S55. Cartesian coordinates for the lowest energy configuration of *E*-**13** in MeCN.

E-**13** (MeCN)			
C	-1.31851853	-1.38795525	0.00029012
C	-0.45314750	-0.32880889	0.00016565
C	-1.25110026	0.88313190	0.00009985
C	-2.60118675	0.47061149	0.00019007
C	-0.94562676	2.24568109	-0.00001929
C	-1.98881245	3.16025269	-0.00004805
C	-3.32026721	2.73338863	0.00004364
C	-3.64996221	1.38385183	0.00016509
H	0.08636638	2.56970926	-0.00008858
H	-1.76872949	4.22275541	-0.00014316
H	-4.11820418	3.46867272	0.00001942
H	-4.67785080	1.05256018	0.00023184
N	-2.62065511	-0.93530118	0.00032243
C	-3.76615583	-1.74673687	0.00033563
N	0.90943753	-0.54152311	0.00010325
N	1.60911133	0.50669889	-0.00004008
H	-1.07967716	-2.43929612	0.00034318
O	-4.86661403	-1.24669698	0.00001182
C	-3.52581829	-3.22340328	0.00030467
H	-4.49288415	-3.72268418	0.00045737
H	-2.95828001	-3.52530707	-0.88423677
H	-2.95793435	-3.52537502	0.88458924
C	2.97397695	0.28563483	-0.00016207
C	3.94356790	1.31103047	-0.00034921
C	3.71682577	-0.89557017	-0.00015647
H	3.78088795	2.38017249	-0.00041636
N	5.00248399	-0.52576461	-0.00032636
H	3.41712171	-1.93139629	-0.00004697
N	5.16799610	0.80994804	-0.00044175
C	6.16394978	-1.38580989	-0.00036120
H	6.76330393	-1.19212070	0.89099778
H	5.83053774	-2.42226824	-0.00040337
H	6.76328996	-1.19204422	-0.89171124

Table S56. Cartesian coordinates for the lowest energy configuration of *E*-**13** in DMSO.

E-**13** (DMSO)			
C	1.31839123	-1.38726644	0.00013175
C	0.45308482	-0.32789827	0.00012685
C	1.25110071	0.88364151	0.00002632
C	2.60119413	0.47060713	0.00001022
C	0.94562956	2.24608061	-0.00005400
C	1.98862148	3.16054917	-0.00016001
C	3.31983893	2.73360486	-0.00018789
C	3.64960392	1.38426166	-0.00010322
H	-0.08635085	2.57027281	-0.00003908
H	1.76860573	4.22306664	-0.00022579
H	4.11783732	3.46884018	-0.00027146
H	4.67776371	1.05408229	-0.00011109
N	2.62014063	-0.93502447	0.00012594
C	3.76571638	-1.74791041	0.00019991
N	-0.90923614	-0.53997735	0.00019867
N	-1.60888396	0.50833751	0.00016493
H	1.07883375	-2.43844677	0.00018229
O	4.86646832	-1.24952951	-0.00054489
C	3.52248492	-3.22398594	-0.00061395
H	4.48833598	-3.72573908	-0.00139686
H	2.95487845	-3.52609538	0.88382633
H	2.95395967	-3.52509308	-0.88480815
C	-2.97354297	0.28676902	0.00015821
C	-3.94389299	1.31128443	0.00011731
C	-3.71539434	-0.89498518	0.00020003
H	-3.78217717	2.38061216	0.00007934
N	-5.00112304	-0.52633143	0.00019721
H	-3.41490719	-1.93058061	0.00024034
N	-5.16782289	0.80911300	0.00014634
C	-6.16134221	-1.38729807	0.00021366
H	-6.76091469	-1.19450673	-0.89122867
H	-5.82702670	-2.42347629	0.00039461
H	-6.76106821	-1.19424532	0.89149458

Table S57. Cartesian coordinates for the lowest energy configuration of *Z*-**13** in MeCN.

Z-**13** (MeCN)			
C	1.46608058	-1.54097060	-0.40426351
C	0.34792495	-1.04325795	-0.99932046
C	0.43804201	0.40202398	-0.94916349
C	1.63474085	0.71020613	-0.26941546
C	-0.36878368	1.42804048	-1.44321439
C	0.02526052	2.73977229	-1.23188181
C	1.20752414	3.03164626	-0.54126096
C	2.03238045	2.02691365	-0.05324615
H	-1.28377375	1.20179092	-1.97913597
H	-0.59035629	3.55149829	-1.60486554
H	1.49294883	4.06688539	-0.38630696
H	2.95027070	2.25211976	0.46966645
N	2.25006458	-0.49992000	0.07652820
C	3.44927475	-0.64361399	0.78345553
N	-0.59557640	-1.84375058	-1.66693739
N	-1.82850066	-1.73447570	-1.46524658
H	1.75470186	-2.57078706	-0.26925010
C	-2.40500199	-0.97132654	-0.46025912
C	-3.80320920	-0.75348900	-0.43218293
N	-3.02586387	0.11865924	1.33261791
H	-4.52896459	-1.06343797	-1.17158281
C	-3.07169200	0.82533682	2.59271356
H	-2.06279663	0.87968092	2.99849309
H	-3.45768490	1.83334054	2.43131629
H	-3.72200559	0.29161477	3.28772759
N	-4.16727396	-0.08288645	0.64276390
C	-1.94661380	-0.37921765	0.72475425
H	-0.97143389	-0.30154775	1.17540264
O	4.05759387	0.33118052	1.16312452
C	3.90478769	-2.04895165	1.02458196
H	4.83896497	-2.01077254	1.58192265
H	3.15992953	-2.60827400	1.59719353
H	4.06520942	-2.57361448	0.07855860

Table S58. Cartesian coordinates for the lowest energy configuration of *Z*-**13** in DMSO.

Z-**13** (DMSO)			
C	1.46898583	-1.54102290	-0.40211684
C	0.34892170	-1.04798469	-0.99756176
C	0.43495024	0.39746733	-0.95088986
C	1.63146279	0.71045681	-0.27304965
C	-0.37565849	1.41993845	-1.44584899
C	0.01469918	2.73311999	-1.23774109
C	1.19689113	3.02988212	-0.54935330
C	2.02530417	2.02878124	-0.06036292
H	-1.29052099	1.19006597	-1.98049608
H	-0.60386015	3.54226754	-1.61148149
H	1.47936353	4.06630915	-0.39688786
H	2.94306487	2.25809736	0.46088820
N	2.25024041	-0.49689688	0.07535347
C	3.45001337	-0.63609240	0.78352462
N	-0.59389867	-1.85256037	-1.66066127
N	-1.82676794	-1.74273959	-1.45844582
H	1.76002377	-2.56980548	-0.26442650
C	-2.40192059	-0.97523746	-0.45615156
C	-3.79959393	-0.75480172	-0.42815920
N	-3.01985435	0.12148890	1.33339747
H	-4.52638912	-1.06570207	-1.16618681
C	-3.06379721	0.83181915	2.59104401
H	-2.05480087	0.88503359	2.99674527
H	-3.44732928	1.84034992	2.42686473
H	-3.71524864	0.30177538	3.28784034
N	-4.16187742	-0.08002205	0.64472645
C	-1.94185608	-0.38007846	0.72673258
H	-0.96646697	-0.30335480	1.17720961
O	4.05651723	0.34037207	1.16045322
C	3.90598821	-2.04042989	1.02863728
H	4.83964709	-2.00112338	1.58676969
H	3.16124538	-2.59883600	1.60233192
H	4.06789740	-2.56784928	0.08439108

8. TEM images and ROS detection

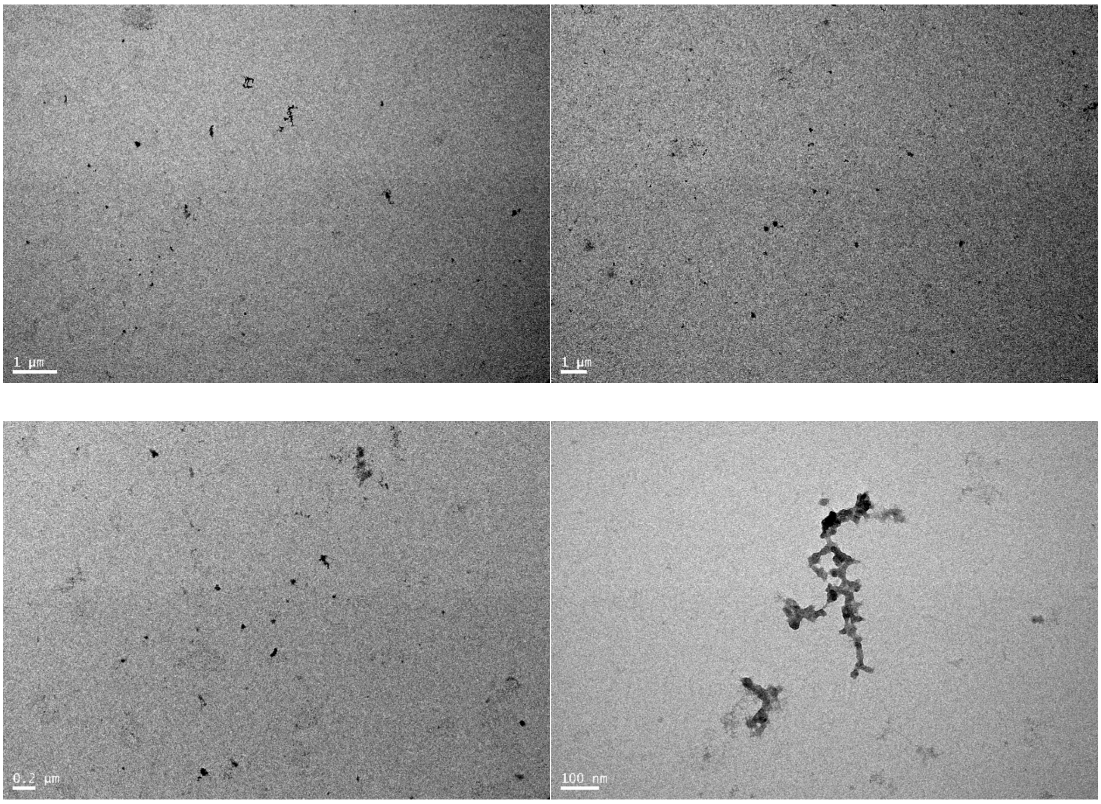

Figure S45. The TEM images of *E*–**5-PS**.

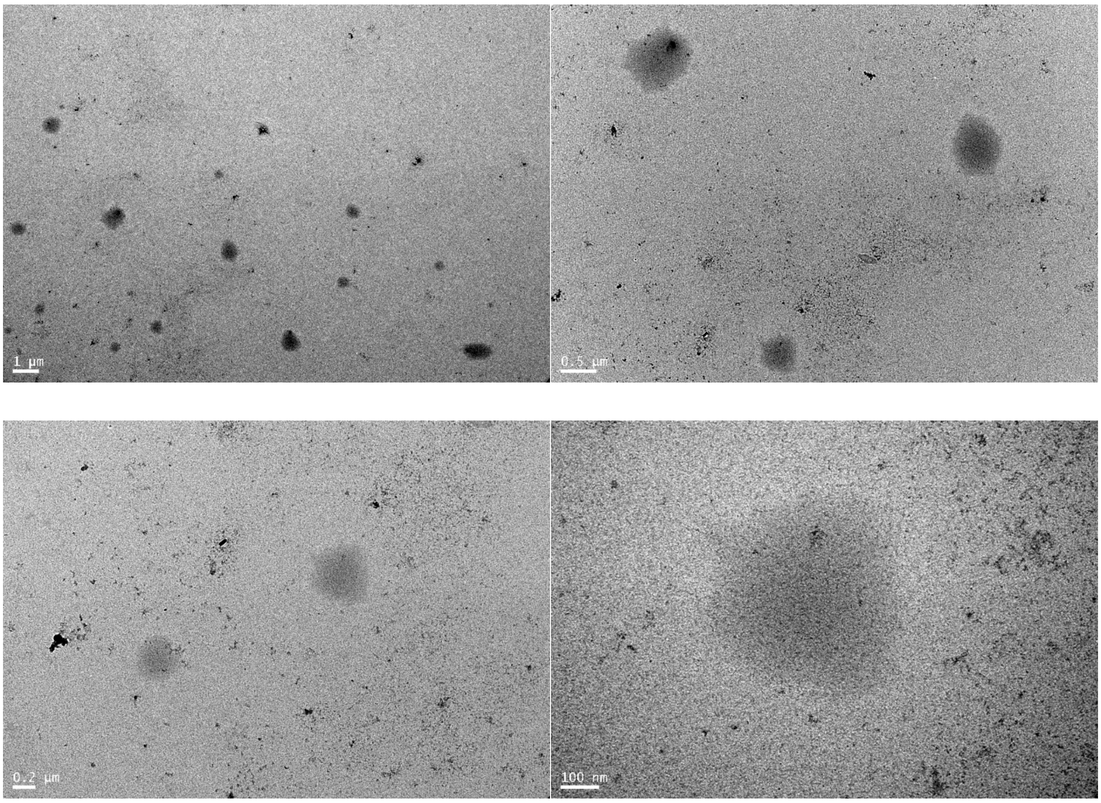

Figure S46. The TEM images of *Z*–**5-PS**.

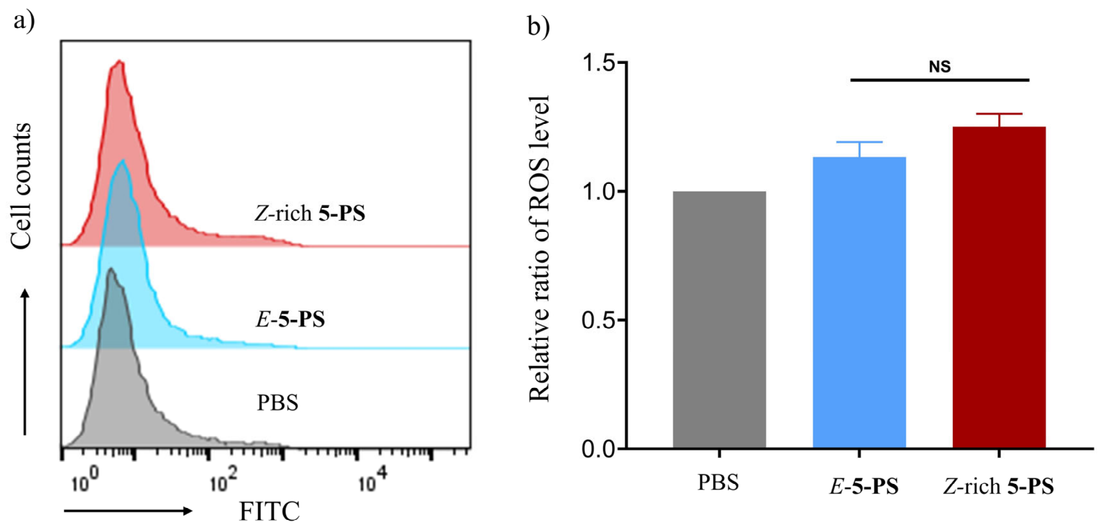

Figure S47. (a-b) Intracellular ROS levels were measured by DCFH-DA staining with flow cytometry. HepG2 cells were treated with the **5-PS** (50 μM) for 15 min and irradiated with a 400 nm laser (15 min) before being incubated with DCFH-DA (10 μM, 15 min). λex = 488 nm, λem = 525 ± 20 nm.

9. References

(1) Frisch, M. J.; Trucks, G. W.; Schlegel, H. B.; Scuseria, G. E.; Robb, M. A.; Cheeseman, J. R.; Scalmani, G.; Barone, V.; Petersson, G. A.; Nakatsuji, H.; Li, X.; Caricato, M.; Marenich, A. V.; Bloino, J.; Janesko, B. G.; Gomperts, R.; Mennucci, B.; Hratchian, H. P.; Ortiz, J. V.; Izmaylov, A. F.; Sonnenberg, J. L.; Williams; Ding, F.; Lipparini, F.; Egidi, F.; Goings, J.; Peng, B.; Petrone, A.; Henderson, T.; Ranasinghe, D.; Zakrzewski, V. G.; Gao, J.; Rega, N.; Zheng, G.; Liang, W.; Hada, M.; Ehara, M.; Toyota, K.; Fukuda, R.; Hasegawa, J.; Ishida, M.; Nakajima, T.; Honda, Y.; Kitao, O.; Nakai, H.; Vreven, T.; Throssell, K.; Montgomery Jr., J. A.; Peralta, J. E.; Ogliaro, F.; Bearpark, M. J.; Heyd, J. J.; Brothers, E. N.; Kudin, K. N.; Staroverov, V. N.; Keith, T. A.; Kobayashi, R.; Normand, J.; Raghavachari, K.; Rendell, A. P.; Burant, J. C.; Iyengar, S. S.; Tomasi, J.; Cossi, M.; Millam, J. M.; Klene, M.; Adamo, C.; Cammi, R.; Ochterski, J. W.; Martin, R. L.; Morokuma, K.; Farkas, O.; Foresman, J. B.; Fox, D. J. Gaussian 16 Revision A.03, Gaussian, Inc. Wallingford, CT, 2016.

(2) (a) Adamo, C.; Barone, V. Toward reliable density functional methods without adjustable parameters: the PBE0 model. *J.Chem. Phys.* **1999**, *110*, 6158−6170; (b) Grimme, S.; Antony, J.; Ehrlich, S.; Krieg, H. A Consistent and Accurate ab Initio Parametrization of Density Functional Dispersion Correction (DFT-D) for the 94 Elements H-Pu. *J. Chem. Phys.* **2010**, *132*, 154104; (c) Grimme, S.; Ehrlich, S.; Goerigk, L. Effect of the Damping Function in Dispersion Corrected Density Functional Theory. *J. Comput. Chem.* **2011**, *32*, 1456−1465; (d) Krishnan, R.; Binkley, J. S.; Seeger, R.; Pople, J. A. Self-Consistent Molecular Orbital Methods. XX. A Basis Set for Correlated Wave Functions. *J. Chem. Phys.* **1980**, *72*, 650−654.

(3) Marenich, A. V.; Cramer, C. J.; Truhlar, D. G., Universal Solvation Model Based on Solute Electron Density and on a Continuum Model of the Solvent Defined by the Bulk Dielectric Constant and Atomic Surface Tensions. *J. Phys. Chem. B* **2009**, *113*, 6378−6396.

(4) (a) Schwabe, T.; Grimme, S., Towards Chemical Accuracy for the Thermodynamics of Large Molecules: New Hybrid Density Functionals Including Non-Local Correlation Effects. *Phys. Chem. Chem. Phys.* **2006**, *8*, 4398; (b) Weigend, F.; Ahlrichs, R. Balanced Basis Sets of Split Valence, Triple Zeta Valence and Quadruple Zeta Valence Quality for H to Rn: Design and Assessment of Accuracy. *Phys. Chem. Chem. Phys.* **2005**, *7*, 3297.

(5) Zhao, Y.; Schultz, N. E.; Truhlar, D. G., Design of density functionals by combining the method of constraint satisfaction with parametrization for thermochemistry, thermochemical kinetics, and noncovalent interactions. *J. Chem. Theory Comput.* **2006**, *2*, 364−382.

(6) Noodleman, L., Valence Bond Description of Antiferromagnetic Coupling in Transition Metal Dimers. *J. Chem. Phys.* **1981**, *74*, 5737–5743.

(7) Johnson, E. R.; Keinan, S.; Mori-Sánchez, P.; Contreras-García, J.; Cohen, A. J.; Yang, W., Revealing Noncovalent Interactions. *J. Am. Chem. Soc.* **2010**, *132*, 6498–6506.

(8) Lu, T.; Chen, F., Multiwfn: A Multifunctional Wavefunction Analyzer. *J Comput. Chem.* **2011**, *33*, 580−592.

(9) Humphrey, W.; Dalke, A.; Schulten, K., VMD: Visual Molecular Dynamics. *J Mol. Graph.* **1996**, 14, 33−38.

(10) (a) Zhang, Z. Y.; He, Y.; Zhou, Y.; Yu, C.; Han, L.; Li, T., Pyrazolylazophenyl Ether-based Photoswitches: Facile Synthesis, (near-)Quantitative Photoconversion, Long Thermal Half-life, Easy Functionalization, and Versatile Applications in Light-Responsive Systems. *Chem. Eur. J.* **2019,** *25*, 13402–13410. (b) He, Y.; Shangguan, Z.; Zhang, Z. Y.; Xie, M.; Yu, C.; Li, T., Azobispyrazole Family as Photoswitches Combining (Near-) Quantitative Bidirectional Isomerization and Widely Tunable Thermal Half-lives from Hours to Years**. *Angew. Chem. Inter. Ed.* **2021,** *60*, 16539–16546. (c) Dang, T.; Dong, D.; Zhang, J.; He, Y.; Zhang, Z. Y.; Li, T., Thiazolylazopyrazoles as Nonsymmetric Bis-Heteroaryl Azo Wwitches: High-Yield Visible-Light Photoisomerization and Increased *Z*-Isomer Stability by *o*-Carbonylation. *Angew. Chem. Inter. Ed.* **2023**, *62*, e202301992.

(11) Zimmerman, G.; Chow, L. Y.; U. J. Paik, The Photochemical Isomerization of Azobenzene. *J. Am. Chem. Soc.* **1958**, *80*, 3528–3531.
